# Supplementary figures and images for: Catalytic activity and autoprocessing of murine caspase-11 mediate noncanonical inflammasome assembly in response to cytosolic LPS (part 1 of 2)
Source: eLife. 2024 Jan 17;13:e83725. doi: 10.7554/eLife.83725 (PMC10794067; doi:10.7554/eLife.83725)

Fig 1-source data 1 (1B)

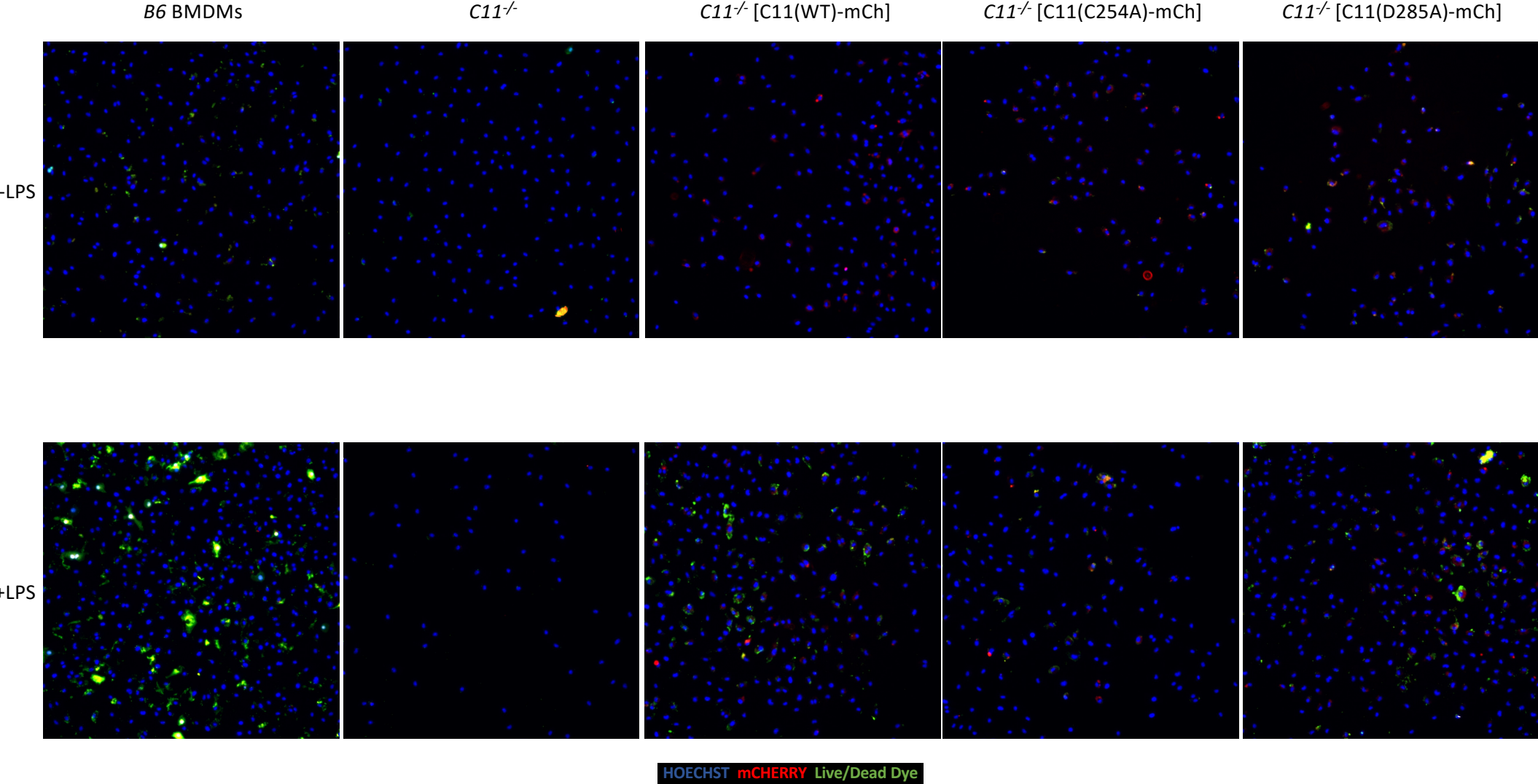

Supplement: Figure 1—source data 1. — Caspase-11-mediated membrane pore formation was assessed by imaging primary bone marrow-derived macrophages (BMDMs) after uptake of Live/Dead green fluorescent dye. Nuclei are stained with Hoechst. [file elife-83725-fig1-data1.zip › figure1-sourcedata1.pdf]

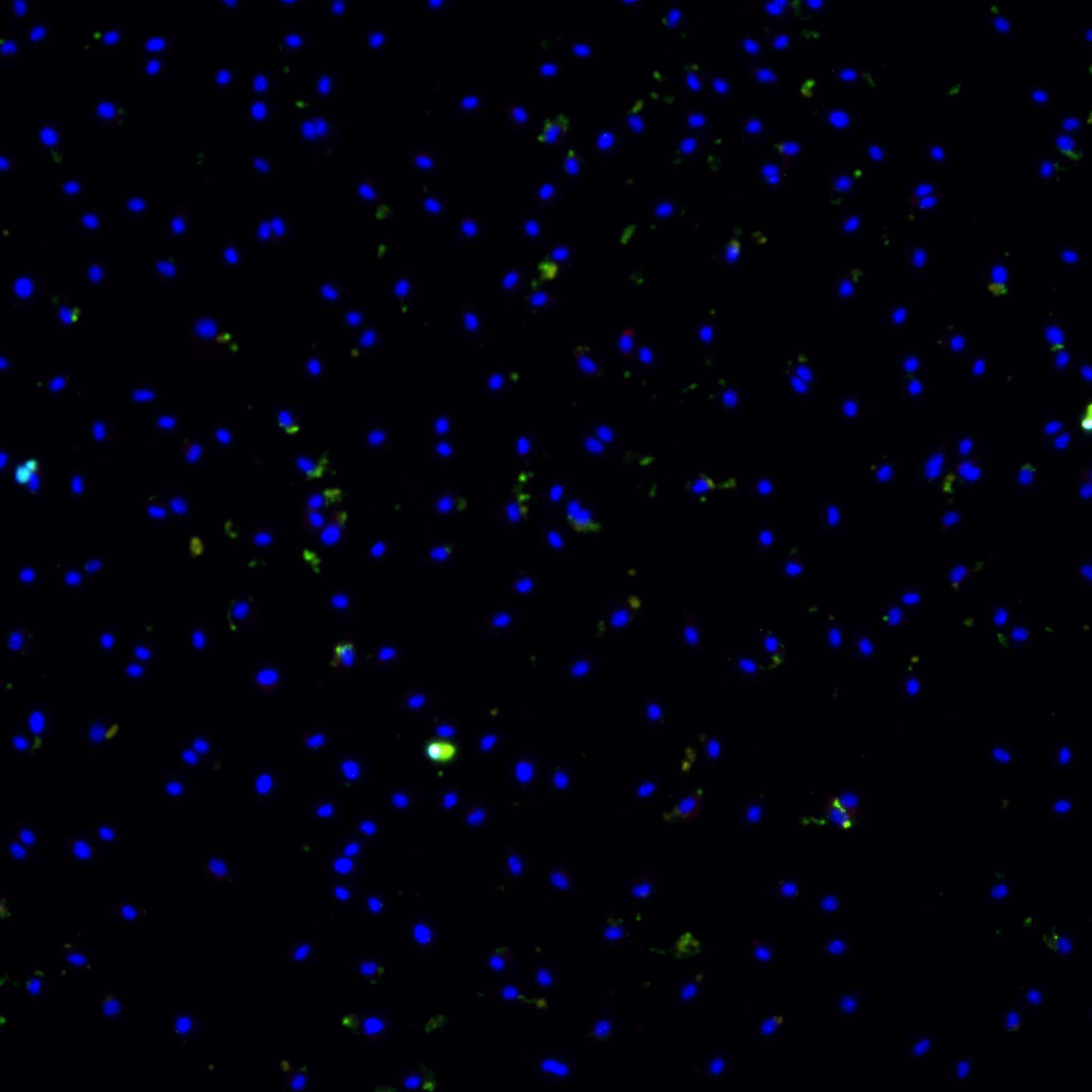

Supplement: Figure 1—source data 1. — Caspase-11-mediated membrane pore formation was assessed by imaging primary bone marrow-derived macrophages (BMDMs) after uptake of Live/Dead green fluorescent dye. Nuclei are stained with Hoechst. [file elife-83725-fig1-data1.zip › No_LPS_B6.tif]

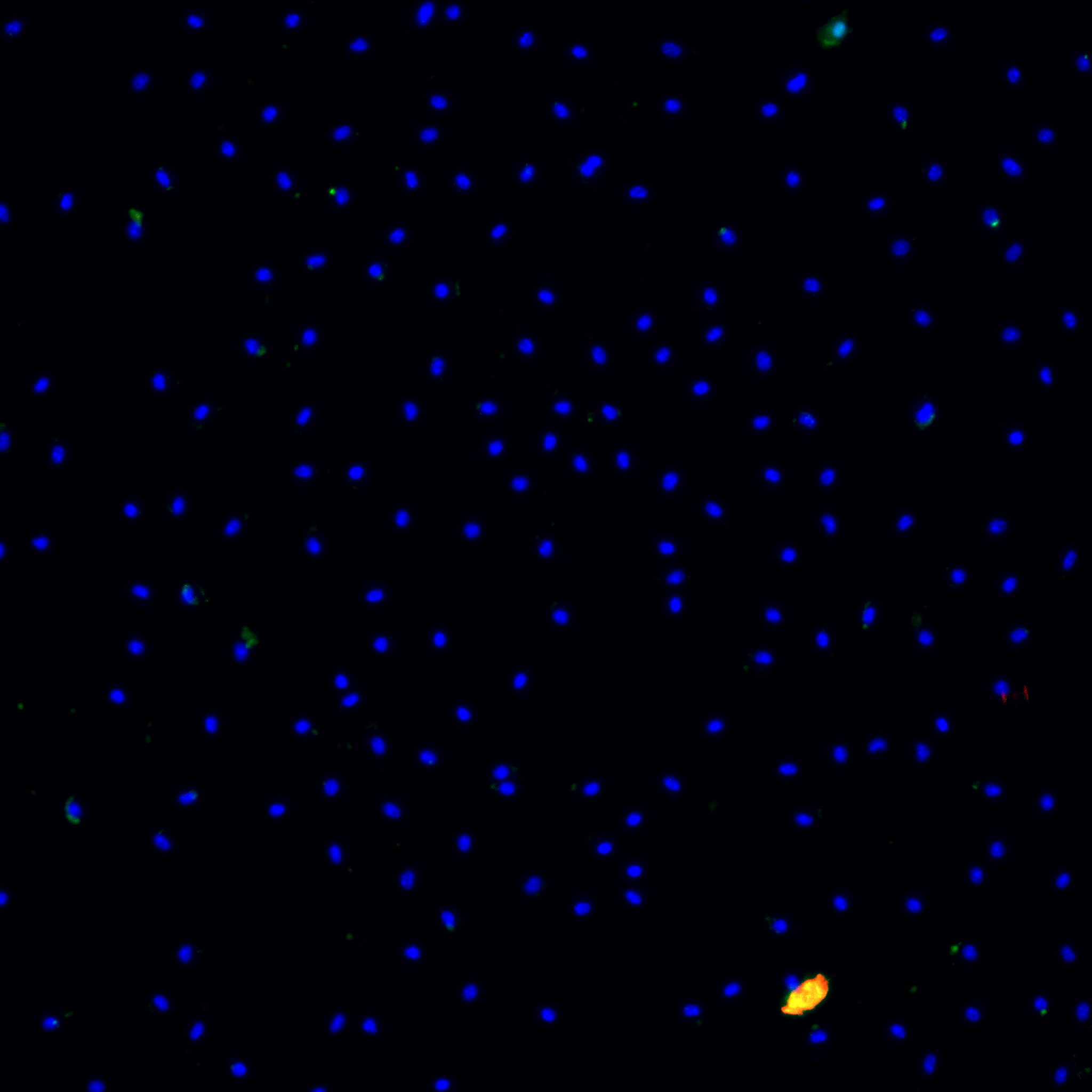

Supplement: Figure 1—source data 1. — Caspase-11-mediated membrane pore formation was assessed by imaging primary bone marrow-derived macrophages (BMDMs) after uptake of Live/Dead green fluorescent dye. Nuclei are stained with Hoechst. [file elife-83725-fig1-data1.zip › No_LPS_C11-:-.tif]

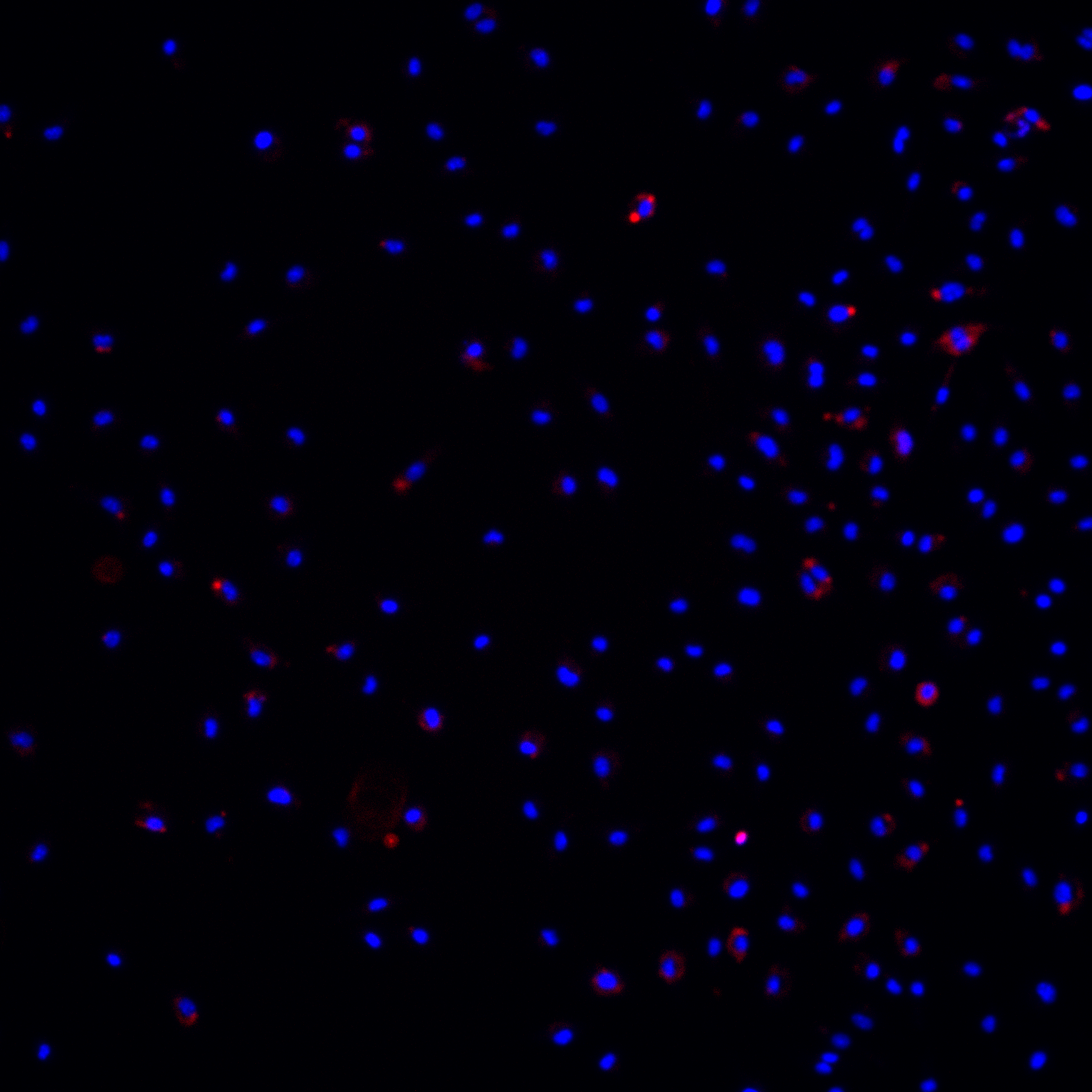

Supplement: Figure 1—source data 1. — Caspase-11-mediated membrane pore formation was assessed by imaging primary bone marrow-derived macrophages (BMDMs) after uptake of Live/Dead green fluorescent dye. Nuclei are stained with Hoechst. [file elife-83725-fig1-data1.zip › No_LPS_C11WT-mCh.tif]

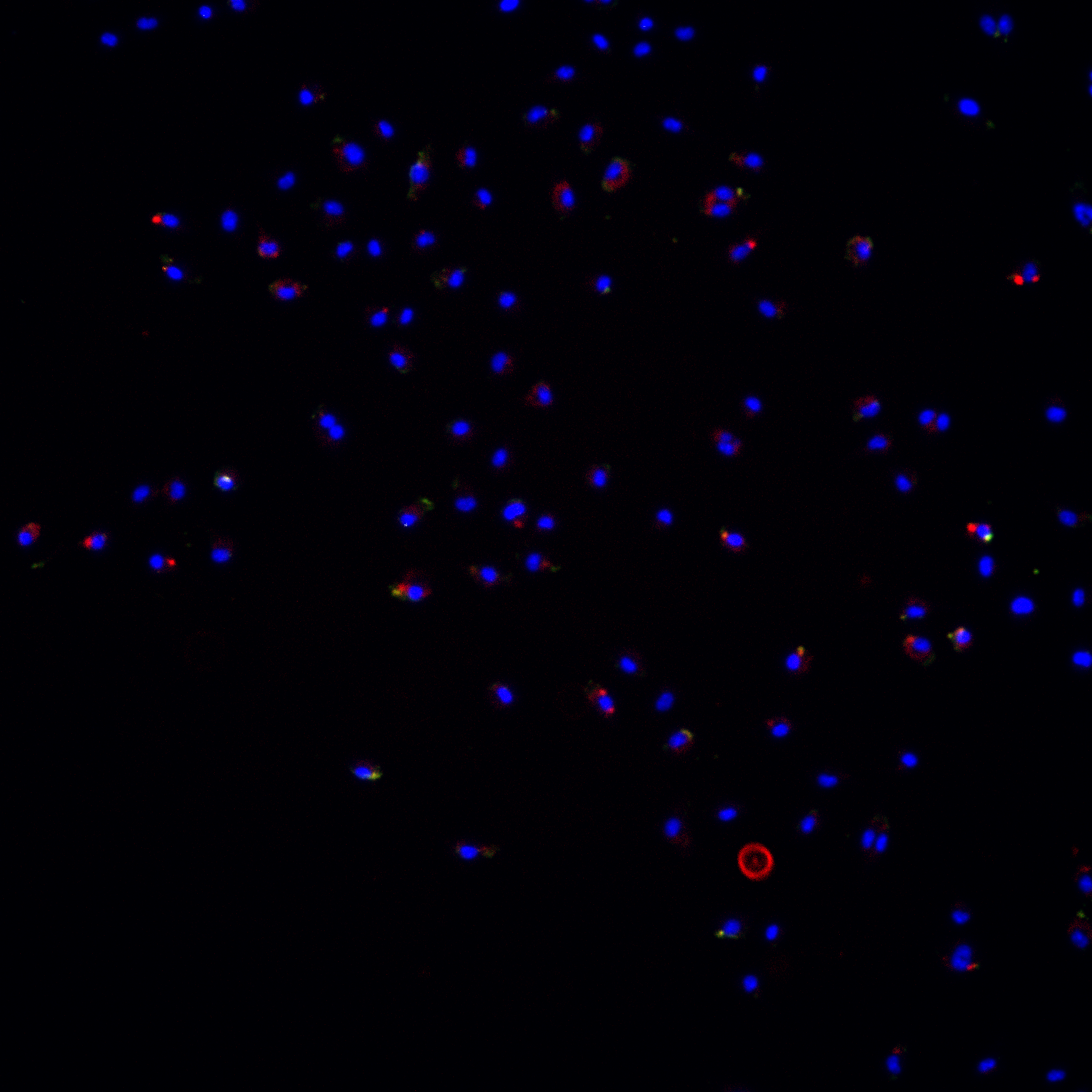

Supplement: Figure 1—source data 1. — Caspase-11-mediated membrane pore formation was assessed by imaging primary bone marrow-derived macrophages (BMDMs) after uptake of Live/Dead green fluorescent dye. Nuclei are stained with Hoechst. [file elife-83725-fig1-data1.zip › No_LPS_C254A-mCh.tif]

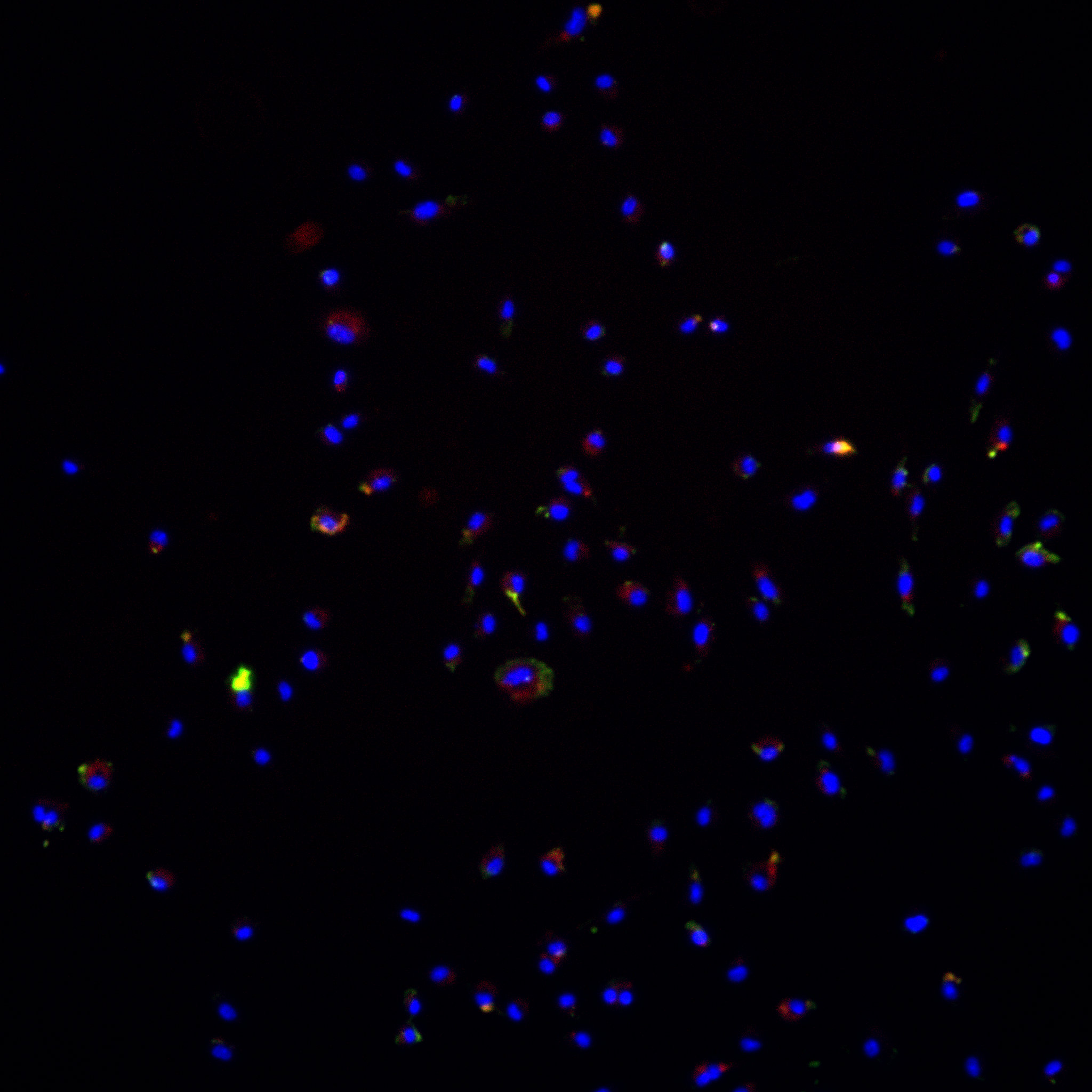

Supplement: Figure 1—source data 1. — Caspase-11-mediated membrane pore formation was assessed by imaging primary bone marrow-derived macrophages (BMDMs) after uptake of Live/Dead green fluorescent dye. Nuclei are stained with Hoechst. [file elife-83725-fig1-data1.zip › No_LPS_D285A-mCh.tif]

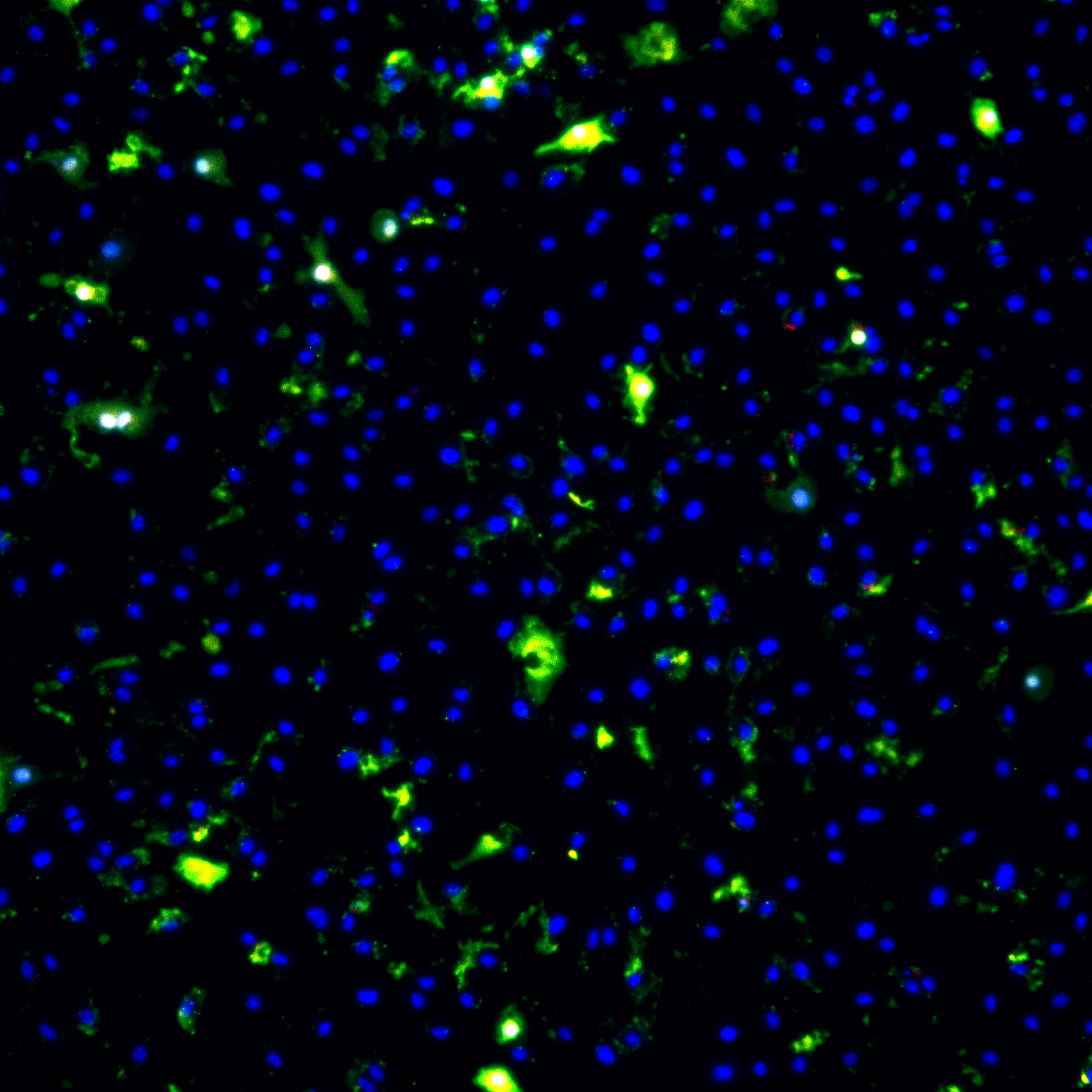

Supplement: Figure 1—source data 1. — Caspase-11-mediated membrane pore formation was assessed by imaging primary bone marrow-derived macrophages (BMDMs) after uptake of Live/Dead green fluorescent dye. Nuclei are stained with Hoechst. [file elife-83725-fig1-data1.zip › Yes_LPS_B6.tif]

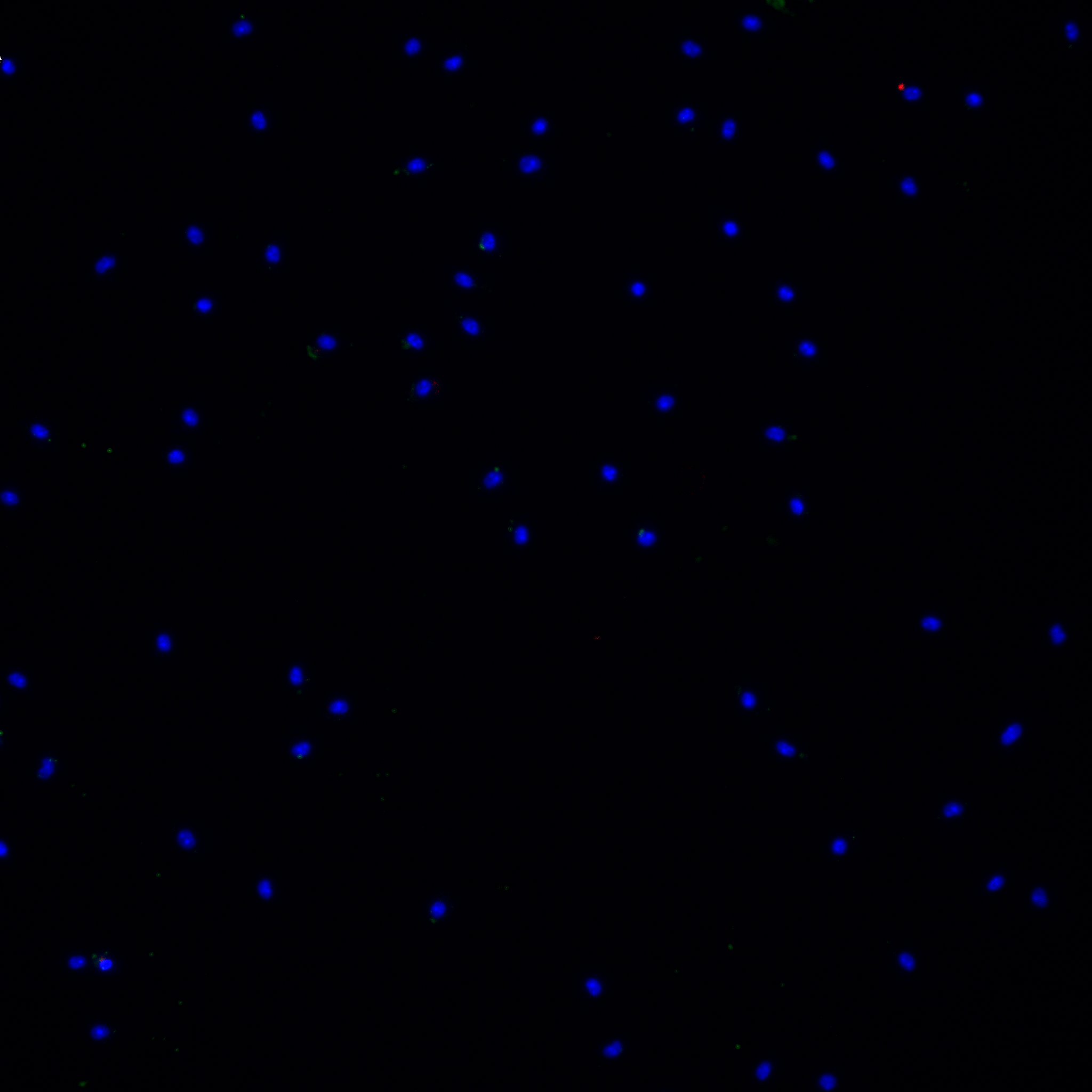

Supplement: Figure 1—source data 1. — Caspase-11-mediated membrane pore formation was assessed by imaging primary bone marrow-derived macrophages (BMDMs) after uptake of Live/Dead green fluorescent dye. Nuclei are stained with Hoechst. [file elife-83725-fig1-data1.zip › Yes_LPS_C11-:-.tif]

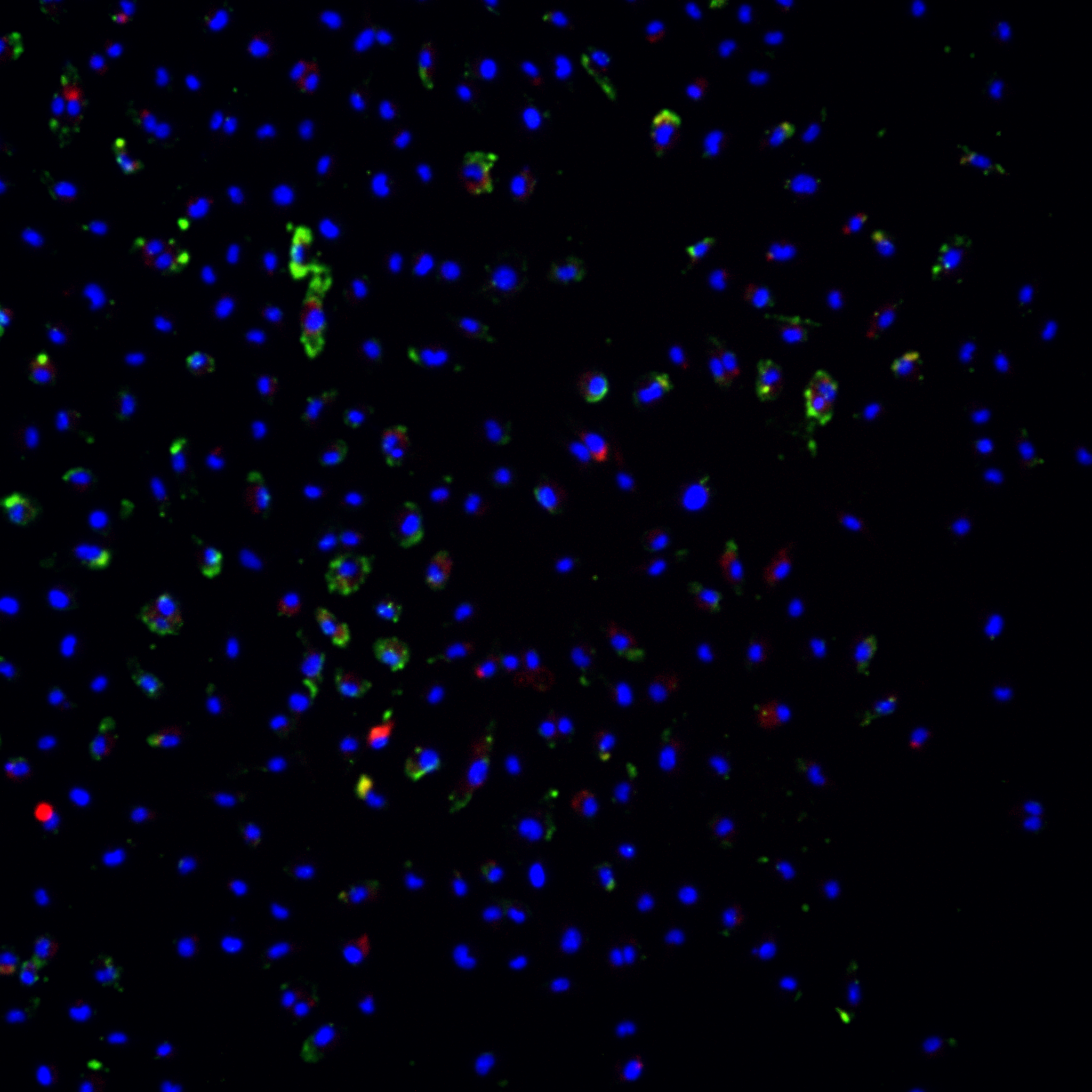

Supplement: Figure 1—source data 1. — Caspase-11-mediated membrane pore formation was assessed by imaging primary bone marrow-derived macrophages (BMDMs) after uptake of Live/Dead green fluorescent dye. Nuclei are stained with Hoechst. [file elife-83725-fig1-data1.zip › Yes_LPS_C11WT-mCh.tif]

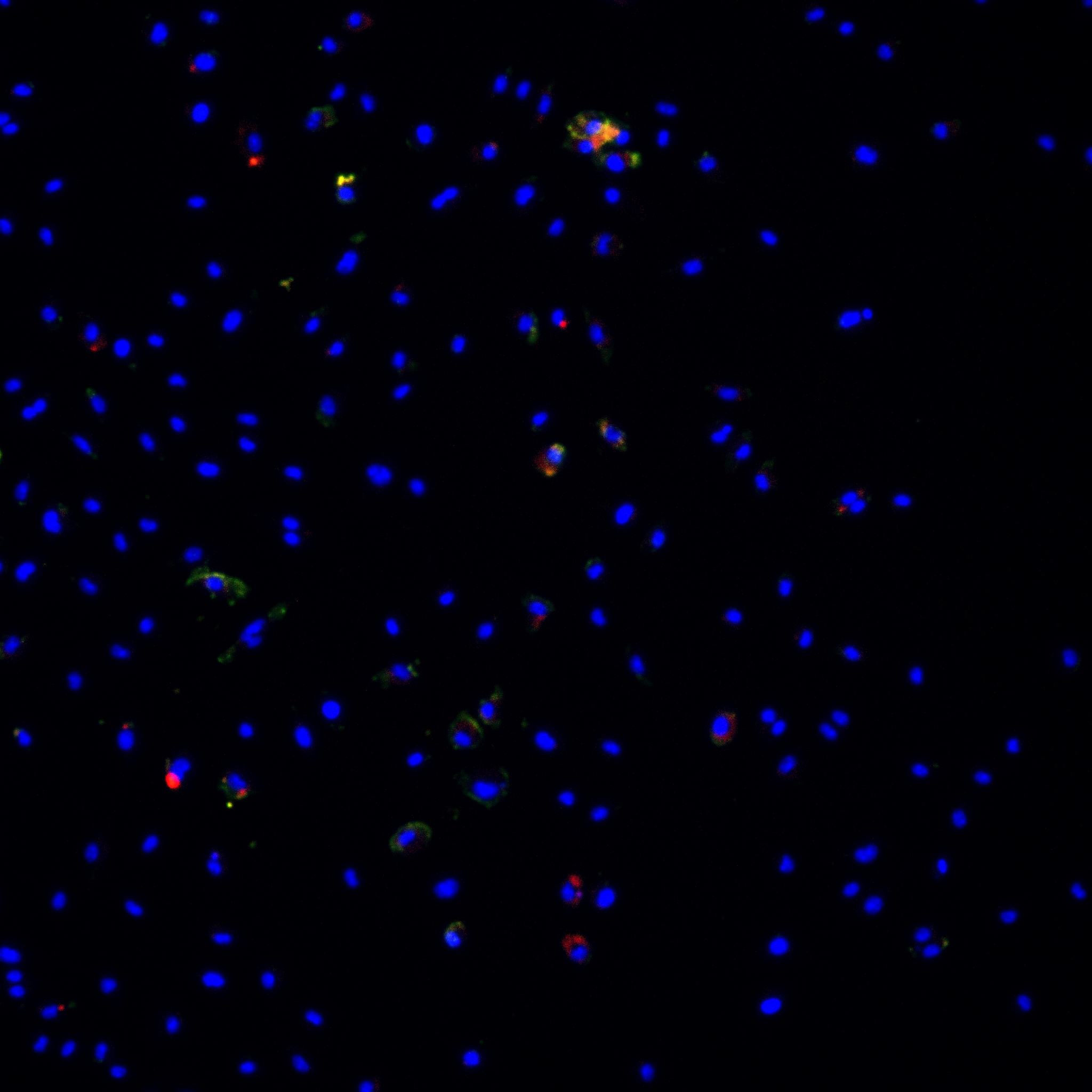

Supplement: Figure 1—source data 1. — Caspase-11-mediated membrane pore formation was assessed by imaging primary bone marrow-derived macrophages (BMDMs) after uptake of Live/Dead green fluorescent dye. Nuclei are stained with Hoechst. [file elife-83725-fig1-data1.zip › Yes_LPS_C254A-mCh.tif]

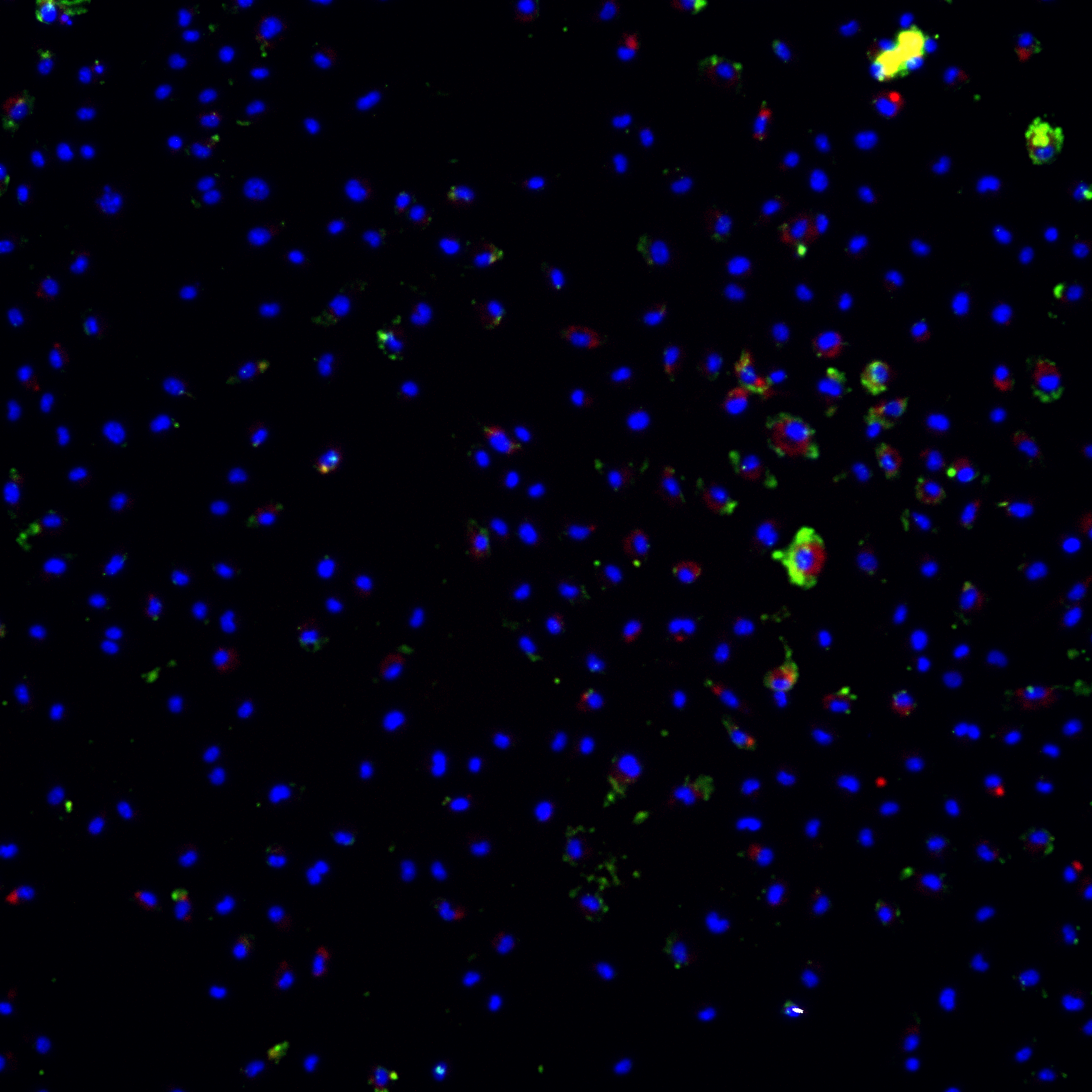

Supplement: Figure 1—source data 1. — Caspase-11-mediated membrane pore formation was assessed by imaging primary bone marrow-derived macrophages (BMDMs) after uptake of Live/Dead green fluorescent dye. Nuclei are stained with Hoechst. [file elife-83725-fig1-data1.zip › Yes_LPS_D285A-mCh.tif]

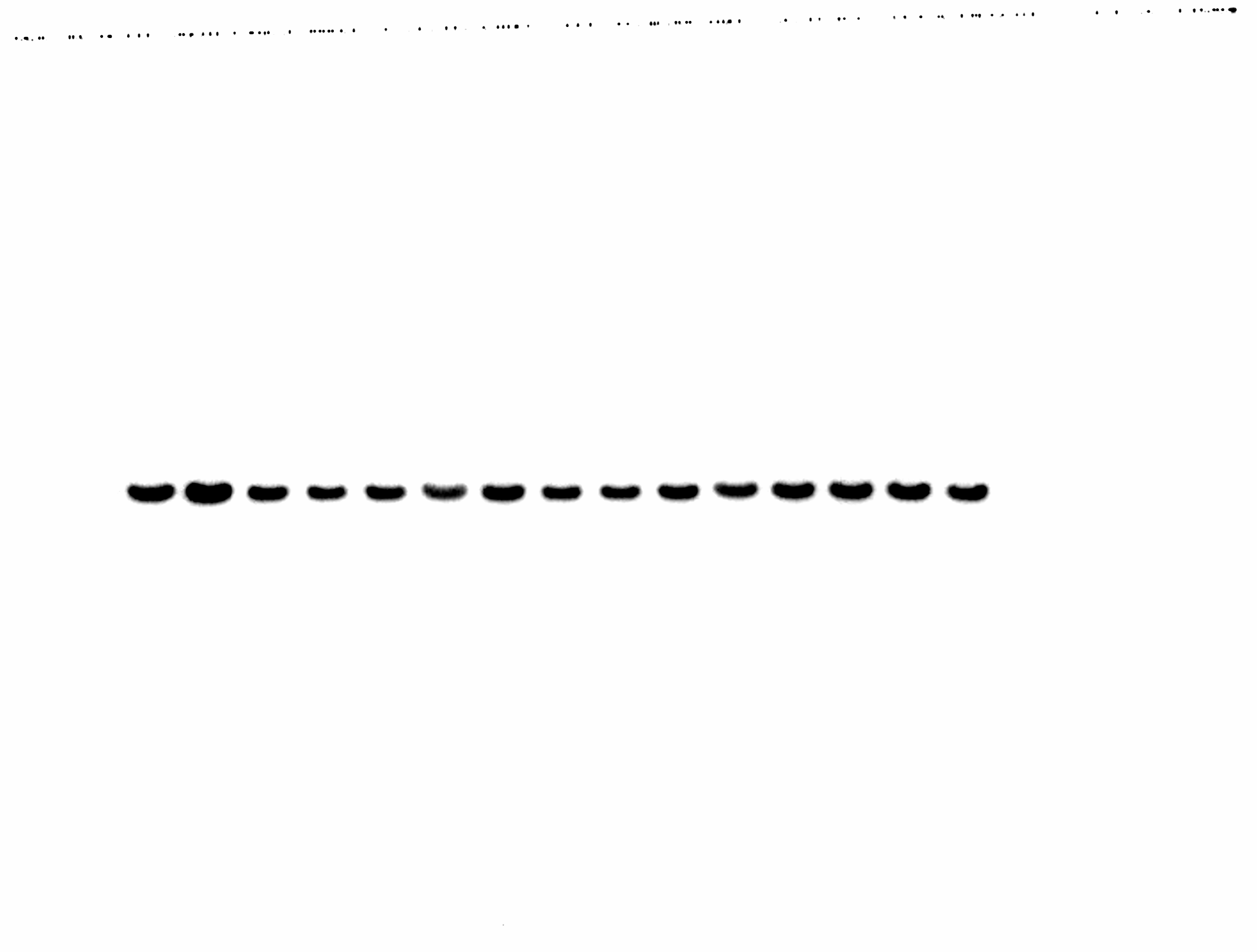

Supplement: Figure 1—source data 2. — Casp11-mCherry expression and gasdermin D (GSDMD) processing in response to lipopolysaccharide (LPS) transfection was assessed by western blotting for mCherry and GSDMD as indicated. β-actin was used as a loading control. [file elife-83725-fig1-data2.zip › Actin_hires.tif]

1C.

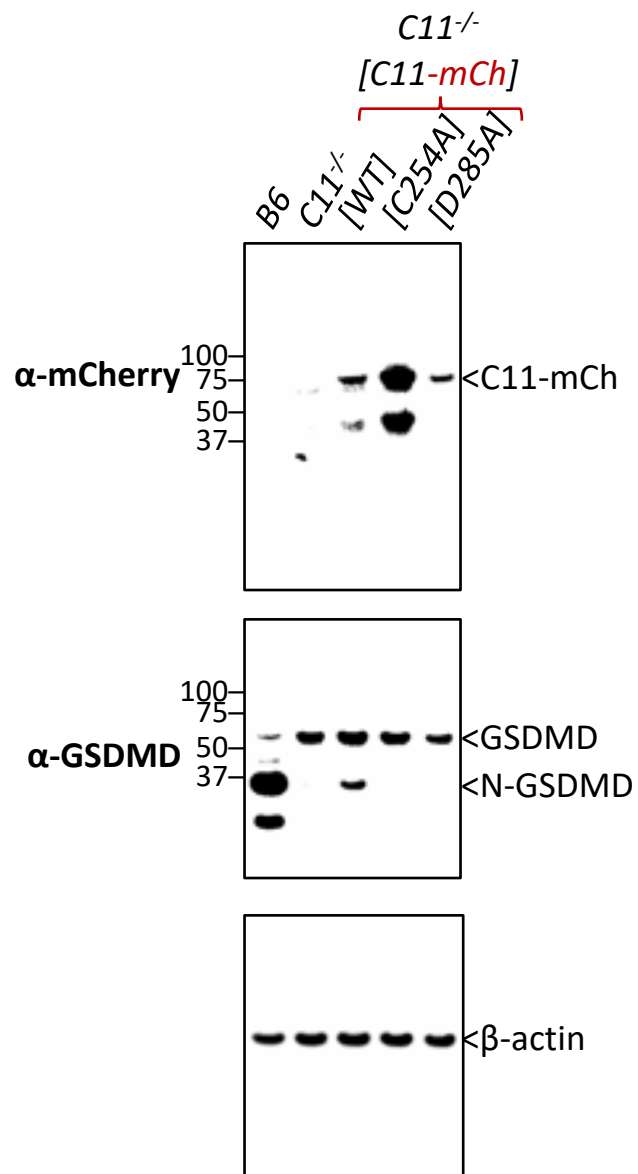

Supplement: Figure 1—source data 2. — Casp11-mCherry expression and gasdermin D (GSDMD) processing in response to lipopolysaccharide (LPS) transfection was assessed by western blotting for mCherry and GSDMD as indicated. β-actin was used as a loading control. [file elife-83725-fig1-data2.zip › Figure_1C_labeled.pdf]

## Slide 1
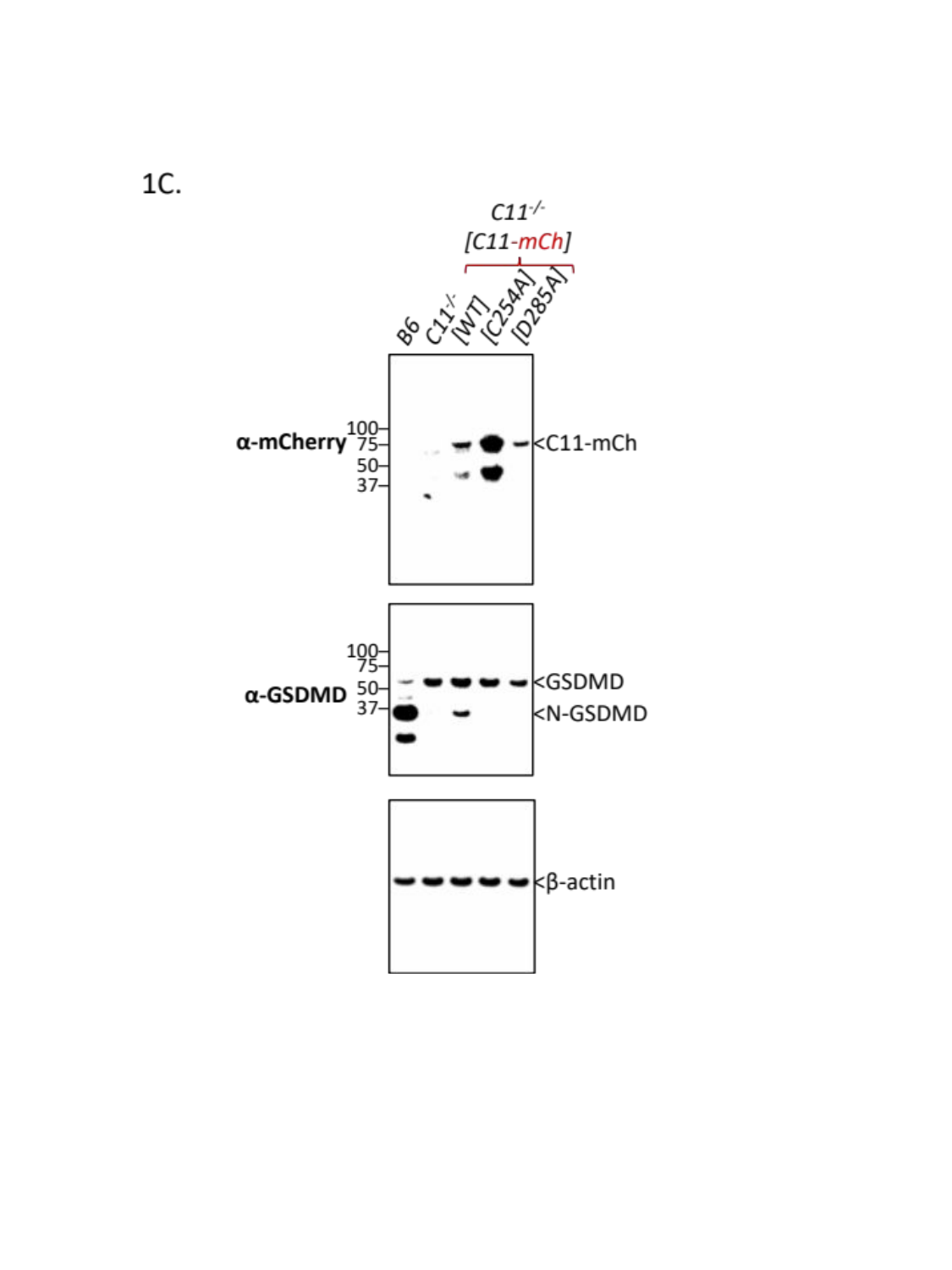

Supplement: Figure 1—source data 2. — Casp11-mCherry expression and gasdermin D (GSDMD) processing in response to lipopolysaccharide (LPS) transfection was assessed by western blotting for mCherry and GSDMD as indicated. β-actin was used as a loading control. [file elife-83725-fig1-data2.zip › Figure_1C_labeled.pptx]

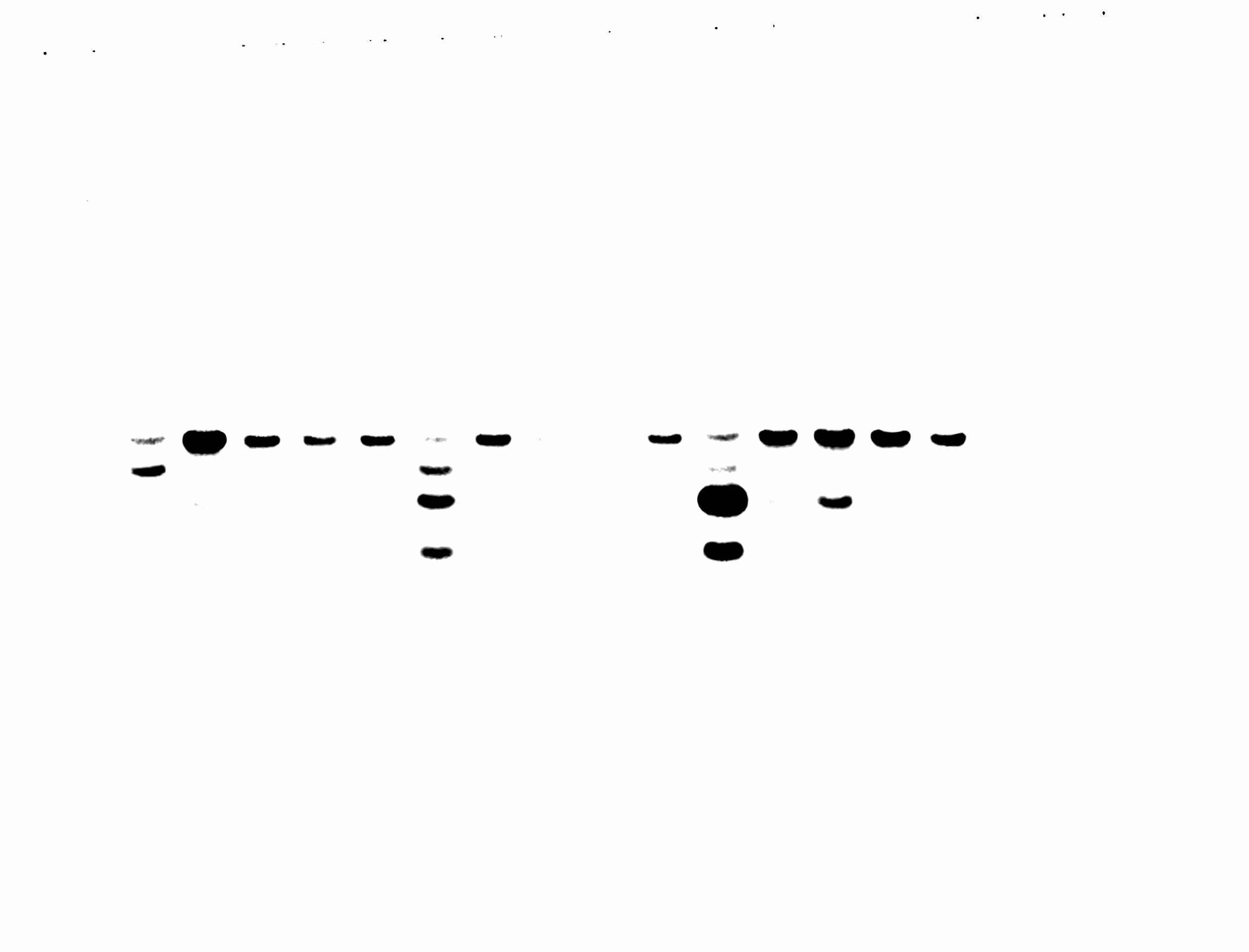

Supplement: Figure 1—source data 2. — Casp11-mCherry expression and gasdermin D (GSDMD) processing in response to lipopolysaccharide (LPS) transfection was assessed by western blotting for mCherry and GSDMD as indicated. β-actin was used as a loading control. [file elife-83725-fig1-data2.zip › GSDMD Hi-res.tif]

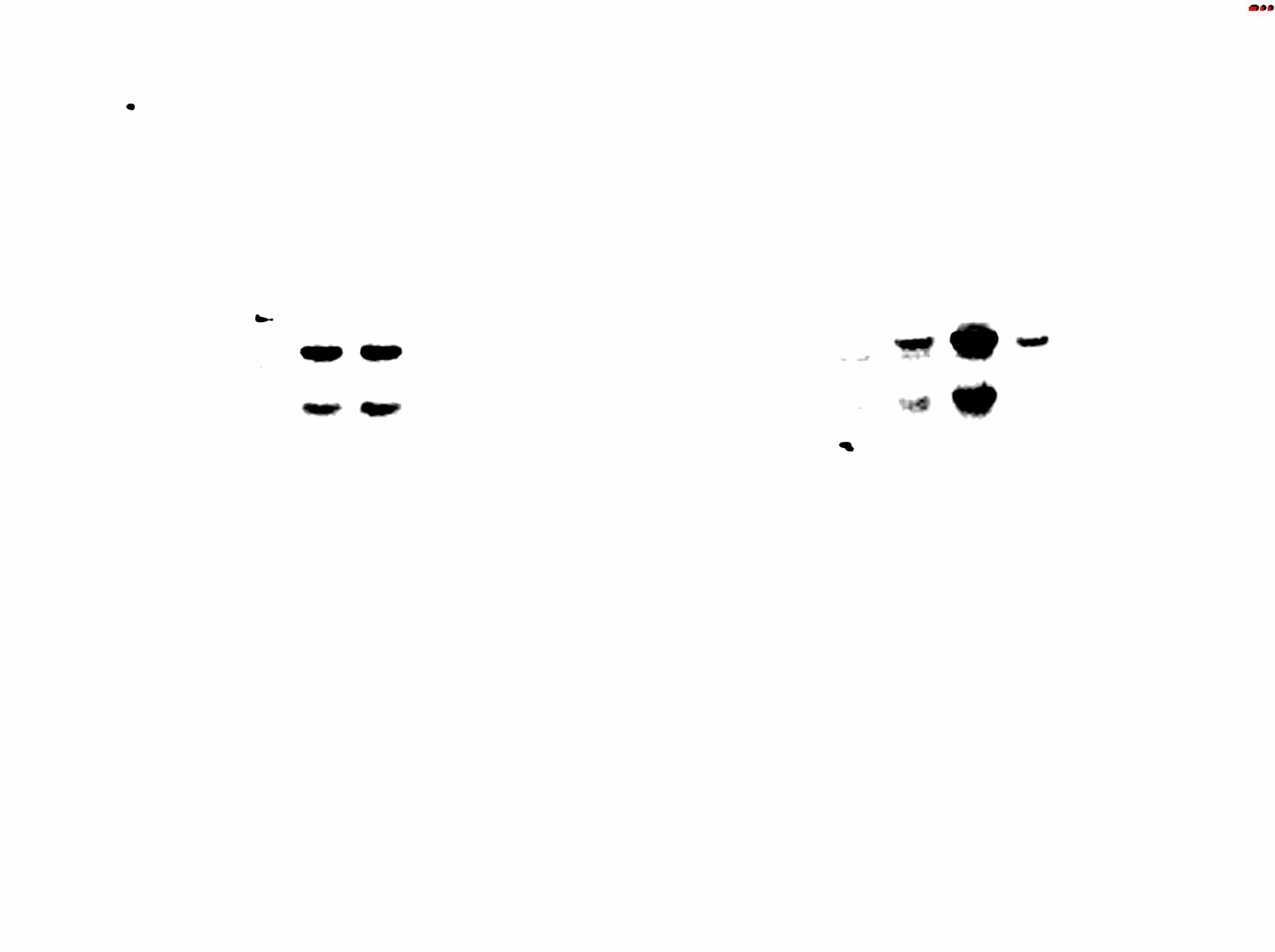

Supplement: Figure 1—source data 2. — Casp11-mCherry expression and gasdermin D (GSDMD) processing in response to lipopolysaccharide (LPS) transfection was assessed by western blotting for mCherry and GSDMD as indicated. β-actin was used as a loading control. [file elife-83725-fig1-data2.zip › mCherry Hi_res.tif]

Fig 1-source data 3 (1D)

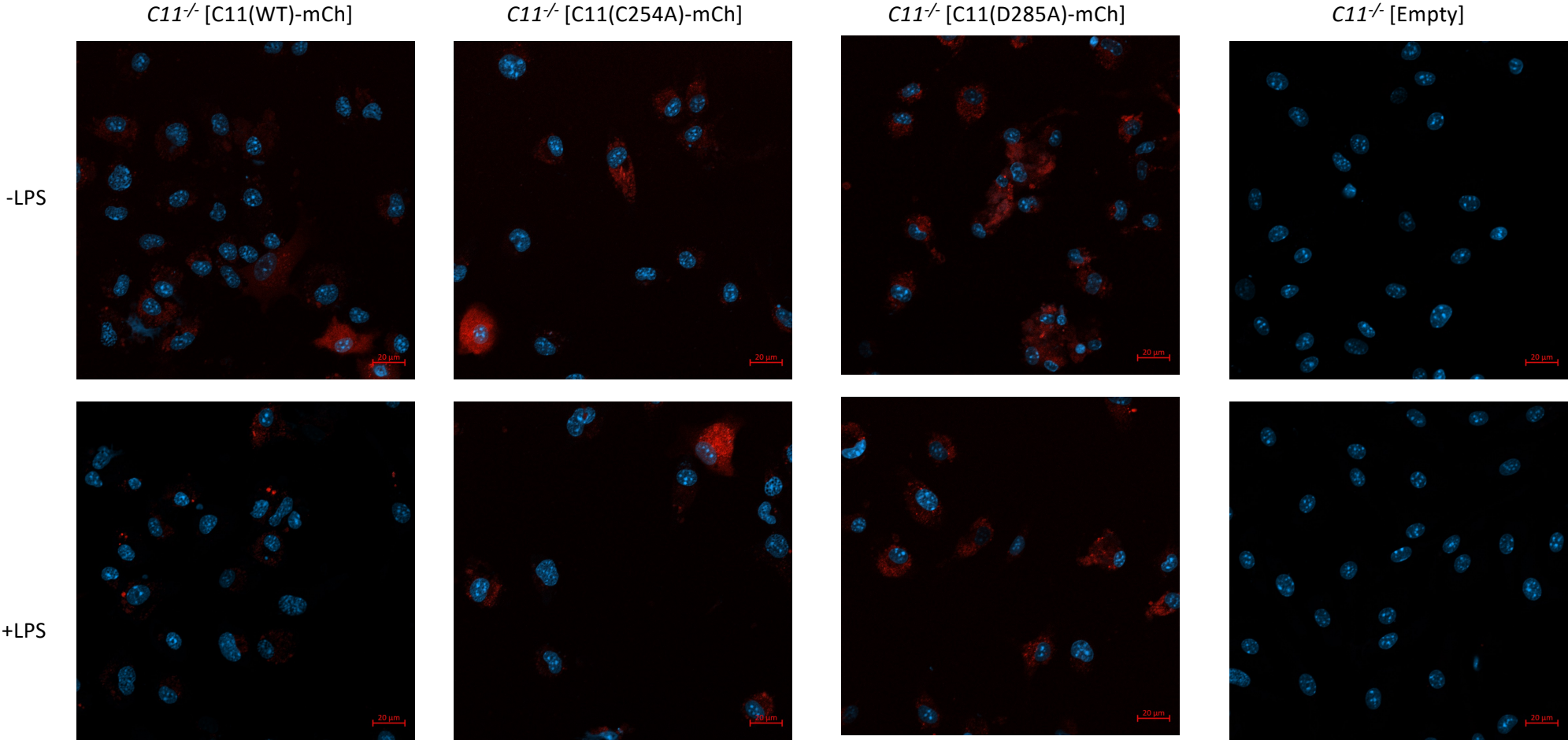

HOECHST mCHERRY

Supplement: Figure 1—source data 3. — Bone marrow-derived macrophages (BMDMs) expressing indicated Casp11-mCherry constructs were fixed post-lipopolysaccharide (LPS) transfection and prepared for confocal microscopy. Nuclei are stained with Hoechst. [file elife-83725-fig1-data3.zip › Figure 1-source data 3.pdf]

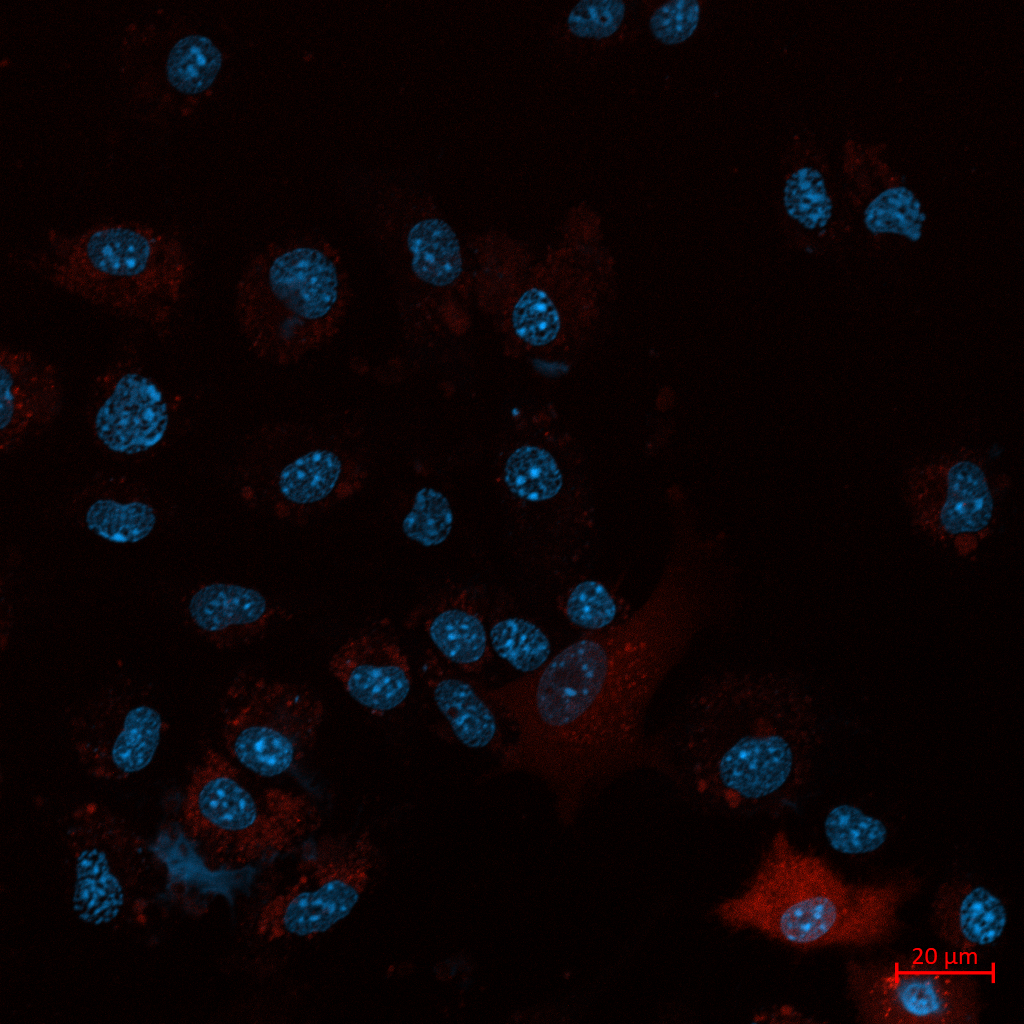

Supplement: Figure 1—source data 3. — Bone marrow-derived macrophages (BMDMs) expressing indicated Casp11-mCherry constructs were fixed post-lipopolysaccharide (LPS) transfection and prepared for confocal microscopy. Nuclei are stained with Hoechst. [file elife-83725-fig1-data3.zip › No_LPS_C11WT-mCh.tif]

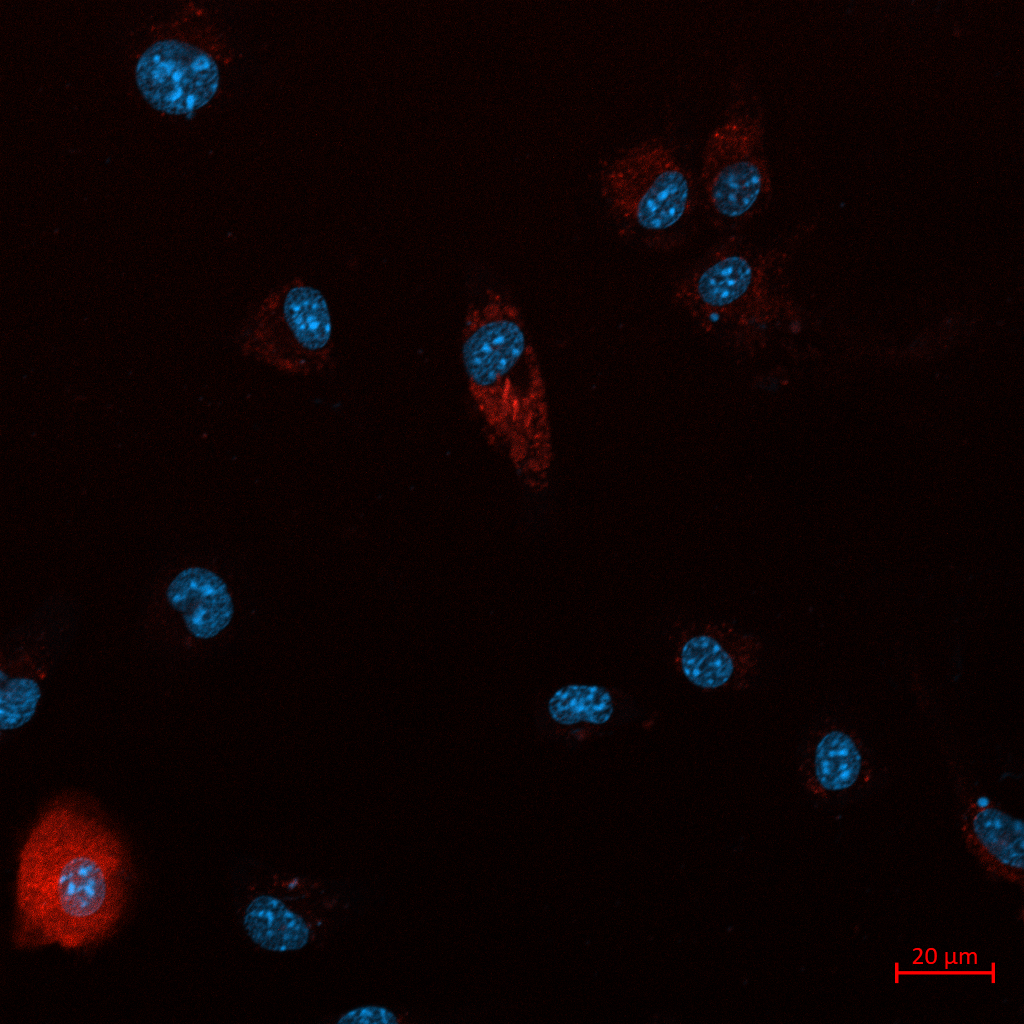

Supplement: Figure 1—source data 3. — Bone marrow-derived macrophages (BMDMs) expressing indicated Casp11-mCherry constructs were fixed post-lipopolysaccharide (LPS) transfection and prepared for confocal microscopy. Nuclei are stained with Hoechst. [file elife-83725-fig1-data3.zip › No_LPS_C254A-mCh.tif]

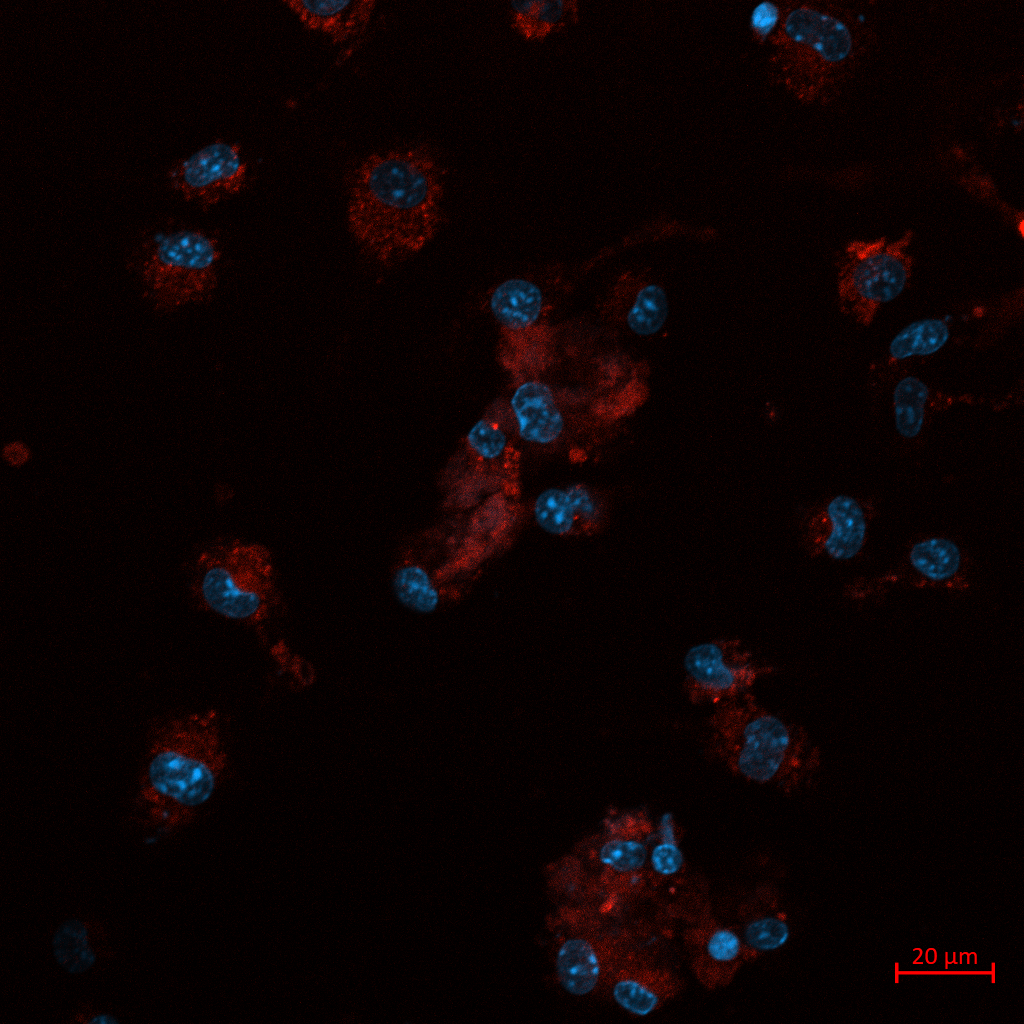

Supplement: Figure 1—source data 3. — Bone marrow-derived macrophages (BMDMs) expressing indicated Casp11-mCherry constructs were fixed post-lipopolysaccharide (LPS) transfection and prepared for confocal microscopy. Nuclei are stained with Hoechst. [file elife-83725-fig1-data3.zip › No_LPS_D285A-mCh.tif]

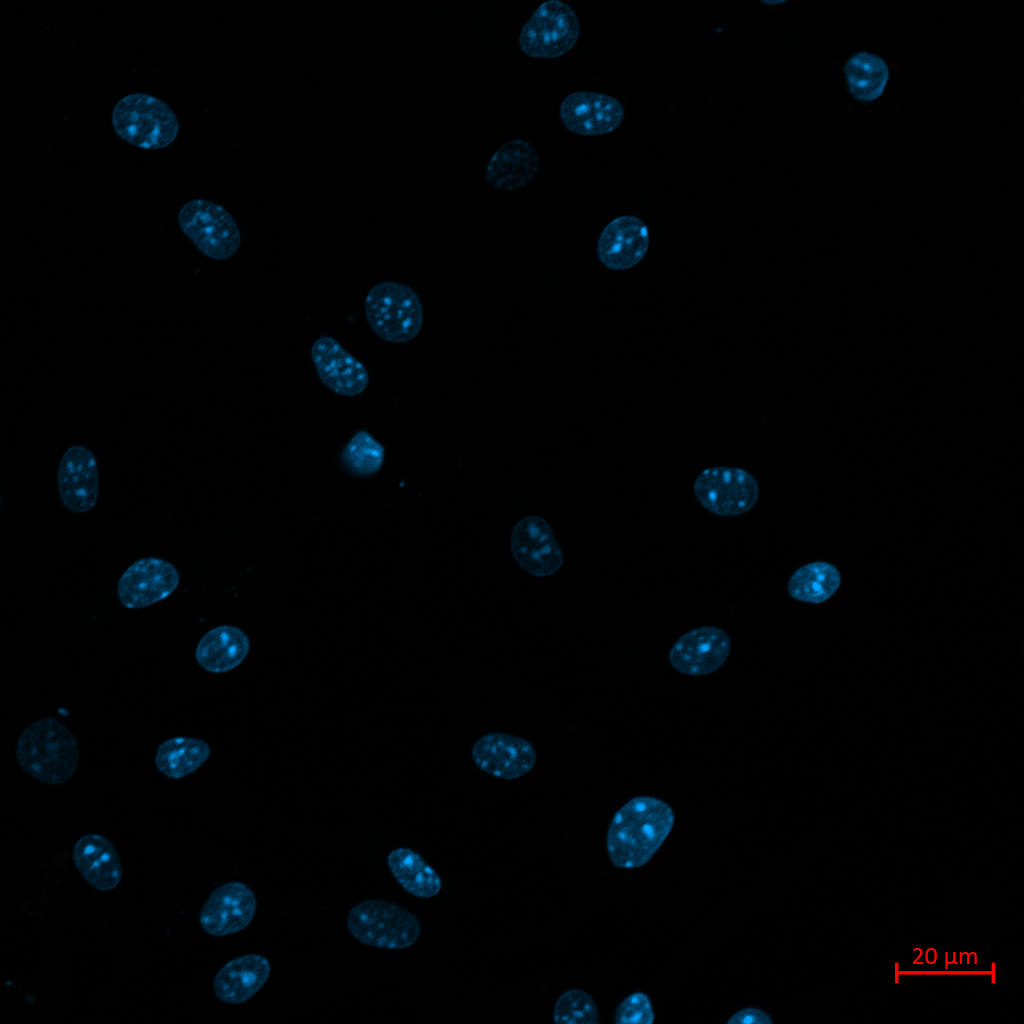

Supplement: Figure 1—source data 3. — Bone marrow-derived macrophages (BMDMs) expressing indicated Casp11-mCherry constructs were fixed post-lipopolysaccharide (LPS) transfection and prepared for confocal microscopy. Nuclei are stained with Hoechst. [file elife-83725-fig1-data3.zip › No_LPS_Empty_plasmid.tif]

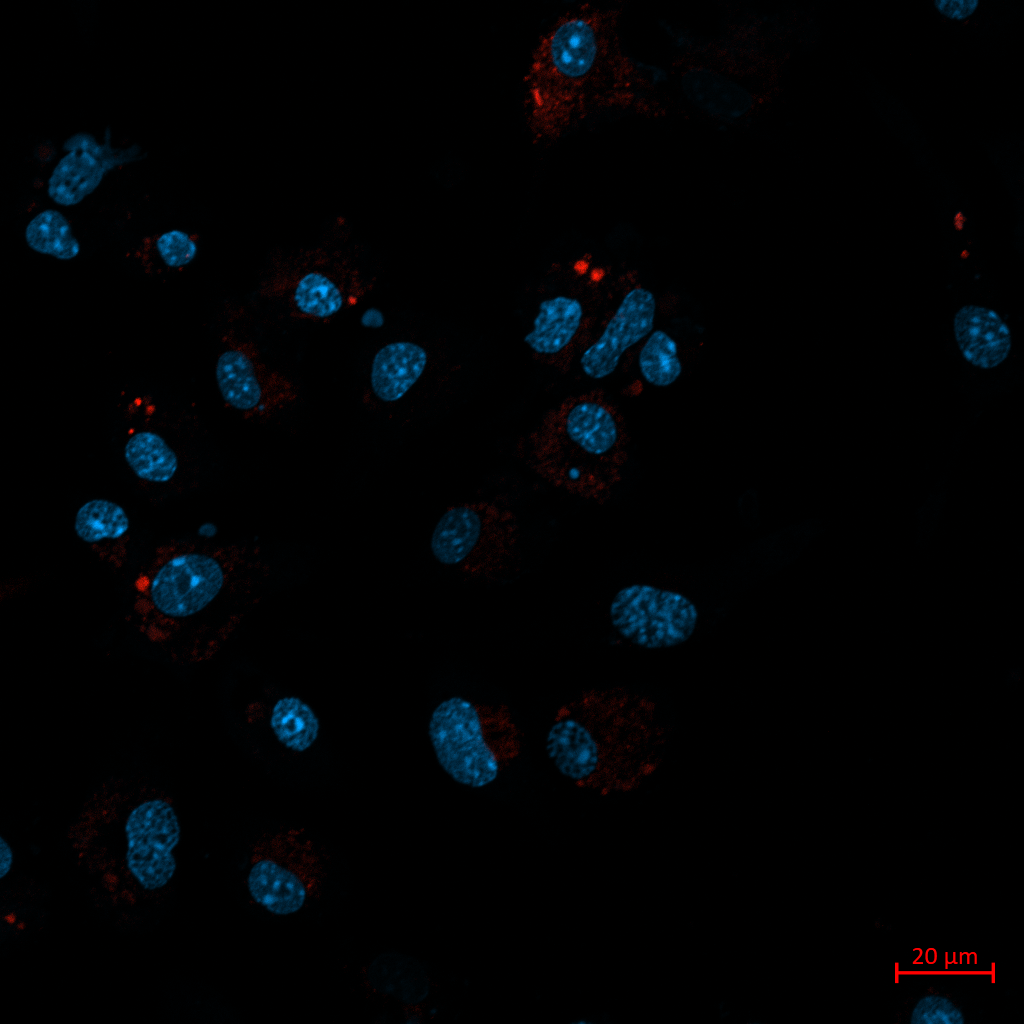

Supplement: Figure 1—source data 3. — Bone marrow-derived macrophages (BMDMs) expressing indicated Casp11-mCherry constructs were fixed post-lipopolysaccharide (LPS) transfection and prepared for confocal microscopy. Nuclei are stained with Hoechst. [file elife-83725-fig1-data3.zip › Yes_LPS_C11WT-mCh.tif]

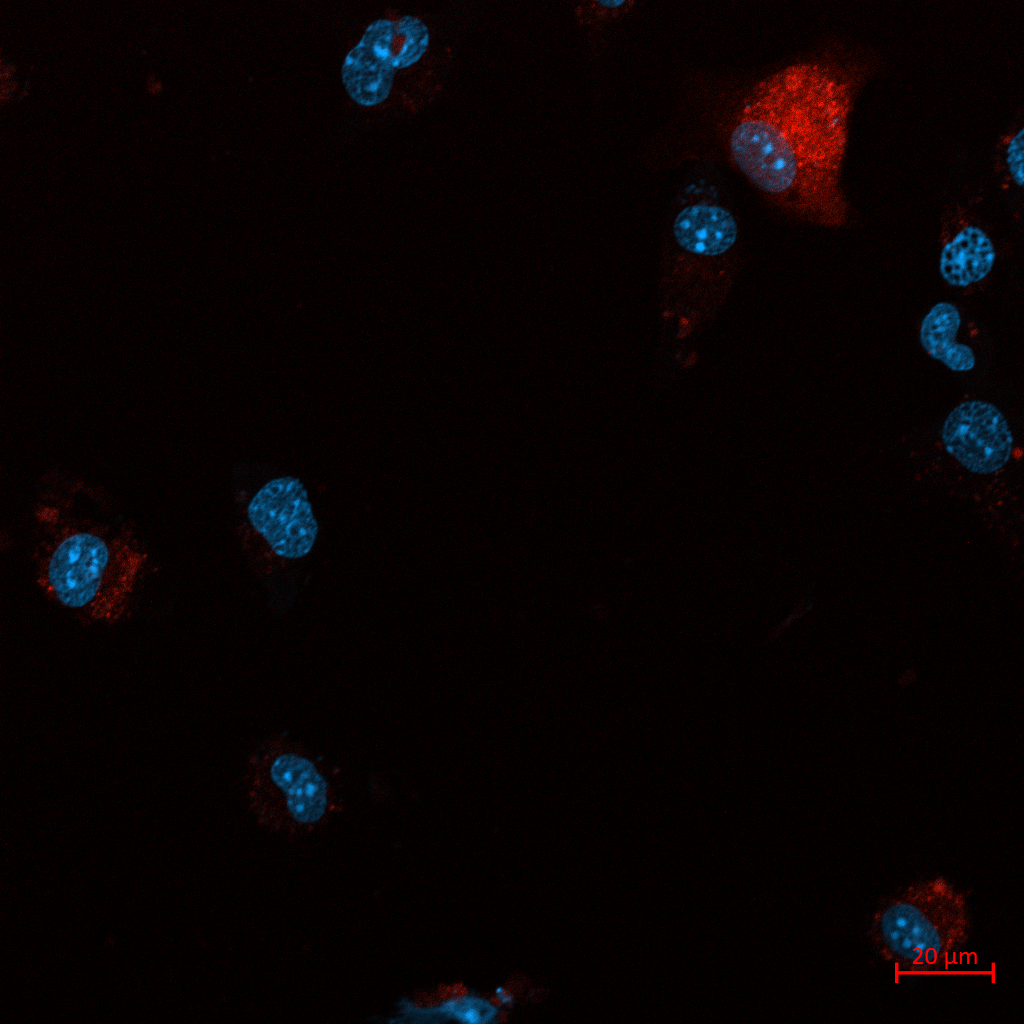

Supplement: Figure 1—source data 3. — Bone marrow-derived macrophages (BMDMs) expressing indicated Casp11-mCherry constructs were fixed post-lipopolysaccharide (LPS) transfection and prepared for confocal microscopy. Nuclei are stained with Hoechst. [file elife-83725-fig1-data3.zip › Yes_LPS_C254A-mCh.tif]

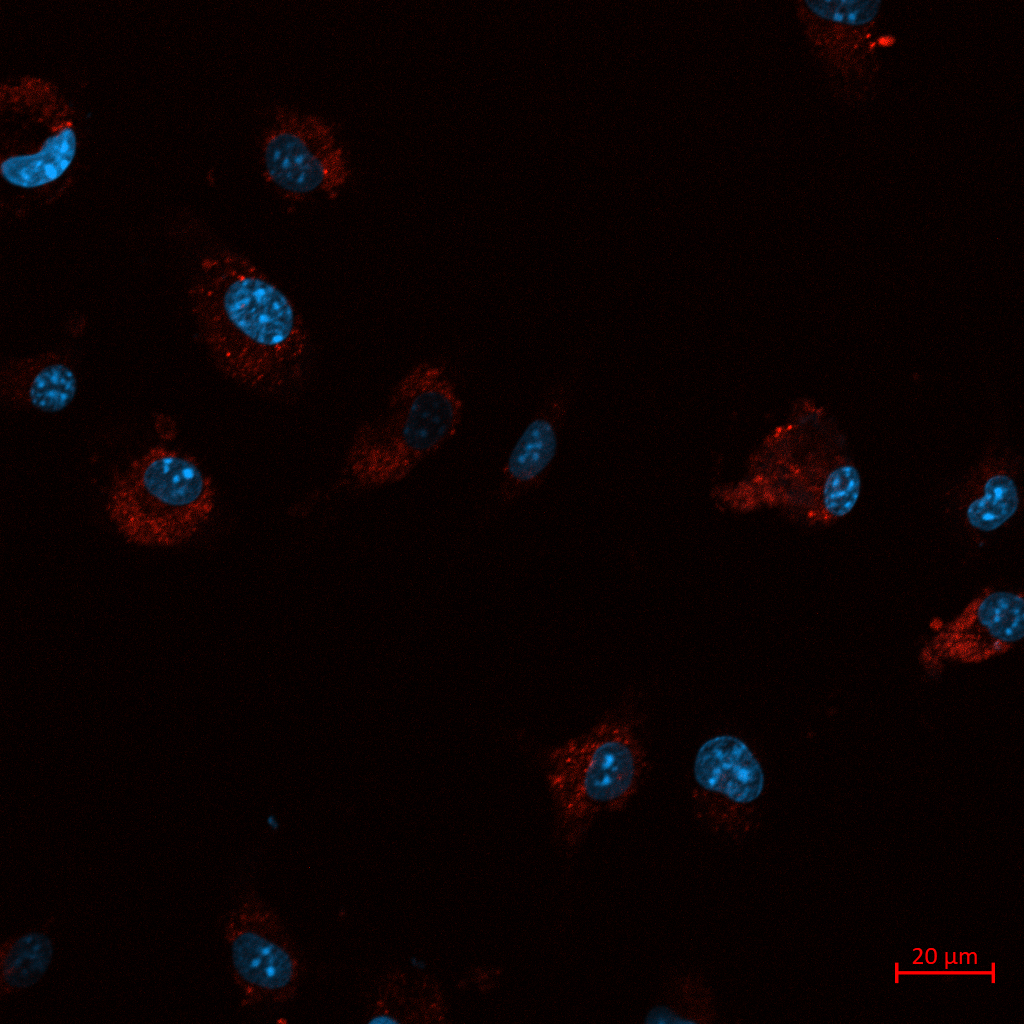

Supplement: Figure 1—source data 3. — Bone marrow-derived macrophages (BMDMs) expressing indicated Casp11-mCherry constructs were fixed post-lipopolysaccharide (LPS) transfection and prepared for confocal microscopy. Nuclei are stained with Hoechst. [file elife-83725-fig1-data3.zip › Yes_LPS_D285A-mCh.tif]

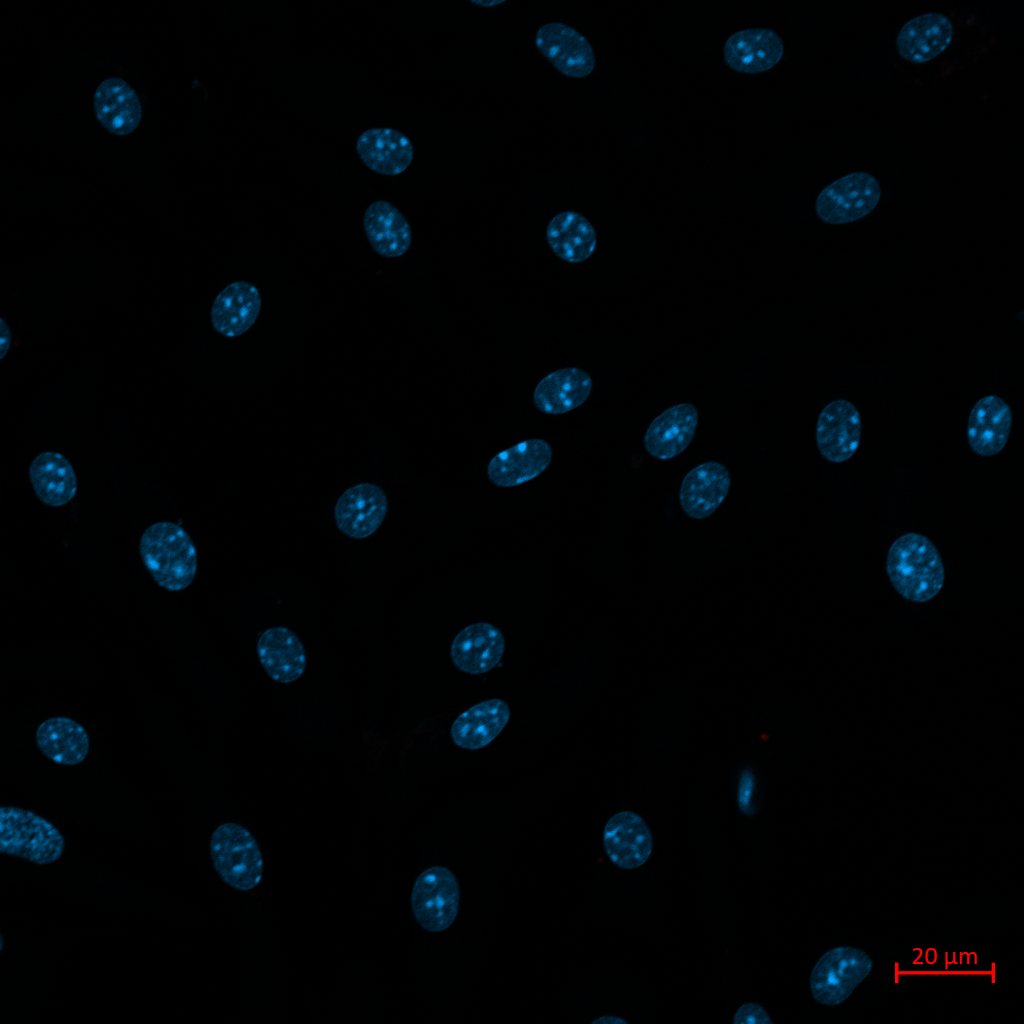

Supplement: Figure 1—source data 3. — Bone marrow-derived macrophages (BMDMs) expressing indicated Casp11-mCherry constructs were fixed post-lipopolysaccharide (LPS) transfection and prepared for confocal microscopy. Nuclei are stained with Hoechst. [file elife-83725-fig1-data3.zip › Yes_LPS_Empty_plasmid.tif]

1F.

[C11-*mCh*]:

[WT]

[C254A]

-*L. pneumo*

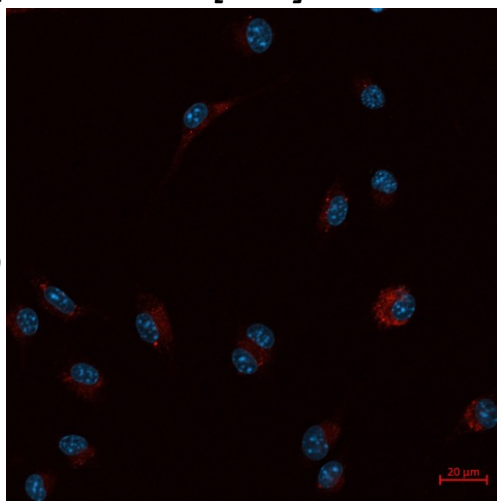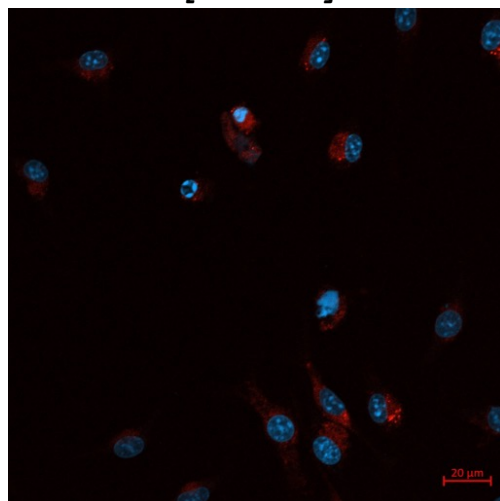

+*L. pneumo*

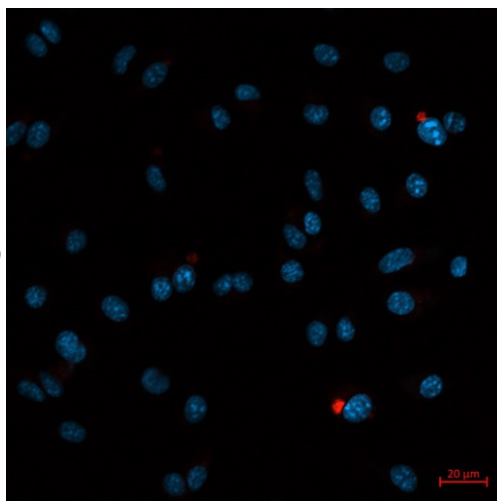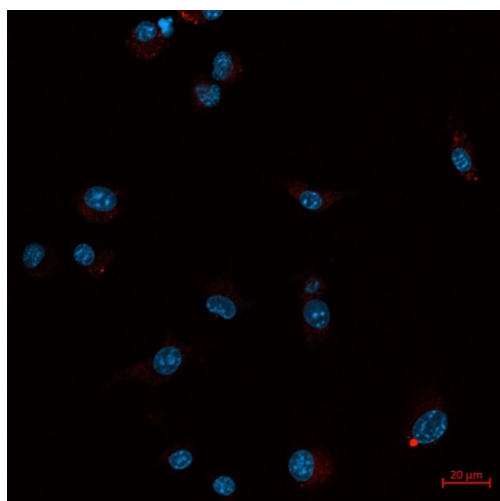

HOECHST *mCHERRY*

*C11*<sup>-/-</sup> BMDMs

Supplement: Figure 1—source data 4. — Bone marrow-derived macrophages (BMDMs) expressing indicated Casp11-mCherry constructs were fixed post-infection with Legionella pneumophila (MOI = 50) and prepared for confocal microscopy. Nuclei are stained with Hoechst. [file elife-83725-fig1-data4.zip › Figure 1-source data 4.pdf]

## Slide 1
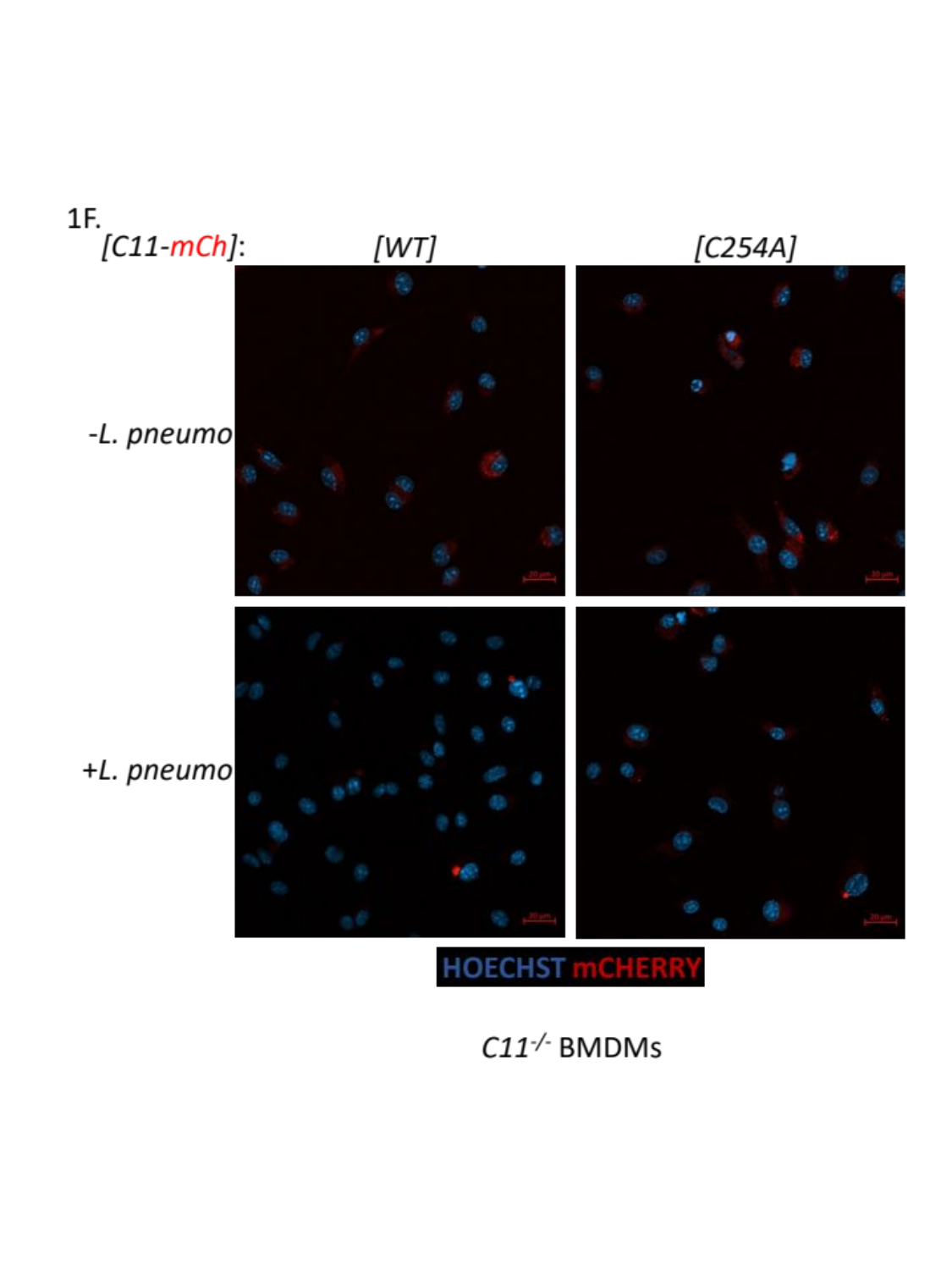

Supplement: Figure 1—source data 4. — Bone marrow-derived macrophages (BMDMs) expressing indicated Casp11-mCherry constructs were fixed post-infection with Legionella pneumophila (MOI = 50) and prepared for confocal microscopy. Nuclei are stained with Hoechst. [file elife-83725-fig1-data4.zip › Figure_1F_labeled.pptx]

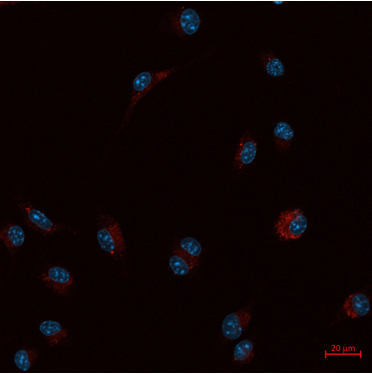

Supplement: Figure 1—source data 4. — Bone marrow-derived macrophages (BMDMs) expressing indicated Casp11-mCherry constructs were fixed post-infection with Legionella pneumophila (MOI = 50) and prepared for confocal microscopy. Nuclei are stained with Hoechst. [file elife-83725-fig1-data4.zip › No_L.pneumo_C11WT-mCh.png]

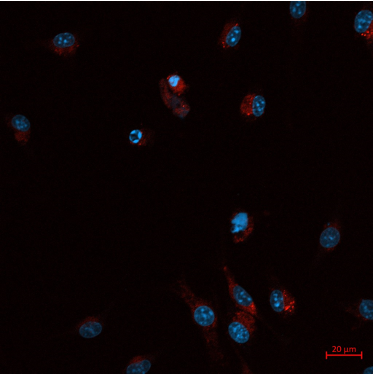

Supplement: Figure 1—source data 4. — Bone marrow-derived macrophages (BMDMs) expressing indicated Casp11-mCherry constructs were fixed post-infection with Legionella pneumophila (MOI = 50) and prepared for confocal microscopy. Nuclei are stained with Hoechst. [file elife-83725-fig1-data4.zip › No_L.pneumo_C254A-mCh.png]

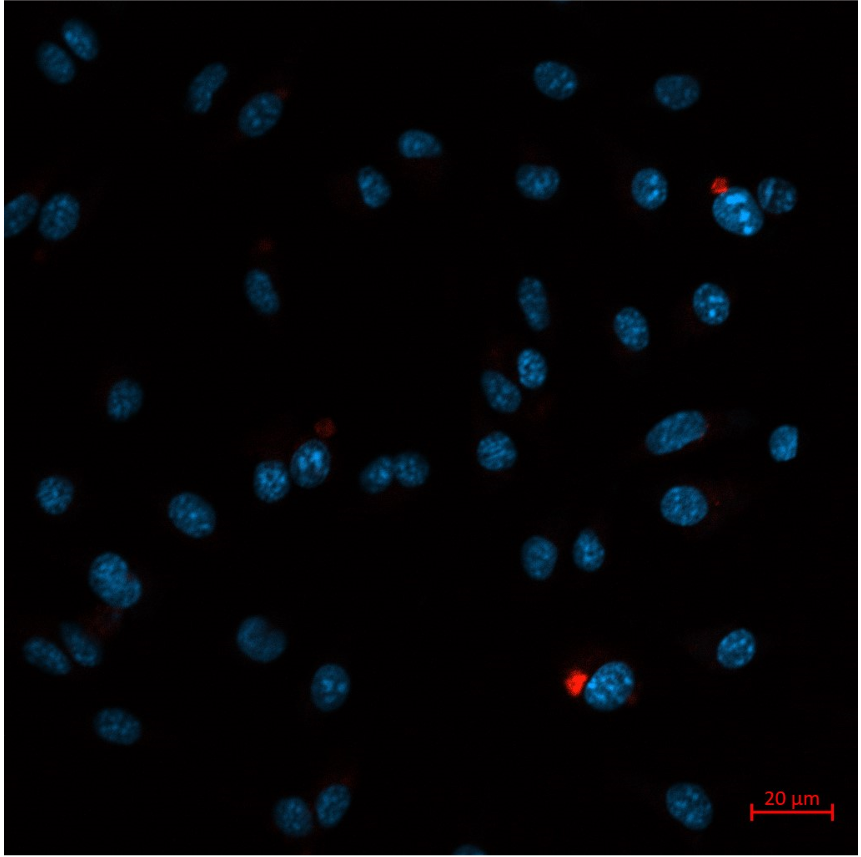

Supplement: Figure 1—source data 4. — Bone marrow-derived macrophages (BMDMs) expressing indicated Casp11-mCherry constructs were fixed post-infection with Legionella pneumophila (MOI = 50) and prepared for confocal microscopy. Nuclei are stained with Hoechst. [file elife-83725-fig1-data4.zip › Yes_L.pneumo_C11WT-mCh.png]

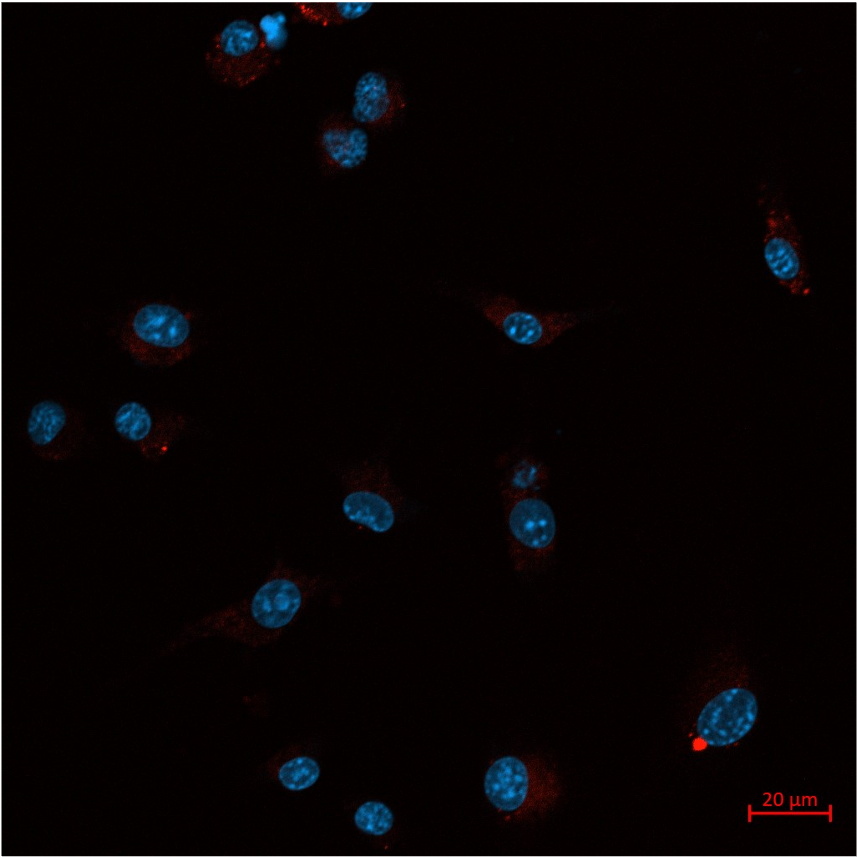

Supplement: Figure 1—source data 4. — Bone marrow-derived macrophages (BMDMs) expressing indicated Casp11-mCherry constructs were fixed post-infection with Legionella pneumophila (MOI = 50) and prepared for confocal microscopy. Nuclei are stained with Hoechst. [file elife-83725-fig1-data4.zip › Yes_L.pneumo_C254A-mCh.png]

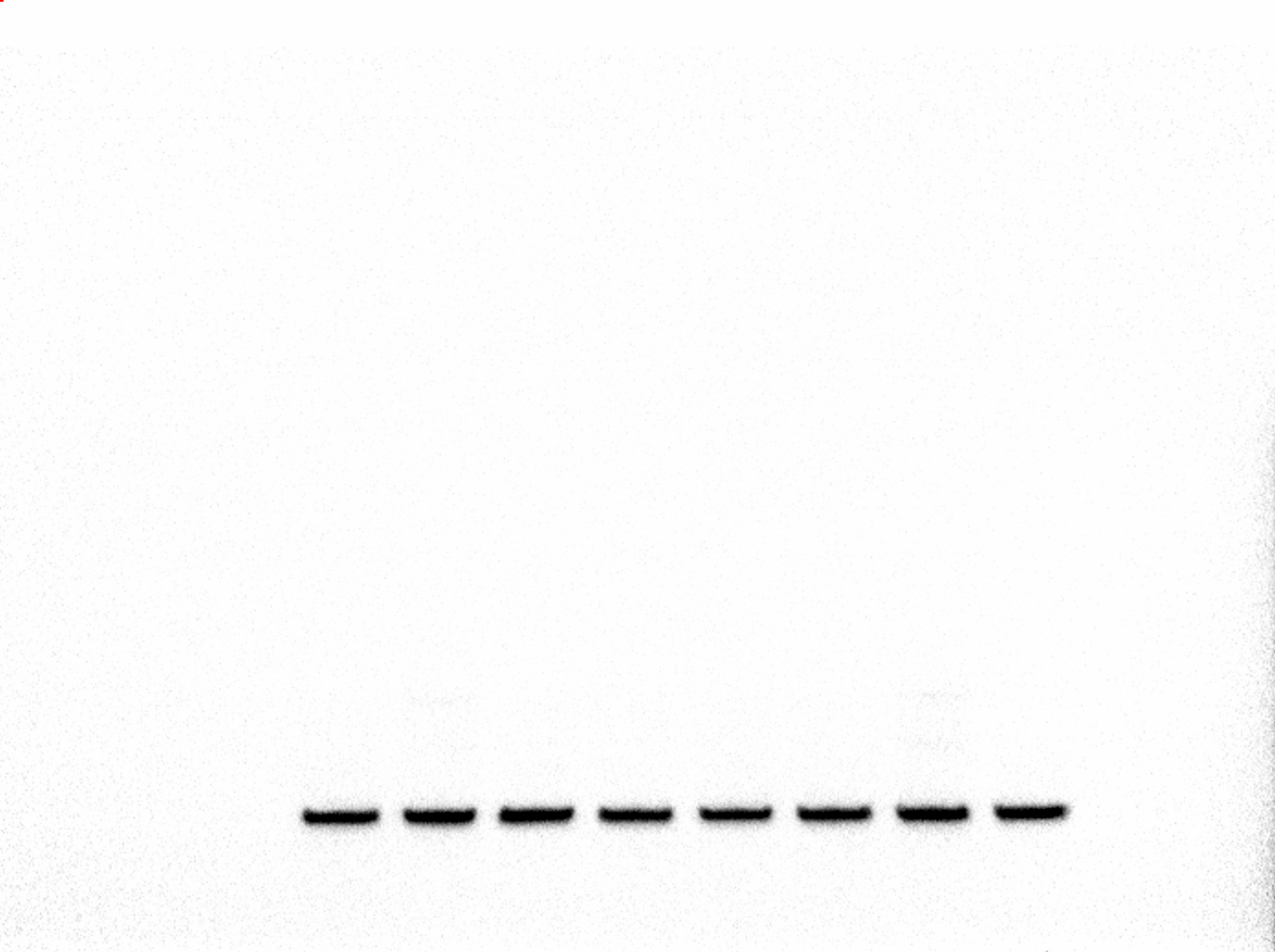

Supplement: Figure 2—source data 1. — Indicated Casp11-mCherry expression plasmids were transfected into HEK293T cells. Cell lysates were immunoblotted for mCherry, Casp11, and β-actin (loading control) 10 hr post-transfection. [file elife-83725-fig2-data1.zip › Actin.tif]

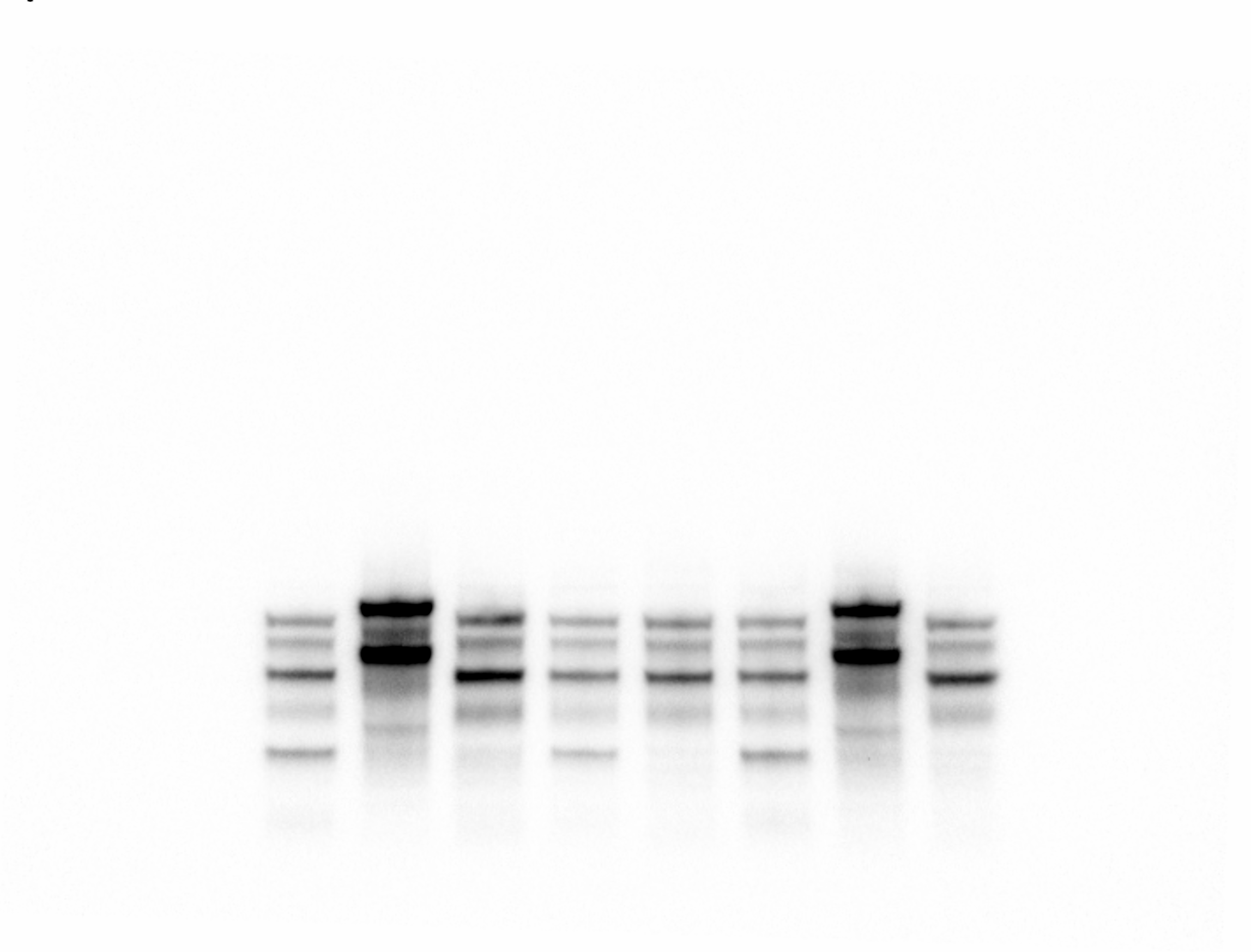

Supplement: Figure 2—source data 1. — Indicated Casp11-mCherry expression plasmids were transfected into HEK293T cells. Cell lysates were immunoblotted for mCherry, Casp11, and β-actin (loading control) 10 hr post-transfection. [file elife-83725-fig2-data1.zip › Casp11.tif]

2A.

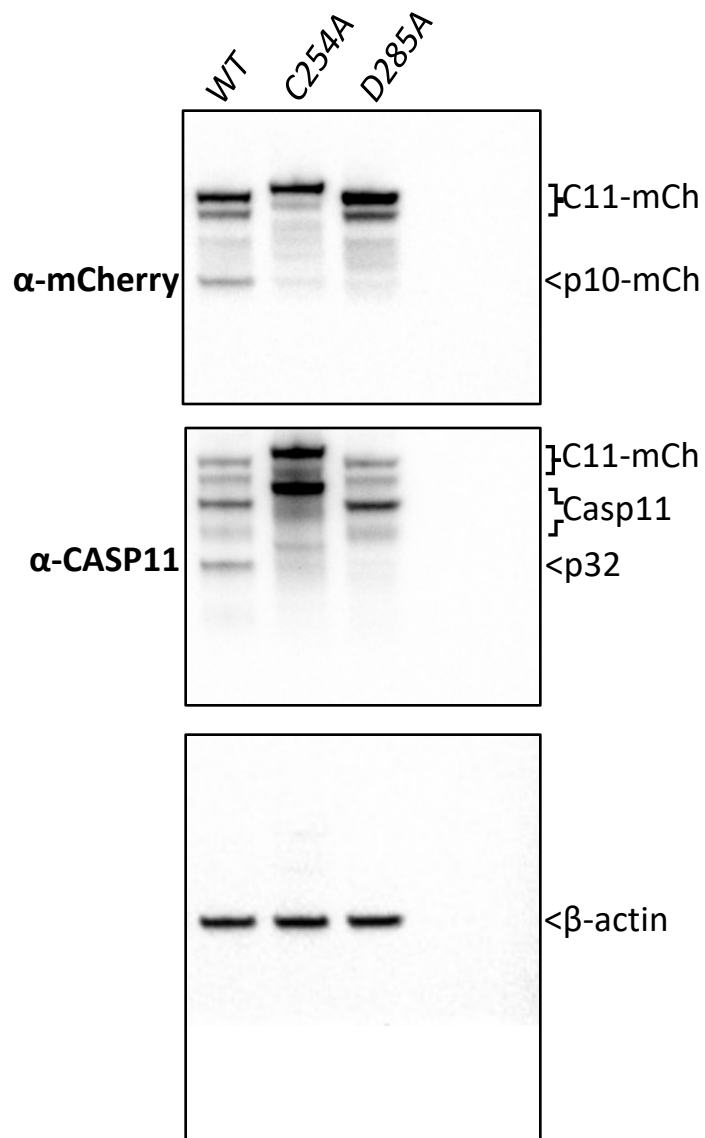

Supplement: Figure 2—source data 1. — Indicated Casp11-mCherry expression plasmids were transfected into HEK293T cells. Cell lysates were immunoblotted for mCherry, Casp11, and β-actin (loading control) 10 hr post-transfection. [file elife-83725-fig2-data1.zip › Figure 2-source data 1.pdf]

## Slide 1
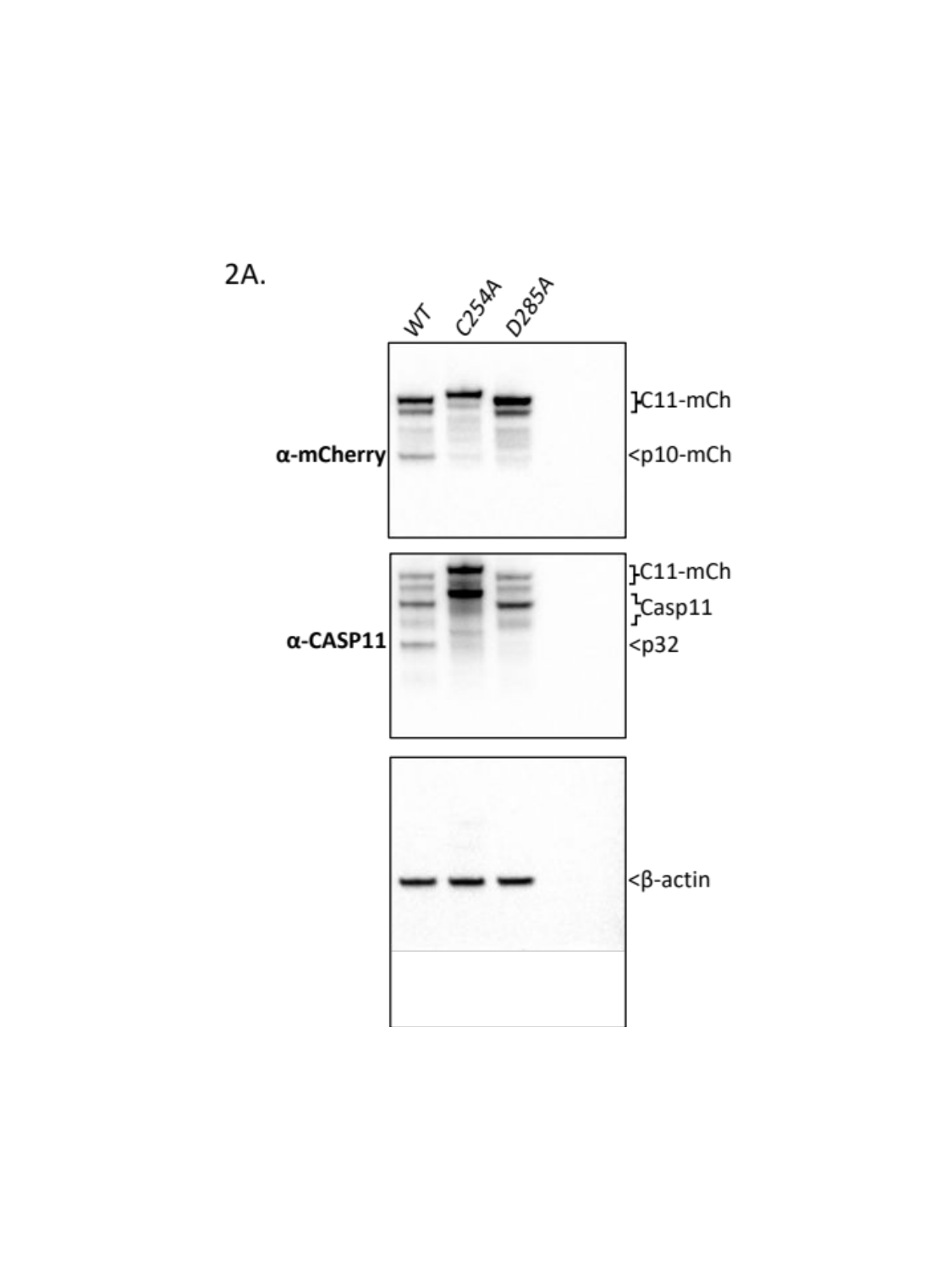

Supplement: Figure 2—source data 1. — Indicated Casp11-mCherry expression plasmids were transfected into HEK293T cells. Cell lysates were immunoblotted for mCherry, Casp11, and β-actin (loading control) 10 hr post-transfection. [file elife-83725-fig2-data1.zip › Figure_2A_labeled.pptx]

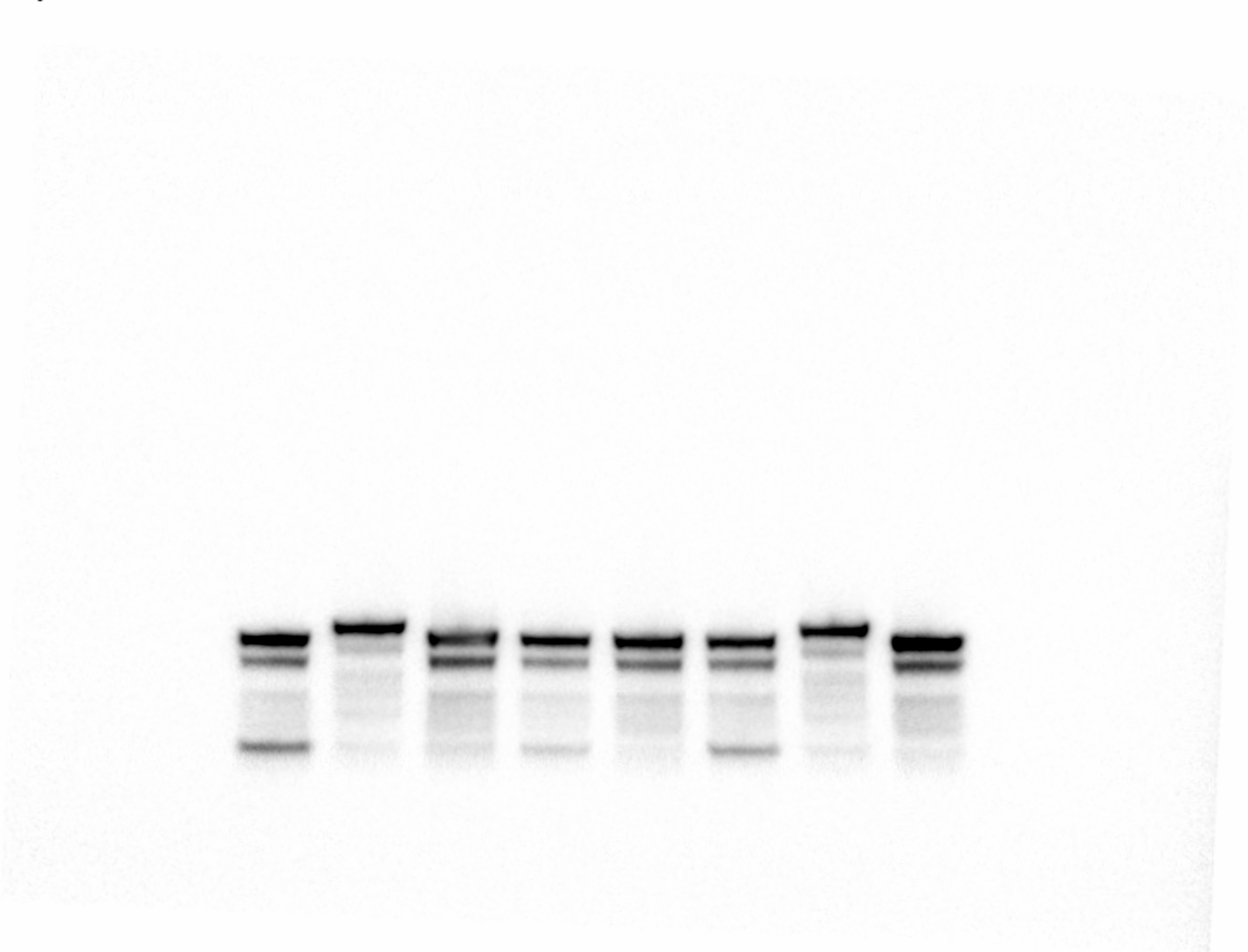

Supplement: Figure 2—source data 1. — Indicated Casp11-mCherry expression plasmids were transfected into HEK293T cells. Cell lysates were immunoblotted for mCherry, Casp11, and β-actin (loading control) 10 hr post-transfection. [file elife-83725-fig2-data1.zip › mCherry.tif]

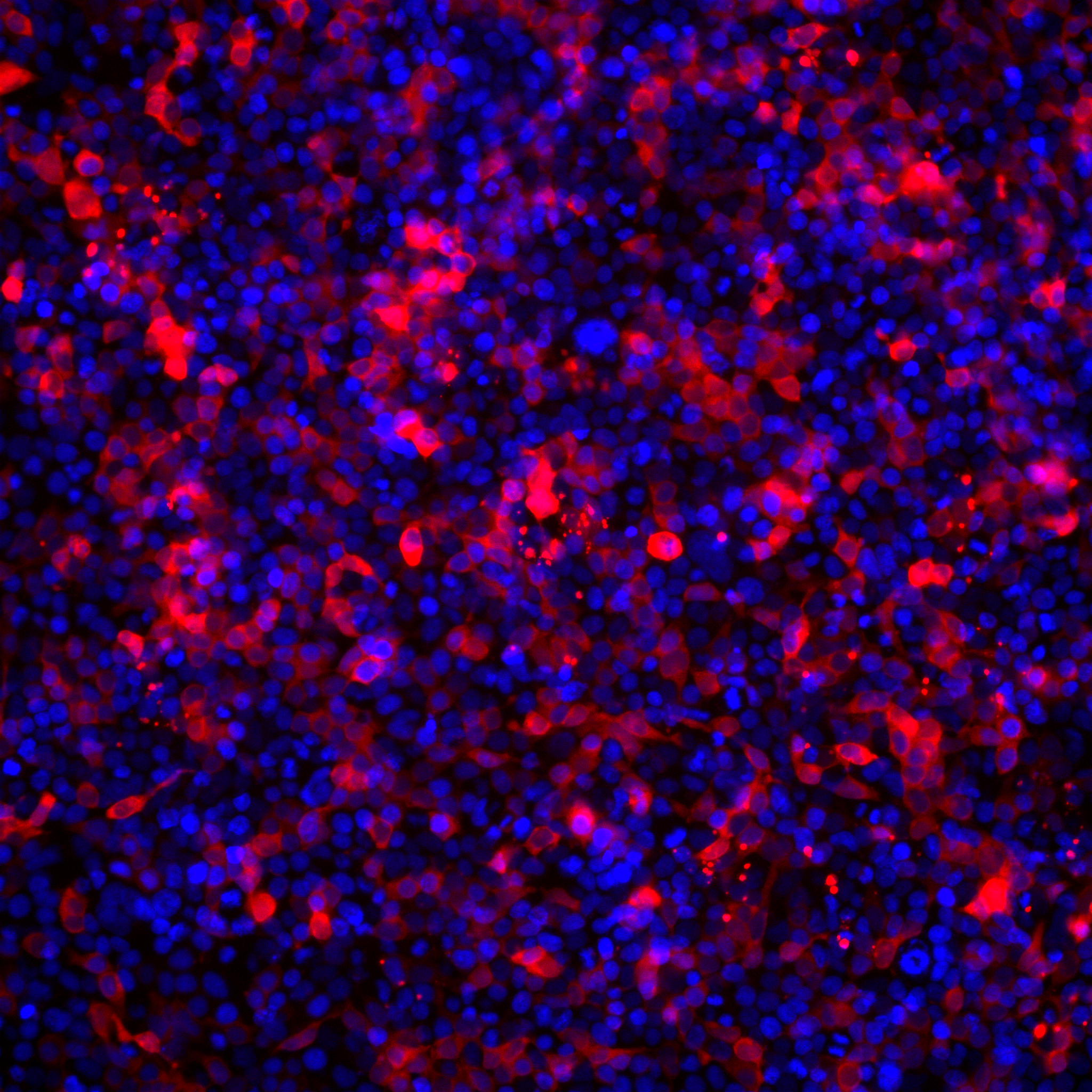

Supplement: Figure 2—source data 2. — HEK293T cells were transfected with wild-type (WT), catalytically inactive (C254A), or non-cleavable (D285A) Casp11-mCherry and imaged by fluorescence microscopy 18 hr post-transfection. Nuclei (blue) were stained with Hoechst. [file elife-83725-fig2-data2.zip › C11(WT)-mCh.tif]

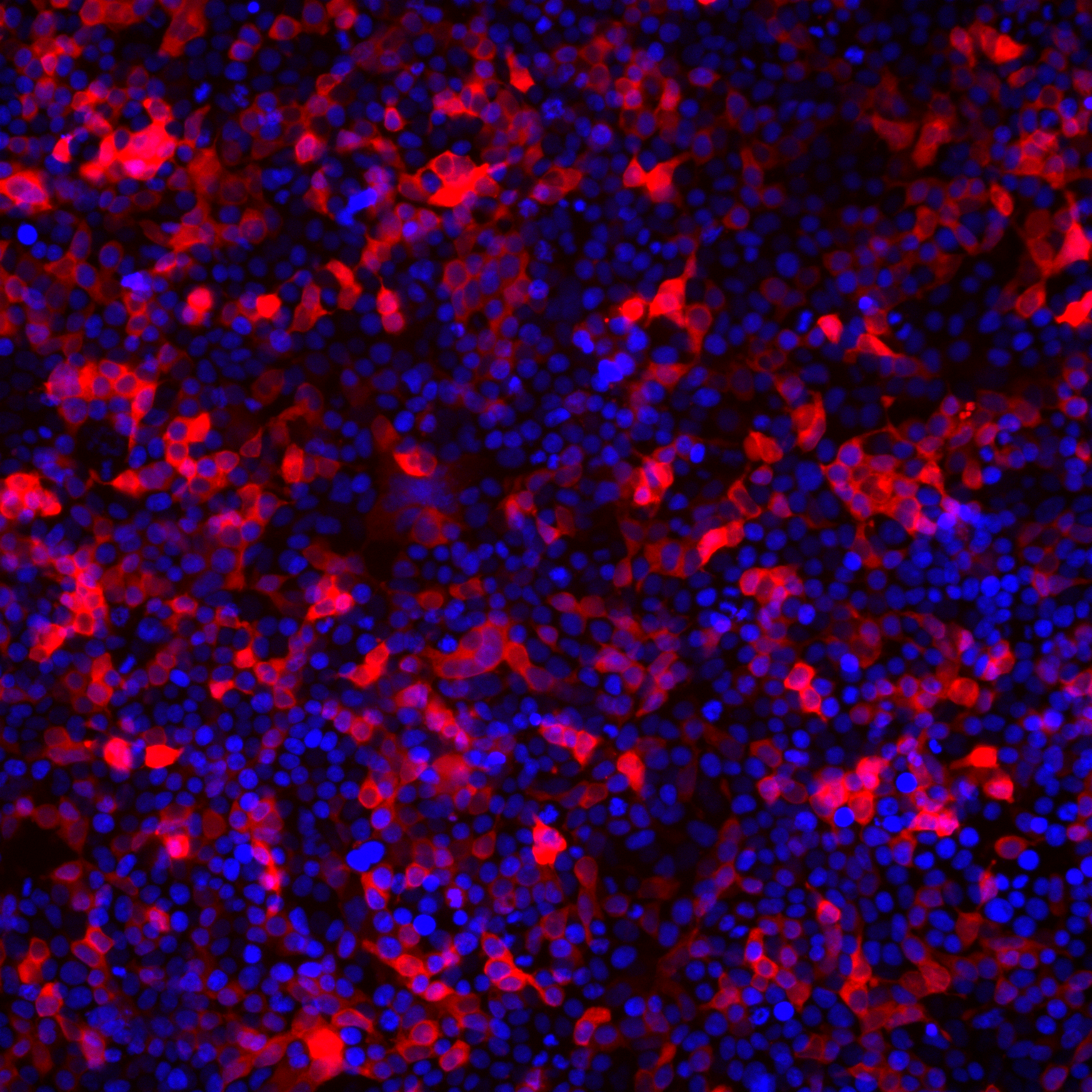

Supplement: Figure 2—source data 2. — HEK293T cells were transfected with wild-type (WT), catalytically inactive (C254A), or non-cleavable (D285A) Casp11-mCherry and imaged by fluorescence microscopy 18 hr post-transfection. Nuclei (blue) were stained with Hoechst. [file elife-83725-fig2-data2.zip › C254A-mCh.tif]

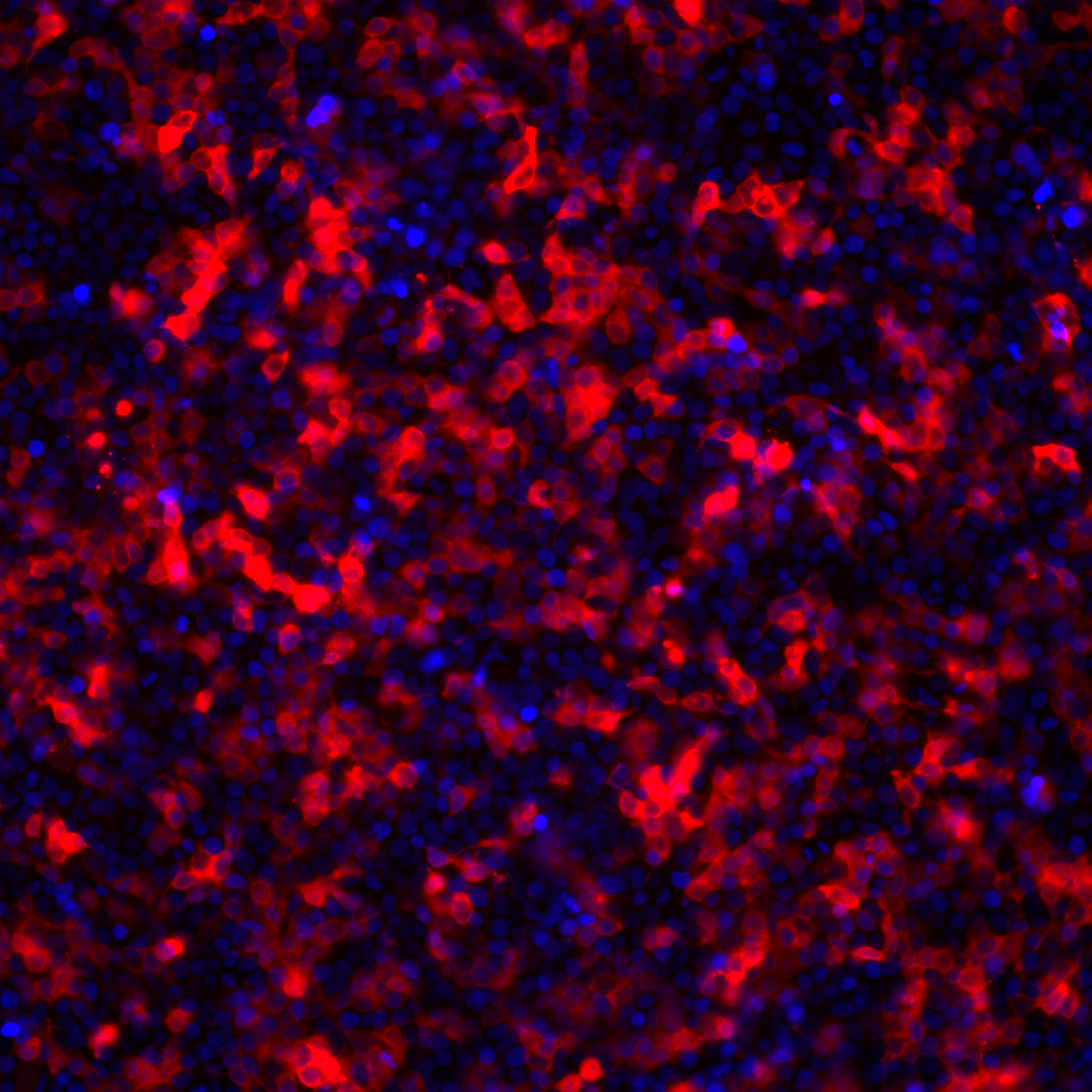

Supplement: Figure 2—source data 2. — HEK293T cells were transfected with wild-type (WT), catalytically inactive (C254A), or non-cleavable (D285A) Casp11-mCherry and imaged by fluorescence microscopy 18 hr post-transfection. Nuclei (blue) were stained with Hoechst. [file elife-83725-fig2-data2.zip › D285A-mCh.tif]

Fig 2-source data 2 (2B)

HEK293T cells

Casp11(WT)-mCh

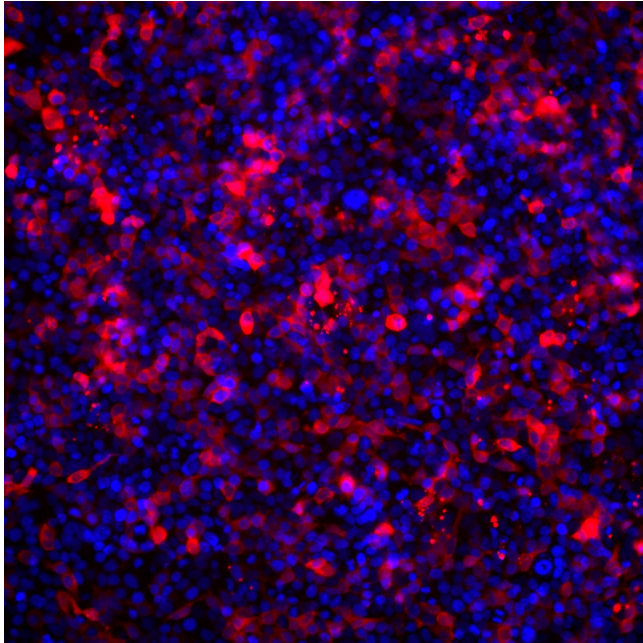

Casp11(C254A)-mCh

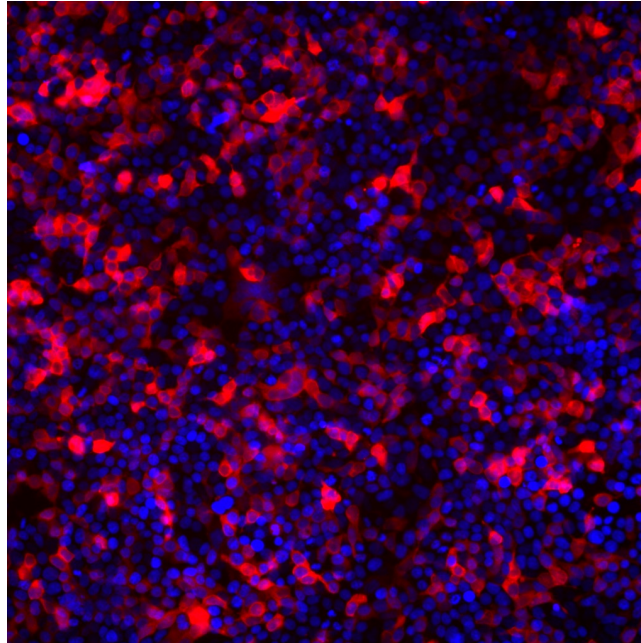

Casp11(C254A)-mCh

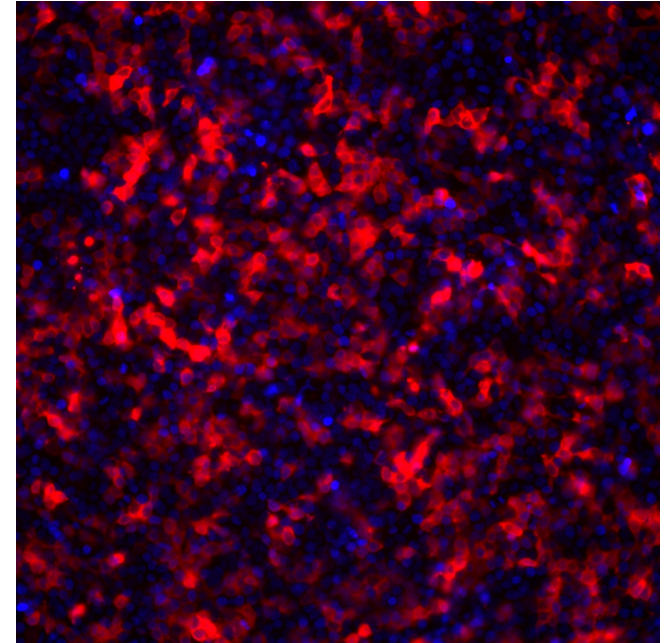

HOECHST mCHERRY

Supplement: Figure 2—source data 2. — HEK293T cells were transfected with wild-type (WT), catalytically inactive (C254A), or non-cleavable (D285A) Casp11-mCherry and imaged by fluorescence microscopy 18 hr post-transfection. Nuclei (blue) were stained with Hoechst. [file elife-83725-fig2-data2.zip › Figure 2-source data 2.pdf]

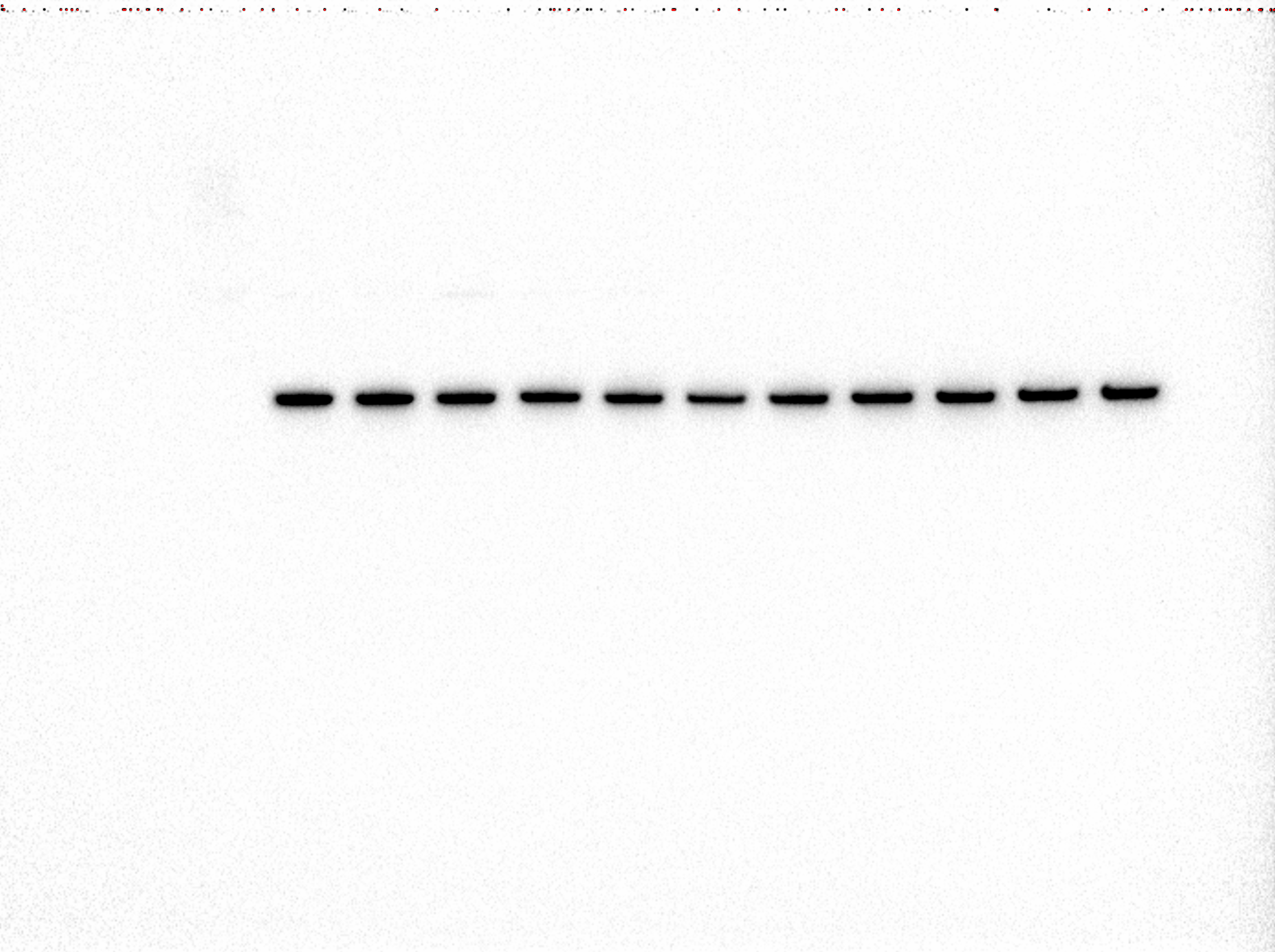

Supplement: Figure 2—source data 3. — HEK293T cells were transfected with Casp11-mCherry constructs and 6 hr post-transfection, cells were incubated with increasing amounts of pan-caspase inhibitor zVAD (0–200 μM; twofold increments). Whole-cell lysates were isolated 12 hr post-transfection and immunoblotted for mCherry or β-actin as loading control as indicated. Cleaved p10-mCherry is denoted. [file elife-83725-fig2-data3.zip › Actin.tif]

2D.

*C11-mCh:*  
zVAD:

*WT*

*C254A*

$\alpha$ -mCh

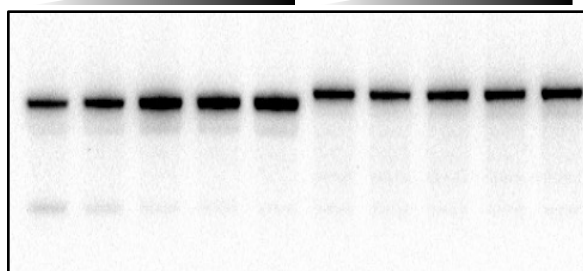

$\alpha$ -C11-mCh

<p10-mCh

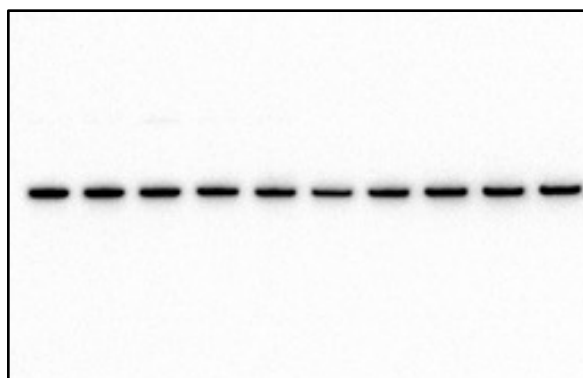

< $\beta$ -actin

Supplement: Figure 2—source data 3. — HEK293T cells were transfected with Casp11-mCherry constructs and 6 hr post-transfection, cells were incubated with increasing amounts of pan-caspase inhibitor zVAD (0–200 μM; twofold increments). Whole-cell lysates were isolated 12 hr post-transfection and immunoblotted for mCherry or β-actin as loading control as indicated. Cleaved p10-mCherry is denoted. [file elife-83725-fig2-data3.zip › Figure 2-source data 3.pdf]

## Slide 1
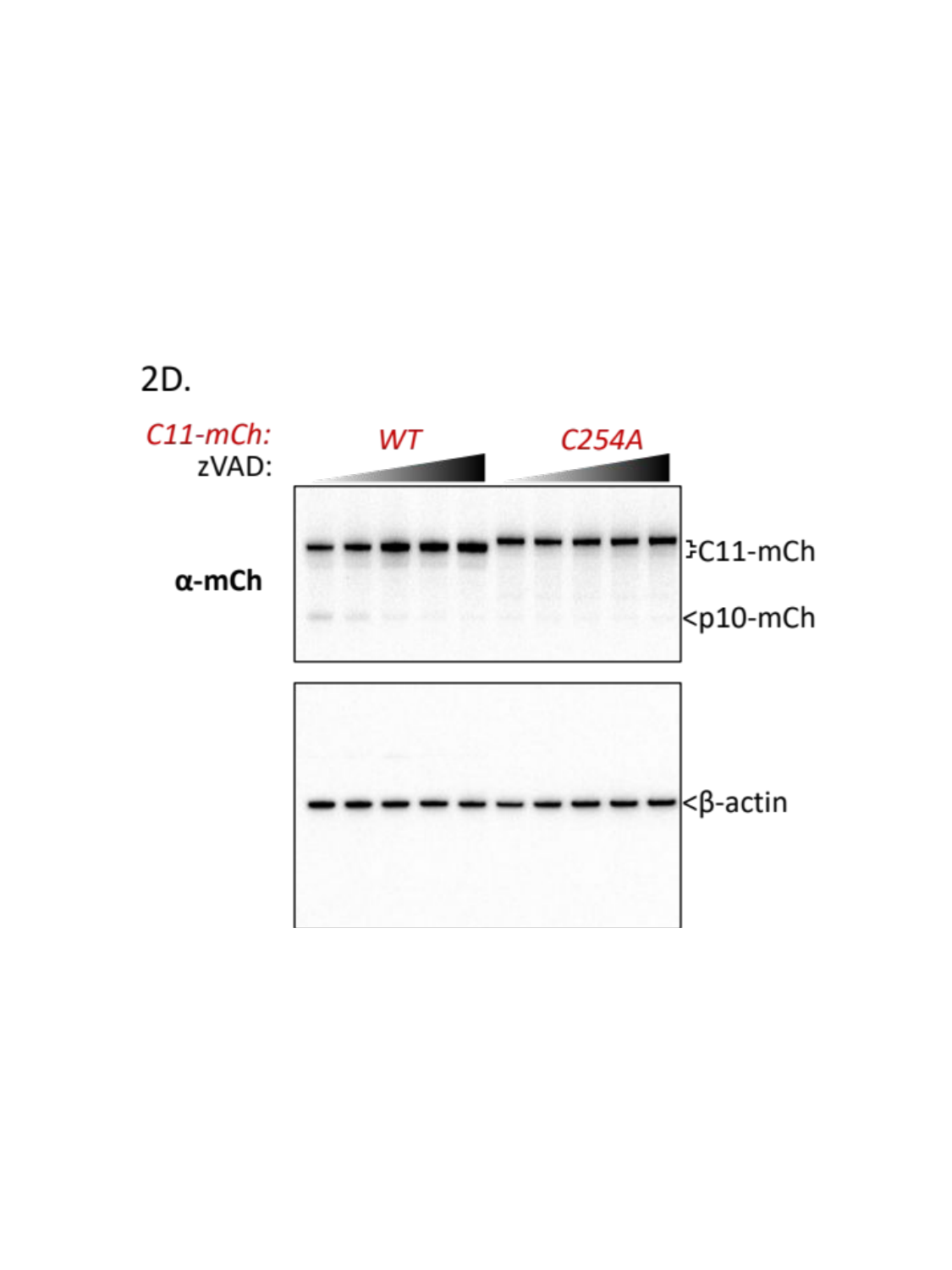

Supplement: Figure 2—source data 3. — HEK293T cells were transfected with Casp11-mCherry constructs and 6 hr post-transfection, cells were incubated with increasing amounts of pan-caspase inhibitor zVAD (0–200 μM; twofold increments). Whole-cell lysates were isolated 12 hr post-transfection and immunoblotted for mCherry or β-actin as loading control as indicated. Cleaved p10-mCherry is denoted. [file elife-83725-fig2-data3.zip › Figure_2D_labeled.pptx]

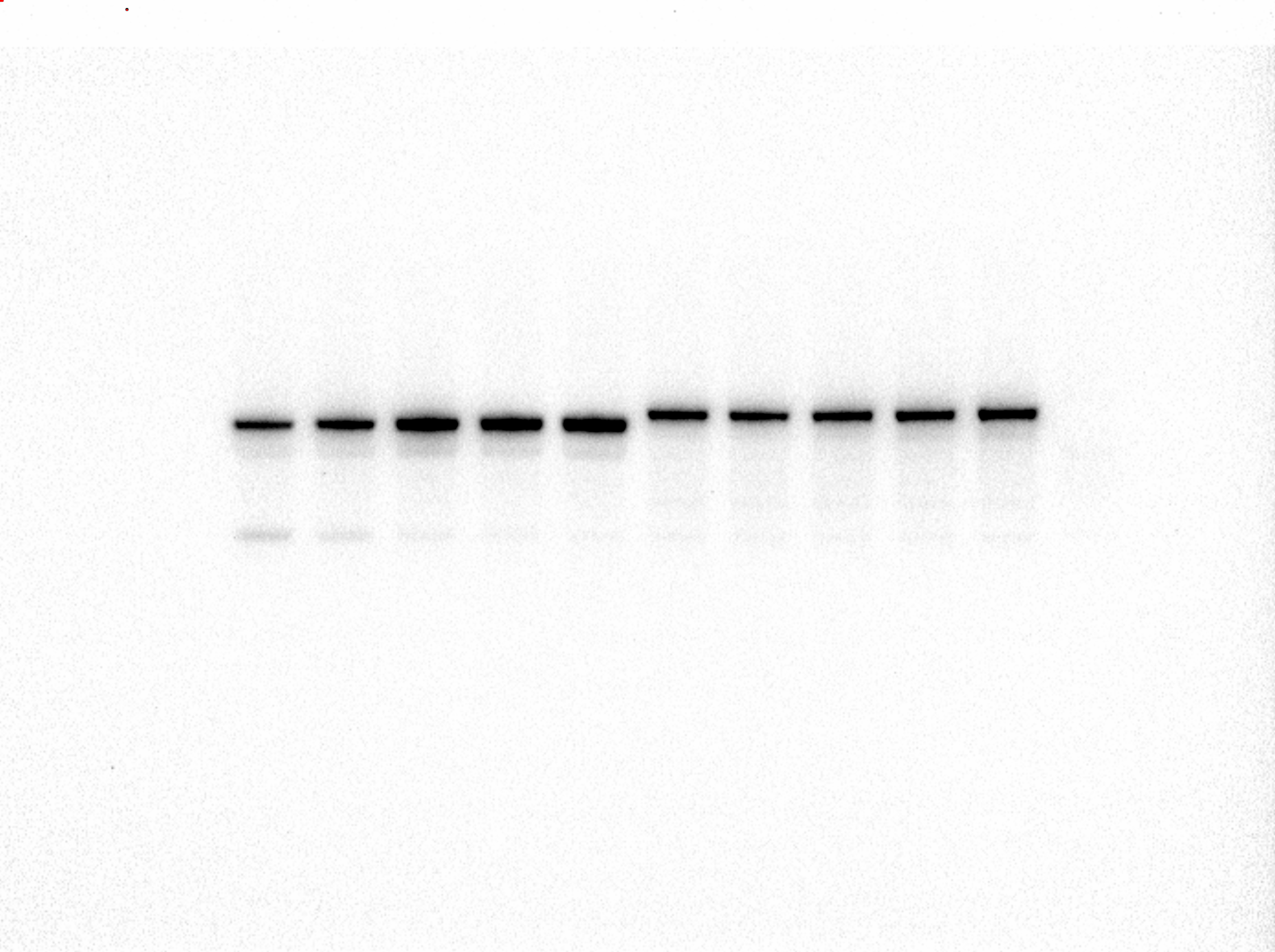

Supplement: Figure 2—source data 3. — HEK293T cells were transfected with Casp11-mCherry constructs and 6 hr post-transfection, cells were incubated with increasing amounts of pan-caspase inhibitor zVAD (0–200 μM; twofold increments). Whole-cell lysates were isolated 12 hr post-transfection and immunoblotted for mCherry or β-actin as loading control as indicated. Cleaved p10-mCherry is denoted. [file elife-83725-fig2-data3.zip › mCherry.tif]

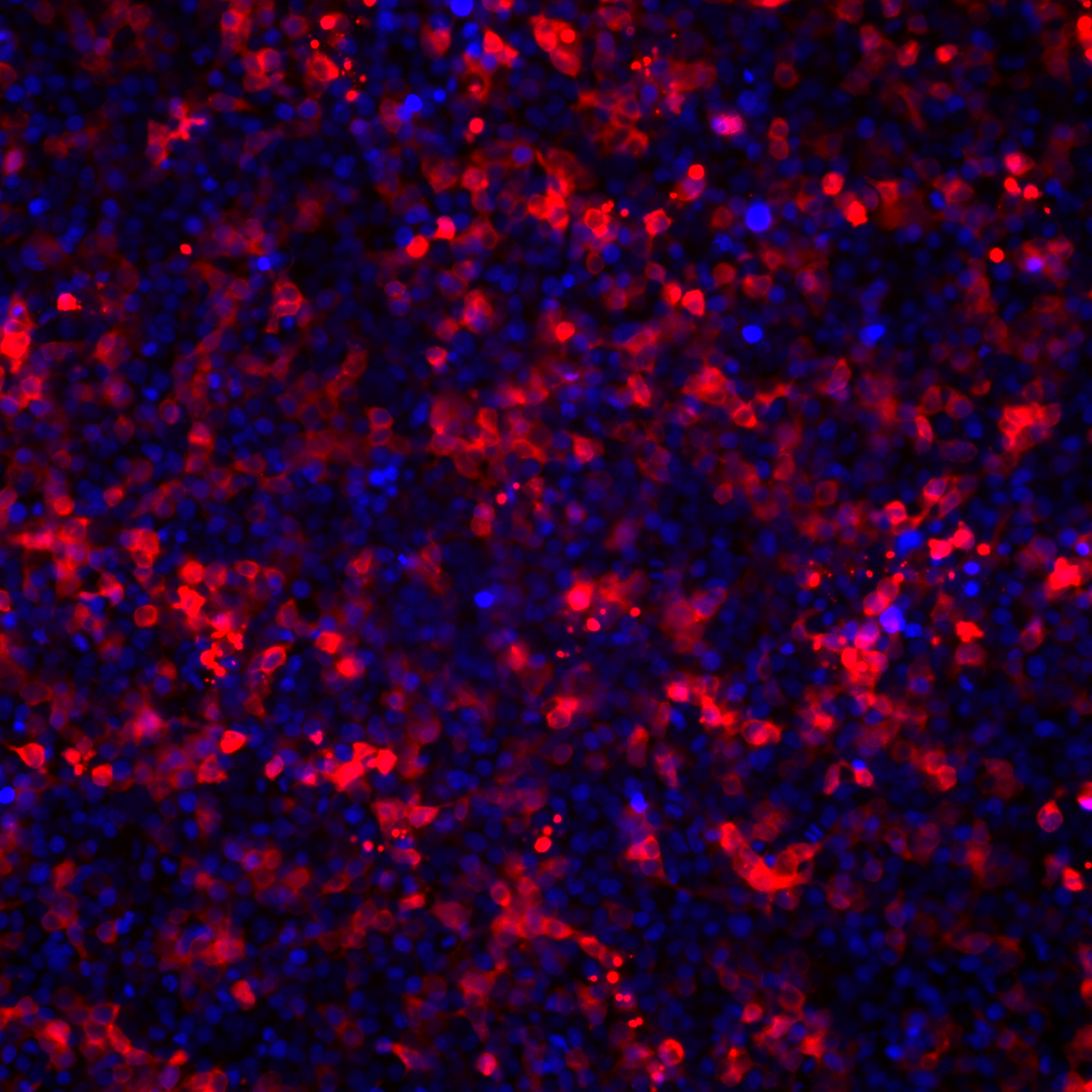

Supplement: Figure 2—source data 4. — Casp11-mCherry speck formation was assayed in zVAD-treated cells by fluorescence microscopy. Nuclei are stained with Hoechst. [file elife-83725-fig2-data4.zip › 0_zVAD_C11(WT)-mCh.tif]

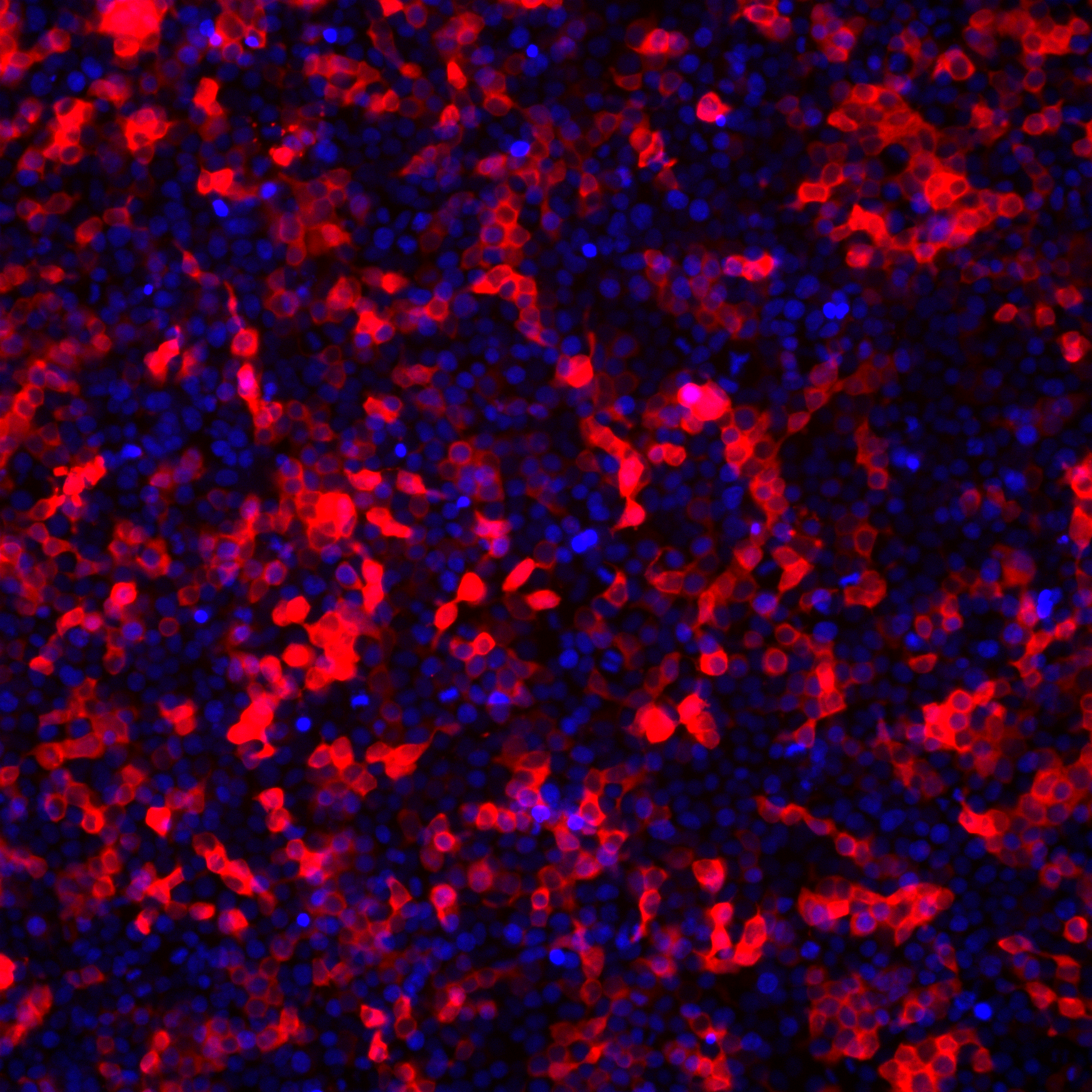

Supplement: Figure 2—source data 4. — Casp11-mCherry speck formation was assayed in zVAD-treated cells by fluorescence microscopy. Nuclei are stained with Hoechst. [file elife-83725-fig2-data4.zip › 0_zVAD_C254A-mCh.tif]

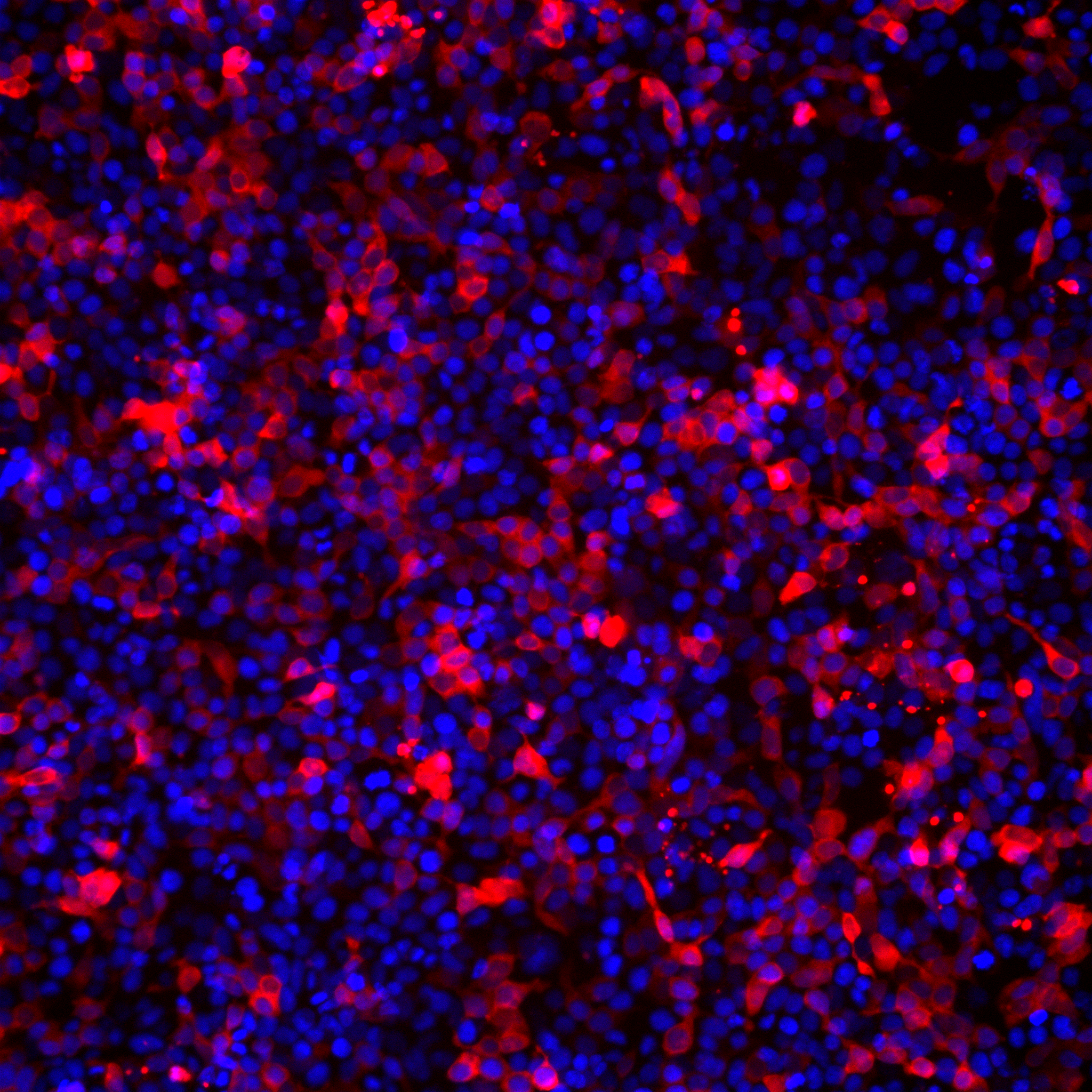

Supplement: Figure 2—source data 4. — Casp11-mCherry speck formation was assayed in zVAD-treated cells by fluorescence microscopy. Nuclei are stained with Hoechst. [file elife-83725-fig2-data4.zip › 50_zVAD_C11(WT)-mCh.tif]

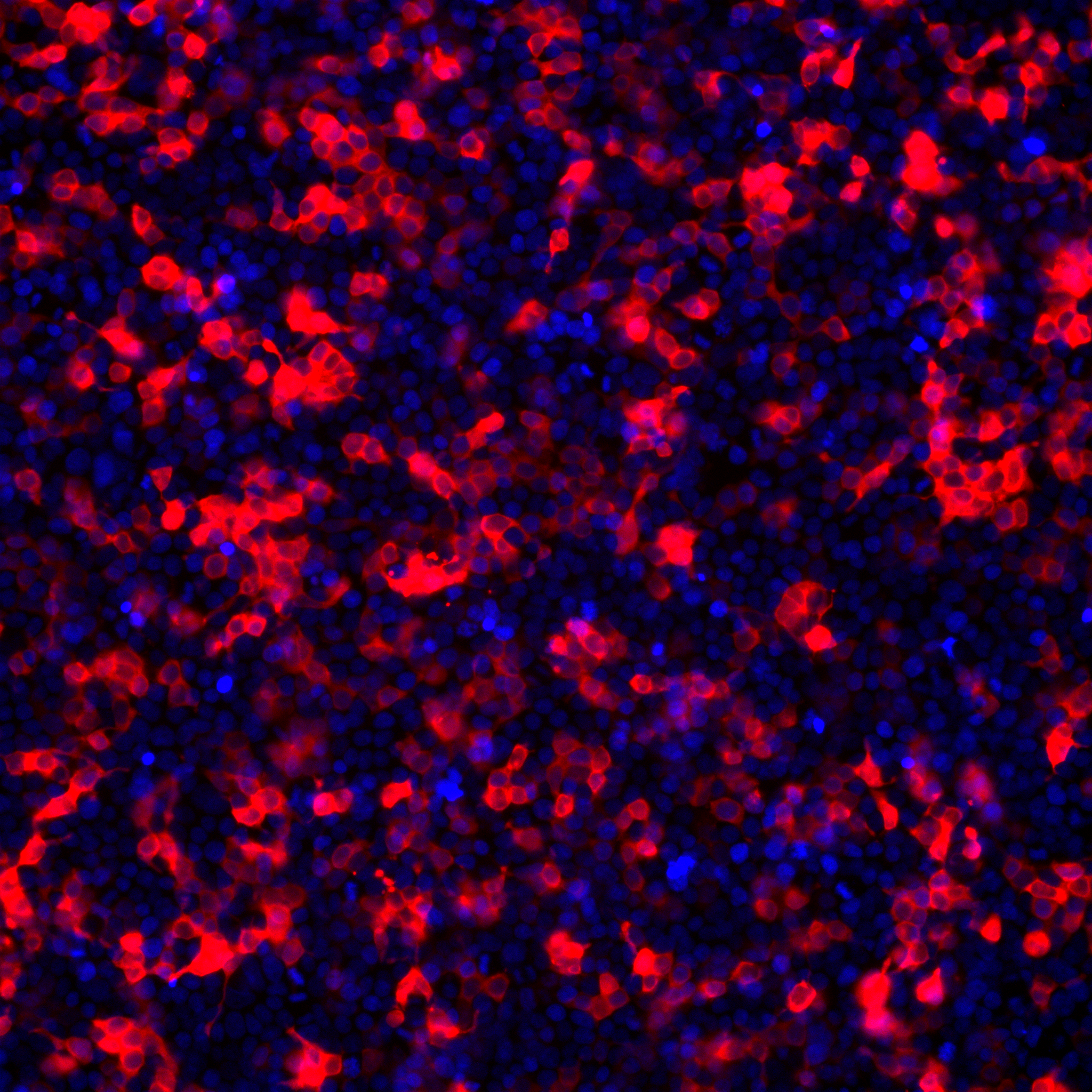

Supplement: Figure 2—source data 4. — Casp11-mCherry speck formation was assayed in zVAD-treated cells by fluorescence microscopy. Nuclei are stained with Hoechst. [file elife-83725-fig2-data4.zip › 50_zVAD_C254A-mCh.tif]

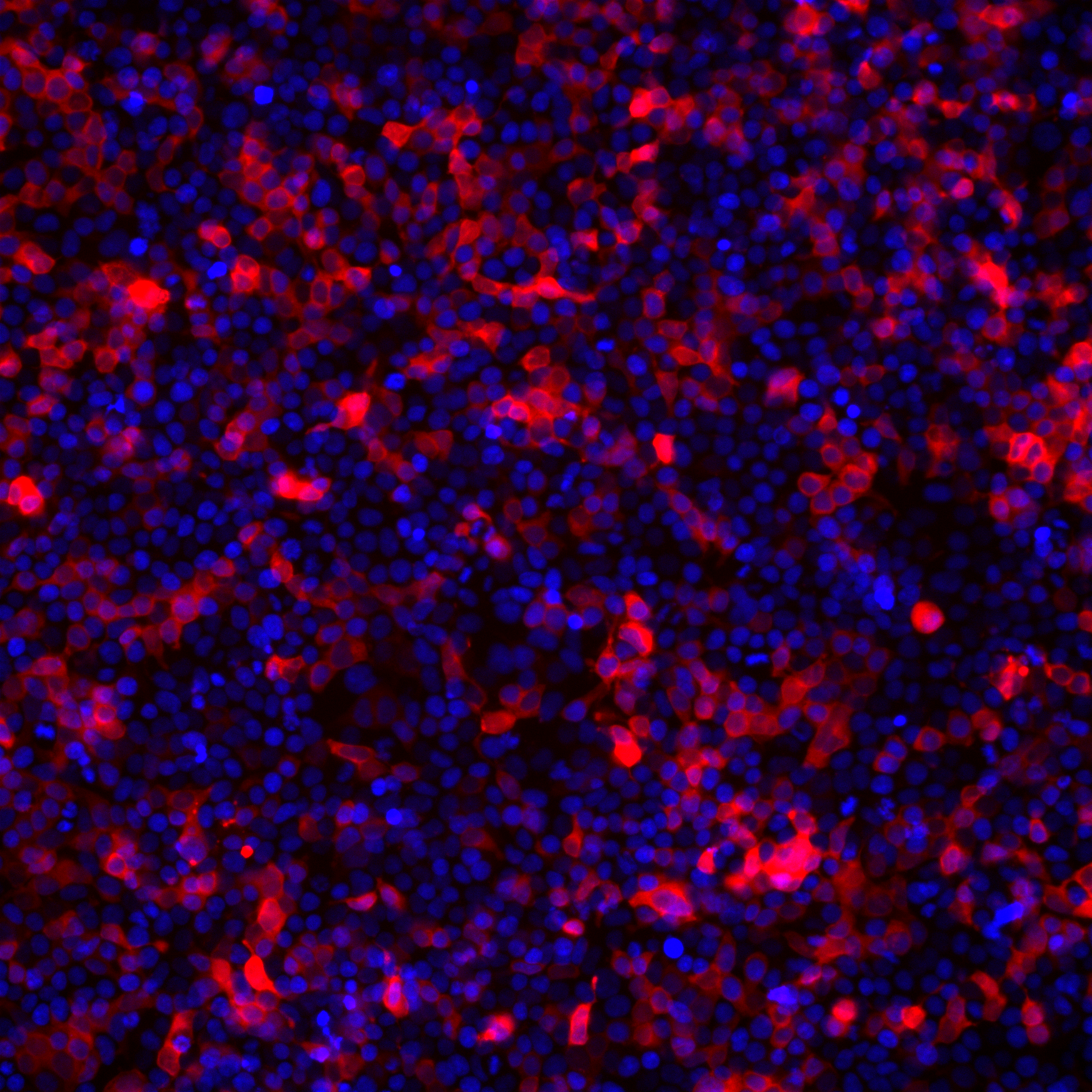

Supplement: Figure 2—source data 4. — Casp11-mCherry speck formation was assayed in zVAD-treated cells by fluorescence microscopy. Nuclei are stained with Hoechst. [file elife-83725-fig2-data4.zip › 200_zVAD_C11(WT)-mCh.tif]

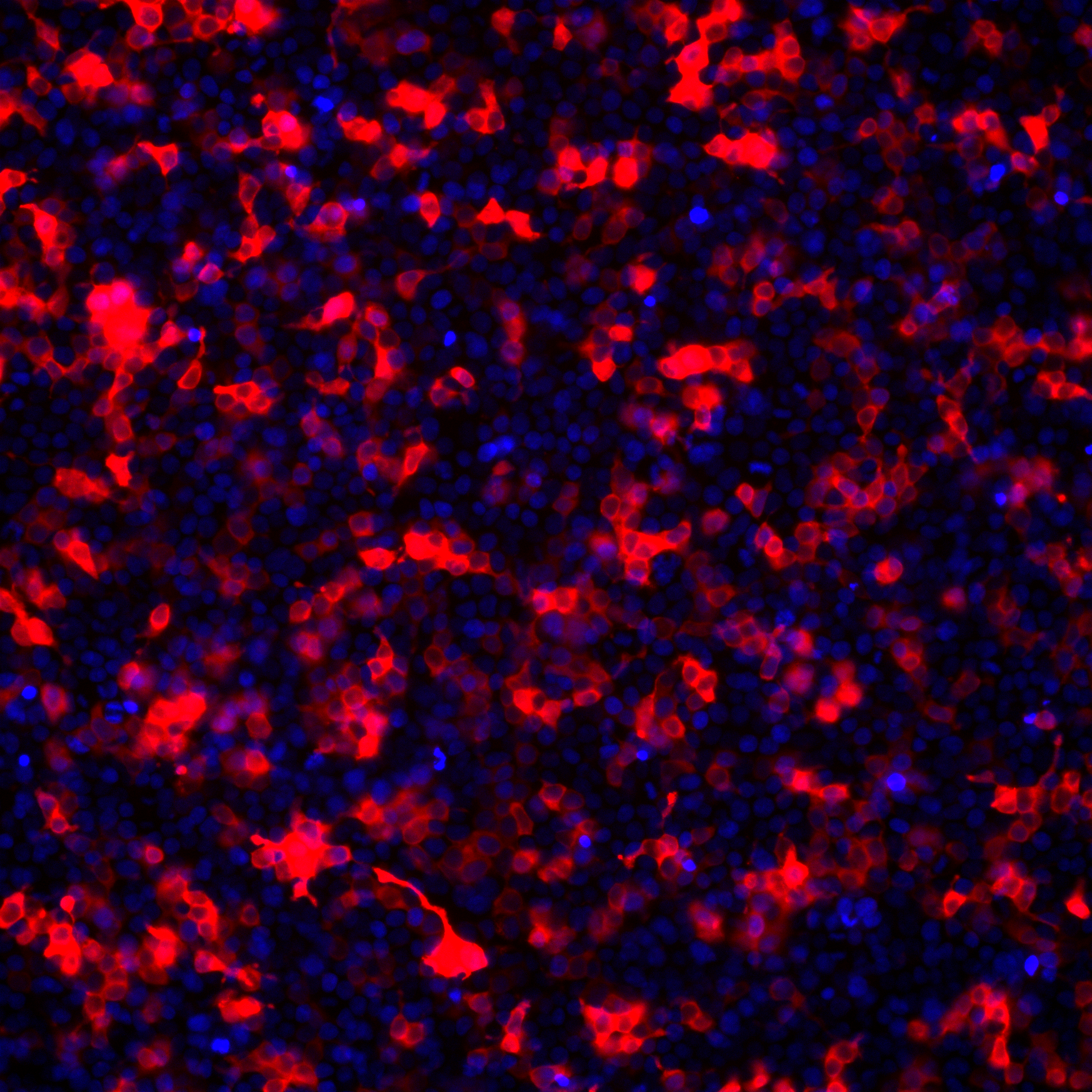

Supplement: Figure 2—source data 4. — Casp11-mCherry speck formation was assayed in zVAD-treated cells by fluorescence microscopy. Nuclei are stained with Hoechst. [file elife-83725-fig2-data4.zip › 200_zVAD_C254A-mCh.tif]

Fig 2-source data 4 (2E)

0  $\mu$ M zVAD

50  $\mu$ M zVAD

200  $\mu$ M zVAD

WT

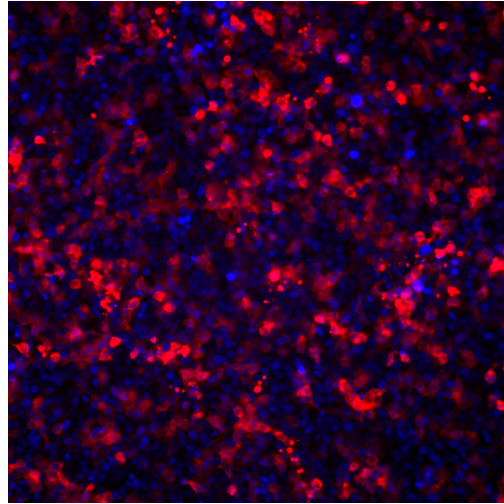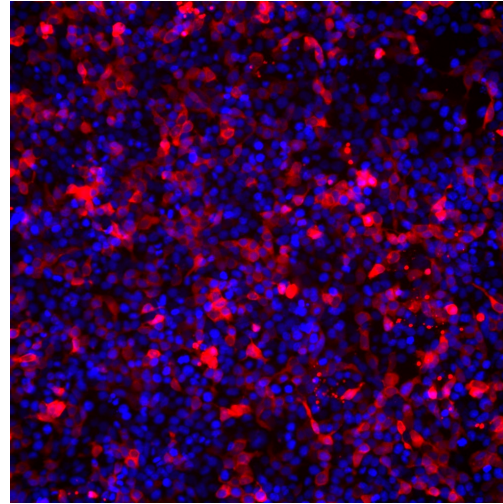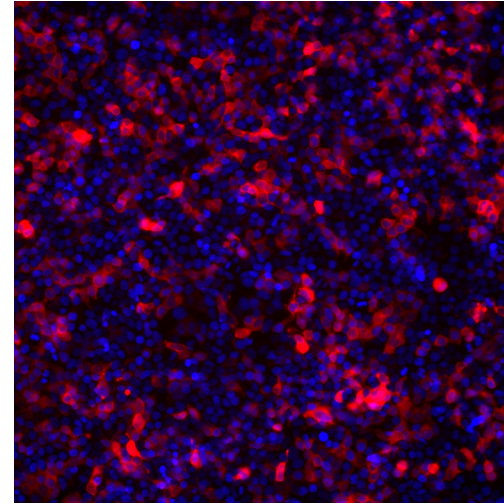

C254A

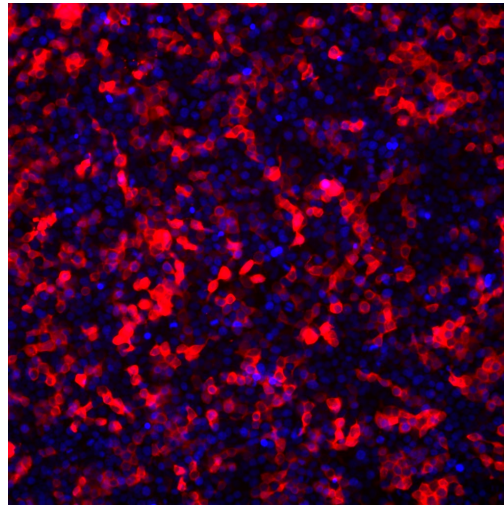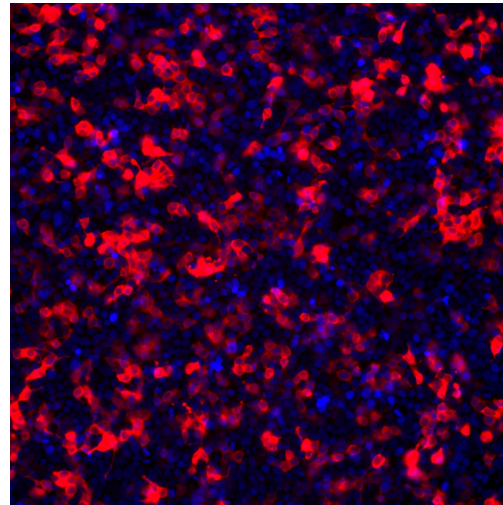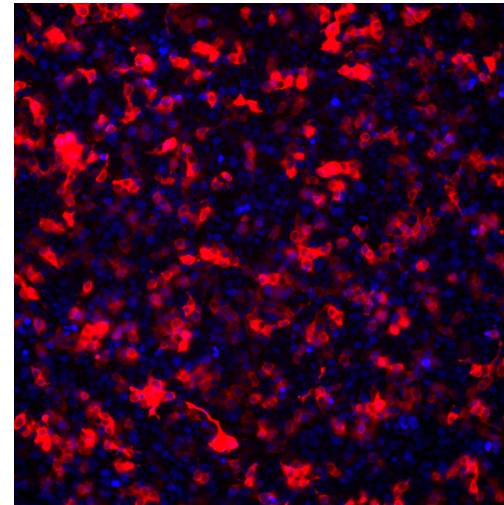

HOECHST mCHERRY

Supplement: Figure 2—source data 4. — Casp11-mCherry speck formation was assayed in zVAD-treated cells by fluorescence microscopy. Nuclei are stained with Hoechst. [file elife-83725-fig2-data4.zip › Figure 2-source data 4.pdf]

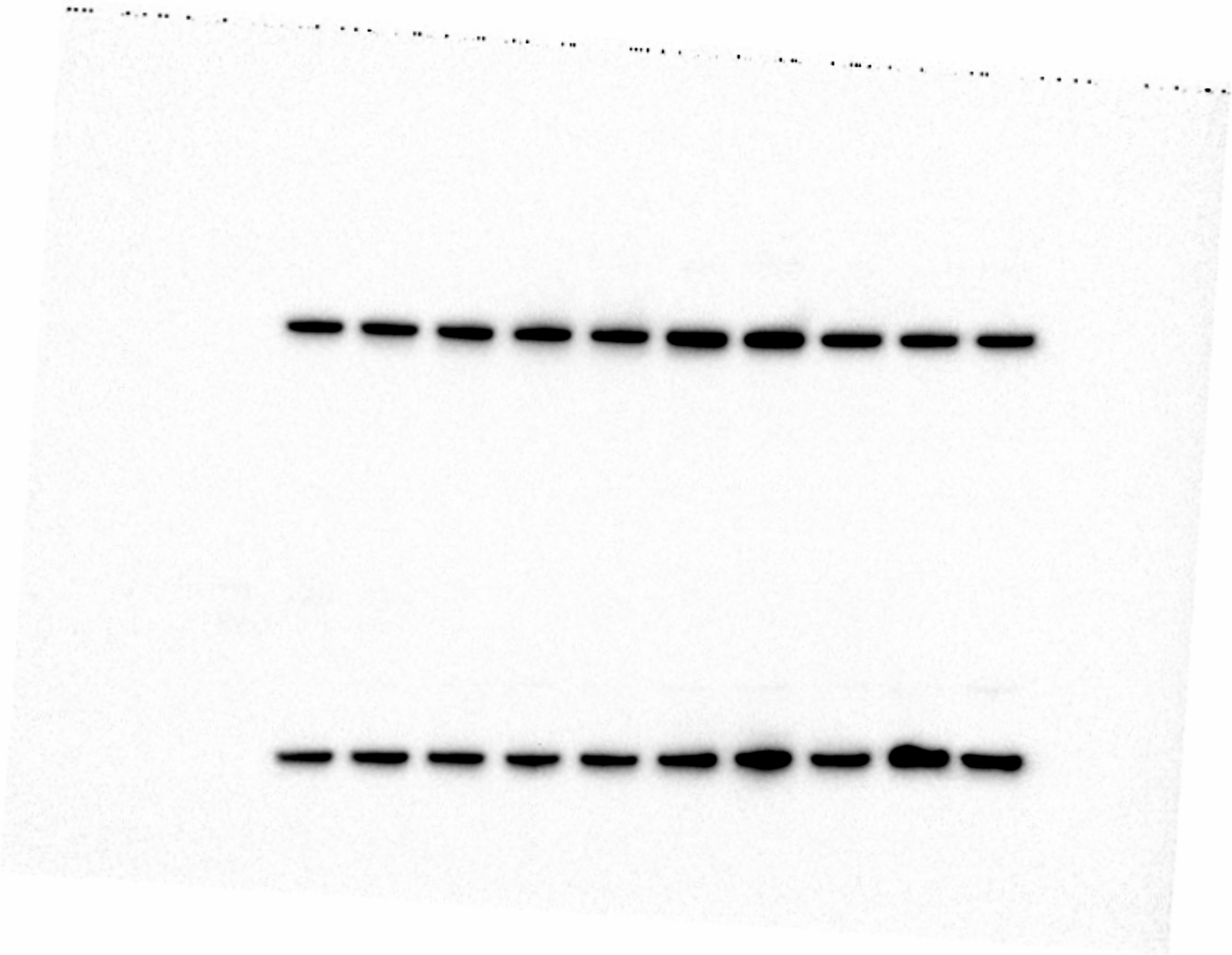

Supplement: Figure 2—figure supplement 1—source data 1. — Gasdermin D (GSDMD) expression plasmid was co-transfected with increasing doses of indicated Casp11 construct in HEK293T cells. WT untagged Casp11 was included as positive control. [file elife-83725-fig2-figsupp1-data1.zip › Actin.tif]

Figure 2-figure supplement 1A

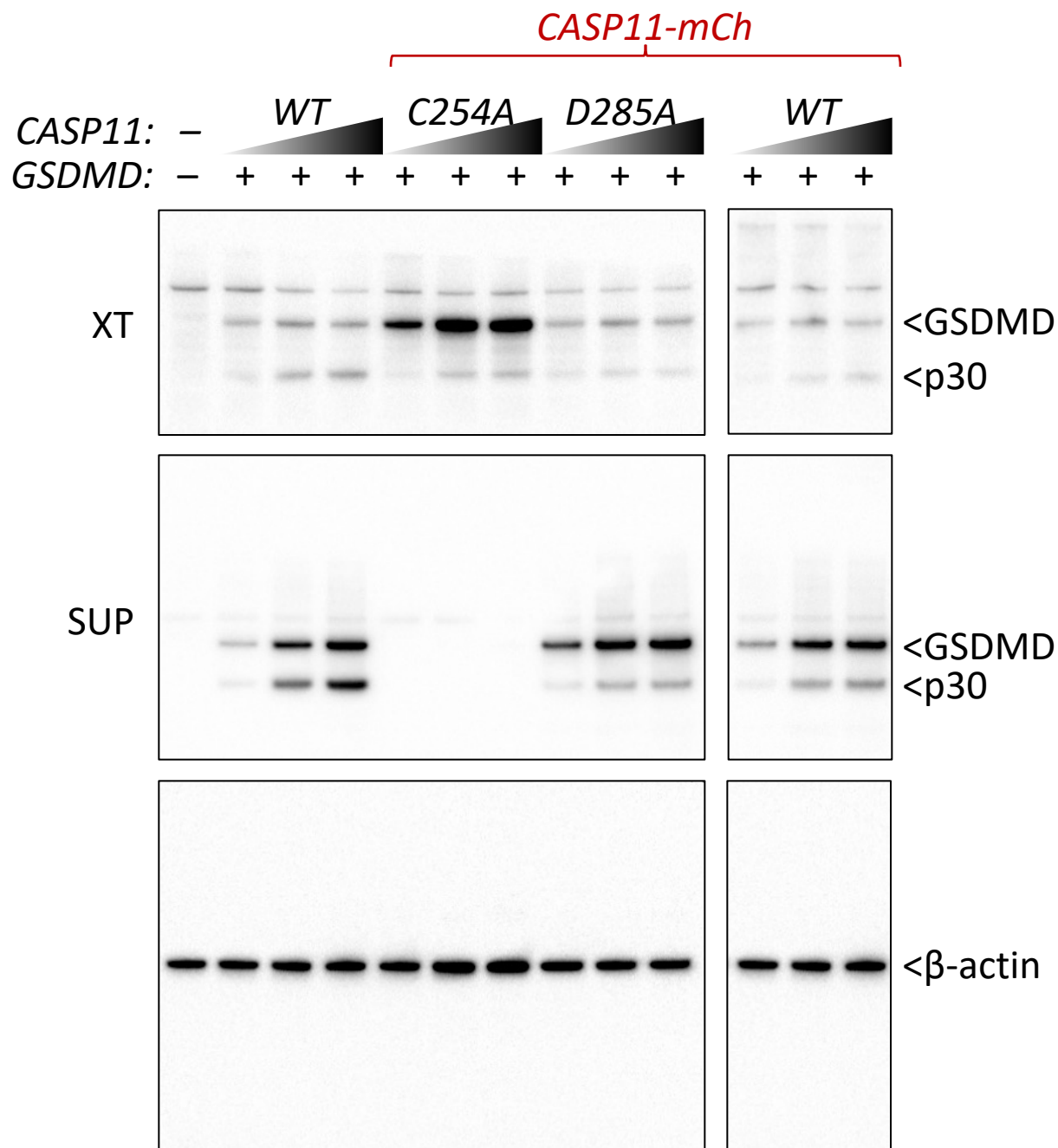

Supplement: Figure 2—figure supplement 1—source data 1. — Gasdermin D (GSDMD) expression plasmid was co-transfected with increasing doses of indicated Casp11 construct in HEK293T cells. WT untagged Casp11 was included as positive control. [file elife-83725-fig2-figsupp1-data1.zip › Figure 2-figure supplement 1-source data 1.pdf]

## Slide 1
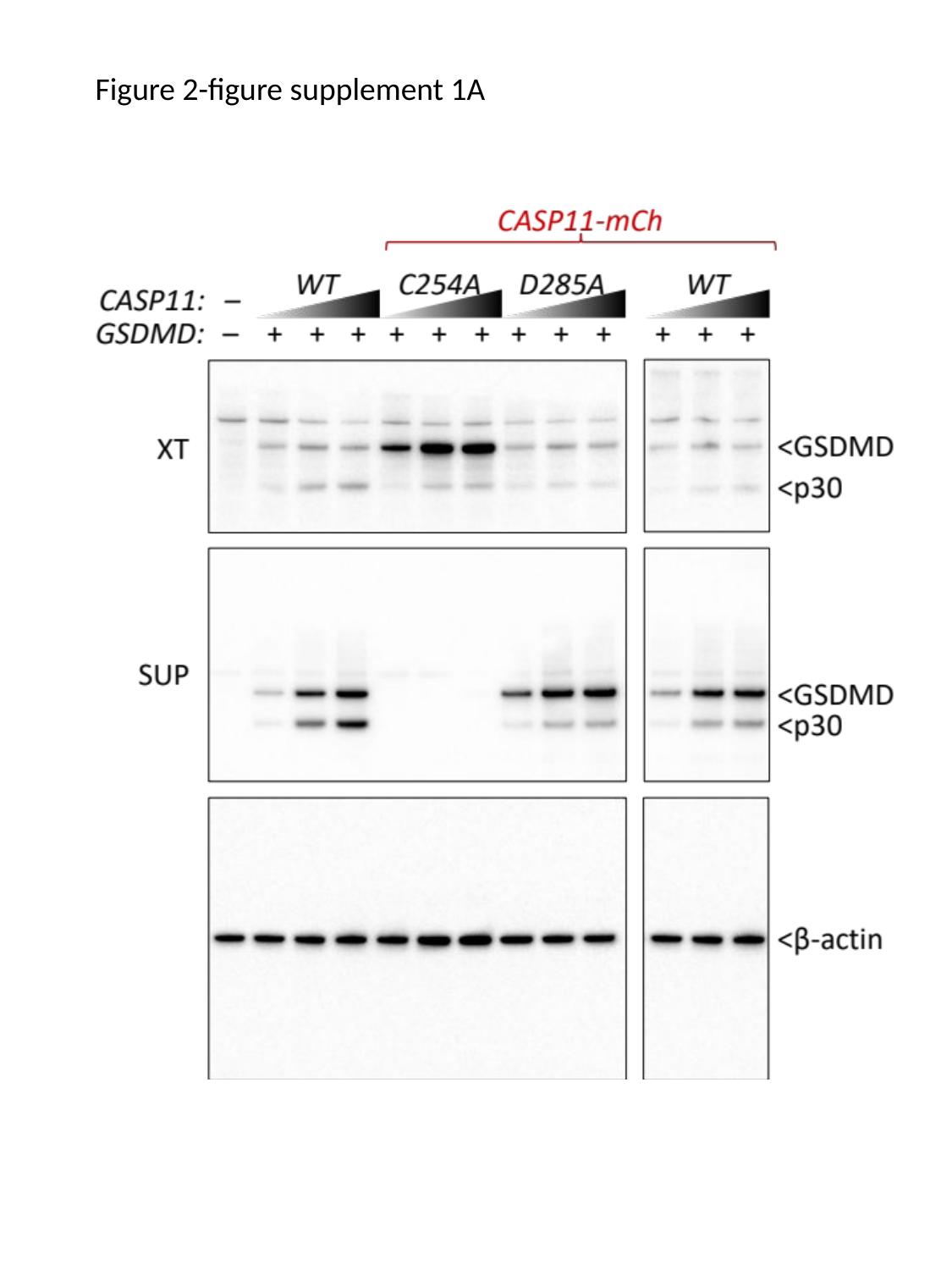

Figure 2-figure supplement 1A

Supplement: Figure 2—figure supplement 1—source data 1. — Gasdermin D (GSDMD) expression plasmid was co-transfected with increasing doses of indicated Casp11 construct in HEK293T cells. WT untagged Casp11 was included as positive control. [file elife-83725-fig2-figsupp1-data1.zip › Figure2-figure supplement 1A_labeled.pptx]

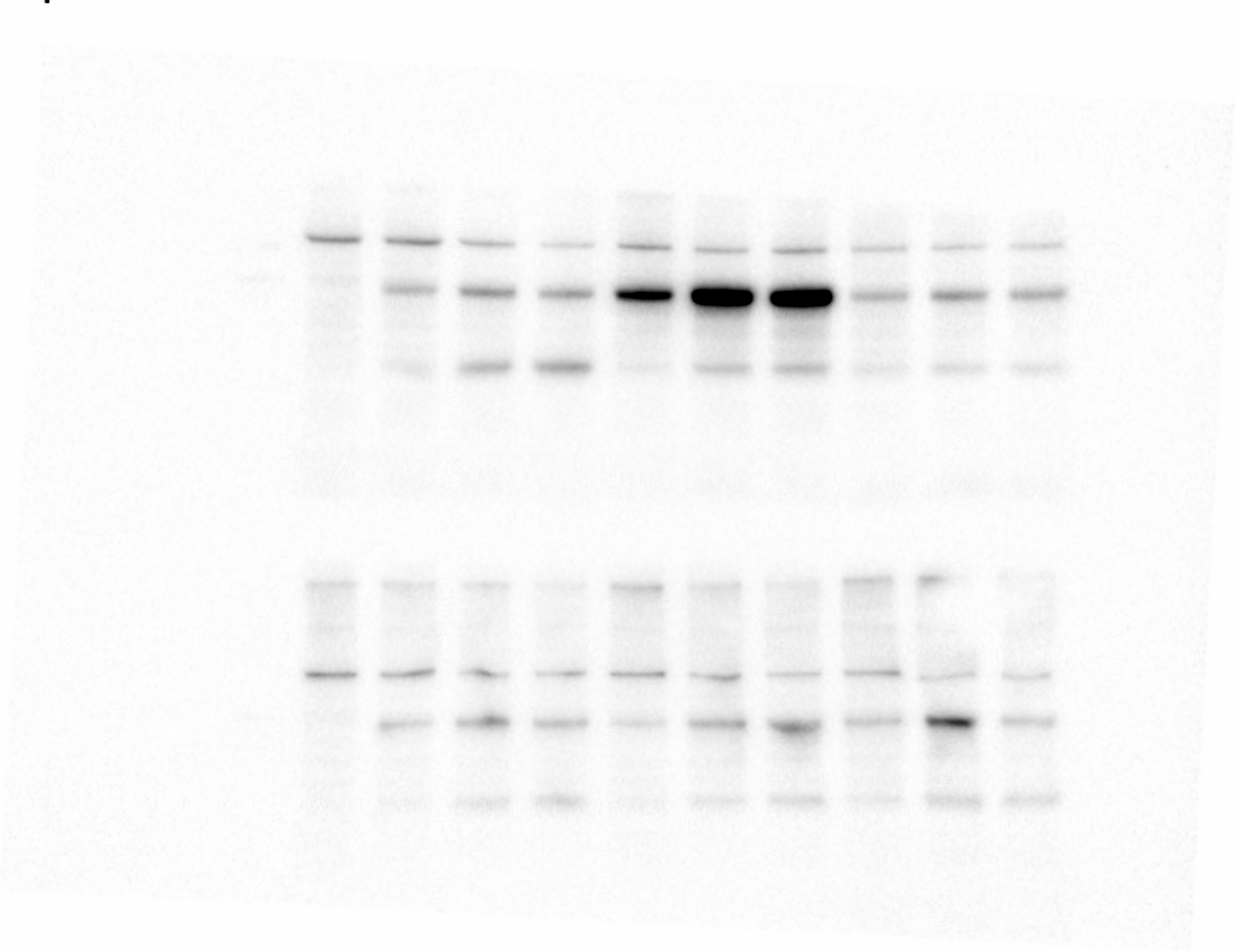

Supplement: Figure 2—figure supplement 1—source data 1. — Gasdermin D (GSDMD) expression plasmid was co-transfected with increasing doses of indicated Casp11 construct in HEK293T cells. WT untagged Casp11 was included as positive control. [file elife-83725-fig2-figsupp1-data1.zip › GSDMD_lysate.tif]

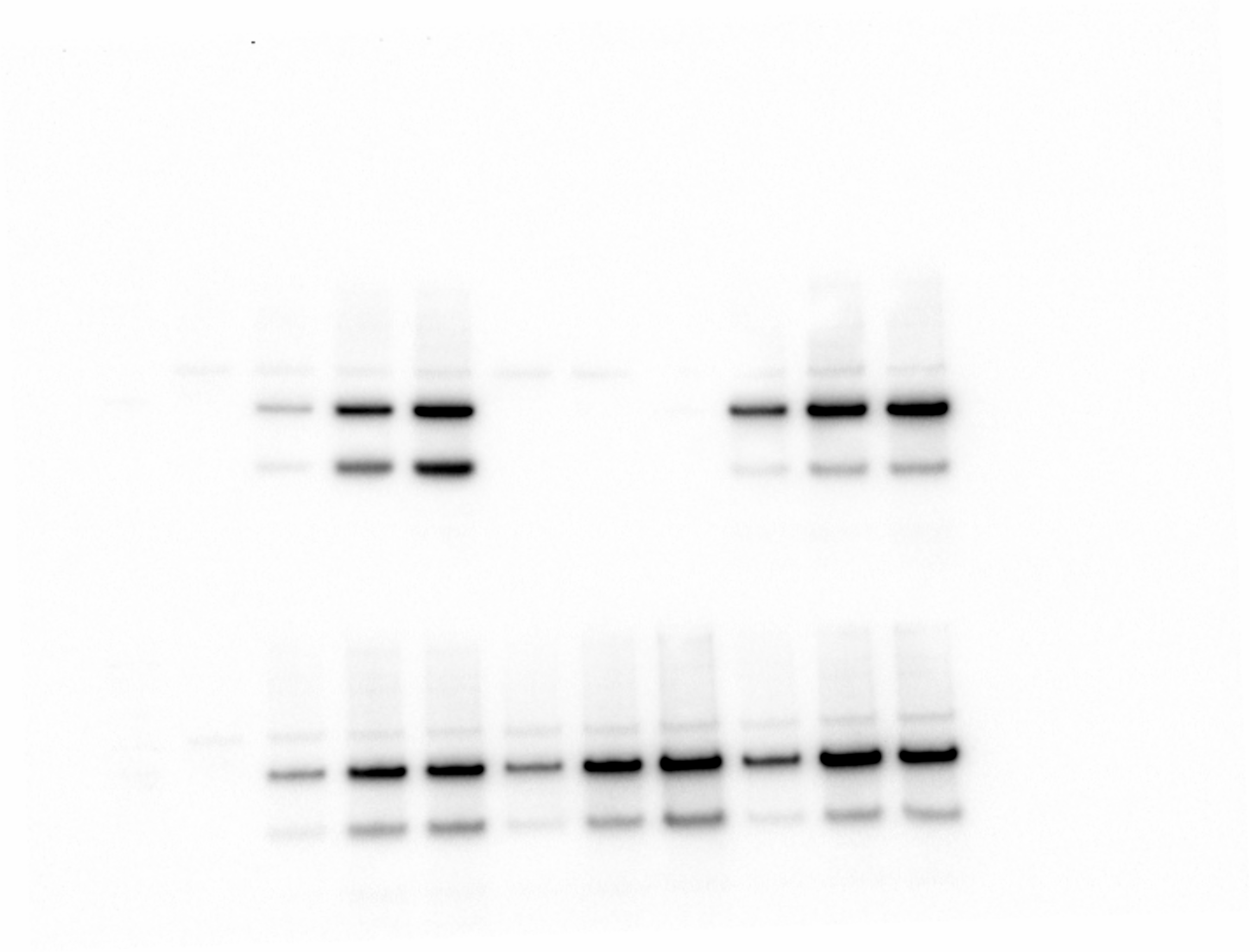

Supplement: Figure 2—figure supplement 1—source data 1. — Gasdermin D (GSDMD) expression plasmid was co-transfected with increasing doses of indicated Casp11 construct in HEK293T cells. WT untagged Casp11 was included as positive control. [file elife-83725-fig2-figsupp1-data1.zip › GSDMD_sup.tif]

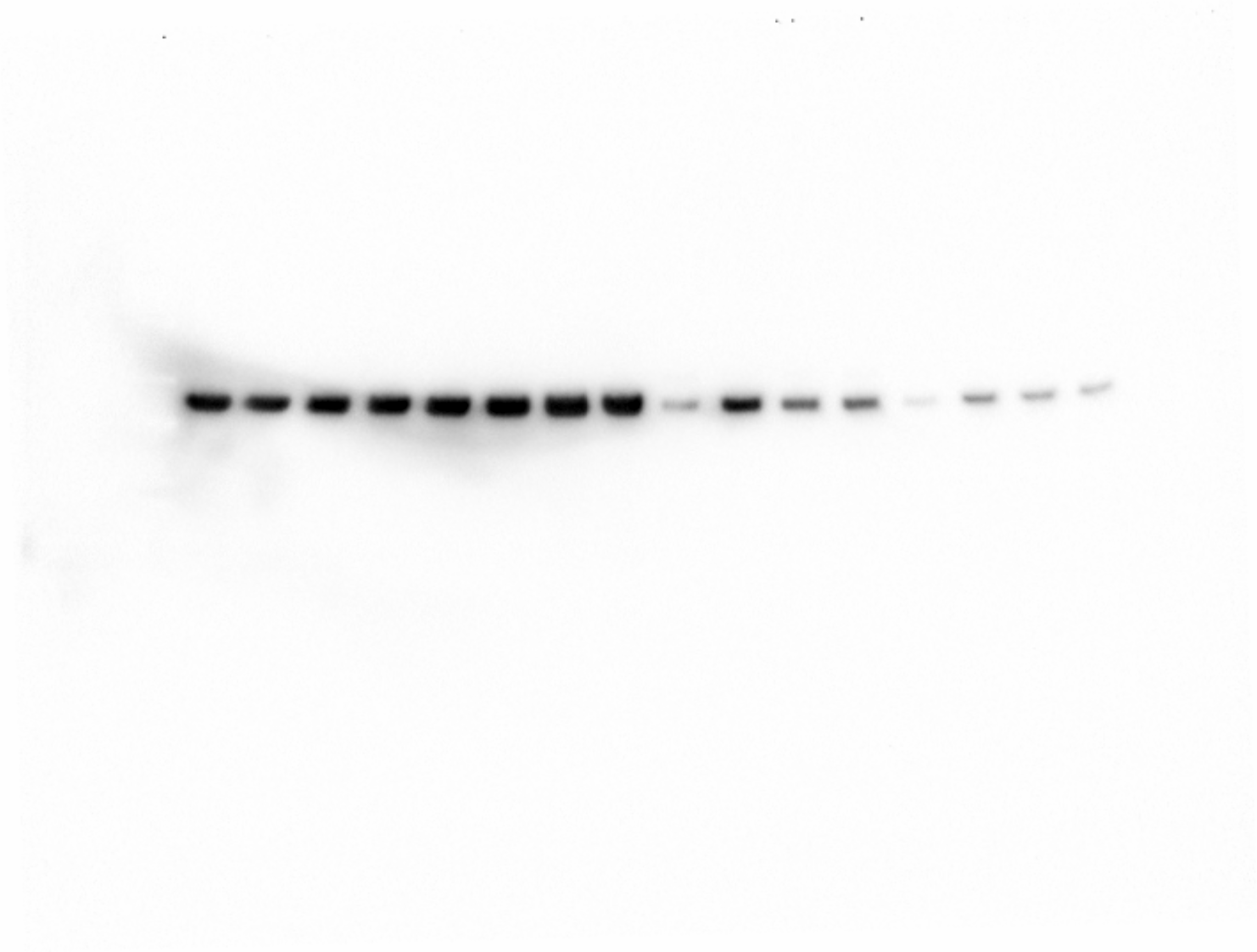

Supplement: Figure 2—figure supplement 2—source data 1. — Immunoblot for gasdermin D (GSDMD) cleavage in supernatants (sup) and whole-cell lysates (XT). β-actin is indicated as loading control. [file elife-83725-fig2-figsupp2-data1.zip › Actin.tif]

Figure 2-figure supplement 2C

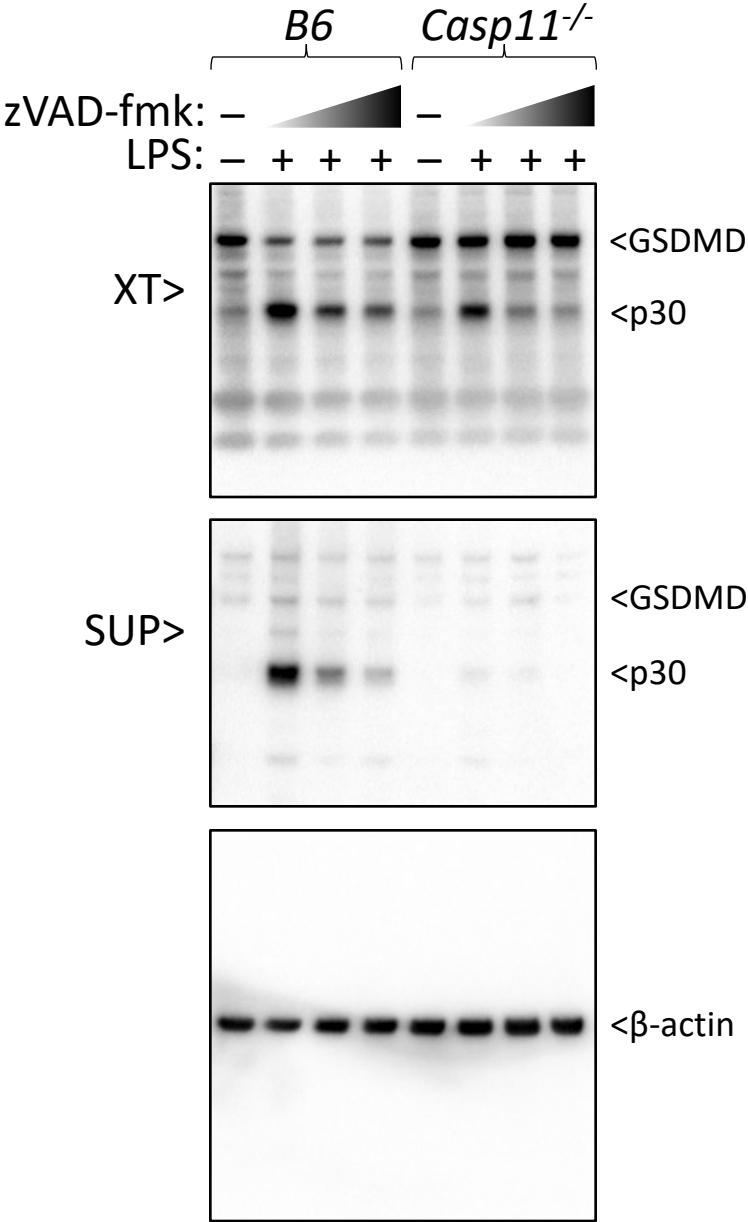

Supplement: Figure 2—figure supplement 2—source data 1. — Immunoblot for gasdermin D (GSDMD) cleavage in supernatants (sup) and whole-cell lysates (XT). β-actin is indicated as loading control. [file elife-83725-fig2-figsupp2-data1.zip › Figure 2-figure supplement 2-source data 1.pdf]

## Slide 1
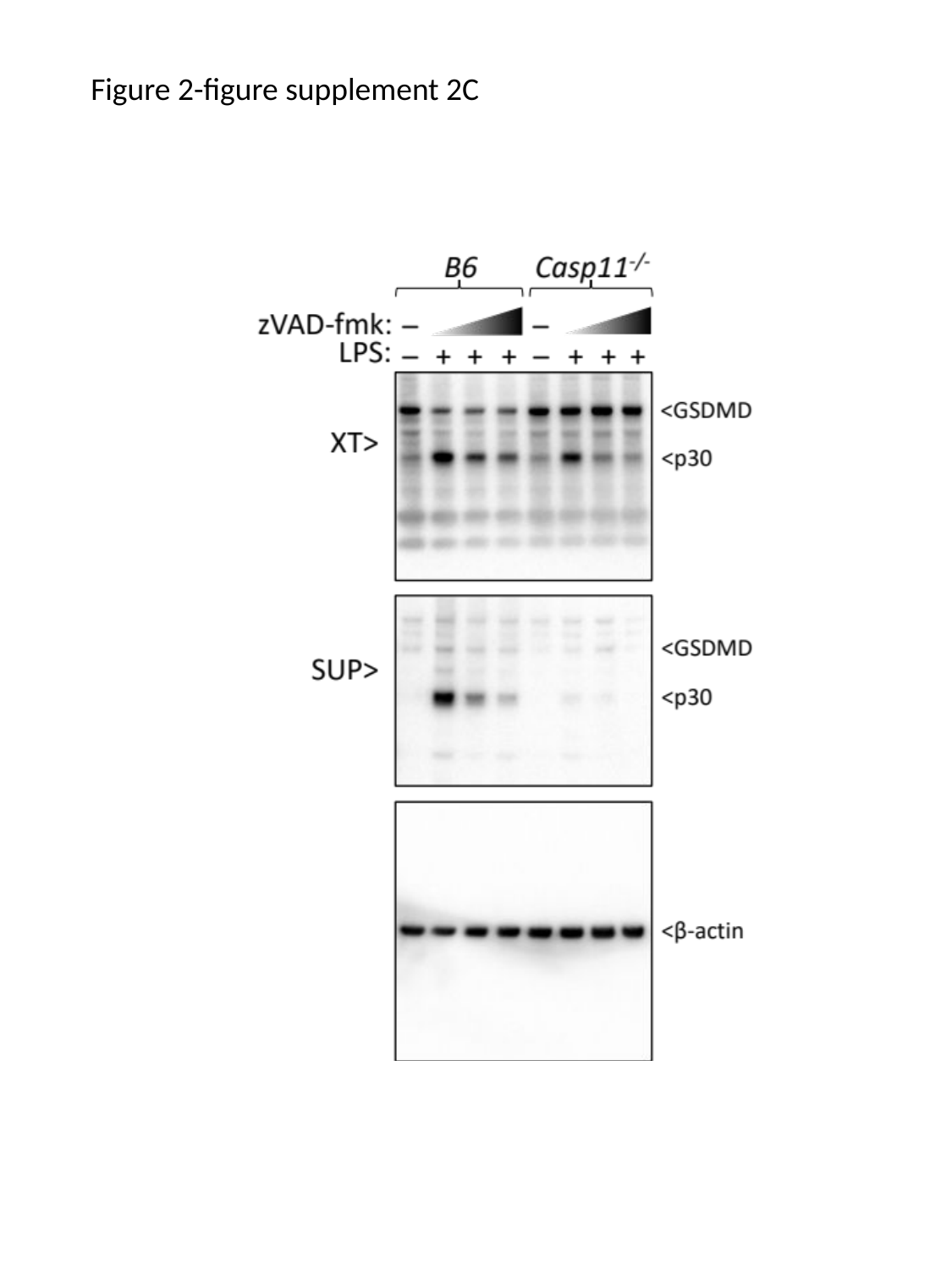

Figure 2-figure supplement 2C

Supplement: Figure 2—figure supplement 2—source data 1. — Immunoblot for gasdermin D (GSDMD) cleavage in supernatants (sup) and whole-cell lysates (XT). β-actin is indicated as loading control. [file elife-83725-fig2-figsupp2-data1.zip › Figure2-figure supplement 2C_labeled.pptx]

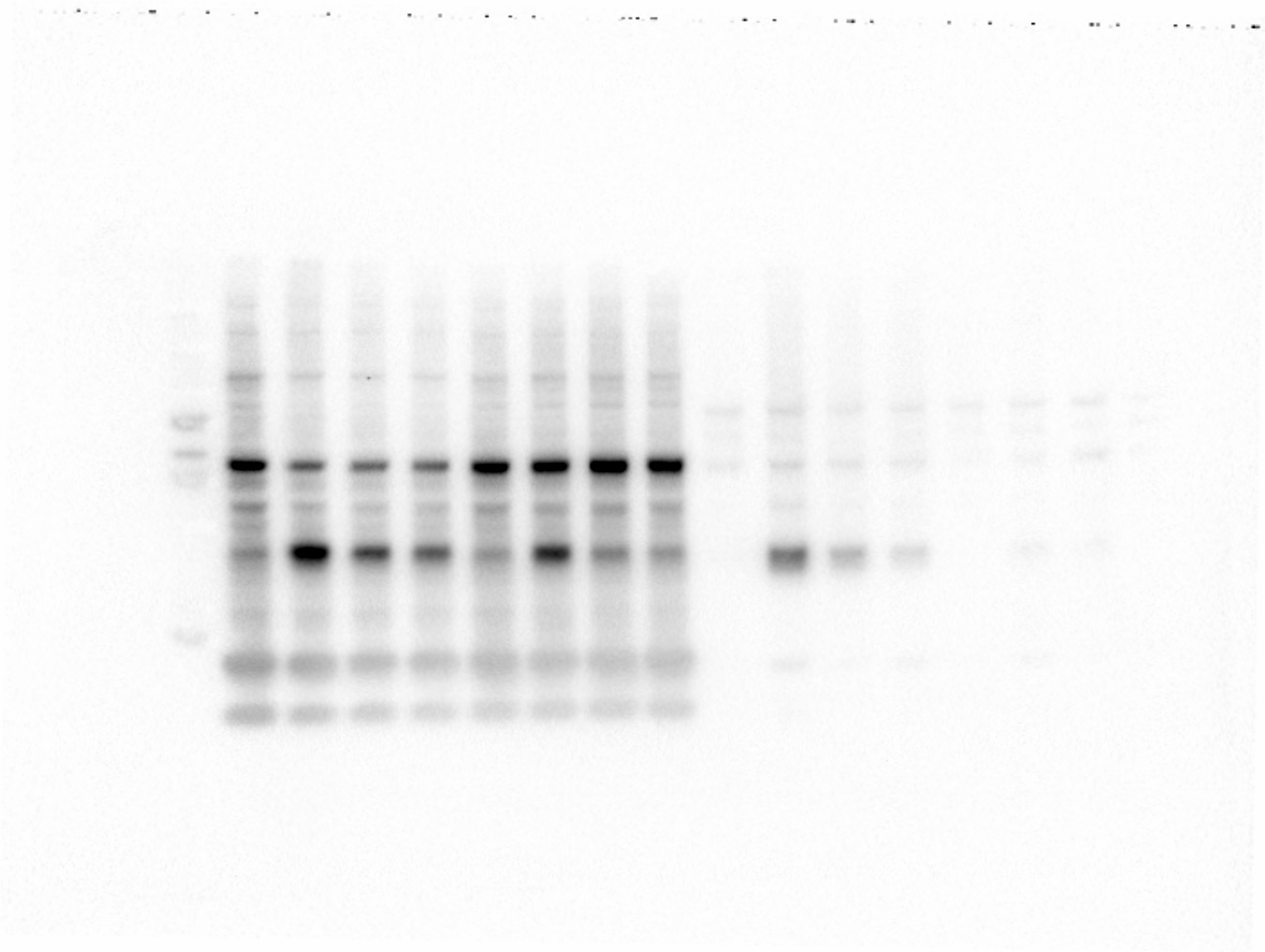

Supplement: Figure 2—figure supplement 2—source data 1. — Immunoblot for gasdermin D (GSDMD) cleavage in supernatants (sup) and whole-cell lysates (XT). β-actin is indicated as loading control. [file elife-83725-fig2-figsupp2-data1.zip › GSDMD_lysate.tif]

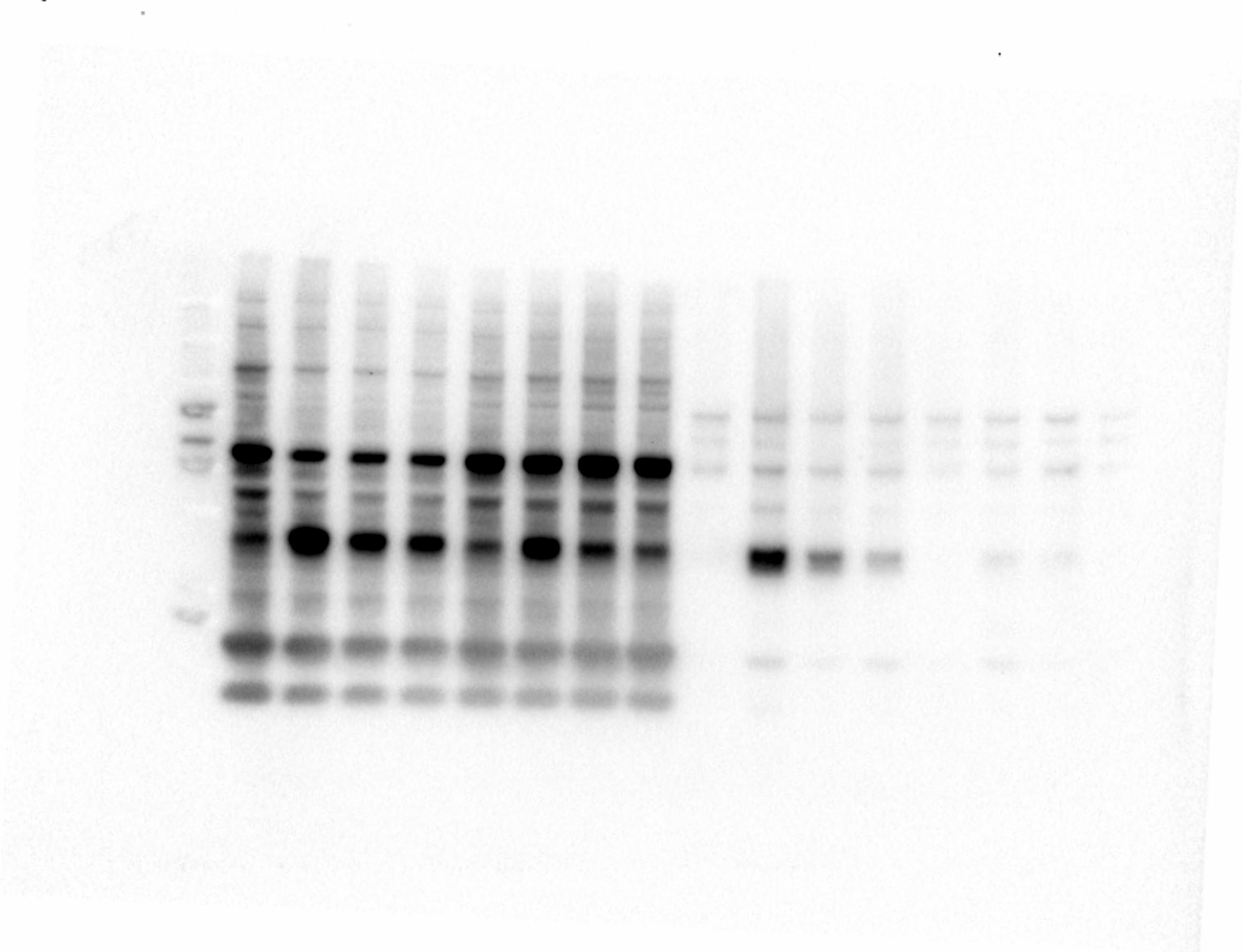

Supplement: Figure 2—figure supplement 2—source data 1. — Immunoblot for gasdermin D (GSDMD) cleavage in supernatants (sup) and whole-cell lysates (XT). β-actin is indicated as loading control. [file elife-83725-fig2-figsupp2-data1.zip › GSDMD_sup.tif]

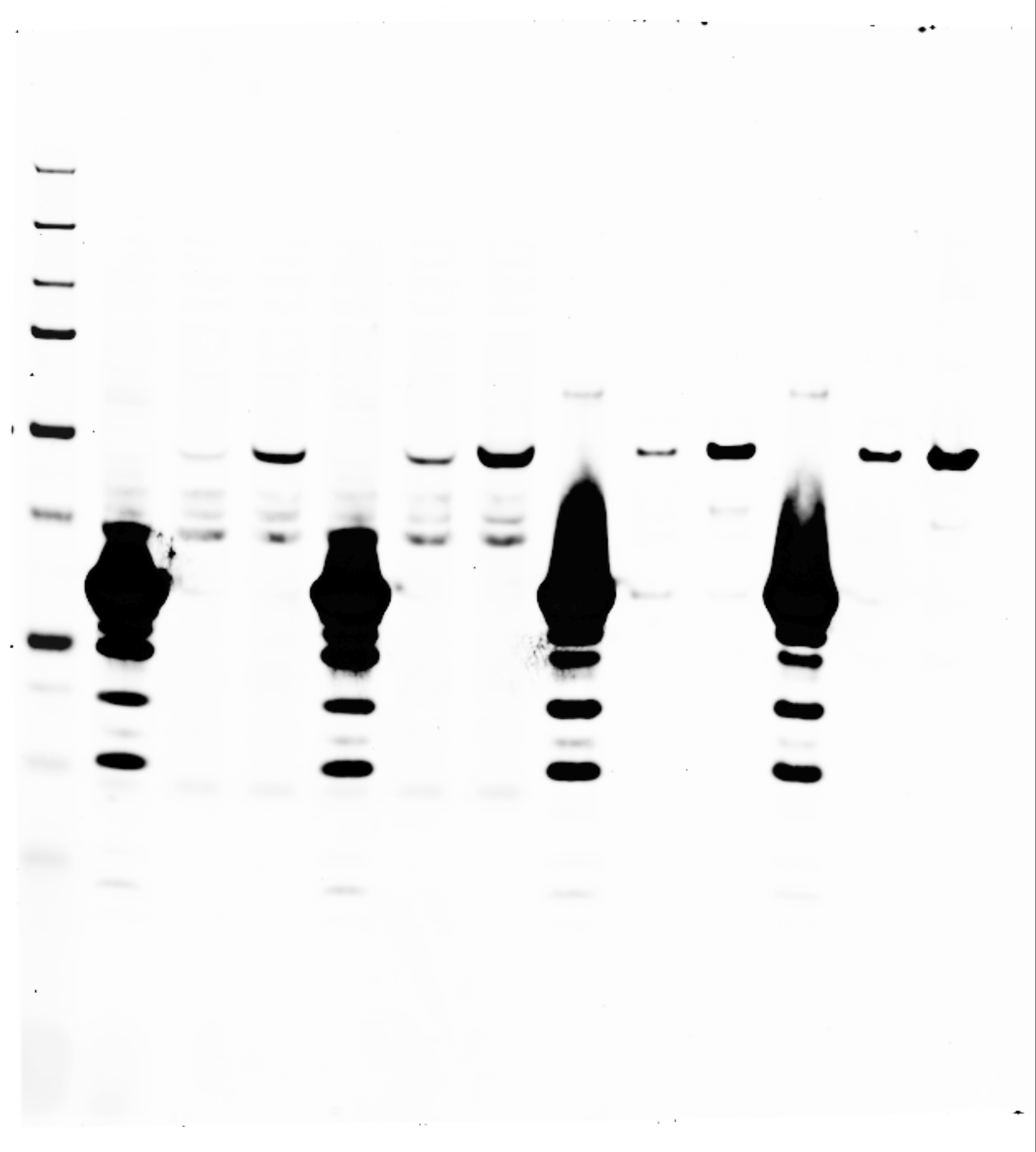

Supplement: Figure 2—figure supplement 3—source data 1. — HEK293T cells were transiently transfected with 2X-FLAG-tagged wild-type (WT) or catalytically inactive Casp11 expression plasmids alongside WT or C254A mCherry-tagged Casp11 (5 μg). 48 hr post-transfection, whole-cell lysates were immunoprecipitated by anti-FLAG antibodies as described in ‘Materials and methods,’ and immunoblotted for mCherry, FLAG, or GAPDH as a loading control, as indicated. [file elife-83725-fig2-figsupp3-data1.zip › 2xFlagC11_input_eluate.tif]

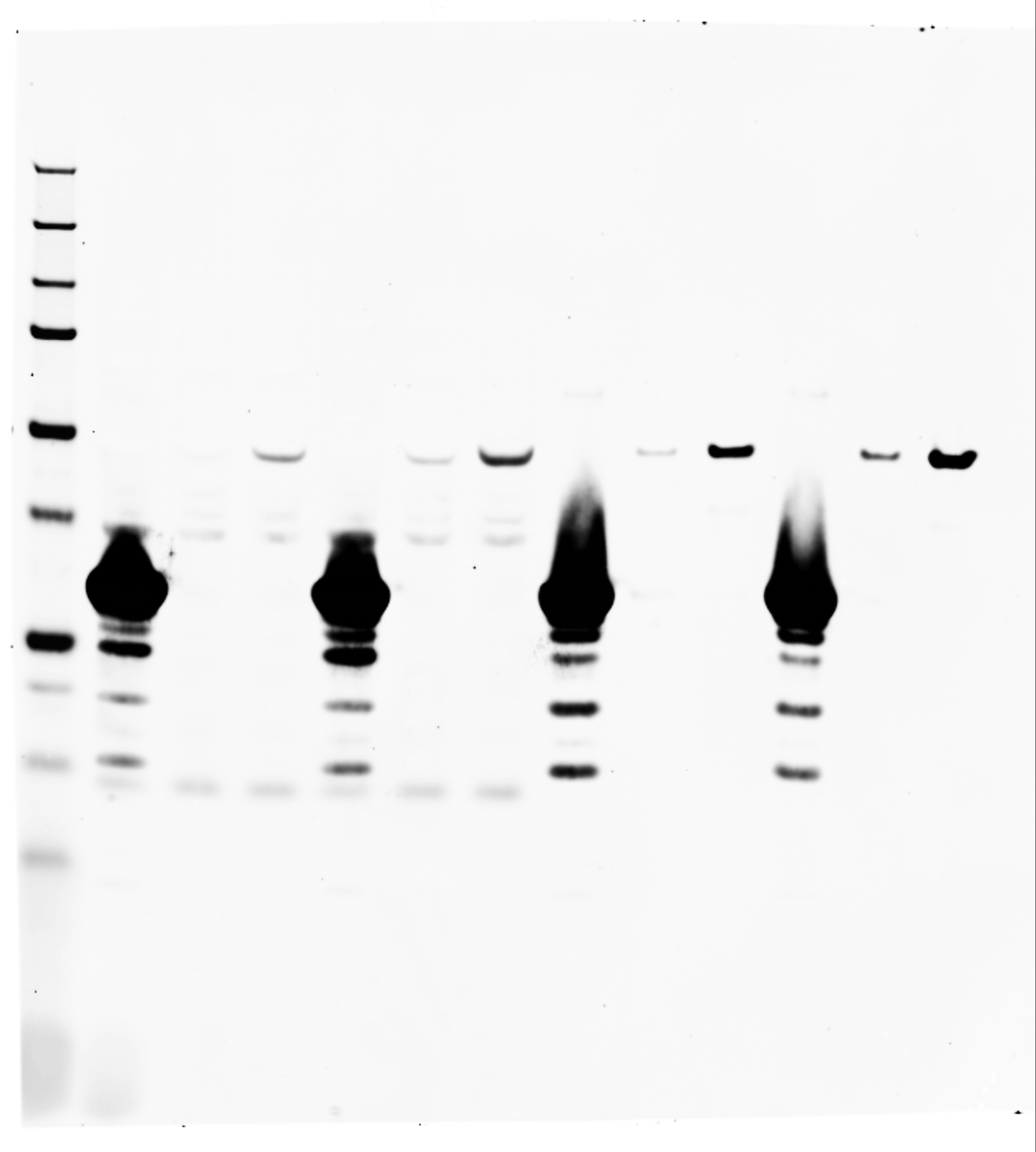

Supplement: Figure 2—figure supplement 3—source data 1. — HEK293T cells were transiently transfected with 2X-FLAG-tagged wild-type (WT) or catalytically inactive Casp11 expression plasmids alongside WT or C254A mCherry-tagged Casp11 (5 μg). 48 hr post-transfection, whole-cell lysates were immunoprecipitated by anti-FLAG antibodies as described in ‘Materials and methods,’ and immunoblotted for mCherry, FLAG, or GAPDH as a loading control, as indicated. [file elife-83725-fig2-figsupp3-data1.zip › FlagGFP.tif]

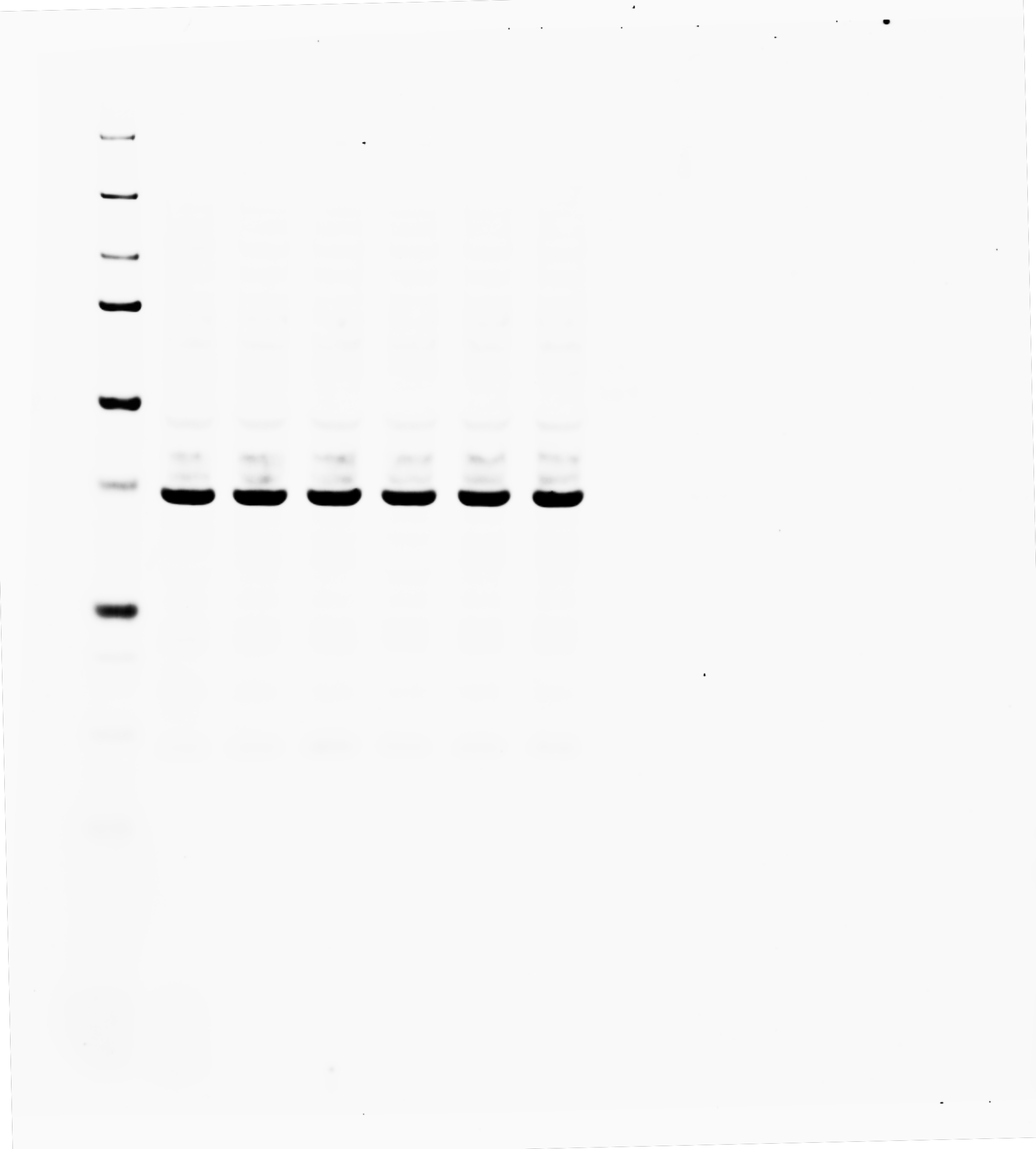

Supplement: Figure 2—figure supplement 3—source data 1. — HEK293T cells were transiently transfected with 2X-FLAG-tagged wild-type (WT) or catalytically inactive Casp11 expression plasmids alongside WT or C254A mCherry-tagged Casp11 (5 μg). 48 hr post-transfection, whole-cell lysates were immunoprecipitated by anti-FLAG antibodies as described in ‘Materials and methods,’ and immunoblotted for mCherry, FLAG, or GAPDH as a loading control, as indicated. [file elife-83725-fig2-figsupp3-data1.zip › GAPDH.tif]

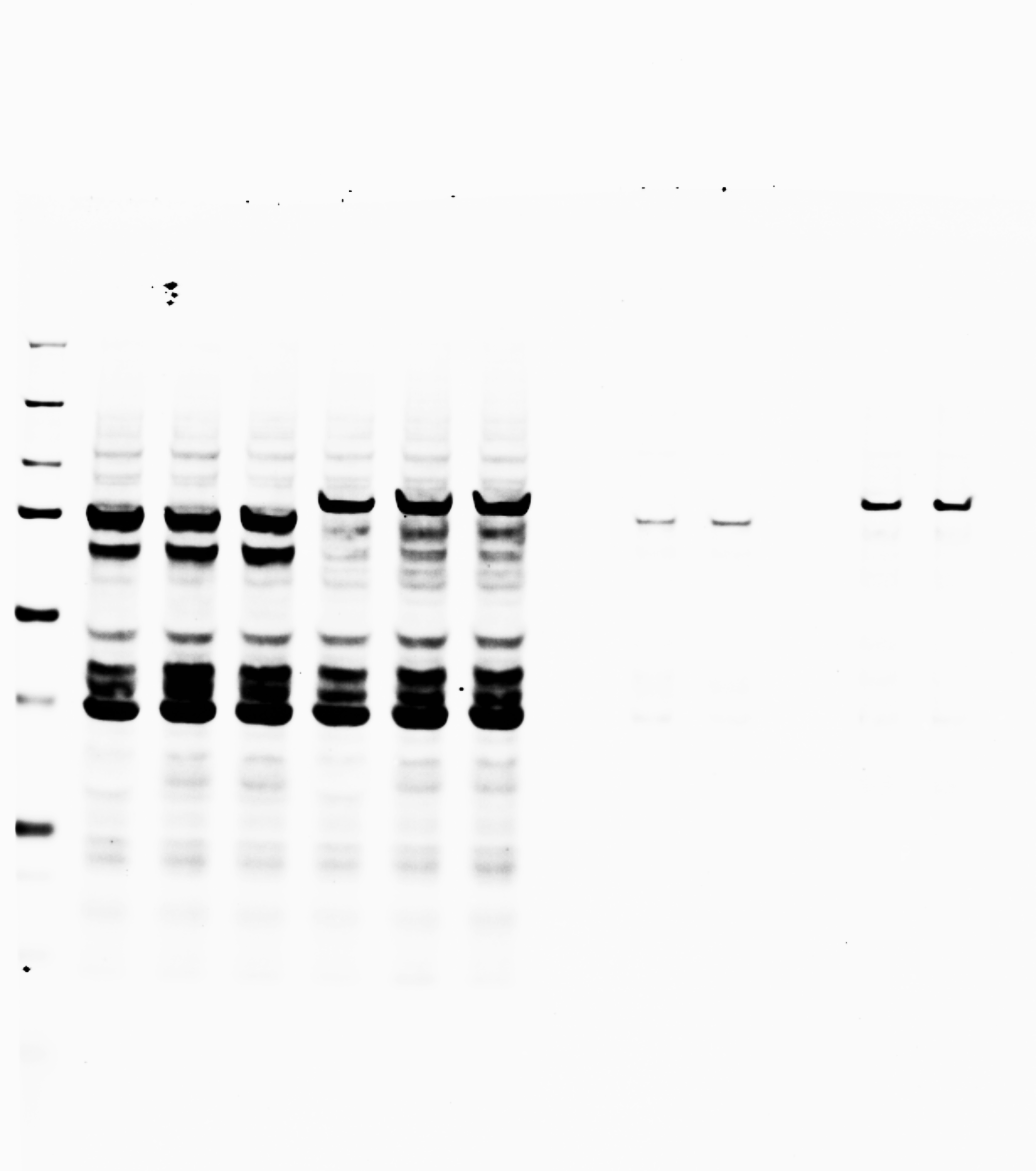

Supplement: Figure 2—figure supplement 3—source data 1. — HEK293T cells were transiently transfected with 2X-FLAG-tagged wild-type (WT) or catalytically inactive Casp11 expression plasmids alongside WT or C254A mCherry-tagged Casp11 (5 μg). 48 hr post-transfection, whole-cell lysates were immunoprecipitated by anti-FLAG antibodies as described in ‘Materials and methods,’ and immunoblotted for mCherry, FLAG, or GAPDH as a loading control, as indicated. [file elife-83725-fig2-figsupp3-data1.zip › mCherry_eluate.tif]

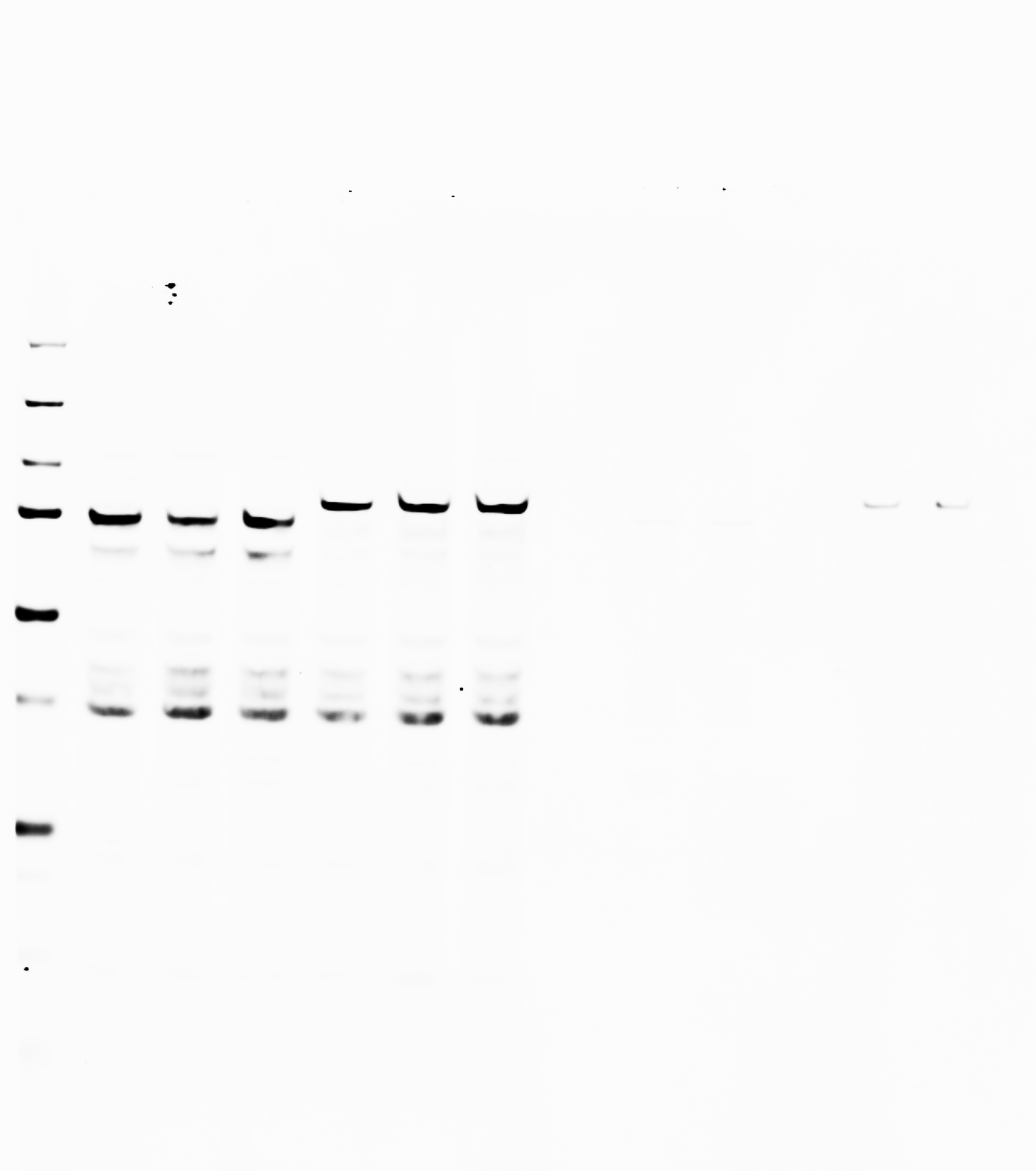

Supplement: Figure 2—figure supplement 3—source data 1. — HEK293T cells were transiently transfected with 2X-FLAG-tagged wild-type (WT) or catalytically inactive Casp11 expression plasmids alongside WT or C254A mCherry-tagged Casp11 (5 μg). 48 hr post-transfection, whole-cell lysates were immunoprecipitated by anti-FLAG antibodies as described in ‘Materials and methods,’ and immunoblotted for mCherry, FLAG, or GAPDH as a loading control, as indicated. [file elife-83725-fig2-figsupp3-data1.zip › mCherry_input.tif]

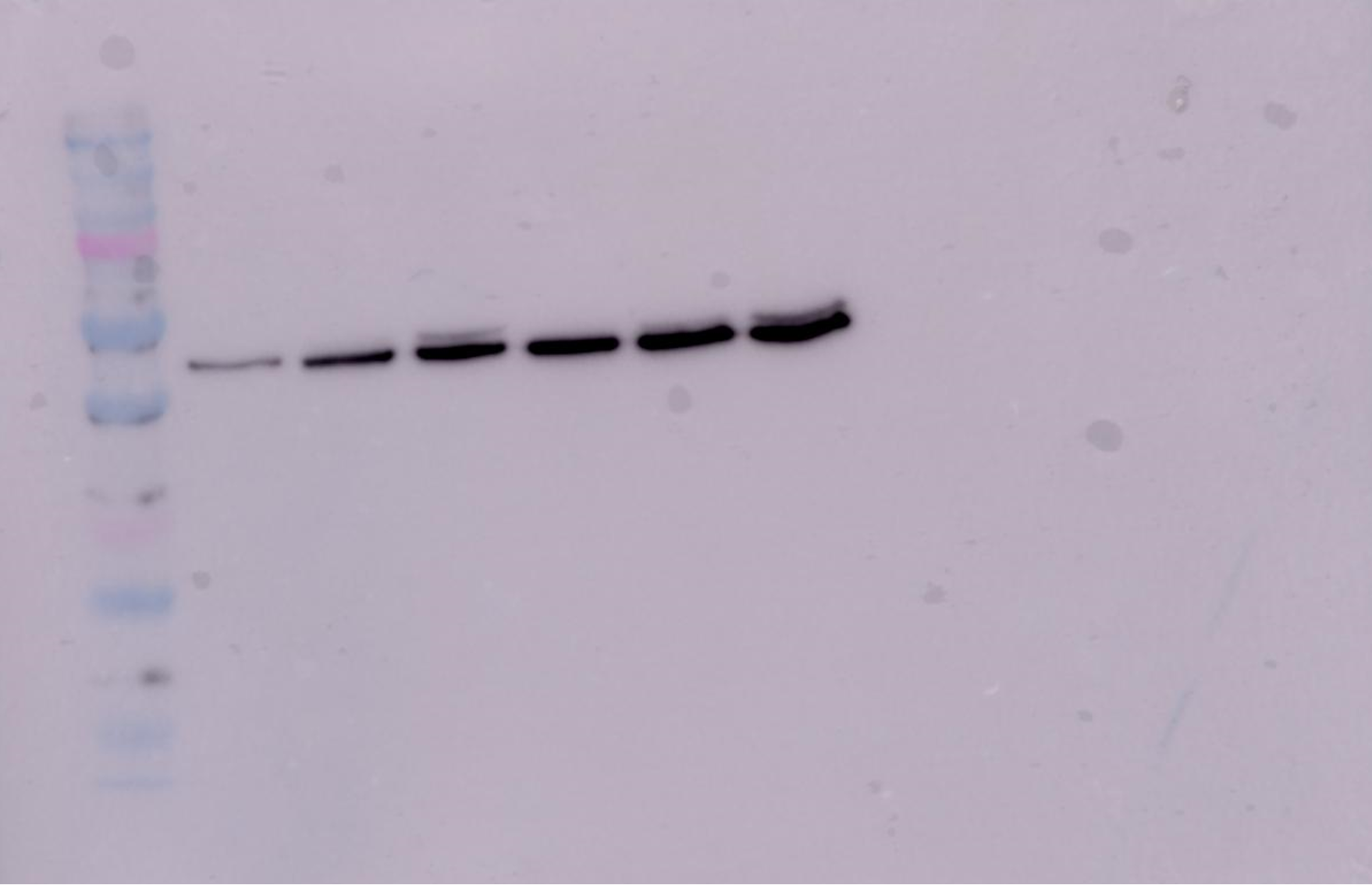

Supplement: Figure 3—source data 1. — 2xFLAG-Casp11 protein levels in each stable HEK293T cell line were determined by immunoblotting for FLAG. β-actin was used as loading control. [file elife-83725-fig3-data1.zip › Actin_blot.png]

3B.

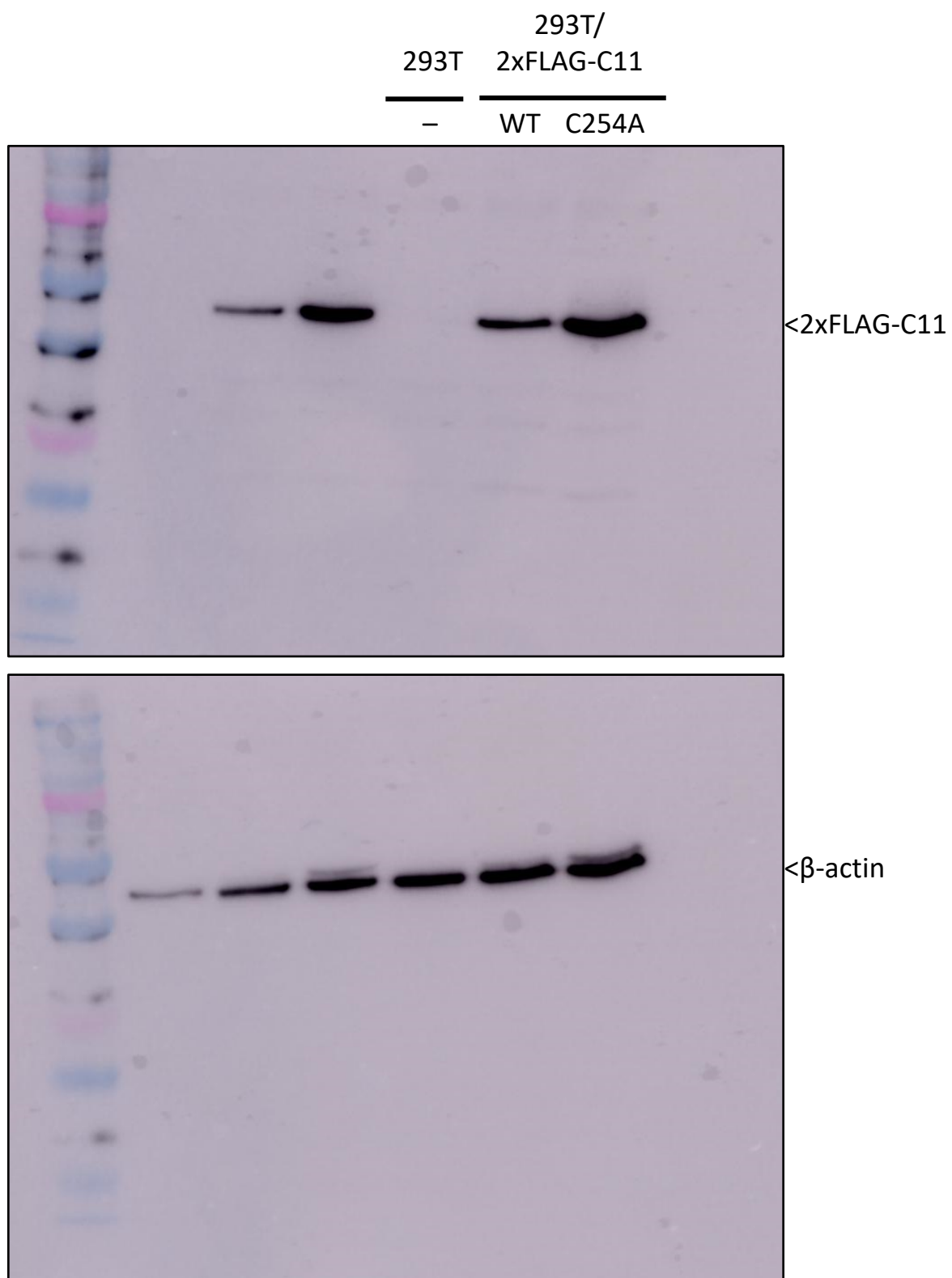

Supplement: Figure 3—source data 1. — 2xFLAG-Casp11 protein levels in each stable HEK293T cell line were determined by immunoblotting for FLAG. β-actin was used as loading control. [file elife-83725-fig3-data1.zip › Figure 3-source data 1.pdf]

## Slide 1
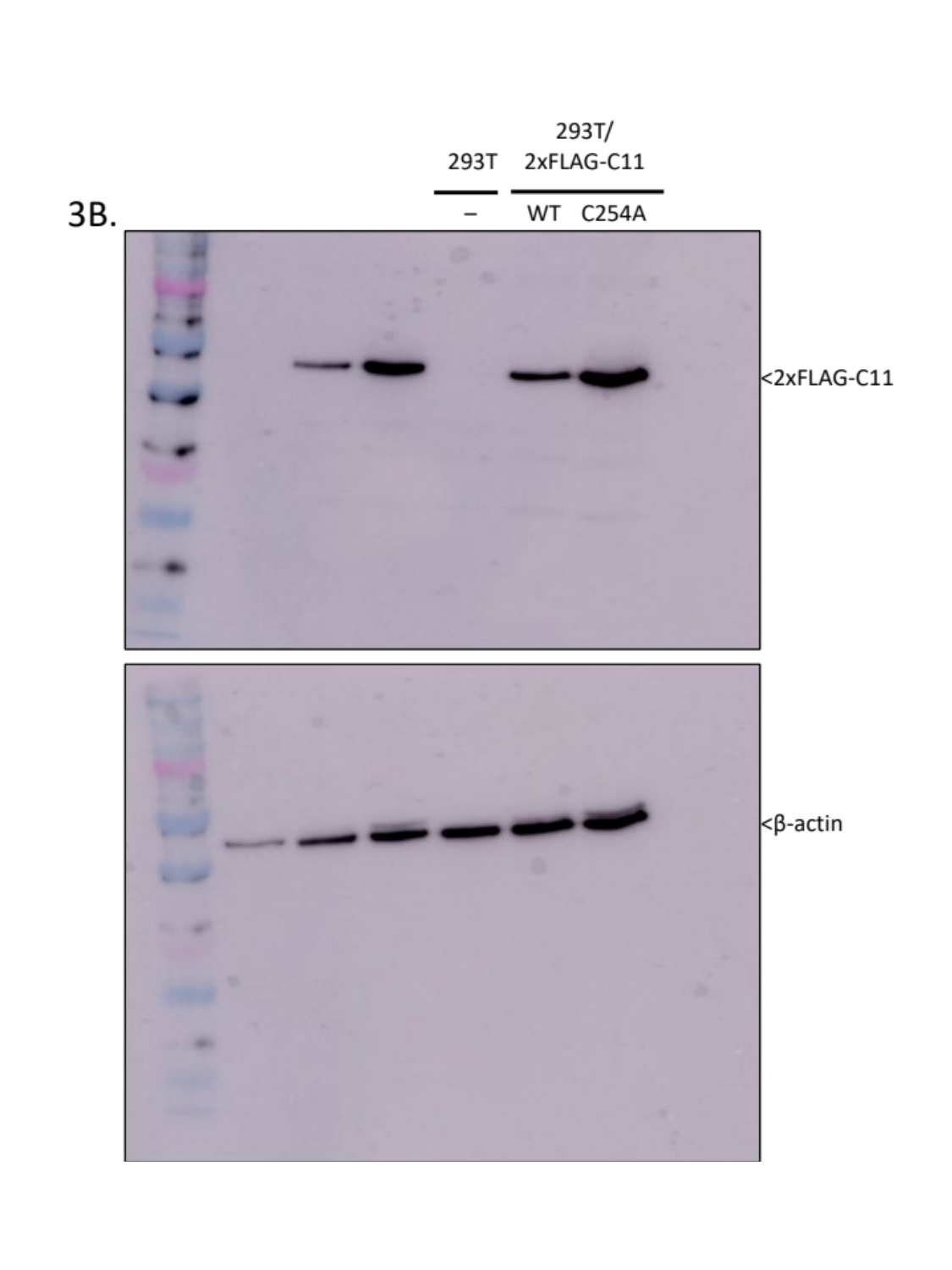

Supplement: Figure 3—source data 1. — 2xFLAG-Casp11 protein levels in each stable HEK293T cell line were determined by immunoblotting for FLAG. β-actin was used as loading control. [file elife-83725-fig3-data1.zip › Figure_3B_labeled.pptx]

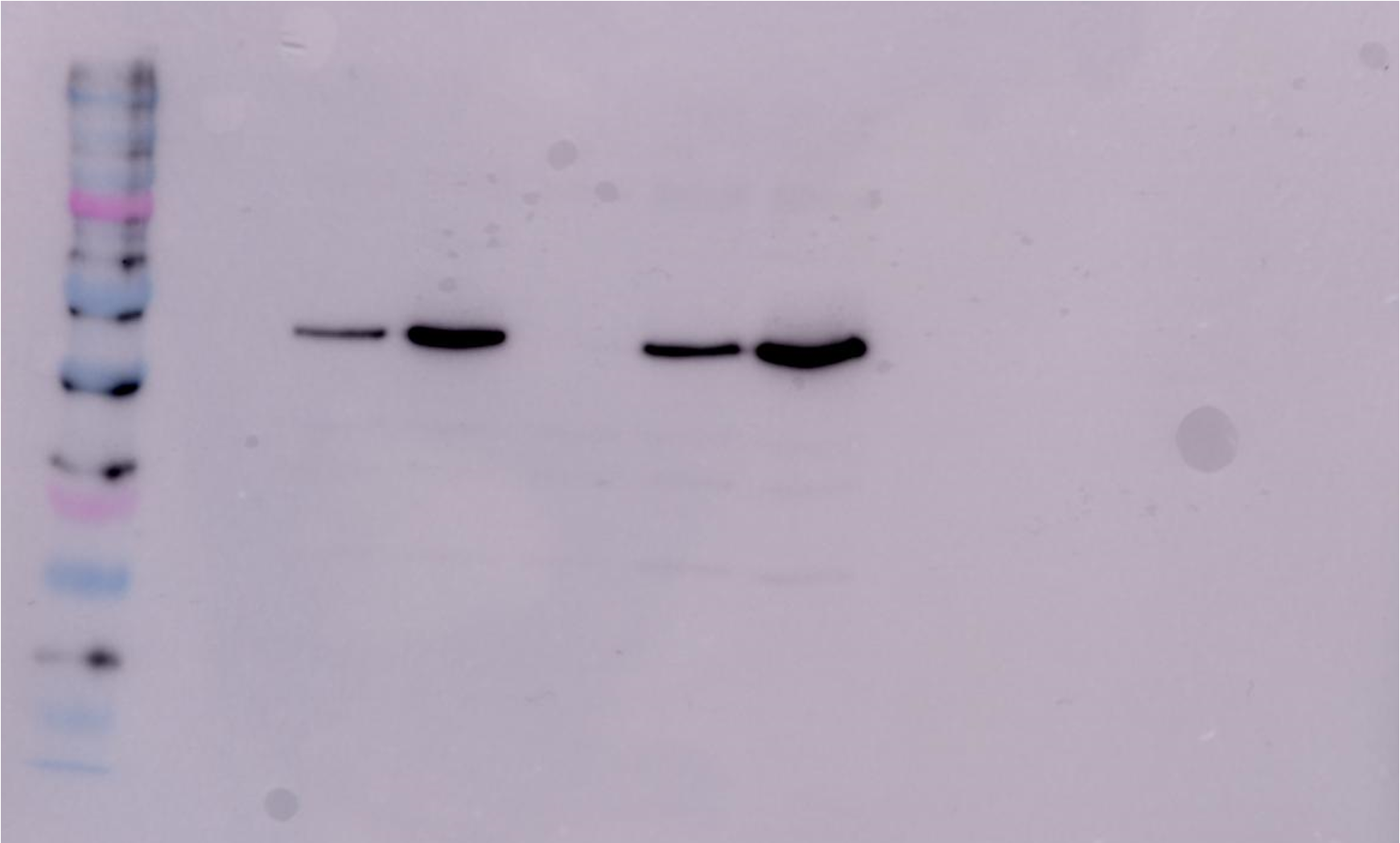

Supplement: Figure 3—source data 1. — 2xFLAG-Casp11 protein levels in each stable HEK293T cell line were determined by immunoblotting for FLAG. β-actin was used as loading control. [file elife-83725-fig3-data1.zip › FLAG_blot.png]

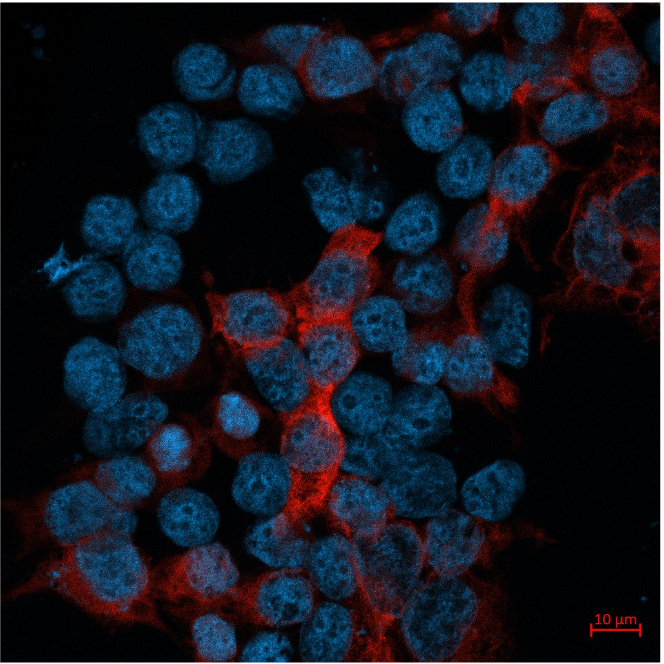

Supplement: Figure 3—figure supplement 1—source data 1. — HEK293T cells stably expressing wild-type (WT) or catalytically inactive (C254A) 2xFLAG-Casp11 were transiently transfected with mCherry-tagged WT or C254A Casp11 constructs for 24 hr. Stably expressed 2xFLAG-Casp11 was stained by immunofluorescence using anti-FLAG (yellow) and cells were imaged by confocal microscopy (×63 objective). Nuclei (blue) are stained with DAPI. [file elife-83725-fig3-figsupp1-data1.zip › 293T_C11(C254A)-mCh.png]

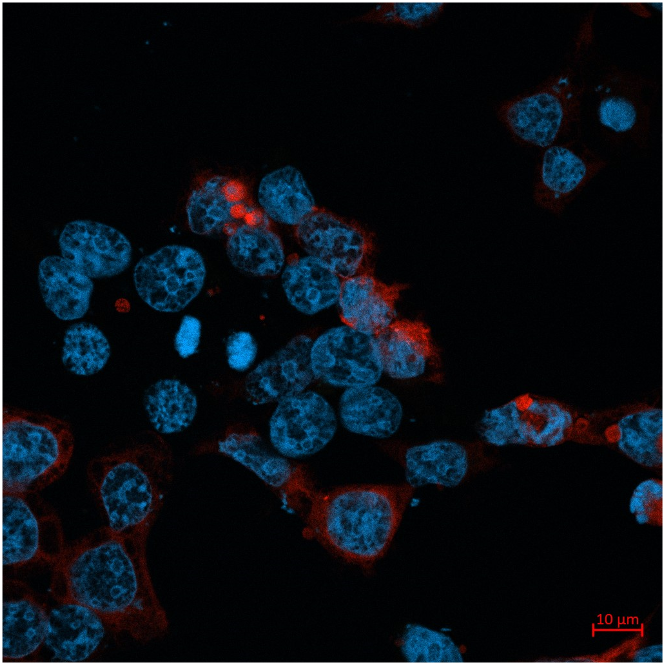

Supplement: Figure 3—figure supplement 1—source data 1. — HEK293T cells stably expressing wild-type (WT) or catalytically inactive (C254A) 2xFLAG-Casp11 were transiently transfected with mCherry-tagged WT or C254A Casp11 constructs for 24 hr. Stably expressed 2xFLAG-Casp11 was stained by immunofluorescence using anti-FLAG (yellow) and cells were imaged by confocal microscopy (×63 objective). Nuclei (blue) are stained with DAPI. [file elife-83725-fig3-figsupp1-data1.zip › 293T_C11(WT)-mCh.png]

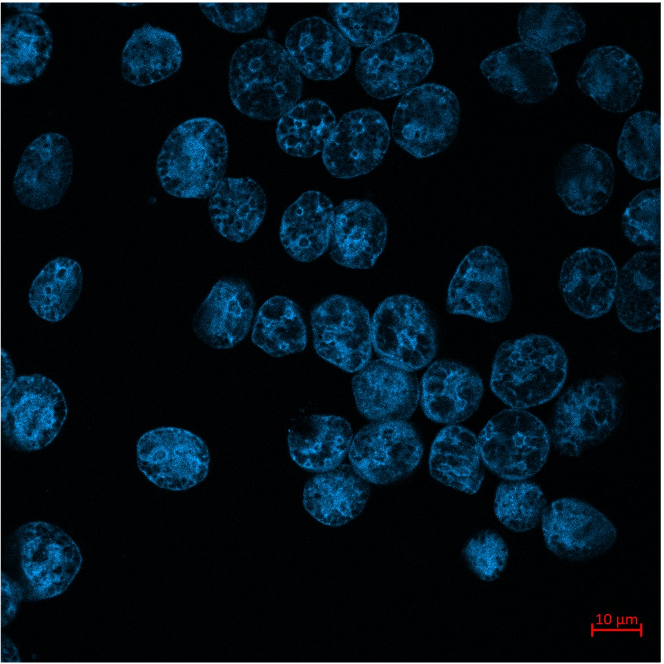

Supplement: Figure 3—figure supplement 1—source data 1. — HEK293T cells stably expressing wild-type (WT) or catalytically inactive (C254A) 2xFLAG-Casp11 were transiently transfected with mCherry-tagged WT or C254A Casp11 constructs for 24 hr. Stably expressed 2xFLAG-Casp11 was stained by immunofluorescence using anti-FLAG (yellow) and cells were imaged by confocal microscopy (×63 objective). Nuclei (blue) are stained with DAPI. [file elife-83725-fig3-figsupp1-data1.zip › 293T_Empty.png]

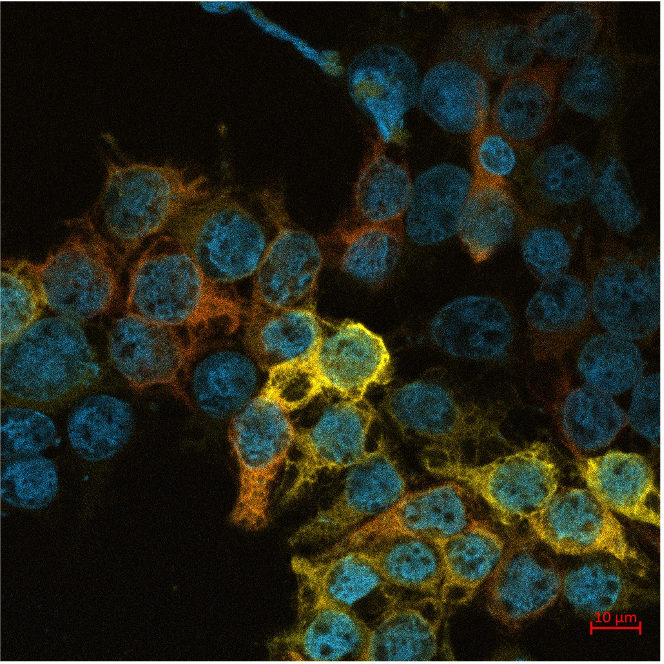

Supplement: Figure 3—figure supplement 1—source data 1. — HEK293T cells stably expressing wild-type (WT) or catalytically inactive (C254A) 2xFLAG-Casp11 were transiently transfected with mCherry-tagged WT or C254A Casp11 constructs for 24 hr. Stably expressed 2xFLAG-Casp11 was stained by immunofluorescence using anti-FLAG (yellow) and cells were imaged by confocal microscopy (×63 objective). Nuclei (blue) are stained with DAPI. [file elife-83725-fig3-figsupp1-data1.zip › 293T:2xFLAG-C11(C254A)_C11(C254A)-mCh.png]

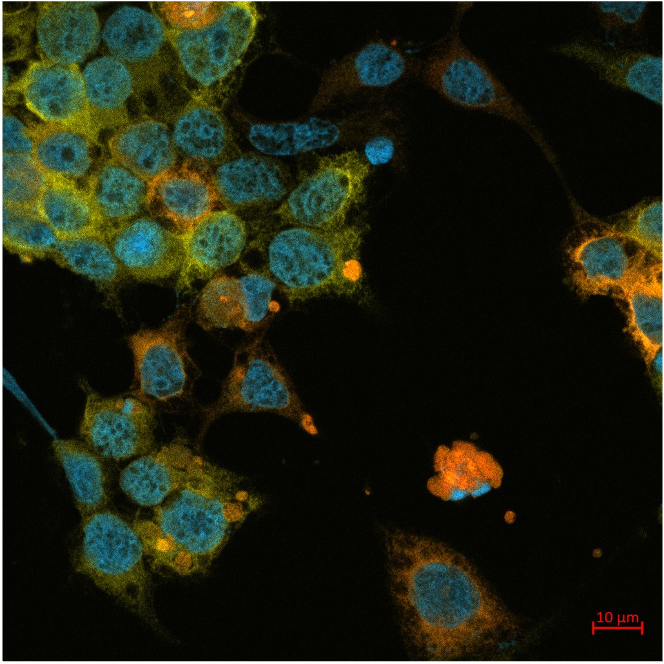

Supplement: Figure 3—figure supplement 1—source data 1. — HEK293T cells stably expressing wild-type (WT) or catalytically inactive (C254A) 2xFLAG-Casp11 were transiently transfected with mCherry-tagged WT or C254A Casp11 constructs for 24 hr. Stably expressed 2xFLAG-Casp11 was stained by immunofluorescence using anti-FLAG (yellow) and cells were imaged by confocal microscopy (×63 objective). Nuclei (blue) are stained with DAPI. [file elife-83725-fig3-figsupp1-data1.zip › 293T:2xFLAG-C11(C254A)_C11(WT)-mCh.png]

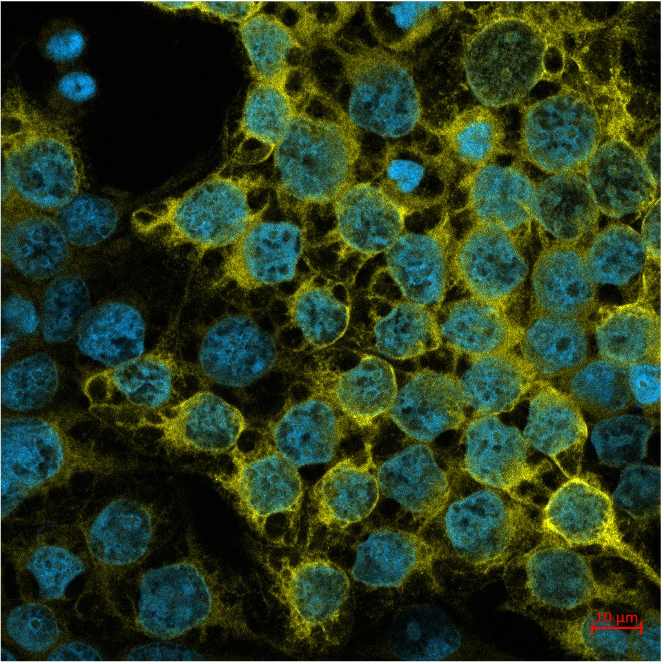

Supplement: Figure 3—figure supplement 1—source data 1. — HEK293T cells stably expressing wild-type (WT) or catalytically inactive (C254A) 2xFLAG-Casp11 were transiently transfected with mCherry-tagged WT or C254A Casp11 constructs for 24 hr. Stably expressed 2xFLAG-Casp11 was stained by immunofluorescence using anti-FLAG (yellow) and cells were imaged by confocal microscopy (×63 objective). Nuclei (blue) are stained with DAPI. [file elife-83725-fig3-figsupp1-data1.zip › 293T:2xFLAG-C11(C254A)_Empty.png]

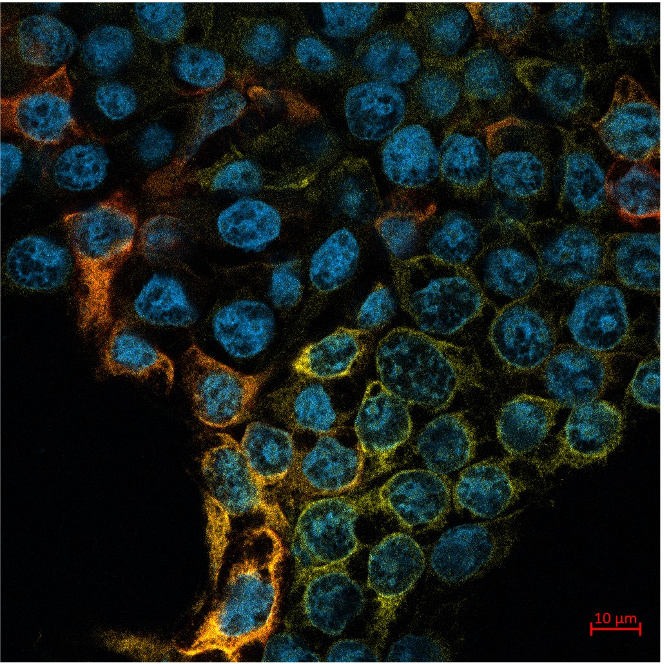

Supplement: Figure 3—figure supplement 1—source data 1. — HEK293T cells stably expressing wild-type (WT) or catalytically inactive (C254A) 2xFLAG-Casp11 were transiently transfected with mCherry-tagged WT or C254A Casp11 constructs for 24 hr. Stably expressed 2xFLAG-Casp11 was stained by immunofluorescence using anti-FLAG (yellow) and cells were imaged by confocal microscopy (×63 objective). Nuclei (blue) are stained with DAPI. [file elife-83725-fig3-figsupp1-data1.zip › 293T:2xFLAG-C11(WT)_C11(C254A)-mCh.png]

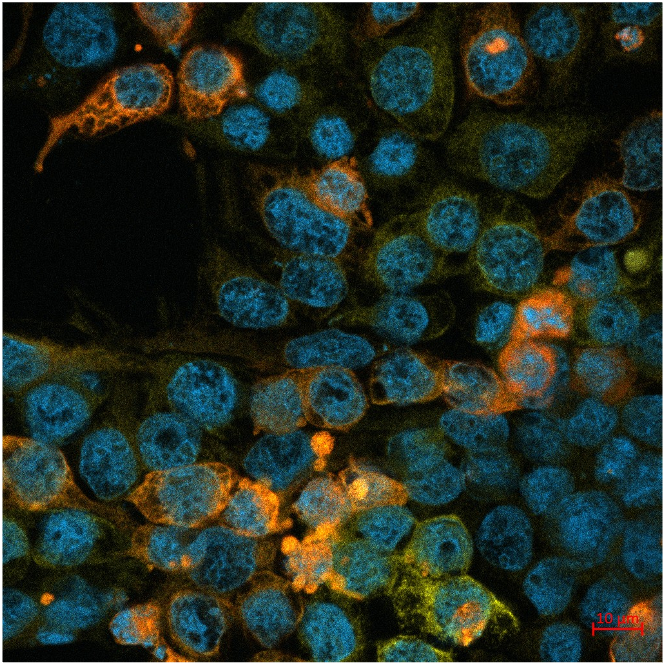

Supplement: Figure 3—figure supplement 1—source data 1. — HEK293T cells stably expressing wild-type (WT) or catalytically inactive (C254A) 2xFLAG-Casp11 were transiently transfected with mCherry-tagged WT or C254A Casp11 constructs for 24 hr. Stably expressed 2xFLAG-Casp11 was stained by immunofluorescence using anti-FLAG (yellow) and cells were imaged by confocal microscopy (×63 objective). Nuclei (blue) are stained with DAPI. [file elife-83725-fig3-figsupp1-data1.zip › 293T:2xFLAG-C11(WT)_C11(WT)-mCh.png]

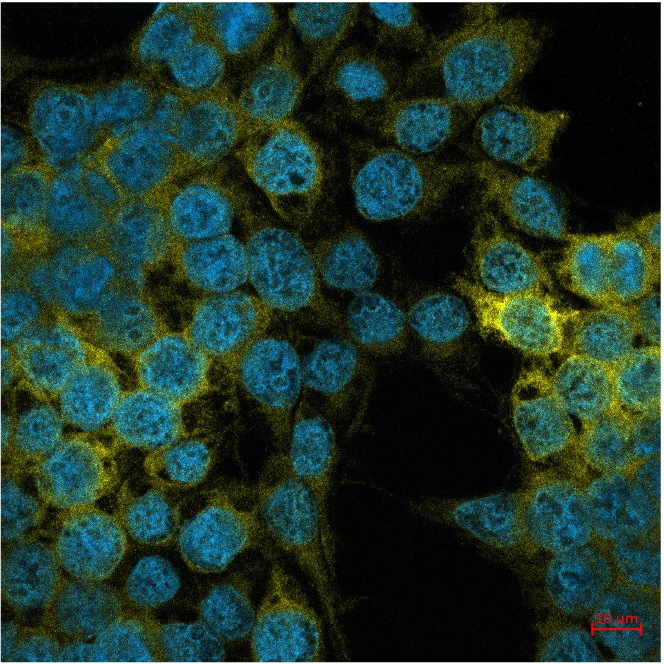

Supplement: Figure 3—figure supplement 1—source data 1. — HEK293T cells stably expressing wild-type (WT) or catalytically inactive (C254A) 2xFLAG-Casp11 were transiently transfected with mCherry-tagged WT or C254A Casp11 constructs for 24 hr. Stably expressed 2xFLAG-Casp11 was stained by immunofluorescence using anti-FLAG (yellow) and cells were imaged by confocal microscopy (×63 objective). Nuclei (blue) are stained with DAPI. [file elife-83725-fig3-figsupp1-data1.zip › 293T:2xFLAG-C11(WT)_Empty.png]

Fig 3-suppl 1-source data 1 (3S1B)

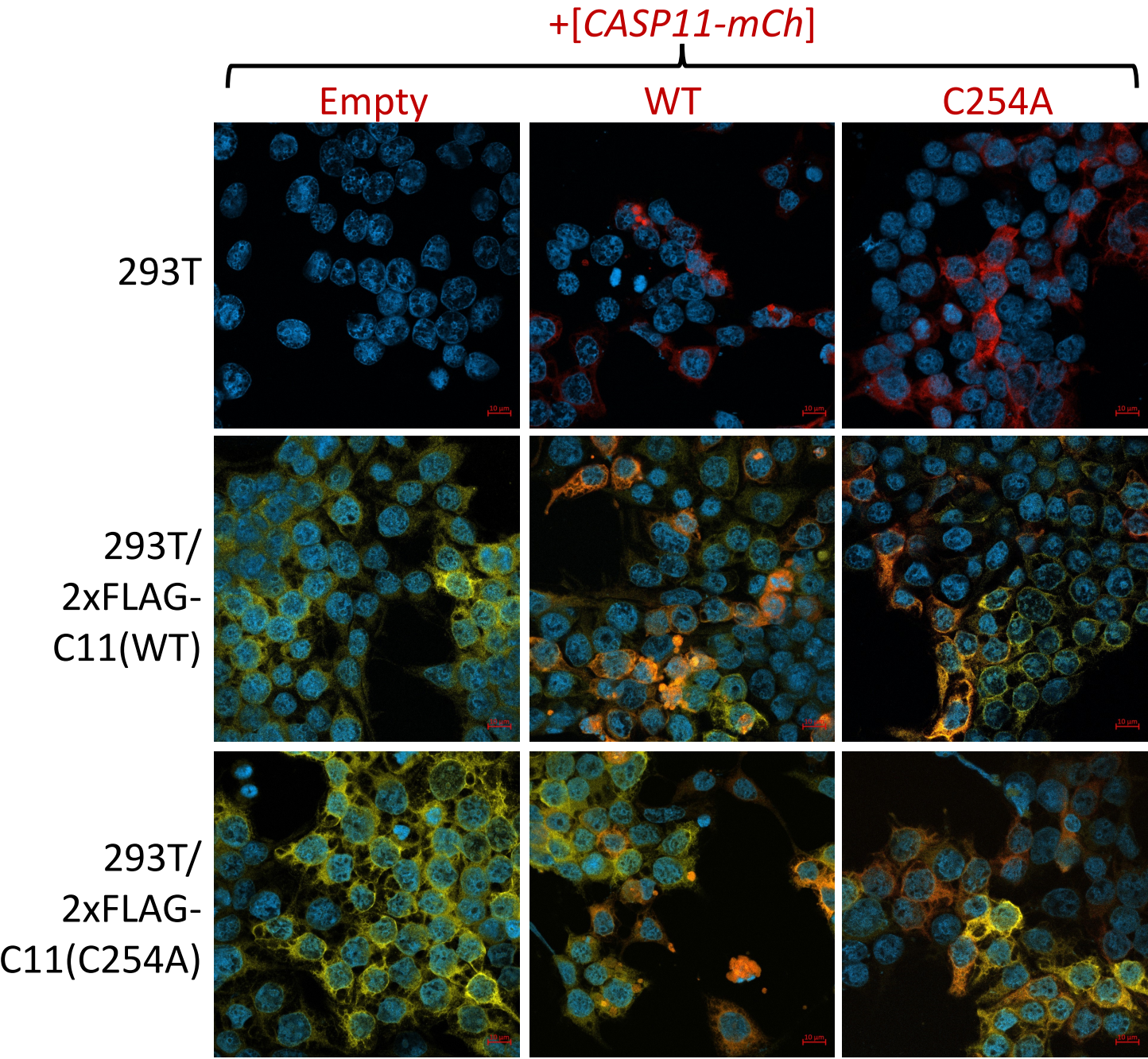

DAPI mCHERRY FLAG

Supplement: Figure 3—figure supplement 1—source data 1. — HEK293T cells stably expressing wild-type (WT) or catalytically inactive (C254A) 2xFLAG-Casp11 were transiently transfected with mCherry-tagged WT or C254A Casp11 constructs for 24 hr. Stably expressed 2xFLAG-Casp11 was stained by immunofluorescence using anti-FLAG (yellow) and cells were imaged by confocal microscopy (×63 objective). Nuclei (blue) are stained with DAPI. [file elife-83725-fig3-figsupp1-data1.zip › Figure 3-figure supplement 1-source data 1.pdf]

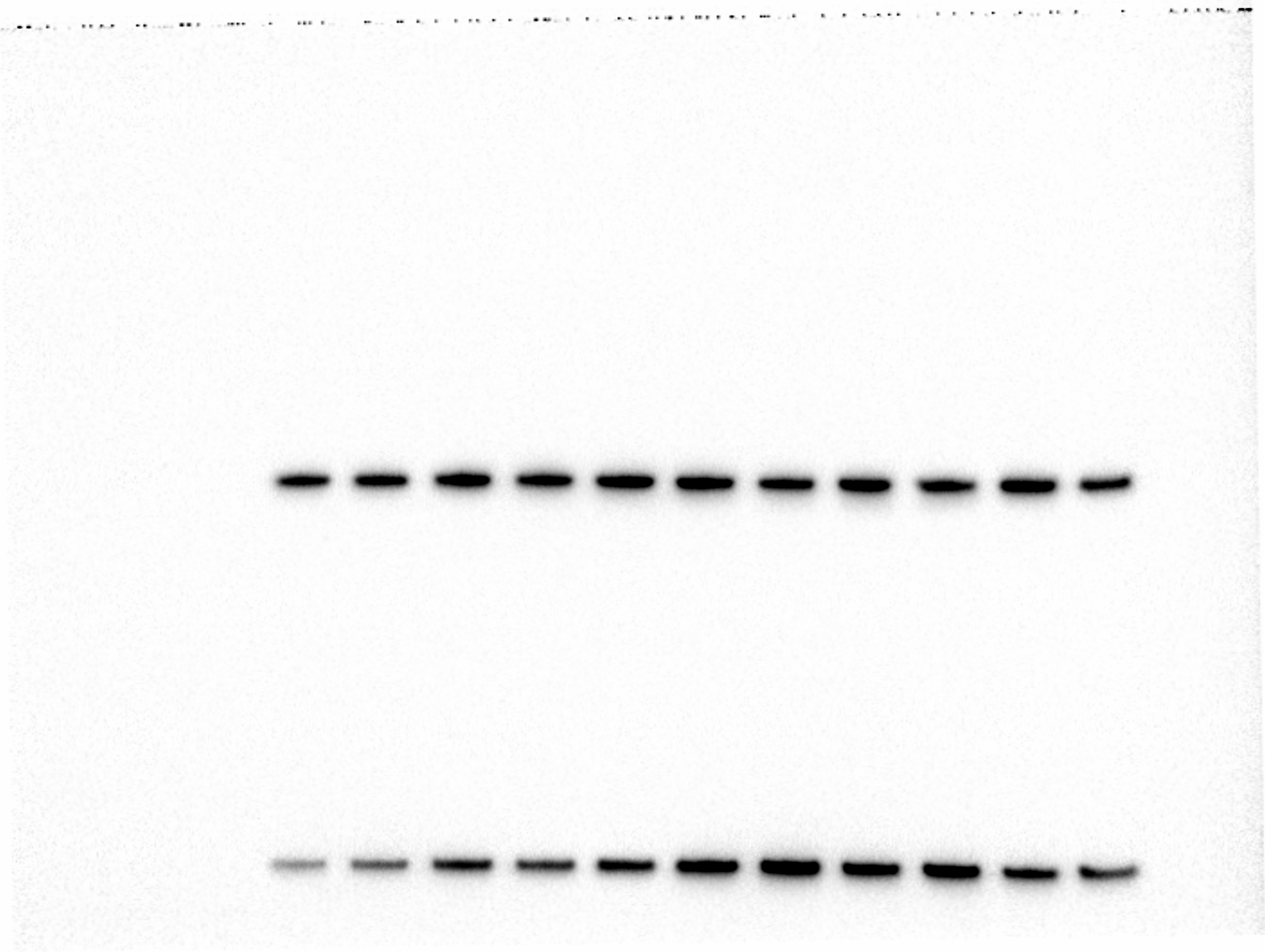

Supplement: Figure 4—source data 1. — 12 hr post-transfection of indicated plasmids, whole-cell lysates were harvested and immunoblotted for mCherry or β-actin as a loading control. [file elife-83725-fig4-data1.zip › Actin.tif]

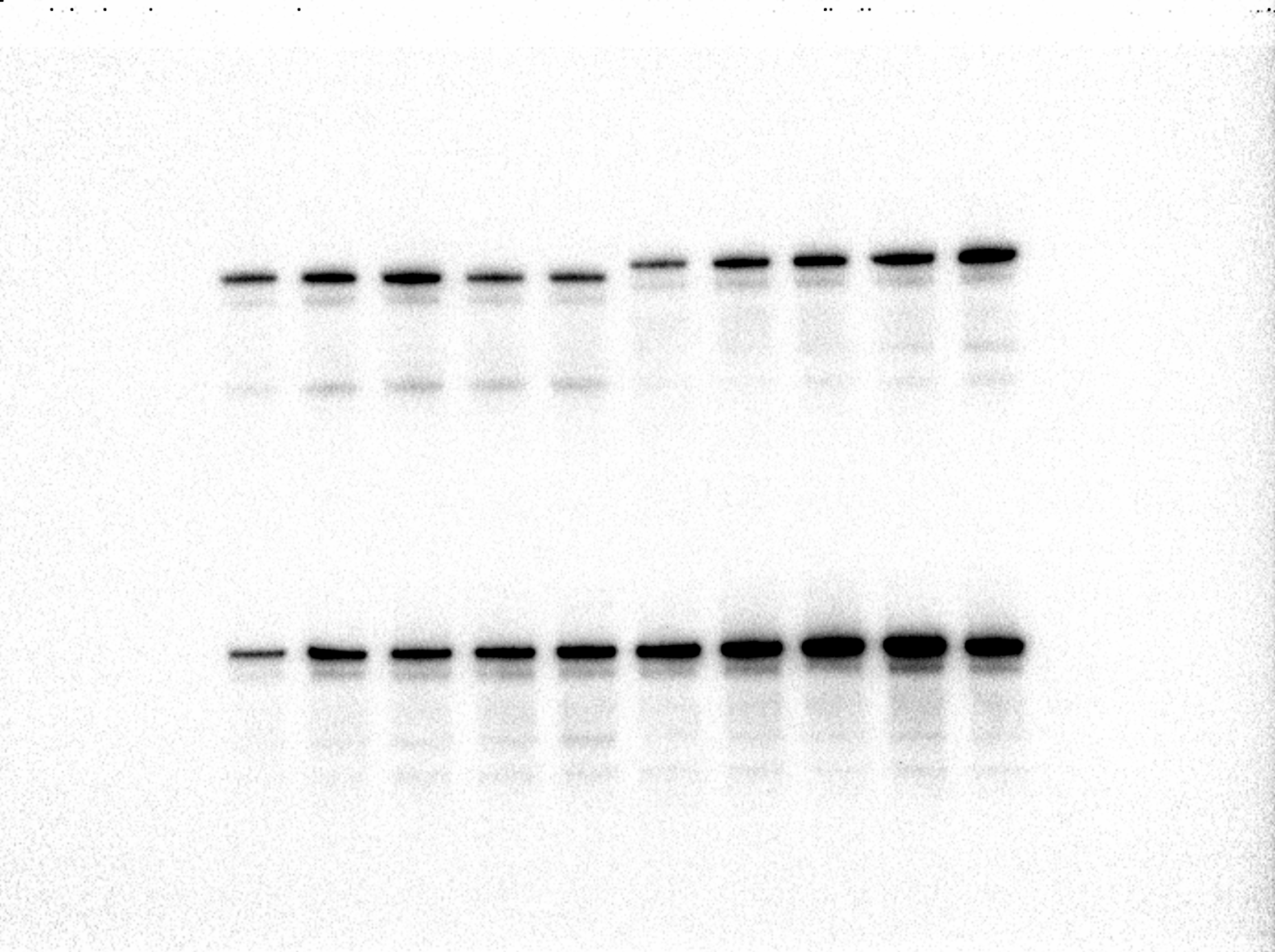

Supplement: Figure 4—source data 1. — 12 hr post-transfection of indicated plasmids, whole-cell lysates were harvested and immunoblotted for mCherry or β-actin as a loading control. [file elife-83725-fig4-data1.zip › Casp11-mCh.tif]

4B.

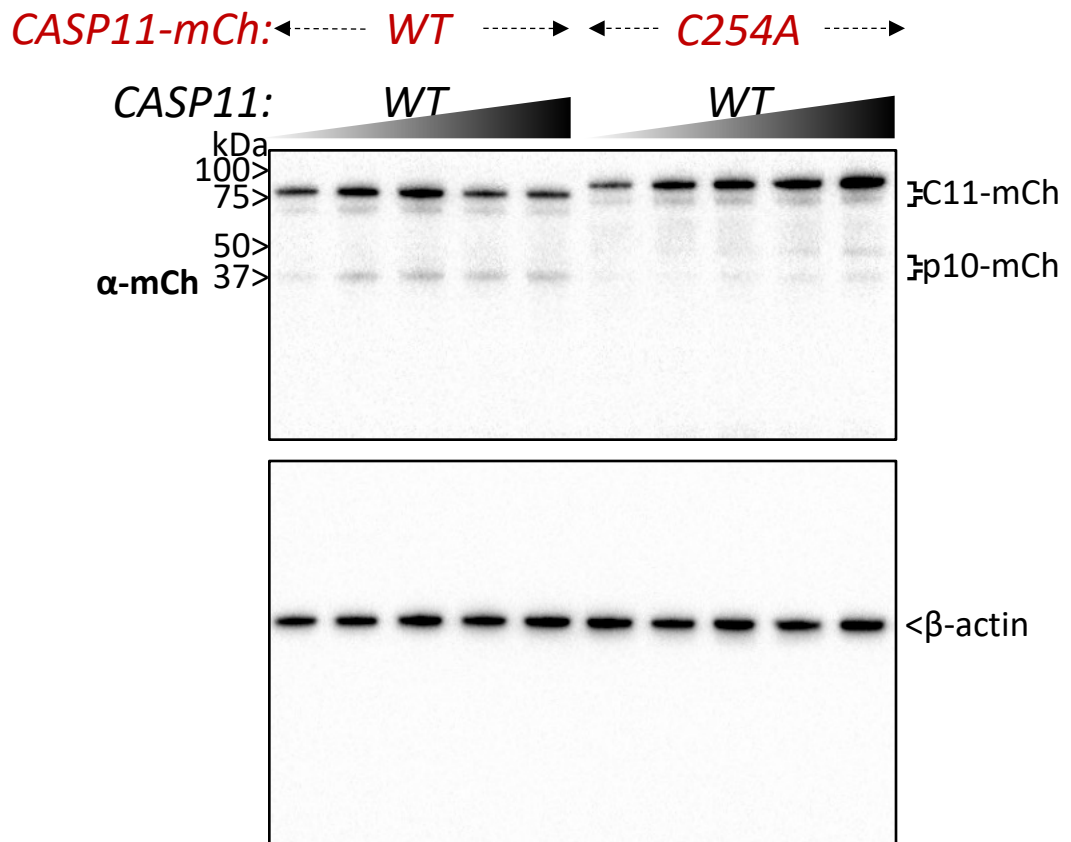

Supplement: Figure 4—source data 1. — 12 hr post-transfection of indicated plasmids, whole-cell lysates were harvested and immunoblotted for mCherry or β-actin as a loading control. [file elife-83725-fig4-data1.zip › Figure 4-source data 1.pdf]

## Slide 1
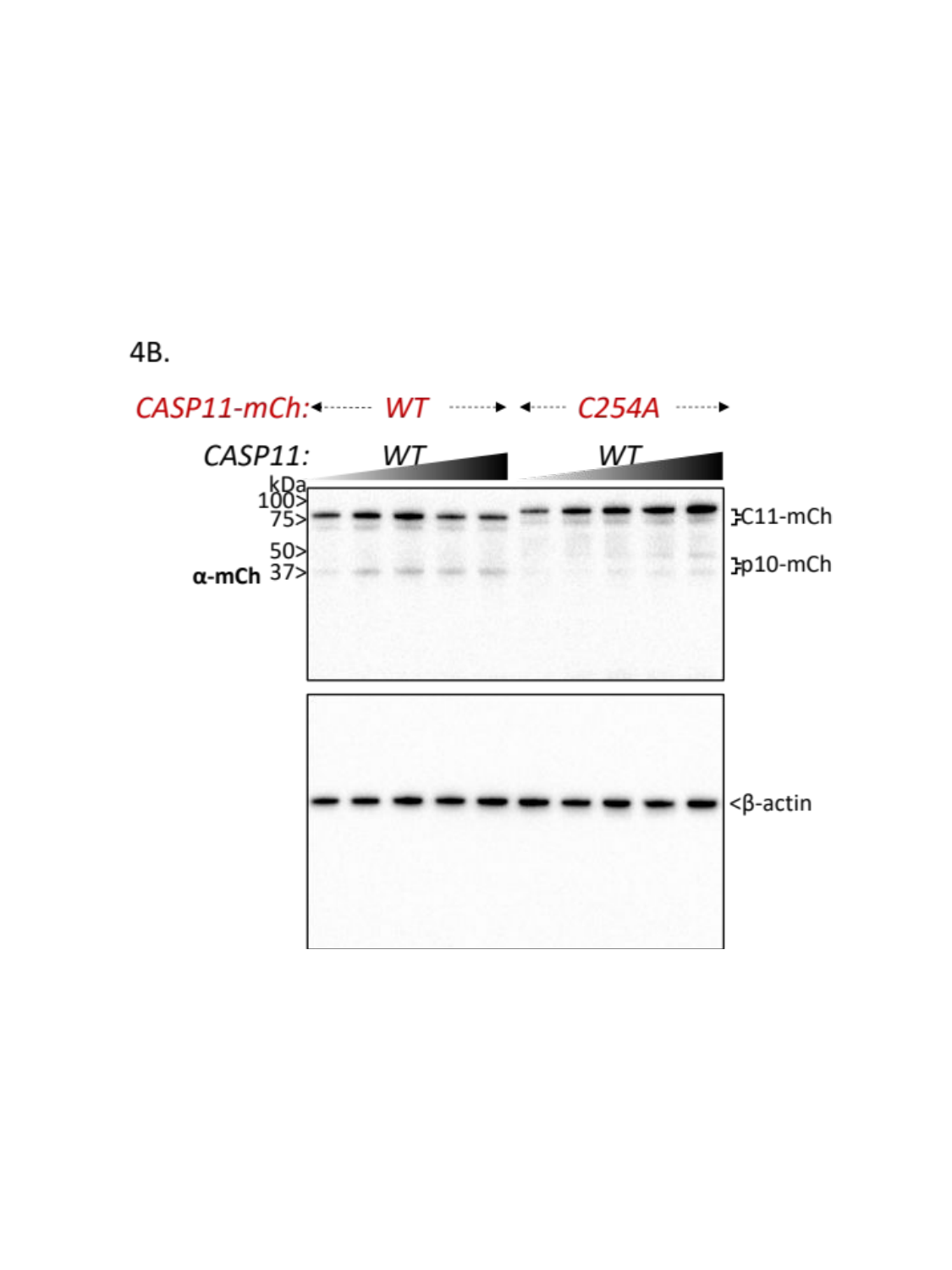

Supplement: Figure 4—source data 1. — 12 hr post-transfection of indicated plasmids, whole-cell lysates were harvested and immunoblotted for mCherry or β-actin as a loading control. [file elife-83725-fig4-data1.zip › Figure_4B_labeled.pptx]

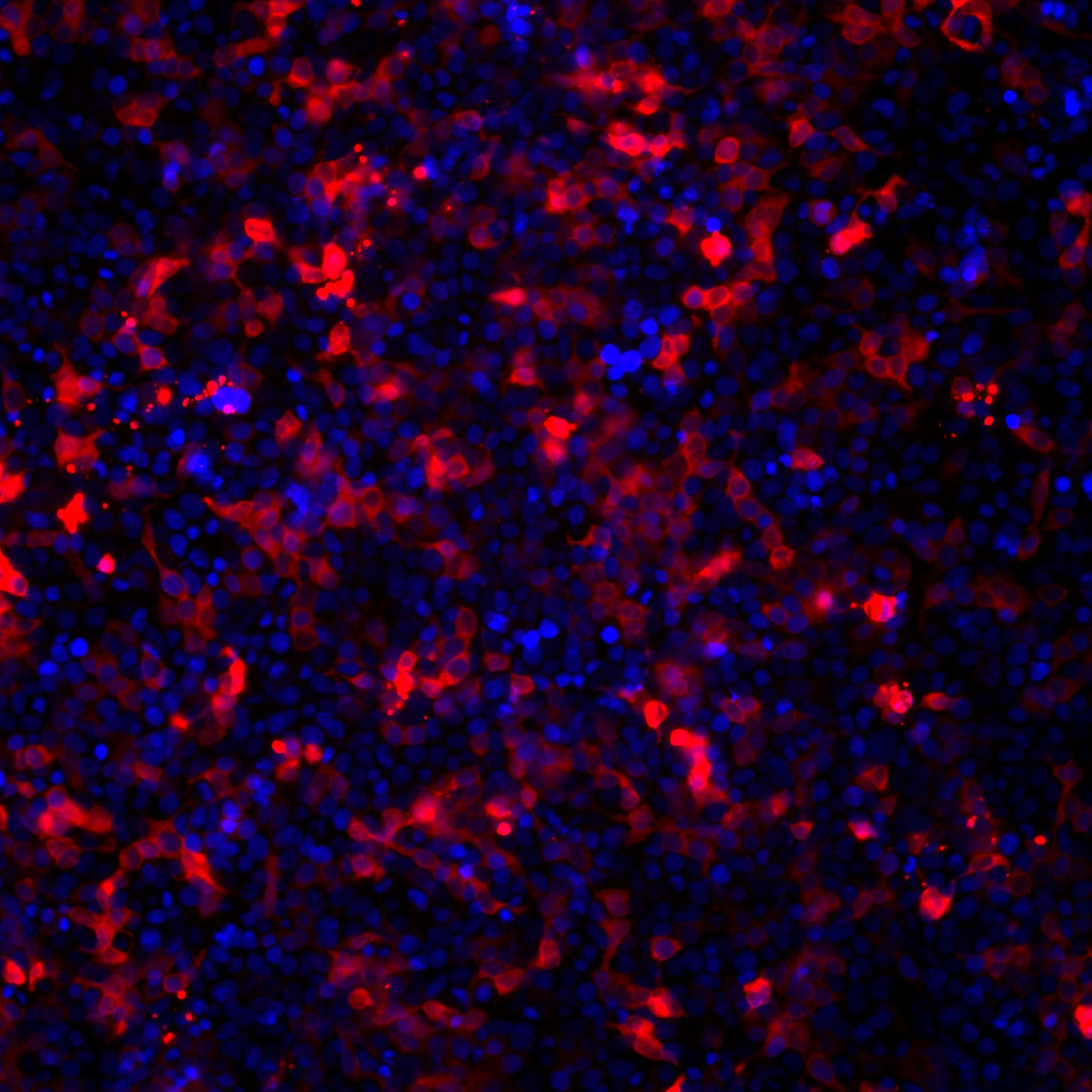

Supplement: Figure 4—source data 2. — Untagged full-length wild-type (WT) caspase-11 gene constructs were transfected at increasing doses, together with a fixed amount of indicated mCherry-tagged Casp11. 18 hr following transfection, the cells were imaged by fluorescence microscopy. Nuclei (blue) were stained with Hoechst. [file elife-83725-fig4-data2.zip › 0_C11(WT)_with_C11(WT)-mCh.tif]

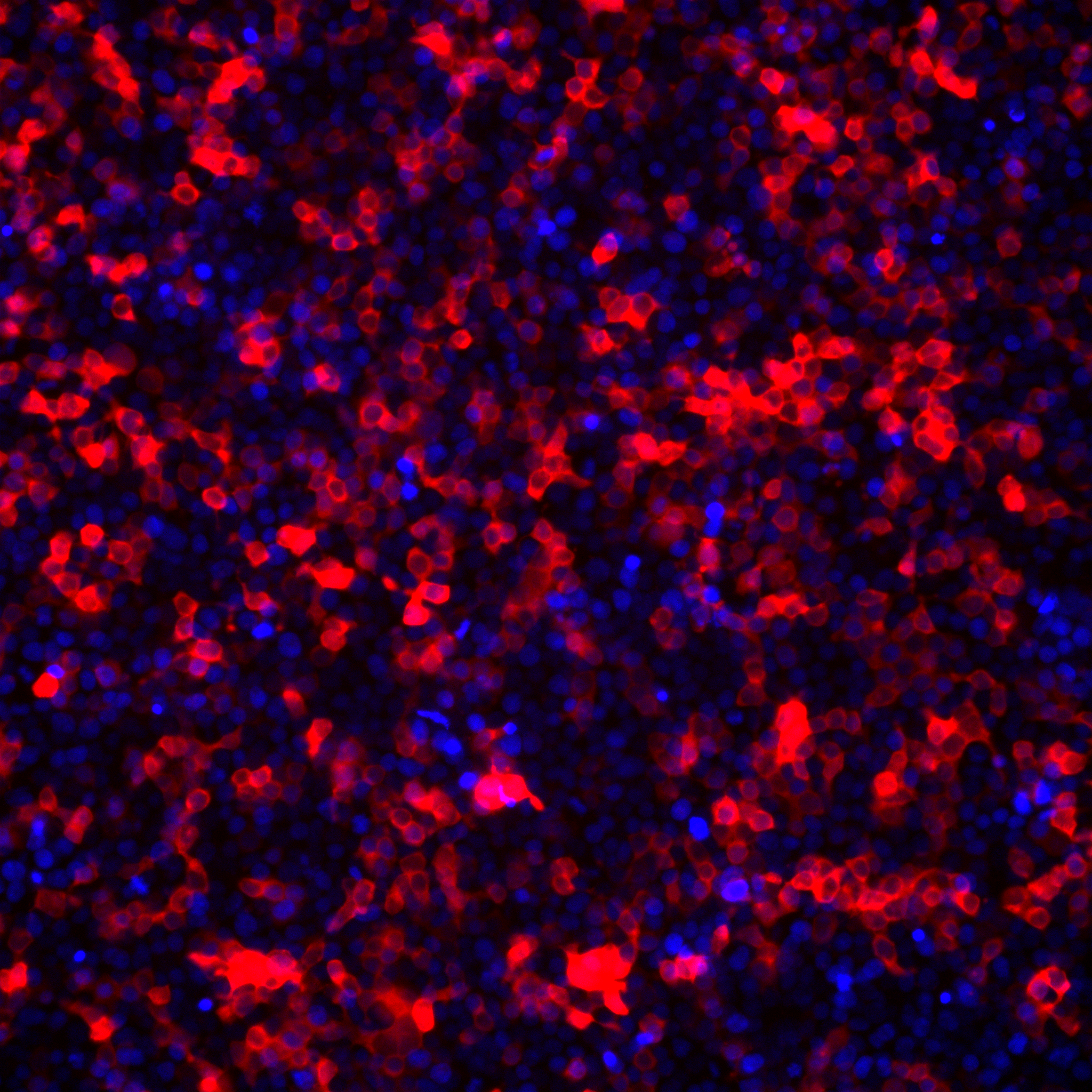

Supplement: Figure 4—source data 2. — Untagged full-length wild-type (WT) caspase-11 gene constructs were transfected at increasing doses, together with a fixed amount of indicated mCherry-tagged Casp11. 18 hr following transfection, the cells were imaged by fluorescence microscopy. Nuclei (blue) were stained with Hoechst. [file elife-83725-fig4-data2.zip › 0_C11(WT)_with_C254A-mCh.tif]

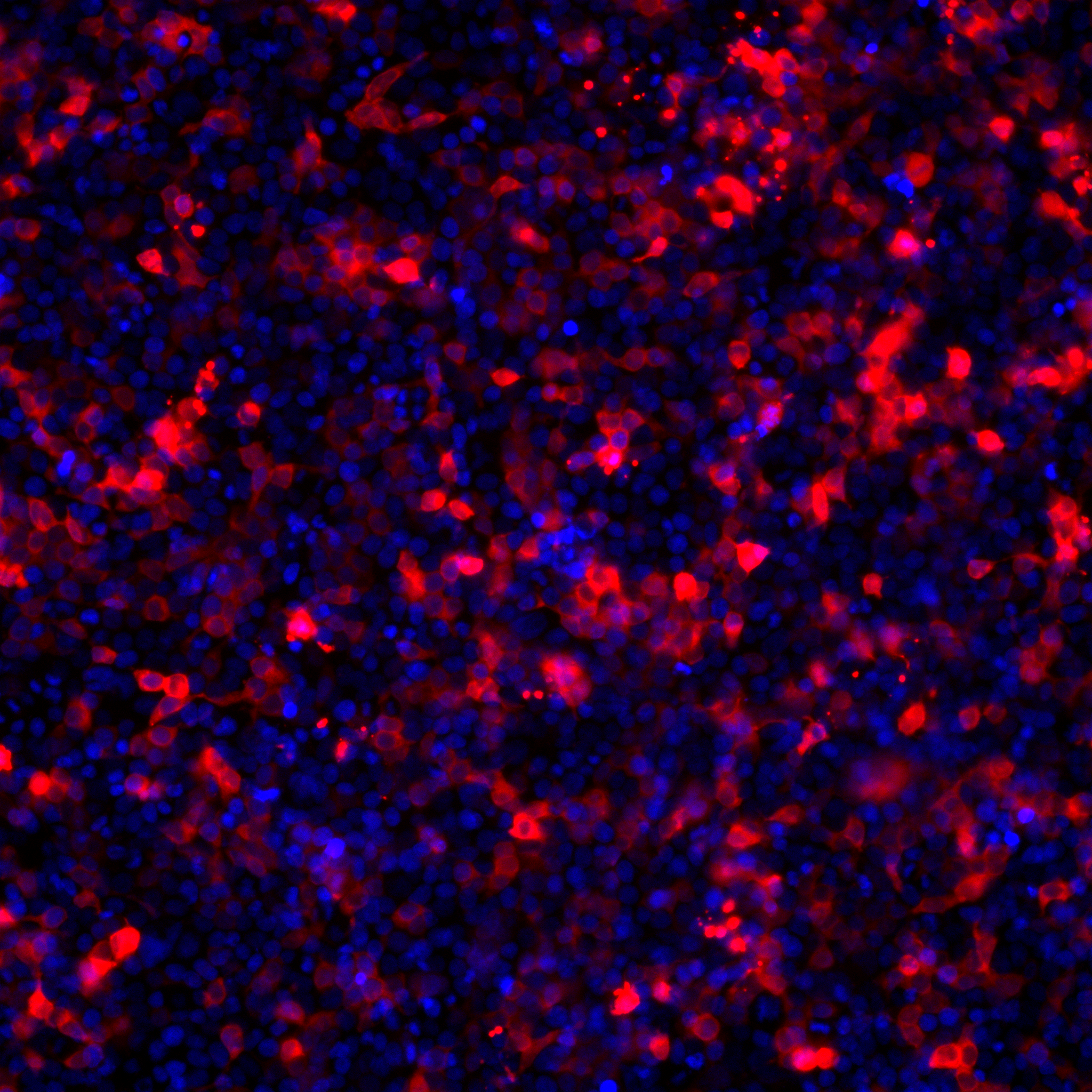

Supplement: Figure 4—source data 2. — Untagged full-length wild-type (WT) caspase-11 gene constructs were transfected at increasing doses, together with a fixed amount of indicated mCherry-tagged Casp11. 18 hr following transfection, the cells were imaged by fluorescence microscopy. Nuclei (blue) were stained with Hoechst. [file elife-83725-fig4-data2.zip › 125_C11(WT)_with_C11(WT)-mCh.tif]

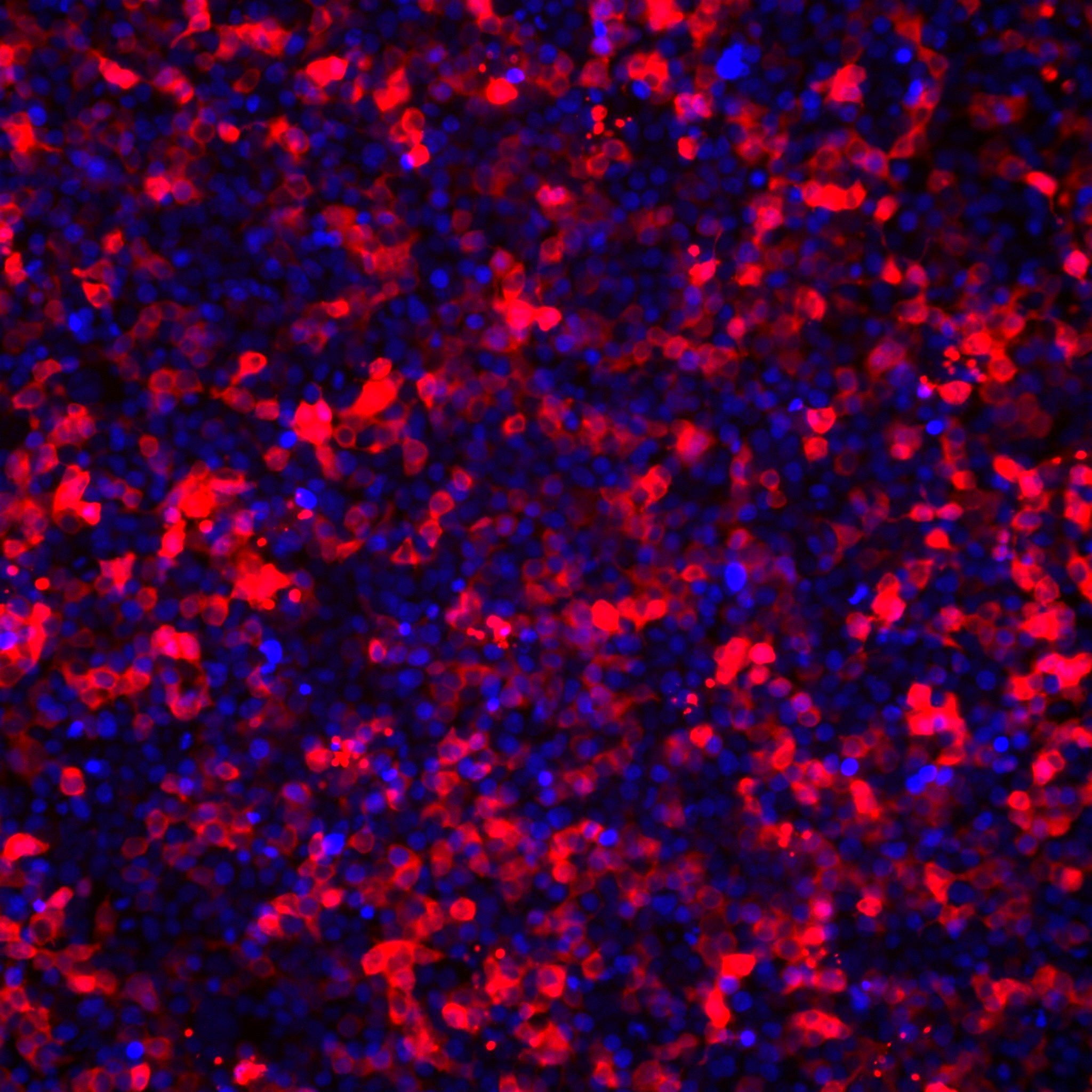

Supplement: Figure 4—source data 2. — Untagged full-length wild-type (WT) caspase-11 gene constructs were transfected at increasing doses, together with a fixed amount of indicated mCherry-tagged Casp11. 18 hr following transfection, the cells were imaged by fluorescence microscopy. Nuclei (blue) were stained with Hoechst. [file elife-83725-fig4-data2.zip › 125_C11(WT)_with_C254A-mCh.tif]

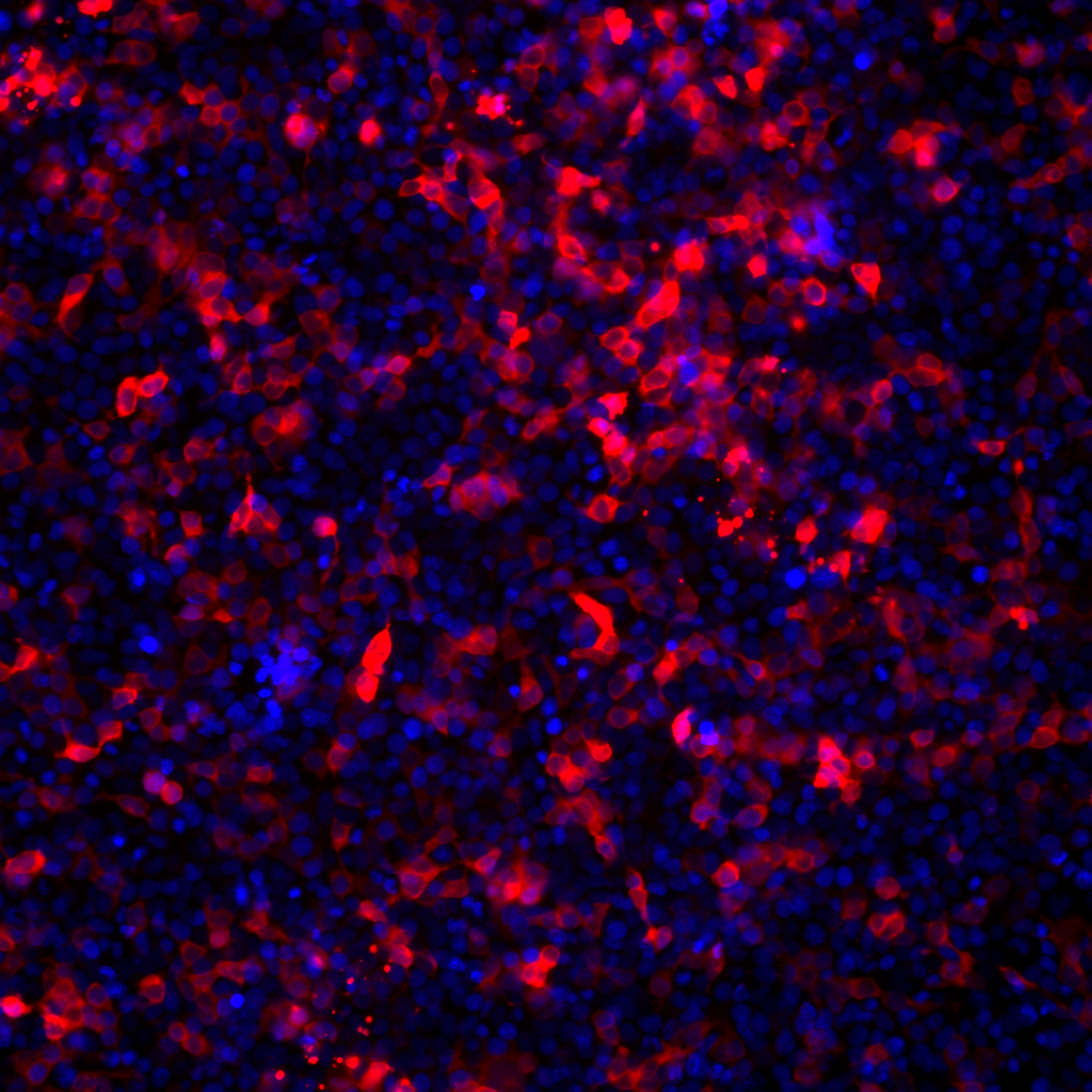

Supplement: Figure 4—source data 2. — Untagged full-length wild-type (WT) caspase-11 gene constructs were transfected at increasing doses, together with a fixed amount of indicated mCherry-tagged Casp11. 18 hr following transfection, the cells were imaged by fluorescence microscopy. Nuclei (blue) were stained with Hoechst. [file elife-83725-fig4-data2.zip › 500_C11(WT)_with_C11(WT)-mCh.tif]

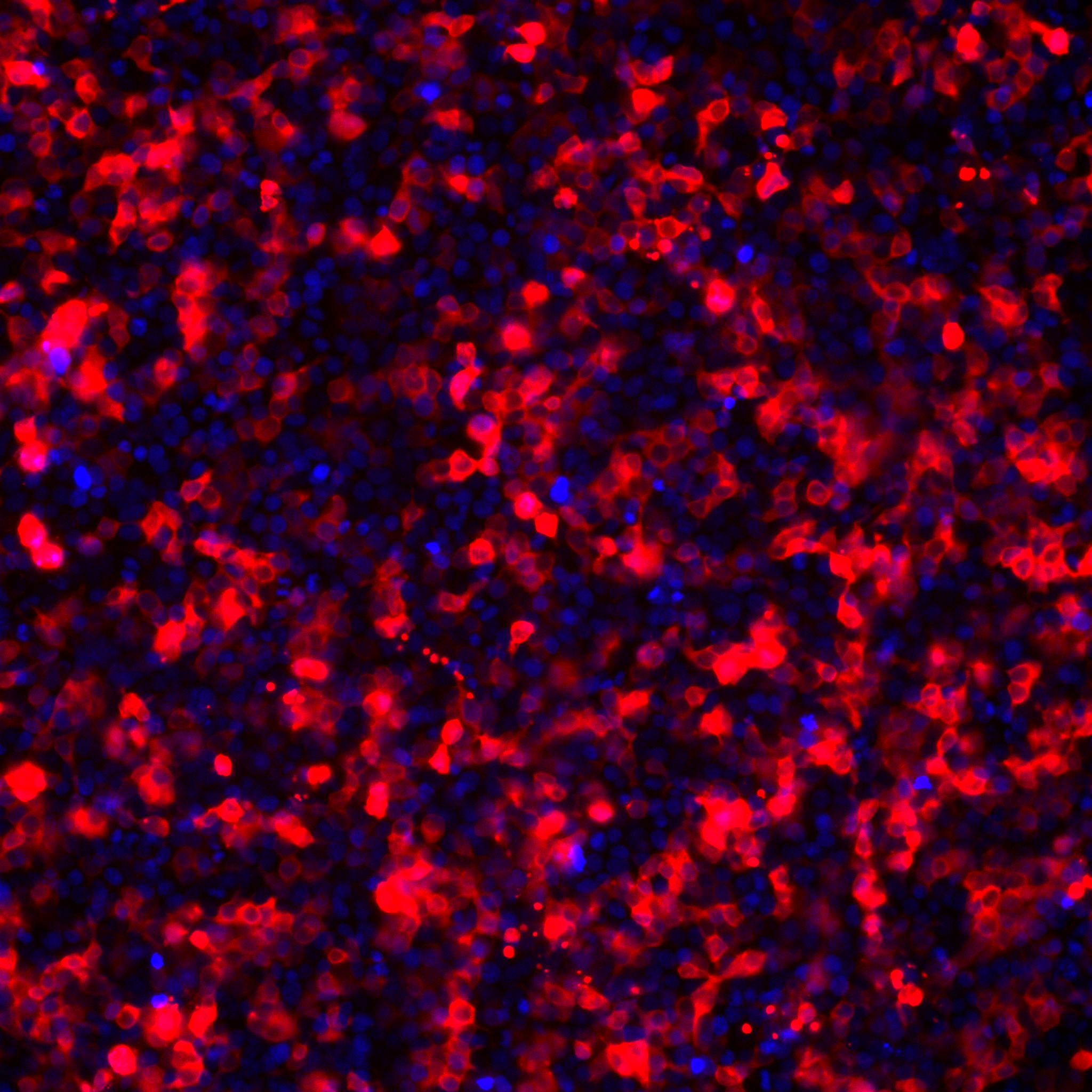

Supplement: Figure 4—source data 2. — Untagged full-length wild-type (WT) caspase-11 gene constructs were transfected at increasing doses, together with a fixed amount of indicated mCherry-tagged Casp11. 18 hr following transfection, the cells were imaged by fluorescence microscopy. Nuclei (blue) were stained with Hoechst. [file elife-83725-fig4-data2.zip › 500_C11(WT)_with_C254A-mCh.tif]

Fig 4-Source Data 2 (4C)

*WT CASP11:*

0 ng

125 ng

500 ng

*WT*

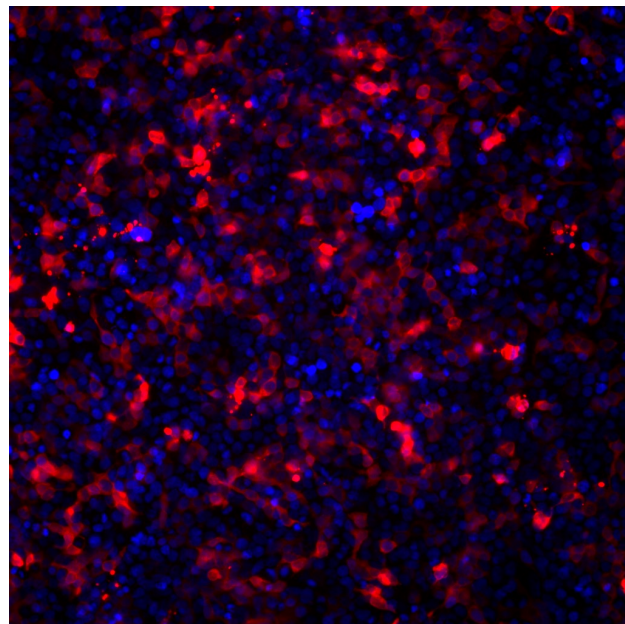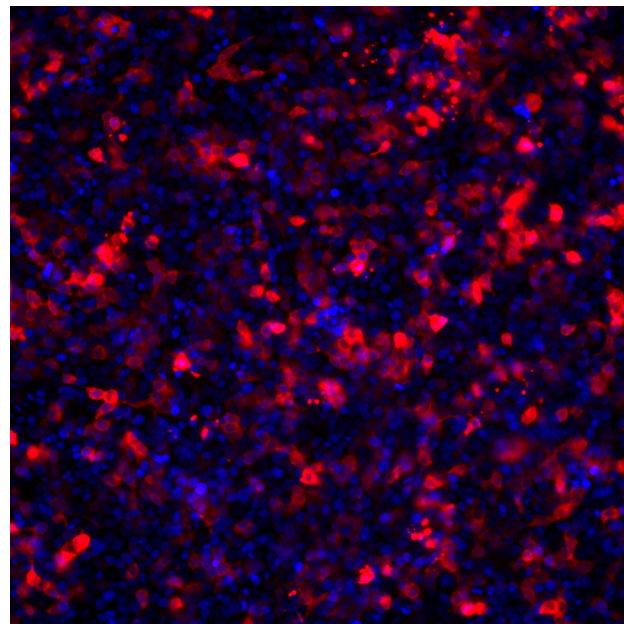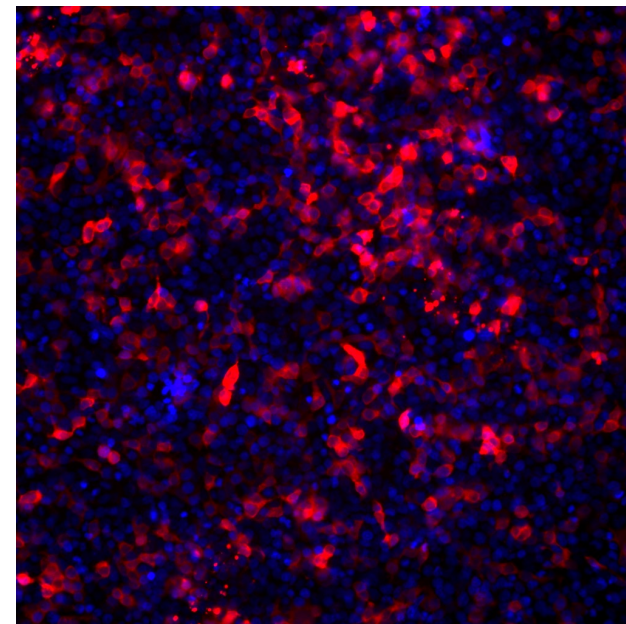

*CASP11-mCh:*

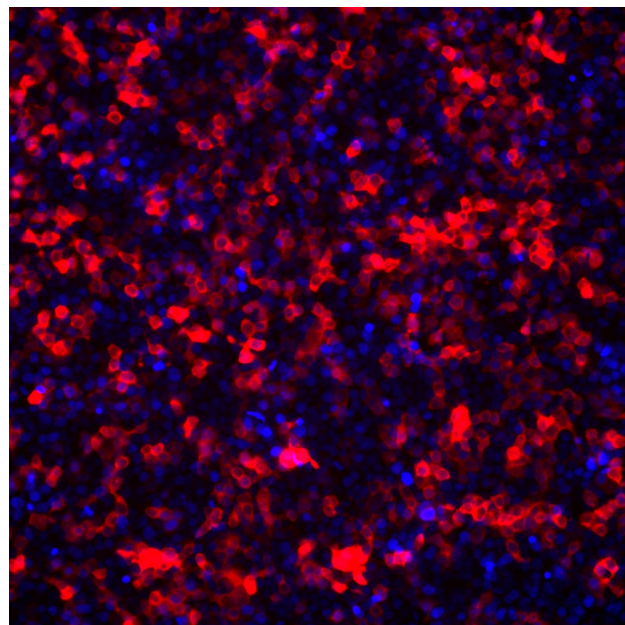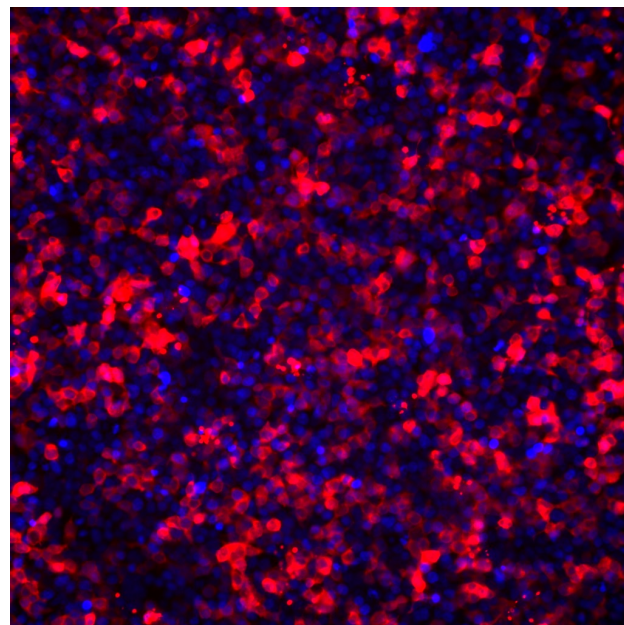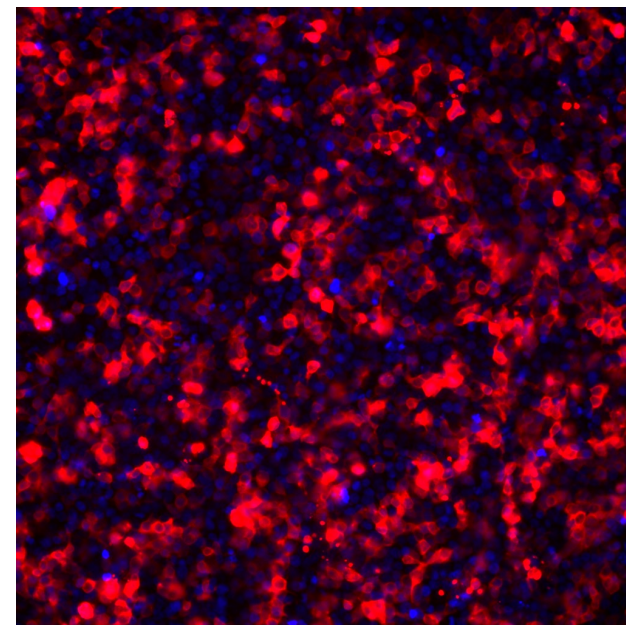

*C254A*

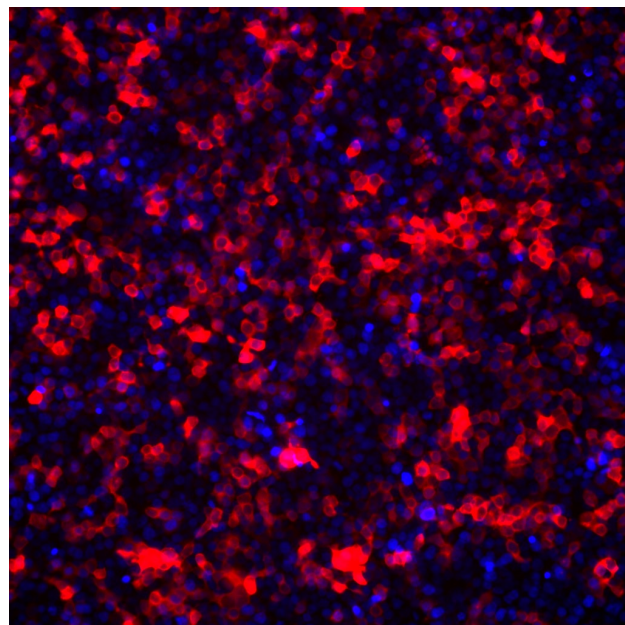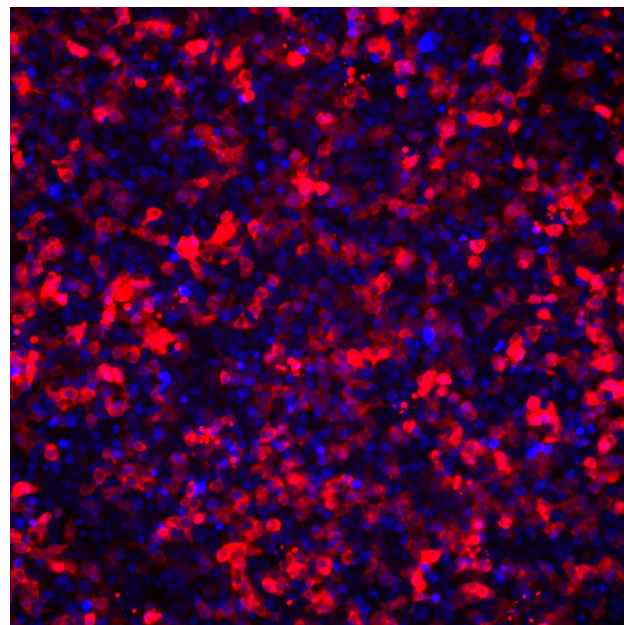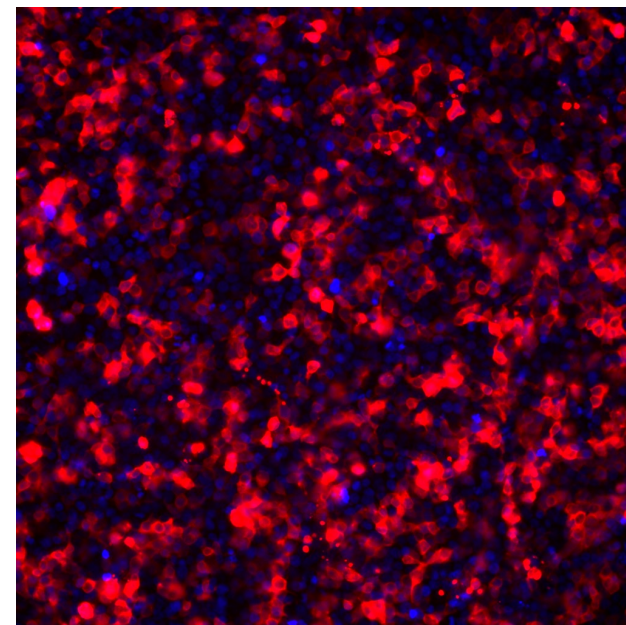

HOECHST mCHERRY

Supplement: Figure 4—source data 2. — Untagged full-length wild-type (WT) caspase-11 gene constructs were transfected at increasing doses, together with a fixed amount of indicated mCherry-tagged Casp11. 18 hr following transfection, the cells were imaged by fluorescence microscopy. Nuclei (blue) were stained with Hoechst. [file elife-83725-fig4-data2.zip › Figure 4-source data 2.pdf]

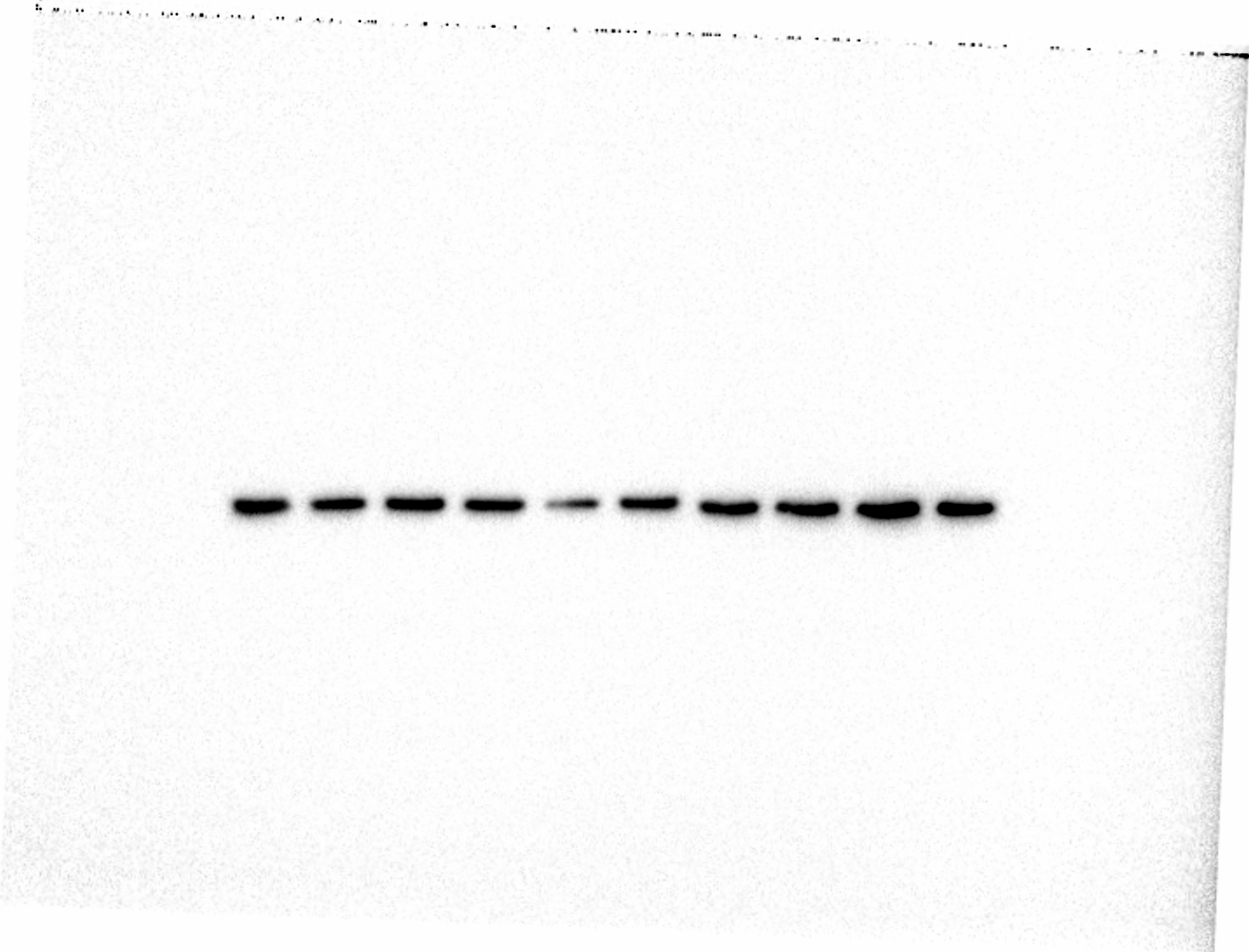

Supplement: Figure 4—source data 4. — Whole-cell lysates of indicated transfected cells were harvested and immunoblotted for mCherry. β-actin was used as loading control. [file elife-83725-fig4-data4.zip › Actin.tif]

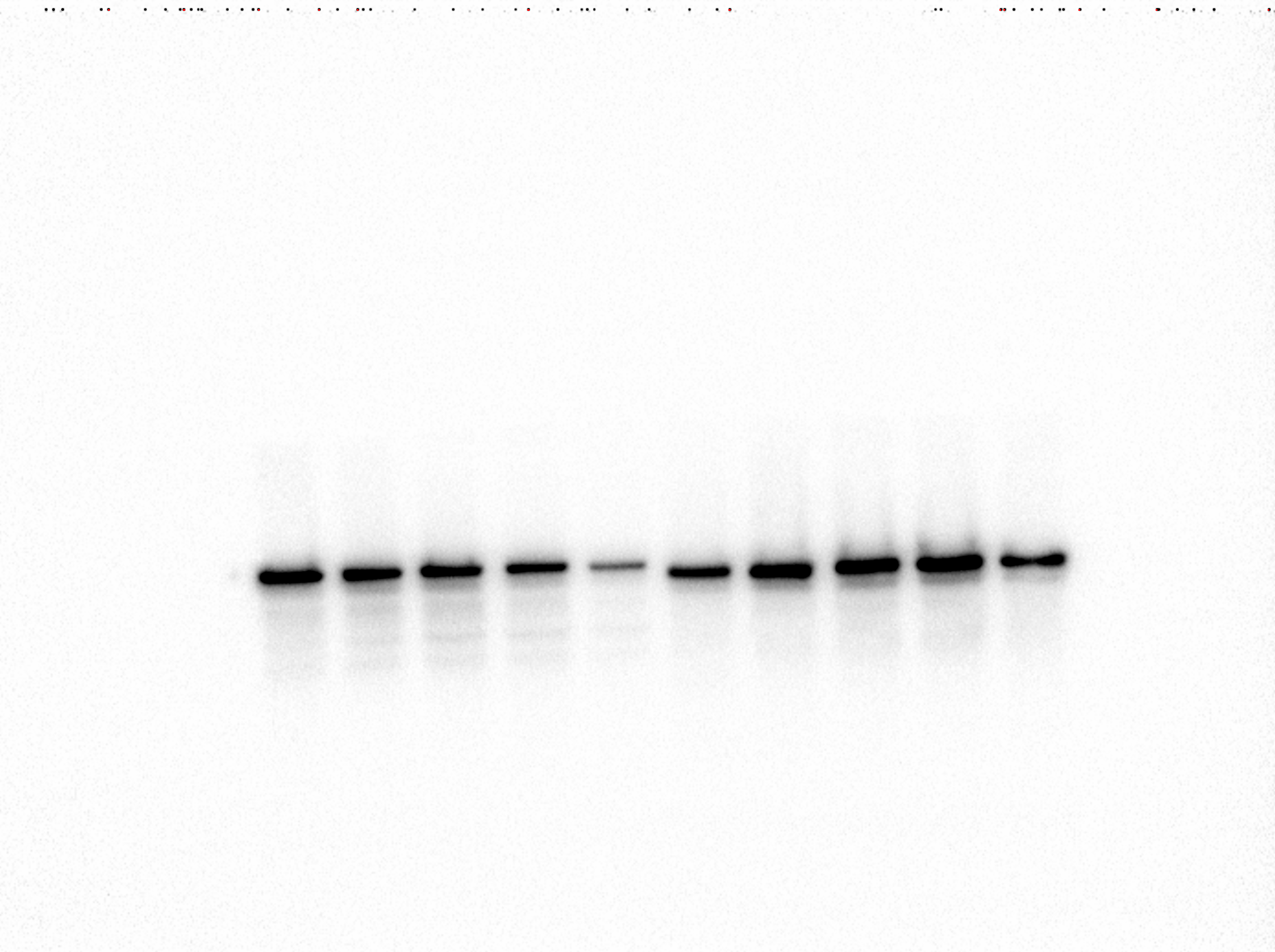

Supplement: Figure 4—source data 4. — Whole-cell lysates of indicated transfected cells were harvested and immunoblotted for mCherry. β-actin was used as loading control. [file elife-83725-fig4-data4.zip › Casp11-mCh.tif]

4F.

*CASP11*: WT

*CASP11-mCh*: C254A

*CASP11-mCh*: C254A/D285A

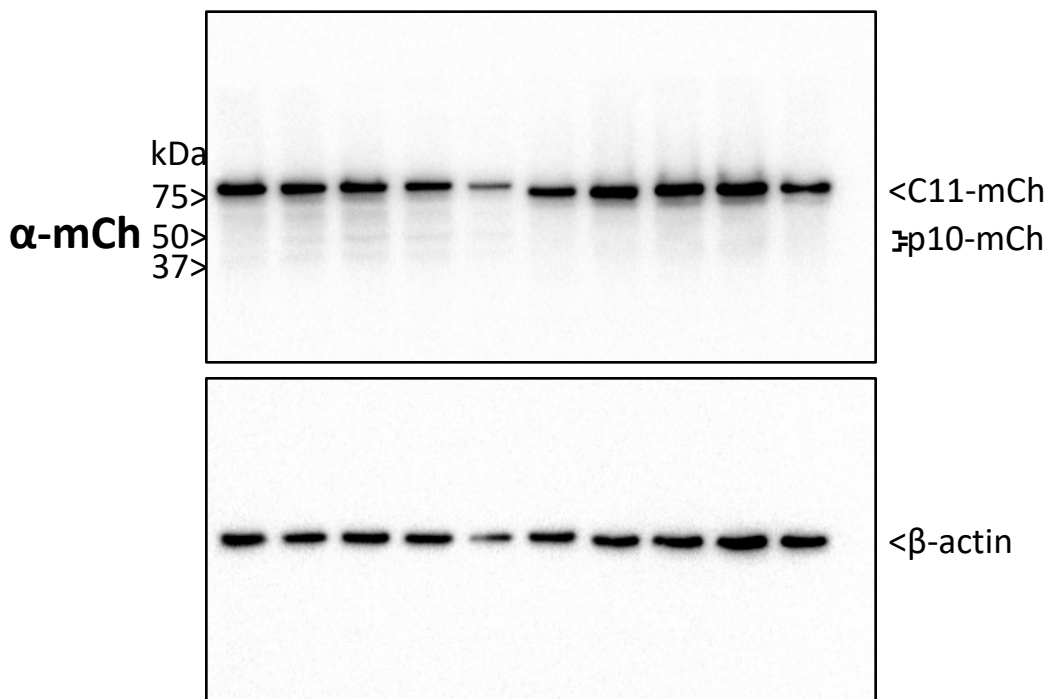

Supplement: Figure 4—source data 4. — Whole-cell lysates of indicated transfected cells were harvested and immunoblotted for mCherry. β-actin was used as loading control. [file elife-83725-fig4-data4.zip › Figure 4-source data 4.pdf]

## Slide 1
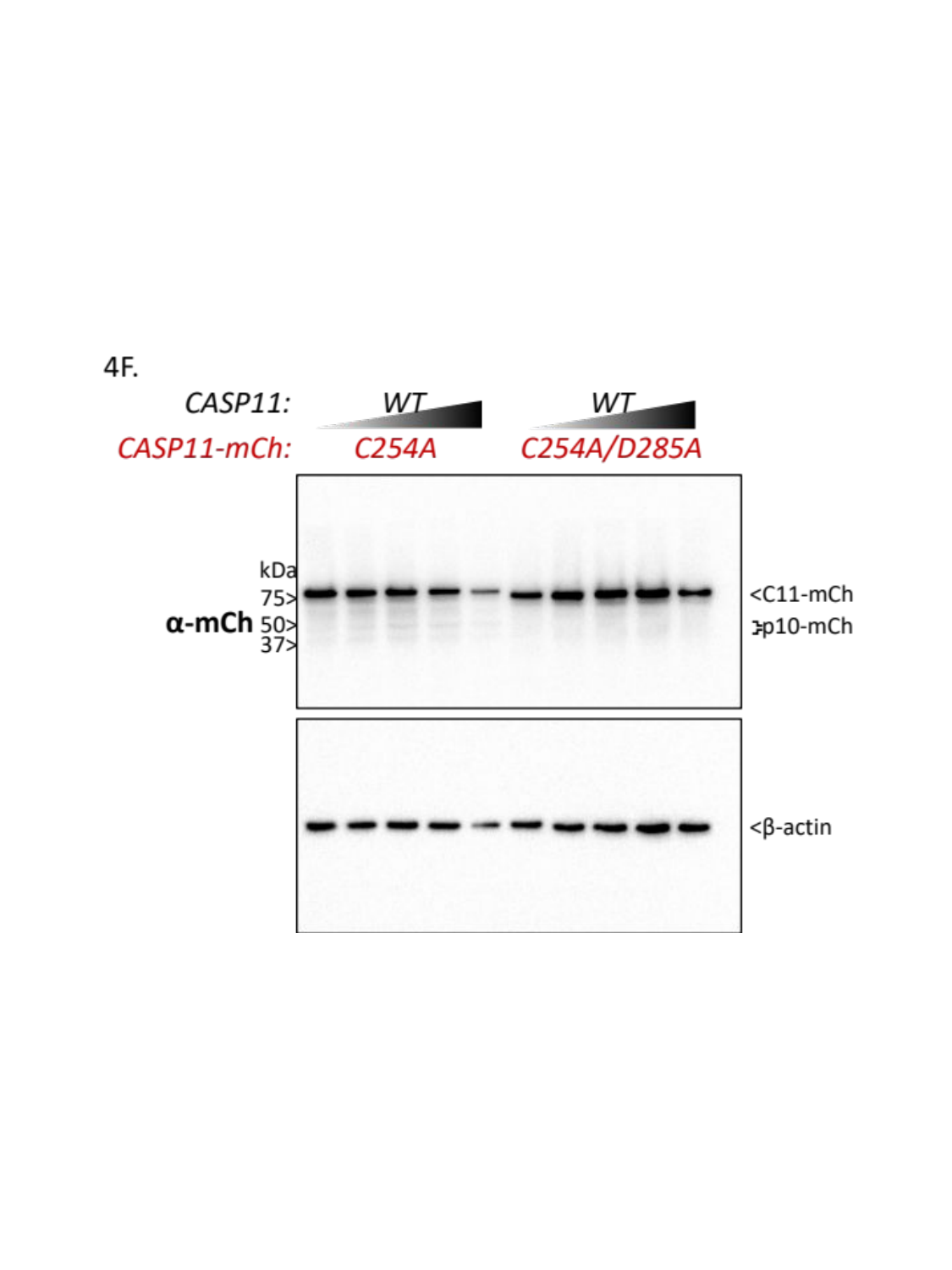

Supplement: Figure 4—source data 4. — Whole-cell lysates of indicated transfected cells were harvested and immunoblotted for mCherry. β-actin was used as loading control. [file elife-83725-fig4-data4.zip › Figure_4F_labeled.pptx]

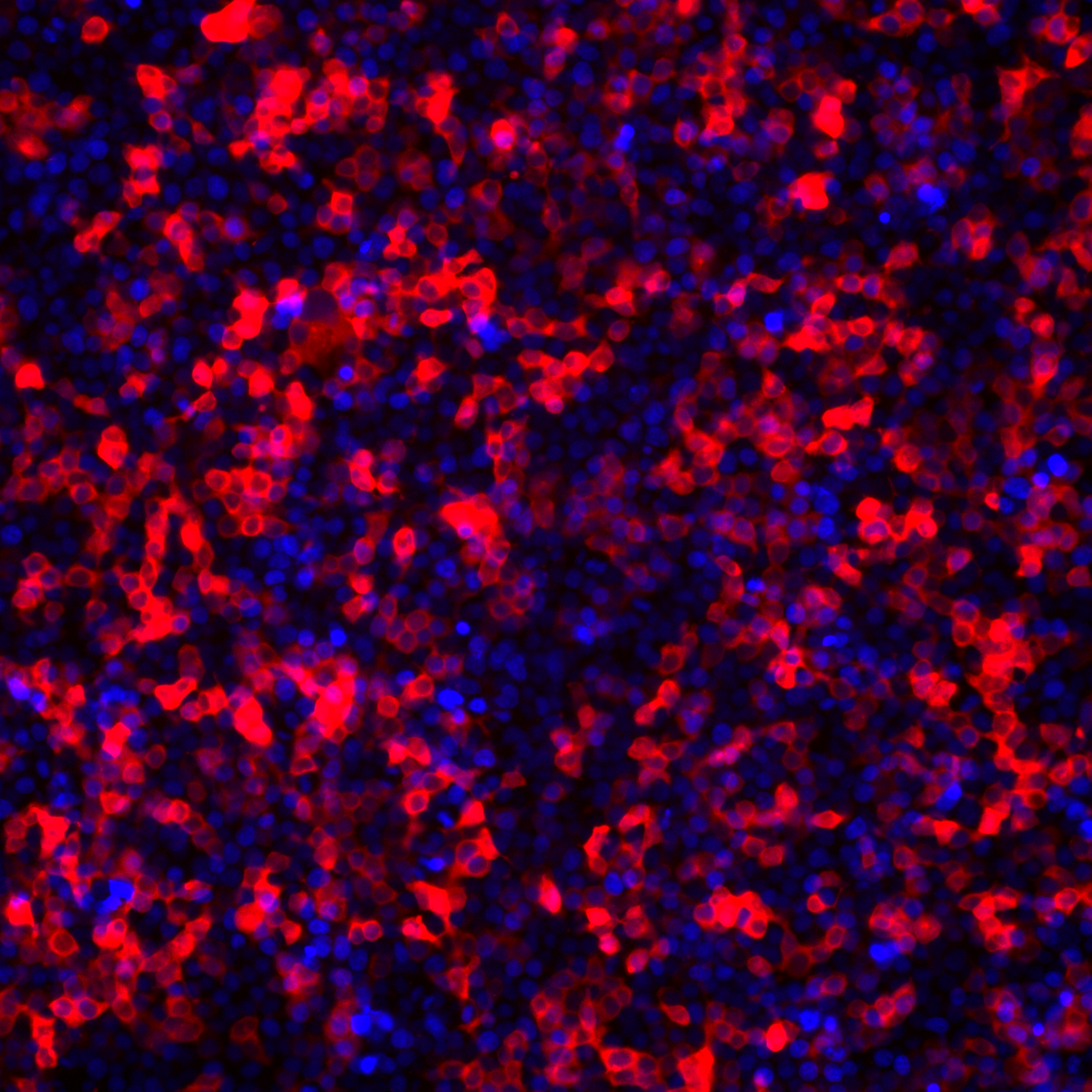

Supplement: Figure 4—source data 5. — HEK293T cells transfected with indicated Casp11 constructs were imaged by fluorescence microscopy. Nuclei (blue) were stained with Hoechst. [file elife-83725-fig4-data5.zip › 0_C11(WT)_with_C254A-mCh.tif]

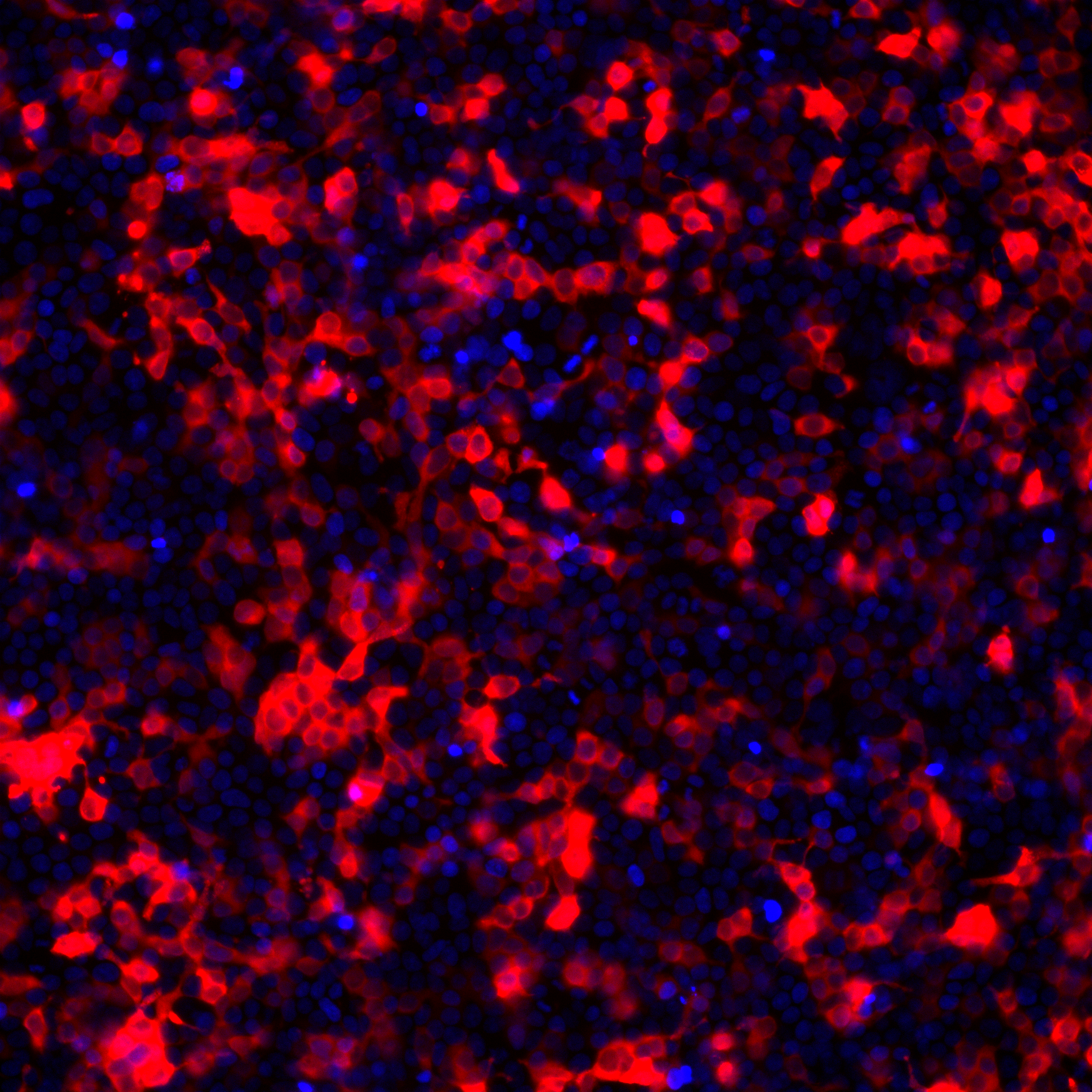

Supplement: Figure 4—source data 5. — HEK293T cells transfected with indicated Casp11 constructs were imaged by fluorescence microscopy. Nuclei (blue) were stained with Hoechst. [file elife-83725-fig4-data5.zip › 0_C11(WT)_with_C254A:D285A-mCh.tif]

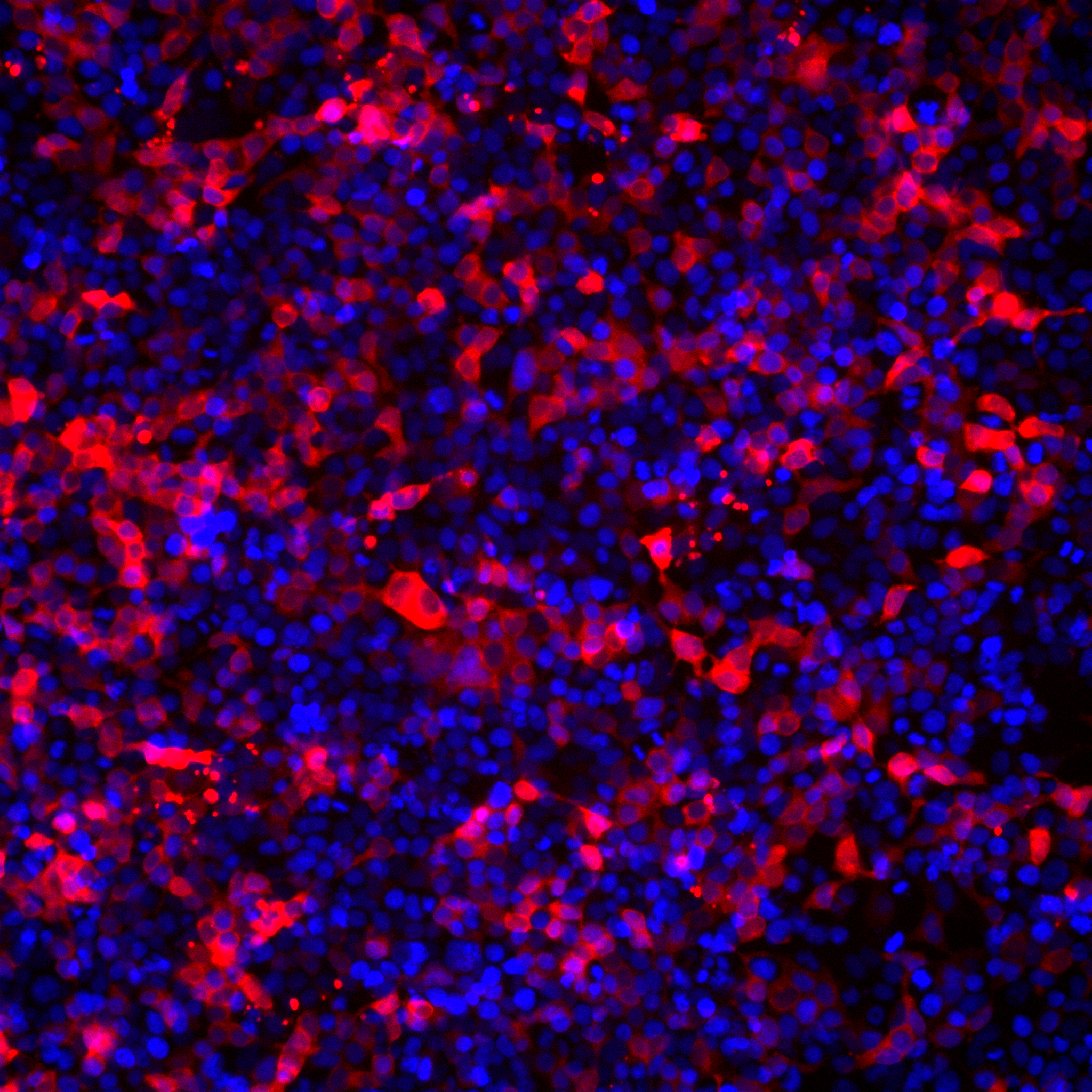

Supplement: Figure 4—source data 5. — HEK293T cells transfected with indicated Casp11 constructs were imaged by fluorescence microscopy. Nuclei (blue) were stained with Hoechst. [file elife-83725-fig4-data5.zip › 500_C11(WT)_with_C254A-mCh.tif]

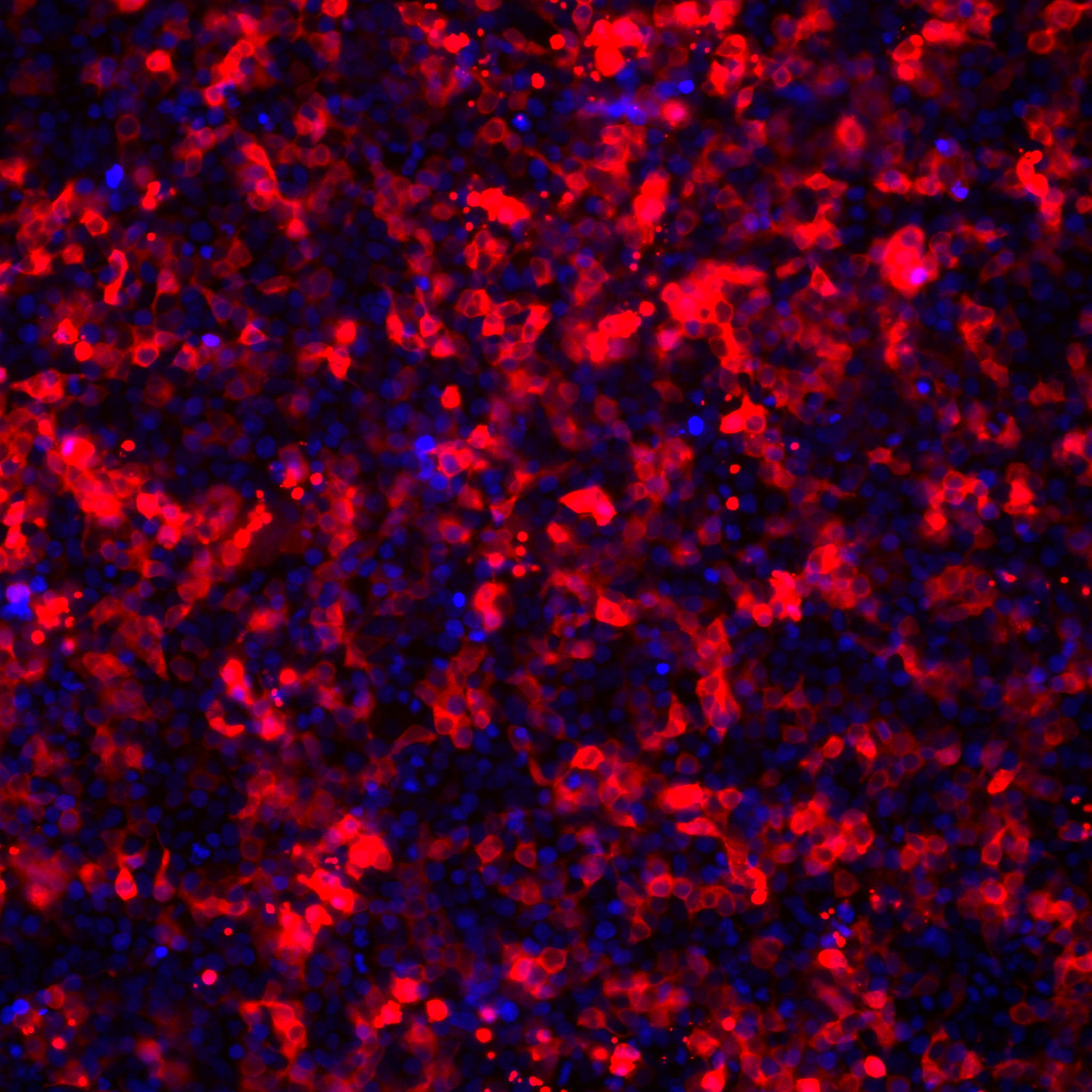

Supplement: Figure 4—source data 5. — HEK293T cells transfected with indicated Casp11 constructs were imaged by fluorescence microscopy. Nuclei (blue) were stained with Hoechst. [file elife-83725-fig4-data5.zip › 500_C11(WT)_with_C254A:D285A-mCh.tif]

Fig 4-Source Data 5 (4G)

*WT CASP11:*

0 ng

500 ng

*C254A*

*CASP11-mCh:*

*C254A/  
D285A*

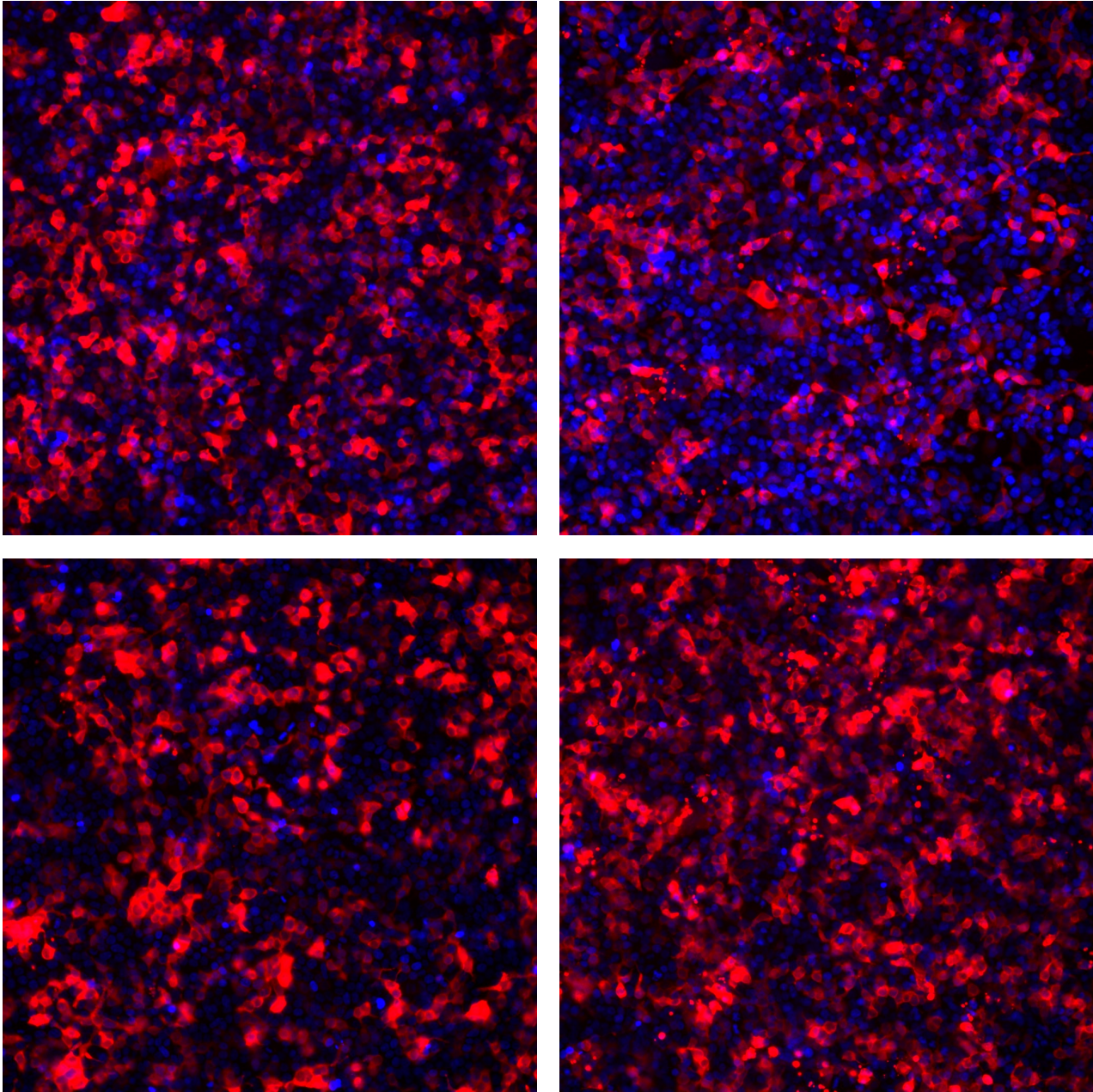

HOECHST mCHERRY

Supplement: Figure 4—source data 5. — HEK293T cells transfected with indicated Casp11 constructs were imaged by fluorescence microscopy. Nuclei (blue) were stained with Hoechst. [file elife-83725-fig4-data5.zip › Figure 4-source data 5.pdf]
