# Supplementary material for: Catalytic activity and autoprocessing of murine caspase-11 mediate noncanonical inflammasome assembly in response to cytosolic LPS
Source: eLife. 2024 Jan 17;13:e83725. doi: 10.7554/eLife.83725 (PMC10794067; doi:10.7554/eLife.83725)
Supplement: Figure 2—source data 5. — Speck formation in Figure 2E was quantified as percentage of Casp11-mCherry-expressing cells containing at least one speck. Dose–response curves were plotted by least-squares nonlinear regression ([Log2(inhibitor) vs. response (three parameters)]; Y = Bottom + (Top-Bottom)/(1 + 10(X-LogIC50)); R2 indicated). [file elife-83725-fig2-data5.zip › Figure 2-source data 5.pdf]

Raw values for Figure 2F

		% Speck formation					
[zVAD] (µM)	Log(2)[zVAD]	WT			C254A		
DMSO	1.64385619	31.6091954	30.56478	31.08699	4.347826	3.040541	3.694183
25	4.64385619	12.37113402	8.759124	10.56513	2.222222	3.754266	2.988244
50	5.64385619	5.64516129	1.408451	3.526806	0	4.035874	2.017937
100	6.64385619	3.015075377	5.095541	4.055308	2.380952	3.313253	2.847103
200	7.64385619	4.504504505	0.653595	2.57905	2.966102	3.125	3.045551

Statistics	WT	C254A
Log(inhibitor) vs. response (three parameters)]		
Y=Bottom + (Top-Bottom)/(1+10^(X-LogIC50))		
Best-fit values		
Bottom	3.046	2.658
Top	31.17	3.702
LogIC50	4.201	4.094
IC50	15869	12416
Span	28.12	1.044
95% CI (profile likelihood)		
Bottom	1.829 to 4.257	1.867 to 3.359
Top	29.18 to 33.16	2.452 to ???
LogIC50	3.994 to 4.368	???
IC50	9869 to 23332	???
Goodness of Fit		
Degrees of Freedom	12	12
R squared	0.9832	0.1601
Sum of Squares	29.54	12.55
Sy.x	1.569	1.022
Number of points		
# of X values	15	15
# Y values analyzed	15	15
