# Supplementary material for: Catalytic activity and autoprocessing of murine caspase-11 mediate noncanonical inflammasome assembly in response to cytosolic LPS
Source: eLife. 2024 Jan 17;13:e83725. doi: 10.7554/eLife.83725 (PMC10794067; doi:10.7554/eLife.83725)
Supplement: Figure 4—source data 3. — Speck formation in Figure 4C was quantified as percentage of Casp11-mCherry-expressing cells containing at least one speck. Dose–response curves were plotted by least-squares nonlinear regression ([Log2(agonist) vs. response (three parameters)]; Y = Bottom + (Top-Bottom)/(1 + 10(LogEC50-X))). [file elife-83725-fig4-data3.zip › Figure 4-source data 3.pdf]

		% Speck formation					
**Plasmid amount (ng)**	**Log(2) (plasmid)**	**WT-mCh**			**C254A-mCh**		
0	4.96578428	19.20904	27.77778	26.0274	0	0.46729	1.449275
62.5	5.96578428	25.342466	22.44898	20.75472	0	0	5.517241
125	6.96578428	31.443299	20.18779	27.5	6.796117	8.888889	12.23022
250	7.96578428	27.922078	24.8731	27.86885	11.05769	8.900524	17.85714
500	8.96578428	35.333333	33.73494	37.2093	16	21.01449	25

**Statistics**	**WT-mCh**	**C254A-mCh**
Log(agonist) vs. response (three parameters)		
Y=Bottom + (Top-Bottom)/(1+10^(LogEC50-X))		
Best-fit values		
Bottom	24.32	0.9816
Top	40.83	18.56
LogEC50	8.655	7.181
EC50	451474764	15181184
Span	16.51	17.57
95% CI (profile likelihood)		
Bottom	21.58 to 26.92	-3.574 to 5.514
Top	31.68 to ???	14.11 to 28.17
LogEC50	7.439 to ???	6.374 to 8.417
EC50	27451337 to ??	2365515 to 261150262
Goodness of Fit		
Degrees of Freedom	12	12
R squared	0.6512	0.8034
Sum of Squares	145	183.3
Sy.x	3.476	3.908
Number of points		
# of X values	15	15
# Y values analyzed	15	15
