# Supplementary material for: Catalytic activity and autoprocessing of murine caspase-11 mediate noncanonical inflammasome assembly in response to cytosolic LPS
Source: eLife. 2024 Jan 17;13:e83725. doi: 10.7554/eLife.83725 (PMC10794067; doi:10.7554/eLife.83725)
Supplement: Figure 4—source data 6. — Speck formation in Figure 4G was quantified as percentage of Casp11-mCherry-expressing cells containing at least one speck. Dose–response curves were plotted by least-squares nonlinear regression ([Log2(agonist) vs. response (three parameters)]; Y=Bottom + (Top-Bottom)/(1+10(LogEC50-X))). [file elife-83725-fig4-data6.zip › Figure 4-source data 6.pdf]

		% Speck formation					
**Plasmid amount (ng)**	**Log(2) (plasmid)**	**C254A-mCh**			**C254A/D285A-mCh**		
0	5.96578428	2.522255193	3.737113	2.120536	2.102804	1.544944	1.612903
125	6.96578428	3.03030303	6.954436	4	5.122951	6.280193	2.869565
250	7.96578428	8.007117438	6.862745	14.4	12.33766	7.142857	8.716475
500	8.96578428	14.37198068	16.44022	12.16545	15.38462	17.79661	21.90813

**Statistics**	**C254A-mCh**	**C254A/D285A-mCh**
Log(agonist) vs. response (three parameters)		
Y=Bottom + (Top-Bottom)/(1+10^(LogEC50-X))		
Best-fit values		
Bottom	2.872	2.539
Top	15.12	20.77
LogEC50	7.837	8.159
EC50	68701919	144063072
Span	12.25	18.23
95% CI (profile likelihood)		
Bottom	-0.4699 to 5.54	-0.03622 to 4.975
Top	11.37 to 20.83	16.25 to 30.91
LogEC50	7.012 to 8.647	7.638 to 8.857
EC50	10280550 to 44	43469665 to 720097869
Goodness of Fit		
Degrees of Freedom	9	9
R squared	0.8226	0.9057
Sum of Squares	52.49	48.57
Sy.x	2.415	2.323
Number of points		
# of X values	12	12
# Y values analyzed	12	12
