# Supplementary material for: Catalytic activity and autoprocessing of murine caspase-11 mediate noncanonical inflammasome assembly in response to cytosolic LPS
Source: eLife. 2024 Jan 17;13:e83725. doi: 10.7554/eLife.83725 (PMC10794067; doi:10.7554/eLife.83725)
Supplement: Figure 4—figure supplement 1—source data 1. — Unlabeled wild-type (WT) Casp11 was transfected into HEK293T cells at increasing doses, together with a fixed dose (0.25 μg) of indicated mCherry-tagged Casp11 construct and a fixed dose of murine gasdermin D (GSDMD, 0.05 μg). 14 hr post-transfection, Casp11-mediated cytotoxicity was measured by determining percent lactate dehydrogenase (LDH) release. Dose–response curves in (B) were plotted by least-squares nonlinear regression ([Log2(agonist) vs. response (three parameters)]; Y = Bottom + (Top-Bottom)/(1 + 10(LogEC50-X))). [file elife-83725-fig4-figsupp1-data1.zip › Figure 4-figure supplement 1-source data 1.pdf]

mCh-tagged Plasmid amount (ng)	Log(2) (mCh-tagged plasmid)	% Cytotoxicity (% LDH release) Empty			C254A-mCh			C254A/D285A-mCh		
0	5.96578428	0.70493454	0.04028197	4.3138332	7.28005127	0.28746681	5.83539321	3.41298178	17.0356129	0.51817266
125	6.96578428	13.5969972	21.1718392	19.3701364	22.4297354	16.6346242	12.8114987	6.34624187	23.6381946	18.8922457
250	7.96578428	16.854344	12.9158656	13.0916415	22.4297354	20.3039458	13.4486863	12.0864231	29.4827428	34.1298178
500	8.96578428	19.2767555	18.7054838	31.4162776	17.7991394	32.4050169	16.854344	12.7895267	16.0908175	35.1460222

Statistics	Empty	C254A-mCh	C254A/D285A-mCh
Log(agonist) vs. response (three parameters)			
Y=Bottom + (Top-Bottom)/(1+10^(LogEC50-X))			
Best-fit values			
Bottom	-1148	-4.958	3.657
Top	19.13	20.89	23.59
LogEC50	4.146	6.206	6.676
EC50	14010	1607592	4742777
Span	1167	25.85	19.94
95% CI (profile likelihood)			
Bottom	???	??? to 11.05	??? to 19.44
Top	14.83 to 24.62	15.78 to 26.84	14.14 to ???
LogEC50	??? to 7.150	??? to 7.705	???
EC50	??? to 141336[illegible]	??? to 507372[illegible]	???
Goodness of Fit			
Degrees of Freedom	9	9	9
R squared	0.7027	0.6497	0.3651
Sum of Squares	270.1	286.1	911.8
Sy.x	5.478	5.638	10.07
Number of points			
# of X values	12	12	12
# Y values analyzed	12	12	12
