# Supplementary figures and images for: Catalytic activity and autoprocessing of murine caspase-11 mediate noncanonical inflammasome assembly in response to cytosolic LPS (part 2 of 2)
Source: eLife. 2024 Jan 17;13:e83725. doi: 10.7554/eLife.83725 (PMC10794067; doi:10.7554/eLife.83725)

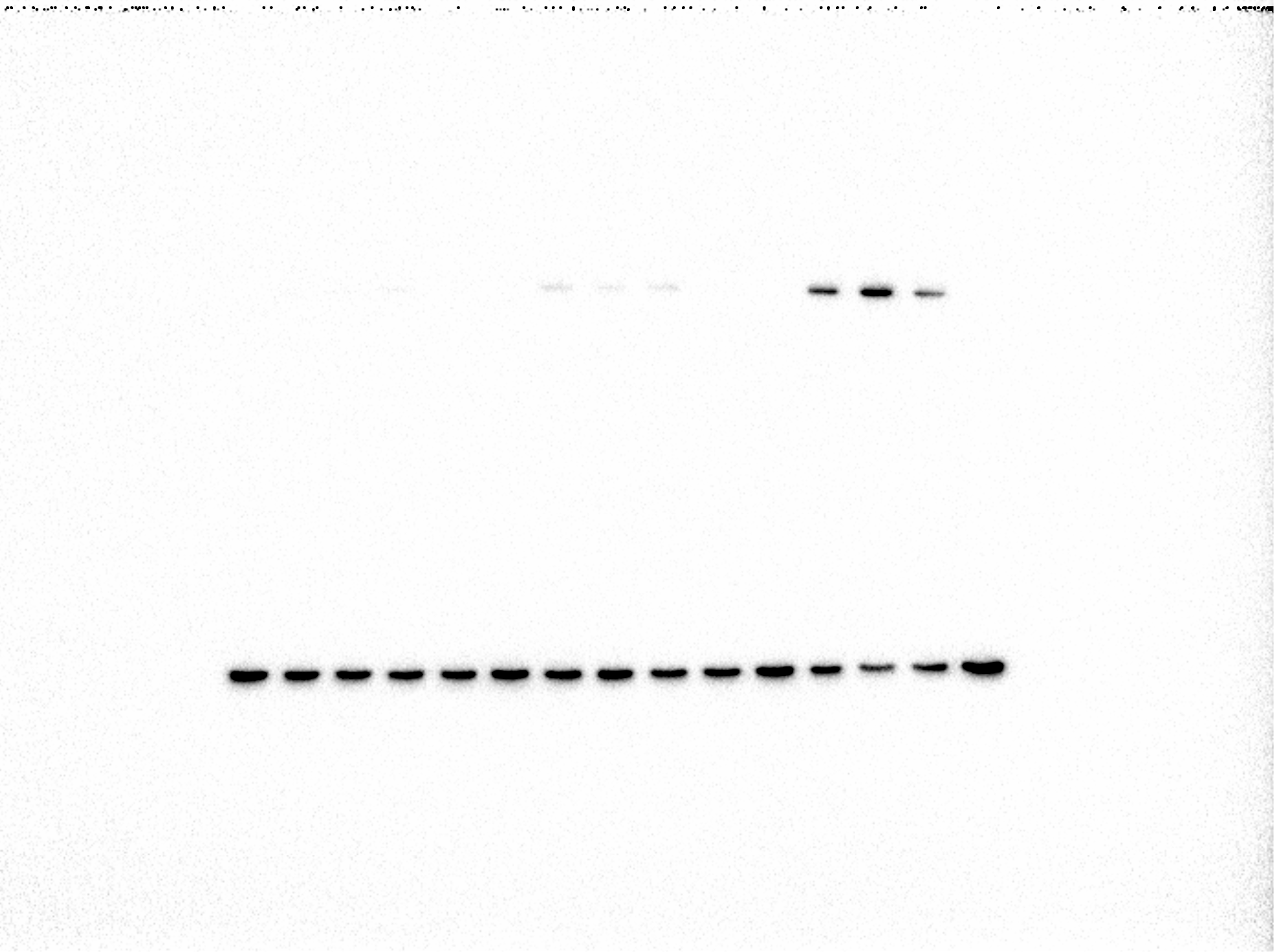

Supplement: Figure 4—figure supplement 1—source data 2. — HEK293T whole-cell lysates and supernatants (sup) from Figure 4—figure supplement 1B were immunoblotted for gasdermin D (GSDMD). β-actin is indicated as loading control. [file elife-83725-fig4-figsupp1-data2.zip › Actin.tif]

Figure 4-figure supplement 1C

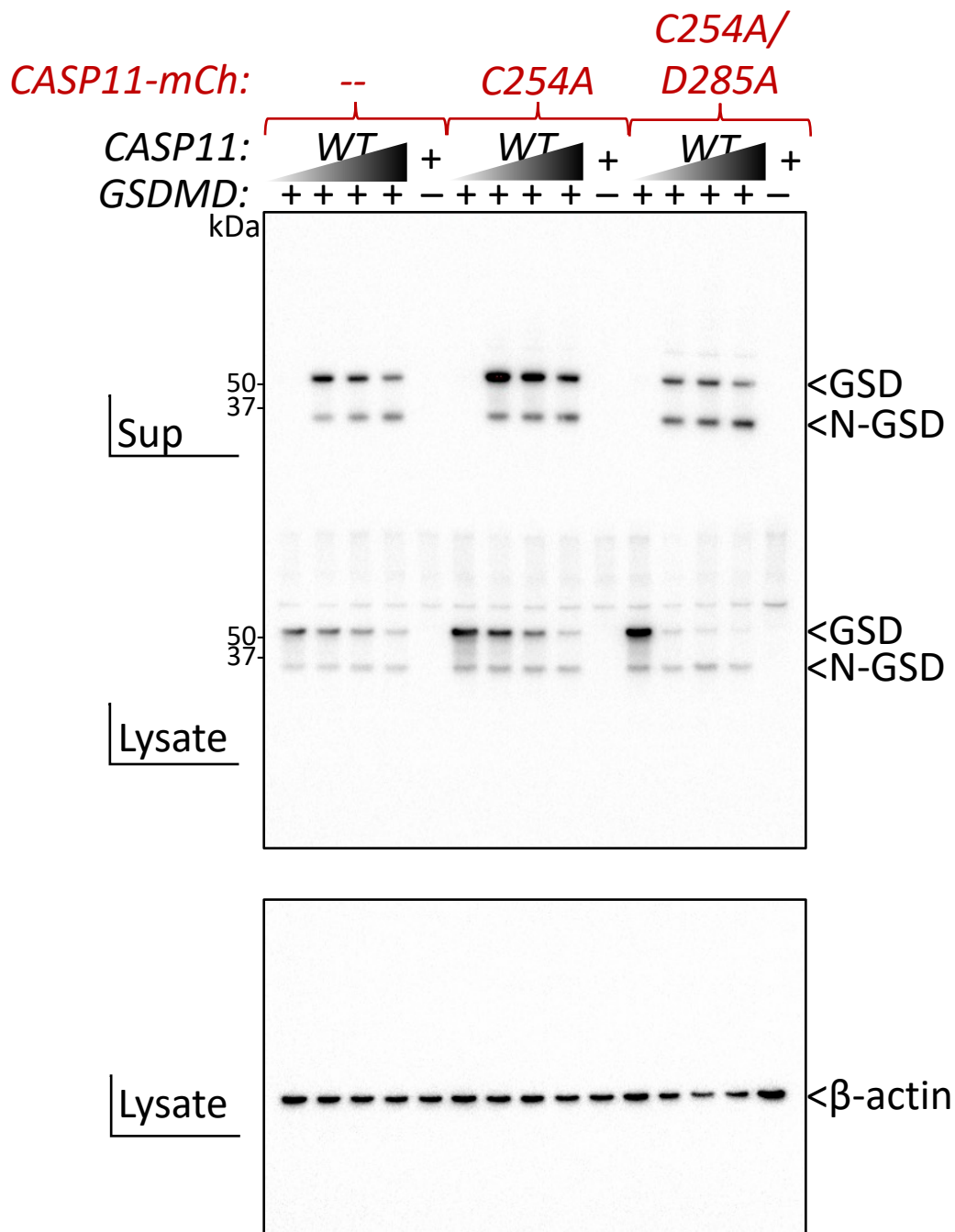

Supplement: Figure 4—figure supplement 1—source data 2. — HEK293T whole-cell lysates and supernatants (sup) from Figure 4—figure supplement 1B were immunoblotted for gasdermin D (GSDMD). β-actin is indicated as loading control. [file elife-83725-fig4-figsupp1-data2.zip › Figure 4-figure supplement 1-source data 2.pdf]

## Slide 1
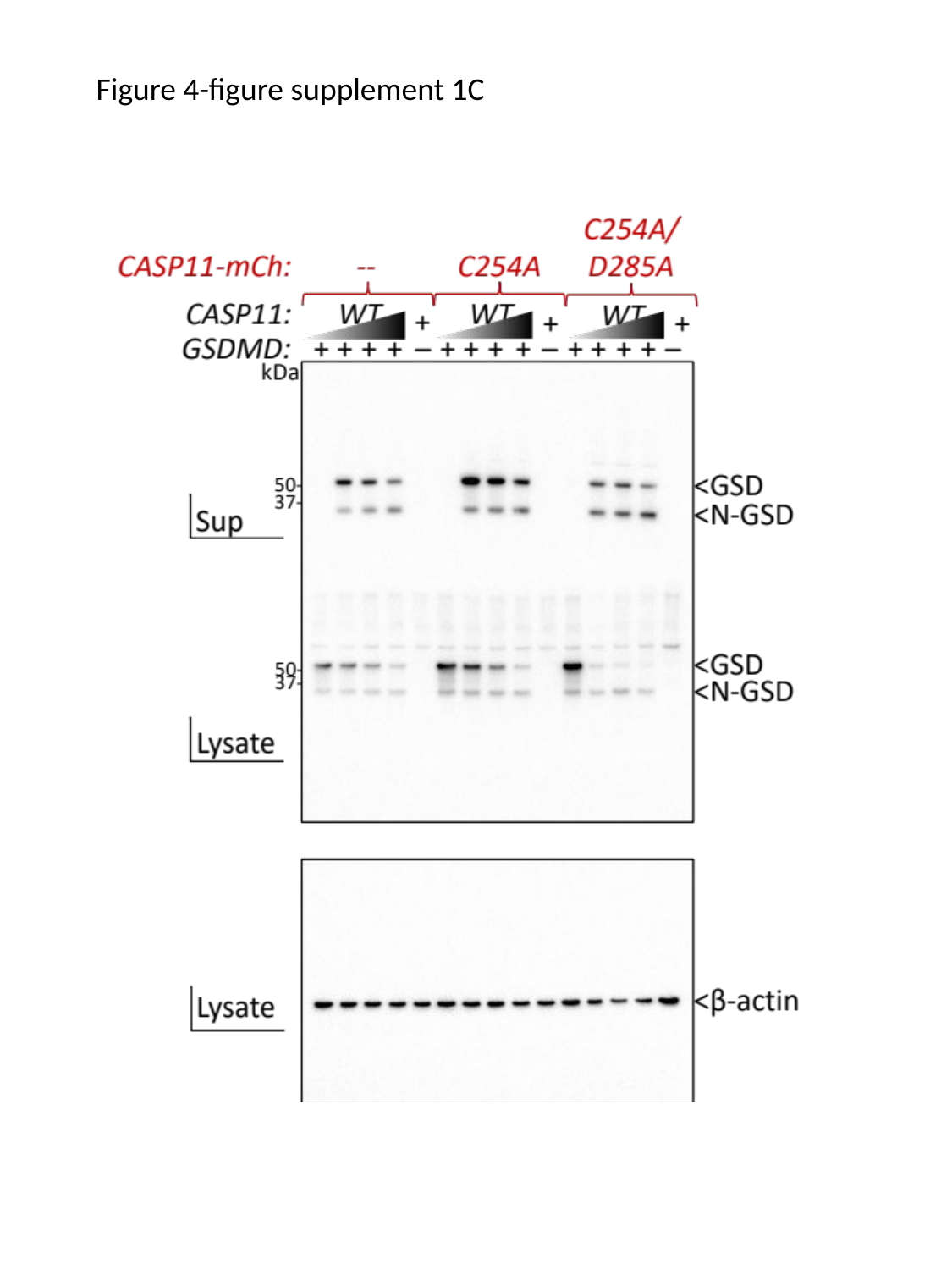

Figure 4-figure supplement 1C

Supplement: Figure 4—figure supplement 1—source data 2. — HEK293T whole-cell lysates and supernatants (sup) from Figure 4—figure supplement 1B were immunoblotted for gasdermin D (GSDMD). β-actin is indicated as loading control. [file elife-83725-fig4-figsupp1-data2.zip › Figure4-figure supplement 1C_labeled.pptx]

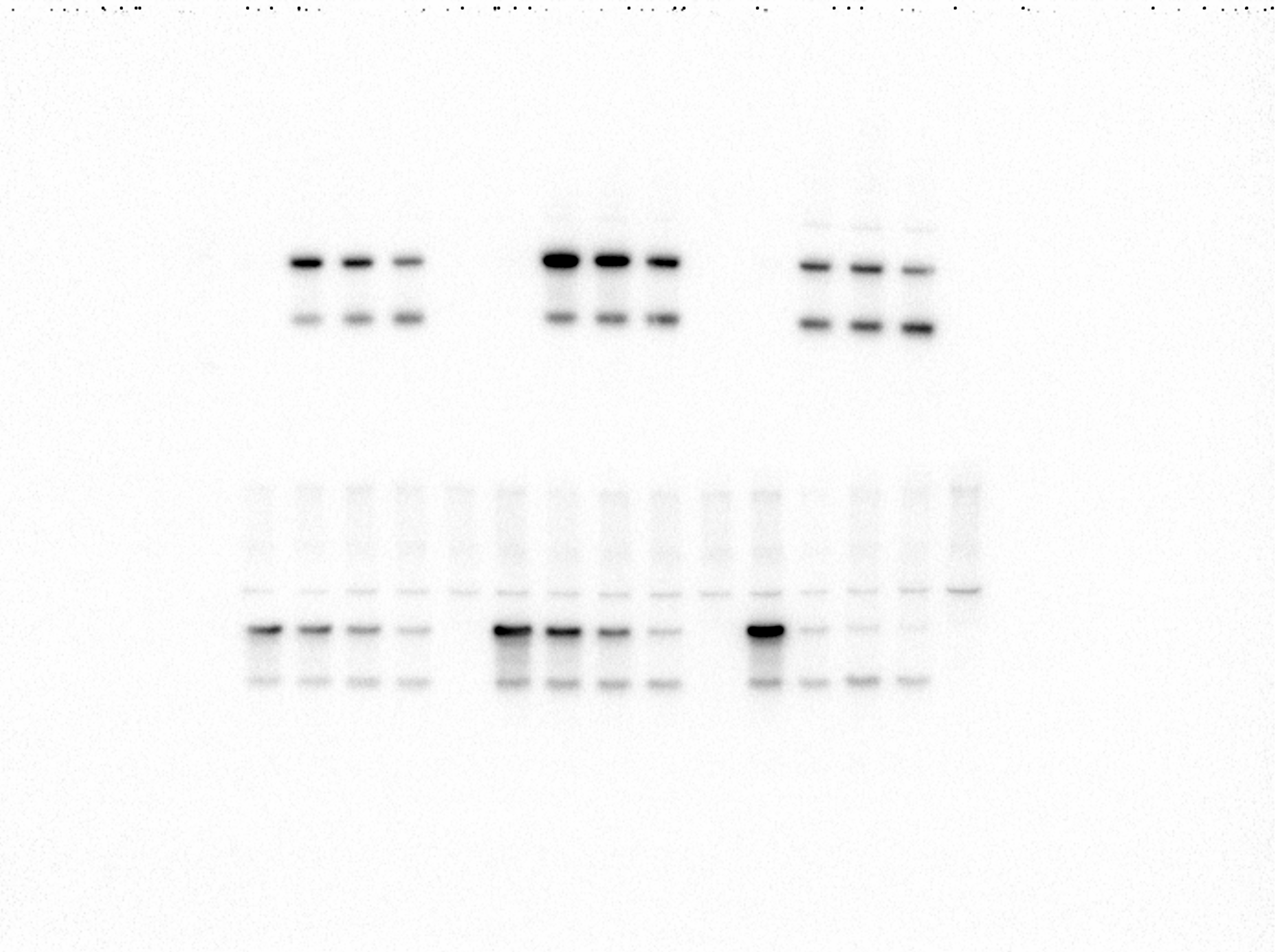

Supplement: Figure 4—figure supplement 1—source data 2. — HEK293T whole-cell lysates and supernatants (sup) from Figure 4—figure supplement 1B were immunoblotted for gasdermin D (GSDMD). β-actin is indicated as loading control. [file elife-83725-fig4-figsupp1-data2.zip › GSDMD_Lysate_Supernatant.tif]

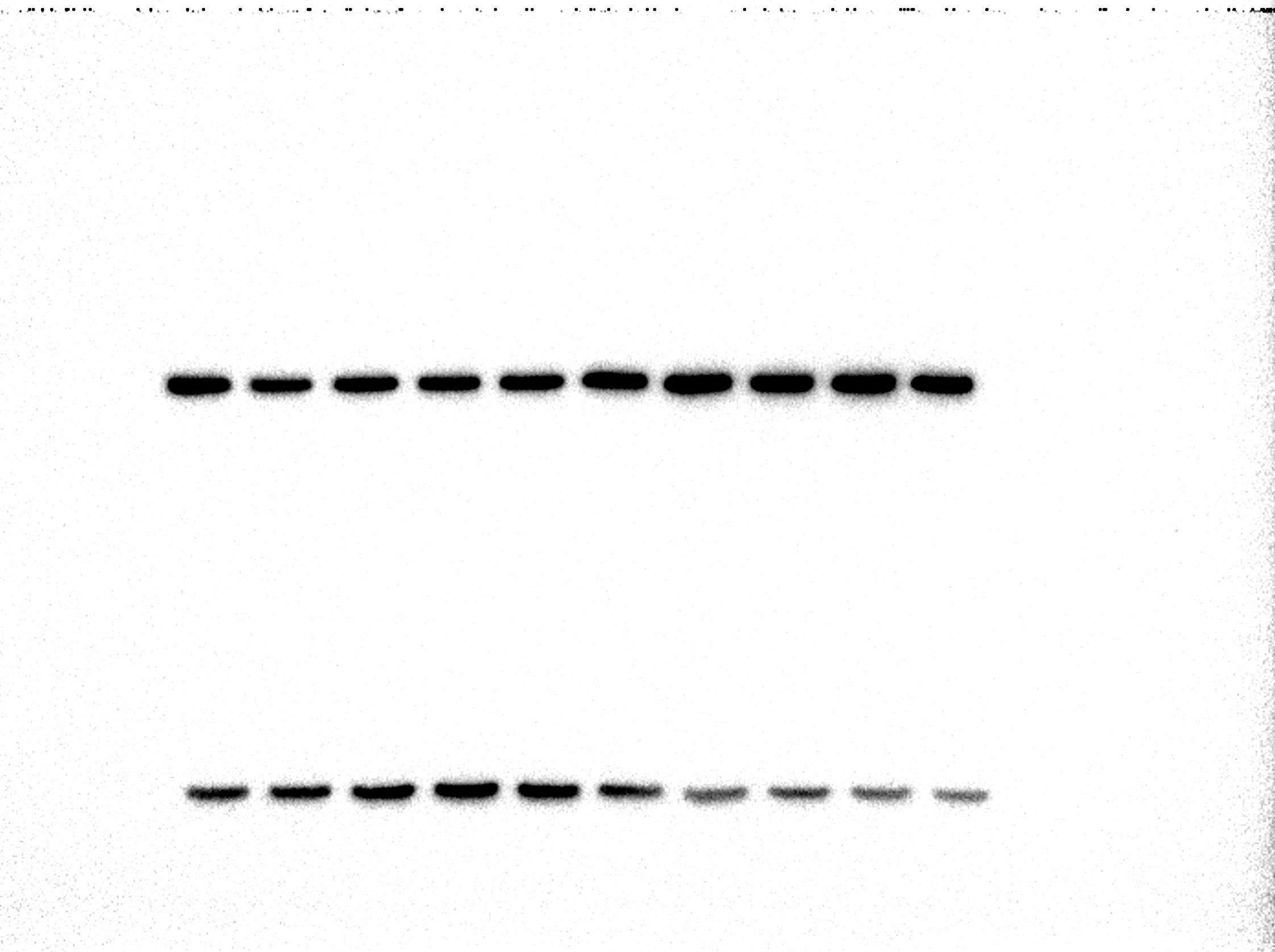

Supplement: Figure 5—source data 1. — Unlabeled wild-type (WT), catalytically inactive (C254A), or non-cleavable (D285A) caspase-11 constructs were transfected into HEK293T cells at increasing doses, together with a fixed dose of catalytically inactive (C254A) mCherry-tagged caspase-11. 12 hr post-transfection, whole-cell lysates were harvested and immunoblotted for mCherry or β-actin as loading control. [file elife-83725-fig5-data1.zip › Actin_2.tif]

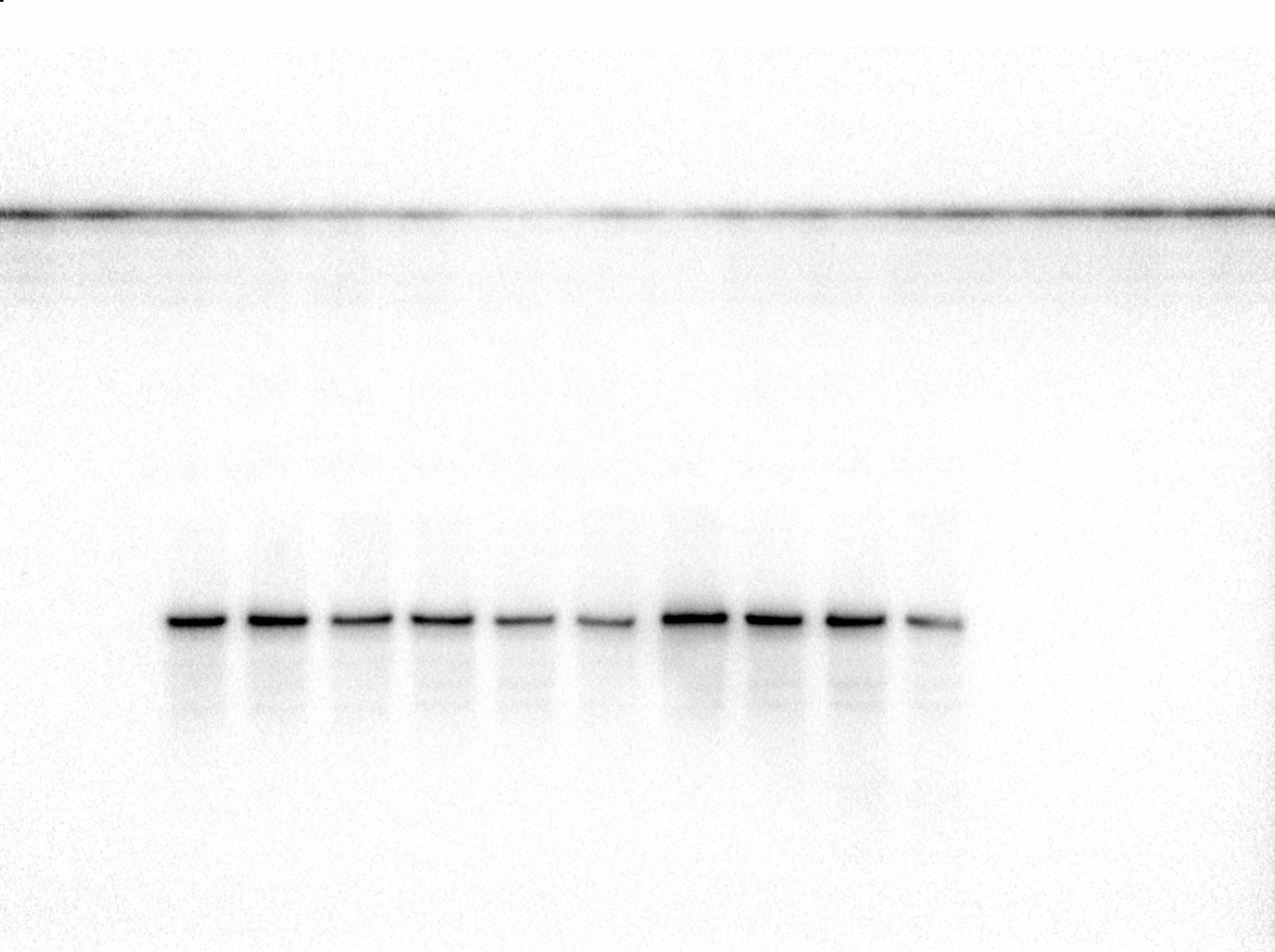

Supplement: Figure 5—source data 1. — Unlabeled wild-type (WT), catalytically inactive (C254A), or non-cleavable (D285A) caspase-11 constructs were transfected into HEK293T cells at increasing doses, together with a fixed dose of catalytically inactive (C254A) mCherry-tagged caspase-11. 12 hr post-transfection, whole-cell lysates were harvested and immunoblotted for mCherry or β-actin as loading control. [file elife-83725-fig5-data1.zip › Casp11-mCh_2.tif]

5B.

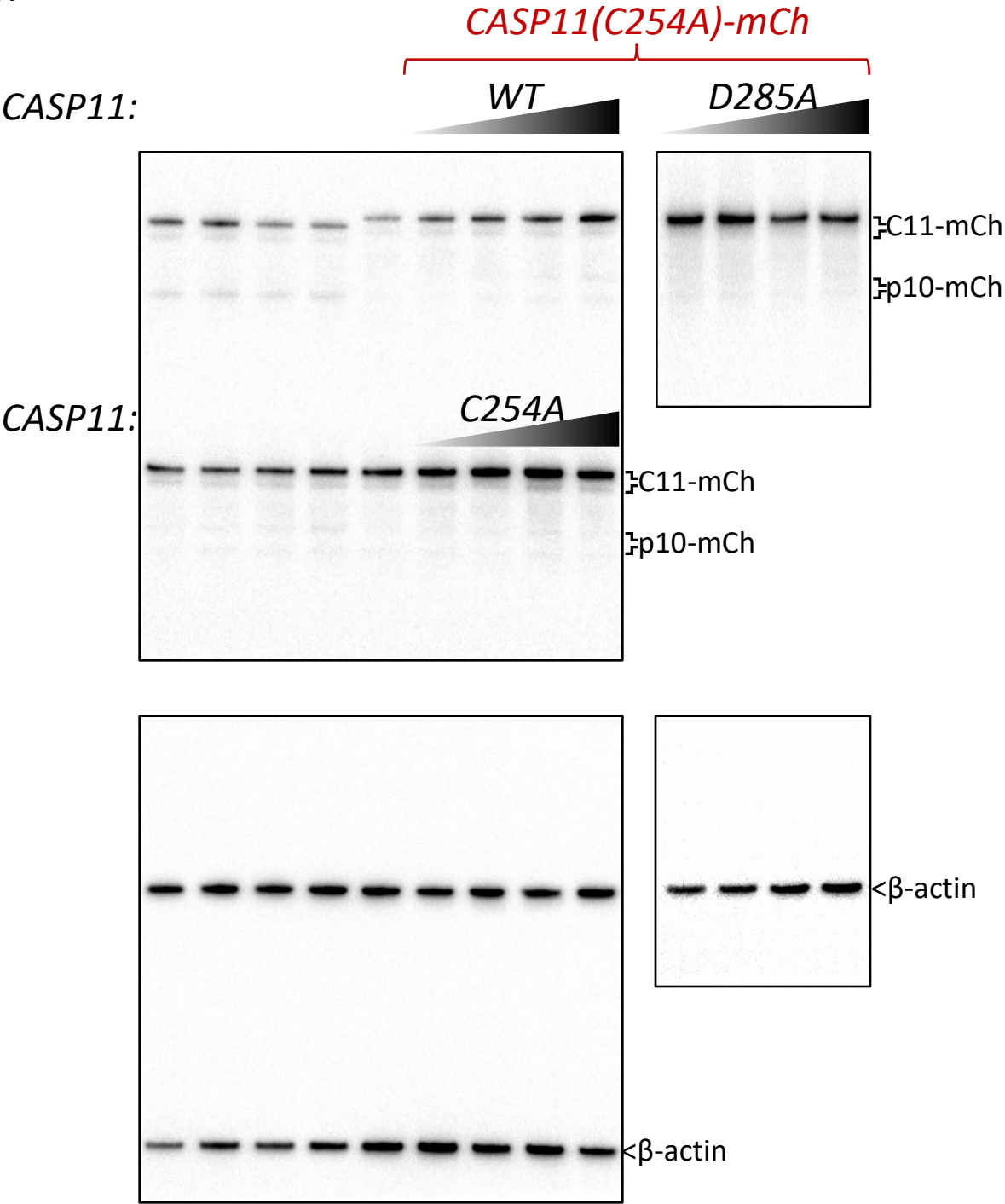

Supplement: Figure 5—source data 1. — Unlabeled wild-type (WT), catalytically inactive (C254A), or non-cleavable (D285A) caspase-11 constructs were transfected into HEK293T cells at increasing doses, together with a fixed dose of catalytically inactive (C254A) mCherry-tagged caspase-11. 12 hr post-transfection, whole-cell lysates were harvested and immunoblotted for mCherry or β-actin as loading control. [file elife-83725-fig5-data1.zip › Figure 5-source data 1.pdf]

## Slide 1
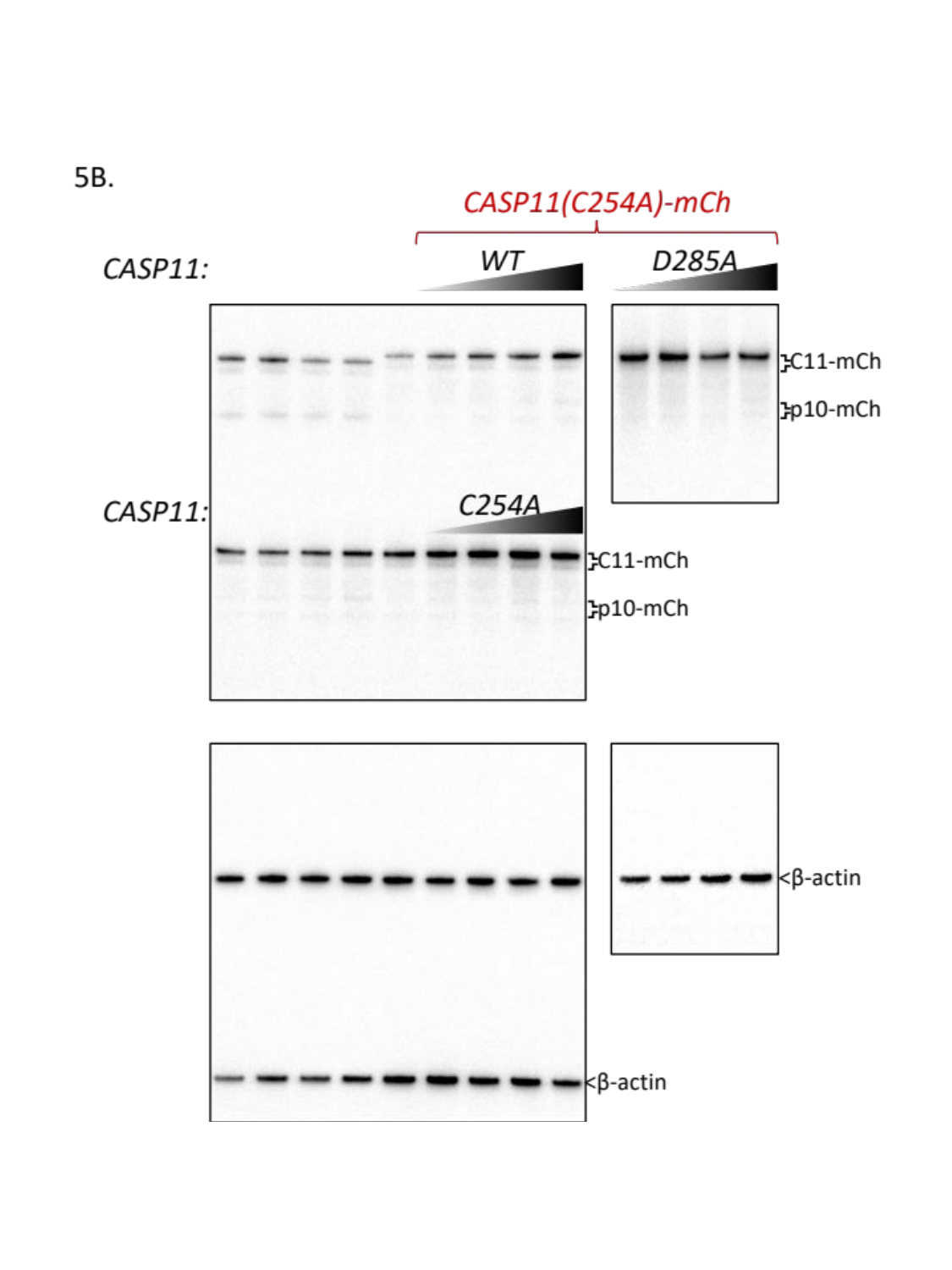

Supplement: Figure 5—source data 1. — Unlabeled wild-type (WT), catalytically inactive (C254A), or non-cleavable (D285A) caspase-11 constructs were transfected into HEK293T cells at increasing doses, together with a fixed dose of catalytically inactive (C254A) mCherry-tagged caspase-11. 12 hr post-transfection, whole-cell lysates were harvested and immunoblotted for mCherry or β-actin as loading control. [file elife-83725-fig5-data1.zip › Figure_5B_labeled.pptx]

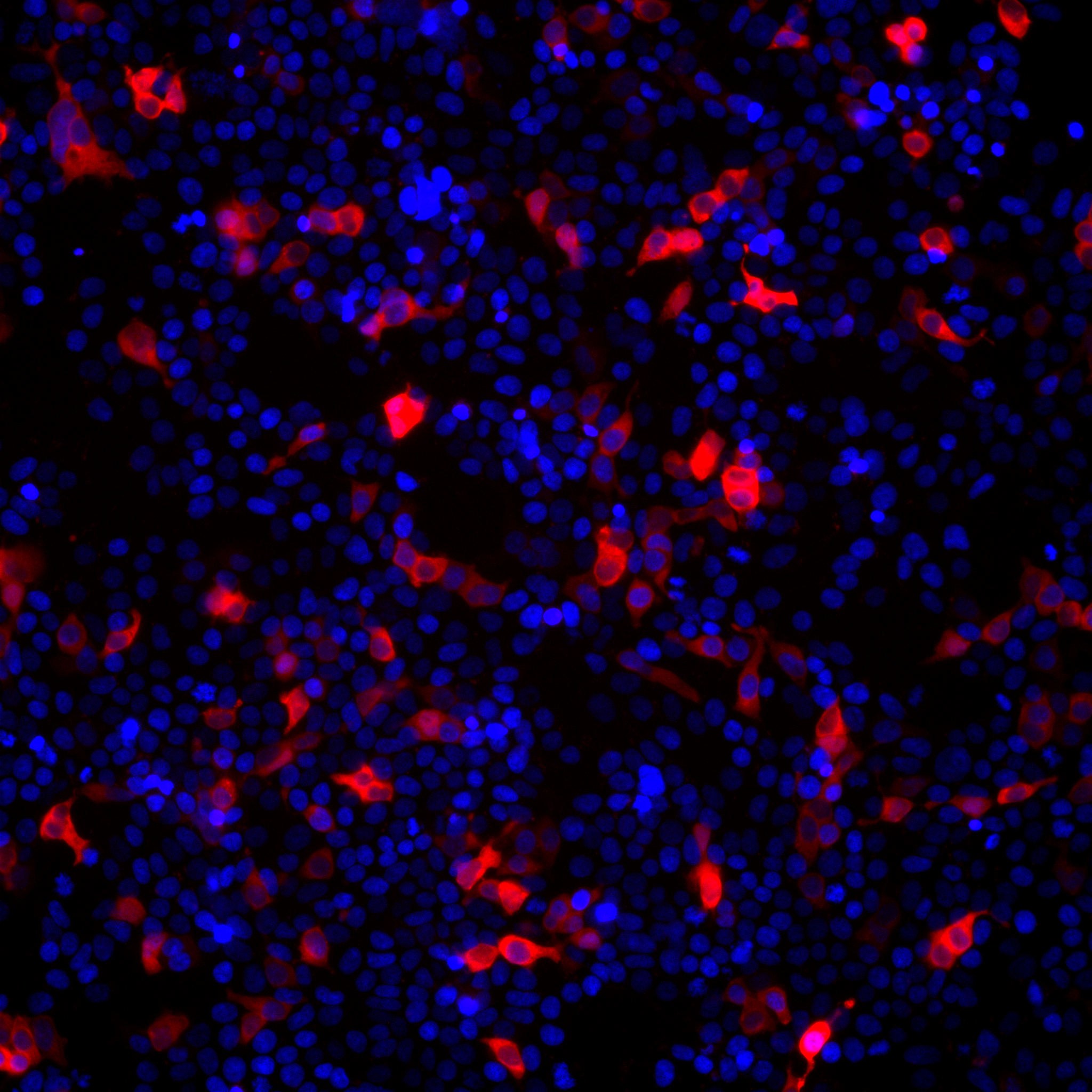

Supplement: Figure 5—source data 2. — Unlabeled wild-type (WT), catalytically inactive (C254A), or non-cleavable (D285A) caspase-11 constructs were transfected into HEK293T cells at increasing doses, together with a fixed dose of catalytically inactive (C254A) mCherry-tagged caspase-11. 18 hr following transfection, cells were imaged by fluorescence microscopy. Nuclei (blue) are stained with Hoechst. [file elife-83725-fig5-data2.zip › 0_C11(WT)_with_C254A-mCh.tif]

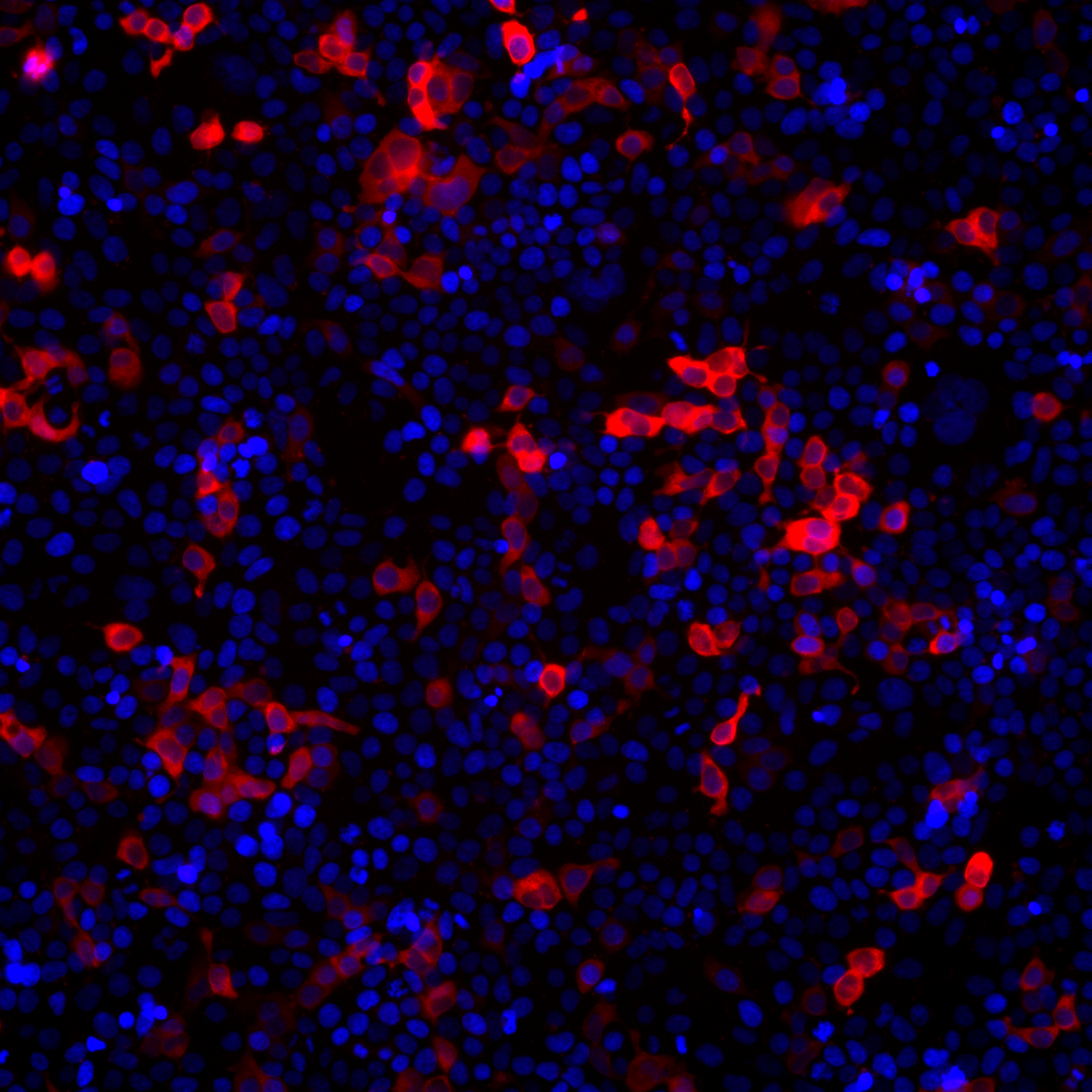

Supplement: Figure 5—source data 2. — Unlabeled wild-type (WT), catalytically inactive (C254A), or non-cleavable (D285A) caspase-11 constructs were transfected into HEK293T cells at increasing doses, together with a fixed dose of catalytically inactive (C254A) mCherry-tagged caspase-11. 18 hr following transfection, cells were imaged by fluorescence microscopy. Nuclei (blue) are stained with Hoechst. [file elife-83725-fig5-data2.zip › 0_C254A_with_C254A-mCh.tif]

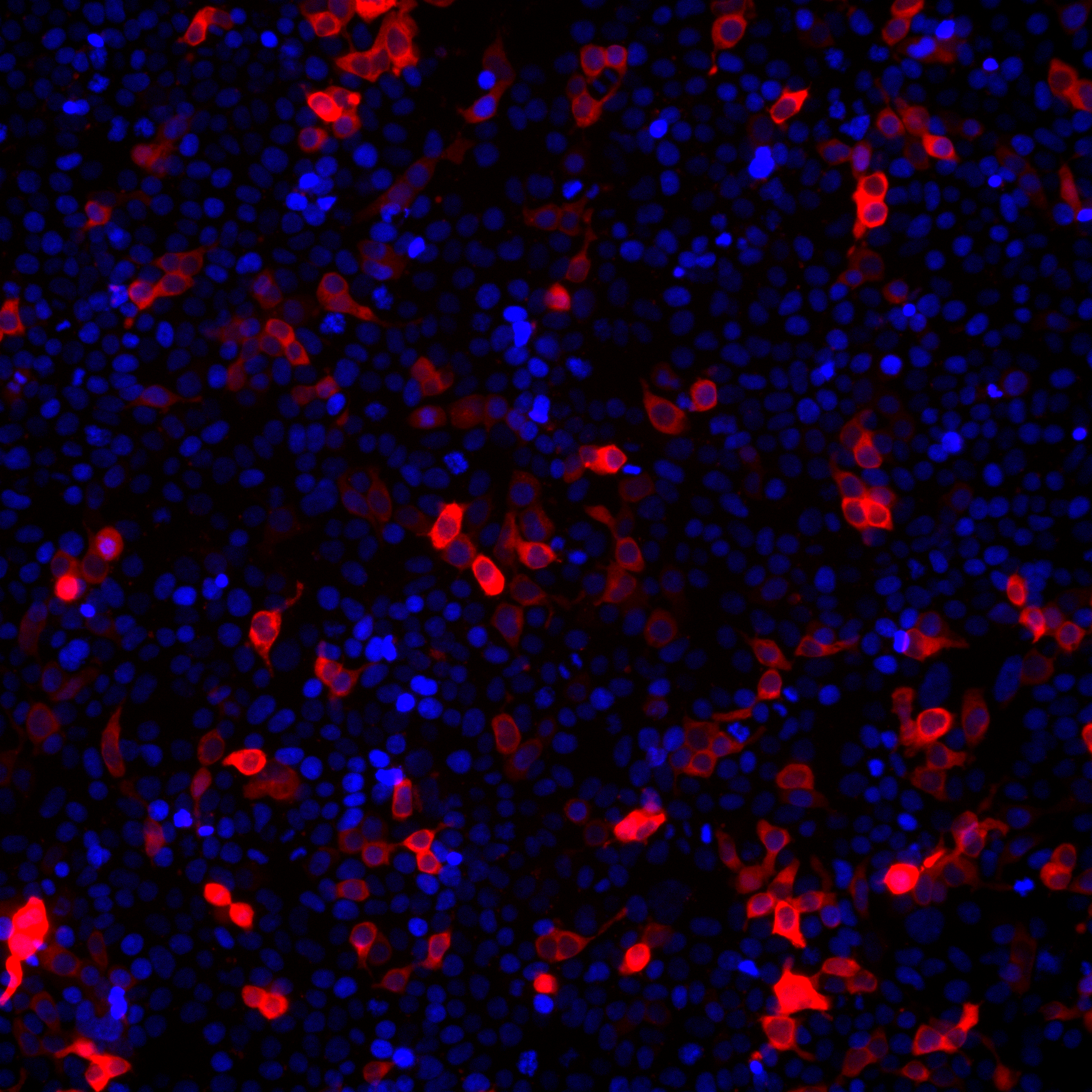

Supplement: Figure 5—source data 2. — Unlabeled wild-type (WT), catalytically inactive (C254A), or non-cleavable (D285A) caspase-11 constructs were transfected into HEK293T cells at increasing doses, together with a fixed dose of catalytically inactive (C254A) mCherry-tagged caspase-11. 18 hr following transfection, cells were imaged by fluorescence microscopy. Nuclei (blue) are stained with Hoechst. [file elife-83725-fig5-data2.zip › 0_D285A_with_C254A-mCh.tif]

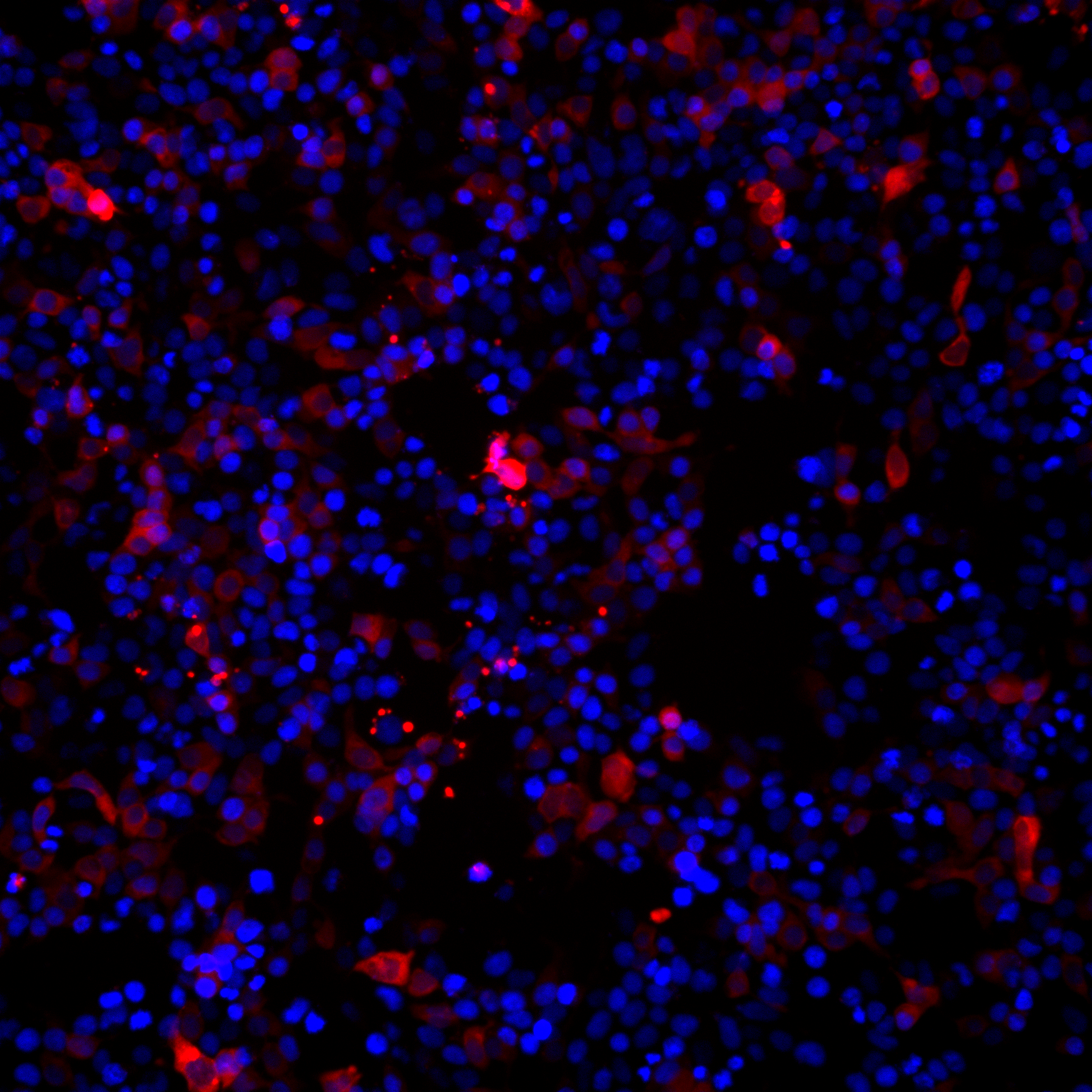

Supplement: Figure 5—source data 2. — Unlabeled wild-type (WT), catalytically inactive (C254A), or non-cleavable (D285A) caspase-11 constructs were transfected into HEK293T cells at increasing doses, together with a fixed dose of catalytically inactive (C254A) mCherry-tagged caspase-11. 18 hr following transfection, cells were imaged by fluorescence microscopy. Nuclei (blue) are stained with Hoechst. [file elife-83725-fig5-data2.zip › 500_C11(WT)_with_C254A-mCh.tif]

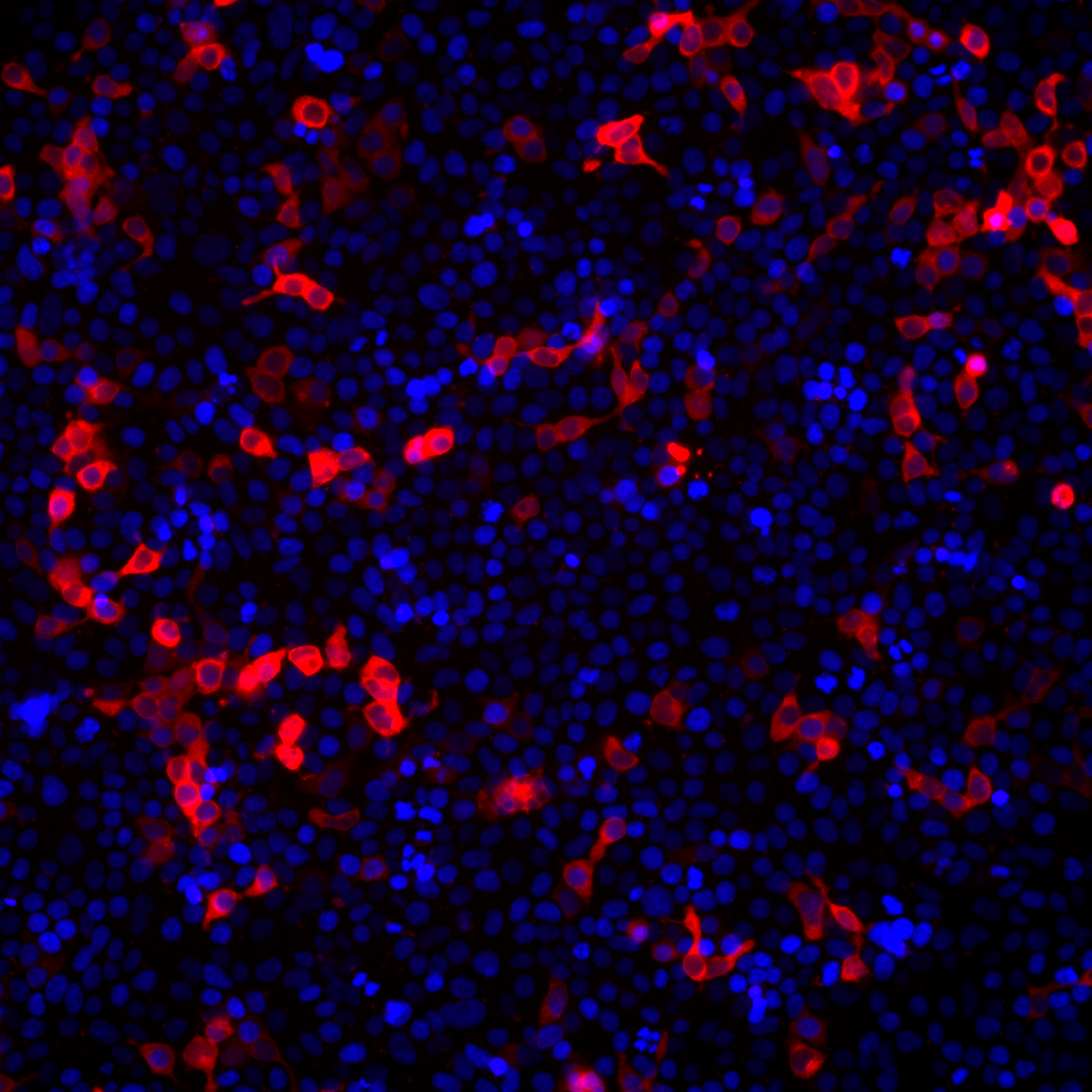

Supplement: Figure 5—source data 2. — Unlabeled wild-type (WT), catalytically inactive (C254A), or non-cleavable (D285A) caspase-11 constructs were transfected into HEK293T cells at increasing doses, together with a fixed dose of catalytically inactive (C254A) mCherry-tagged caspase-11. 18 hr following transfection, cells were imaged by fluorescence microscopy. Nuclei (blue) are stained with Hoechst. [file elife-83725-fig5-data2.zip › 500_C254A_with_C254A-mCh.tif]

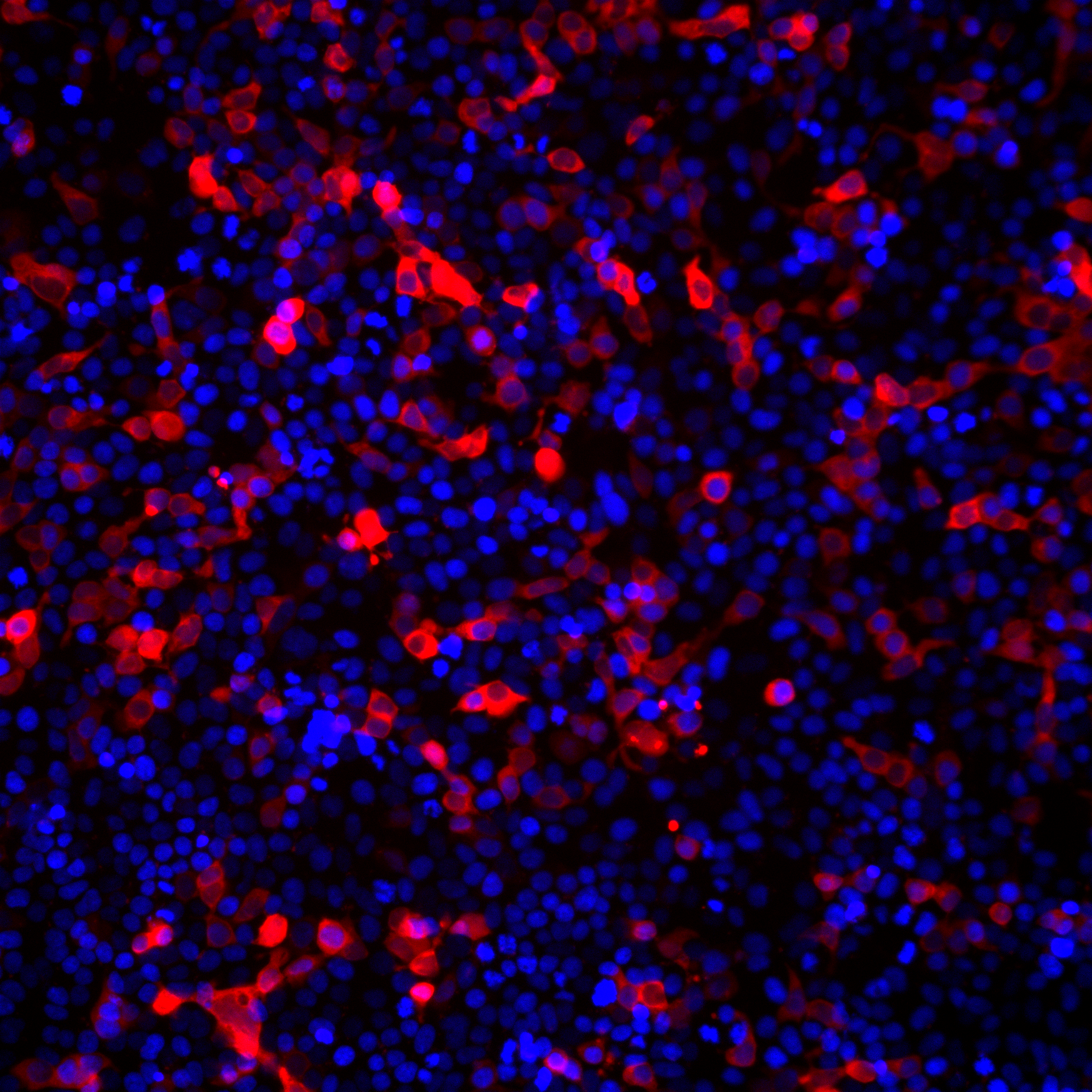

Supplement: Figure 5—source data 2. — Unlabeled wild-type (WT), catalytically inactive (C254A), or non-cleavable (D285A) caspase-11 constructs were transfected into HEK293T cells at increasing doses, together with a fixed dose of catalytically inactive (C254A) mCherry-tagged caspase-11. 18 hr following transfection, cells were imaged by fluorescence microscopy. Nuclei (blue) are stained with Hoechst. [file elife-83725-fig5-data2.zip › 500_D285A_with_C254A-mCh.tif]

Fig 5-Source Data 2 (5C)

*CASP11*:

WT

C254A

D285A

0 ng

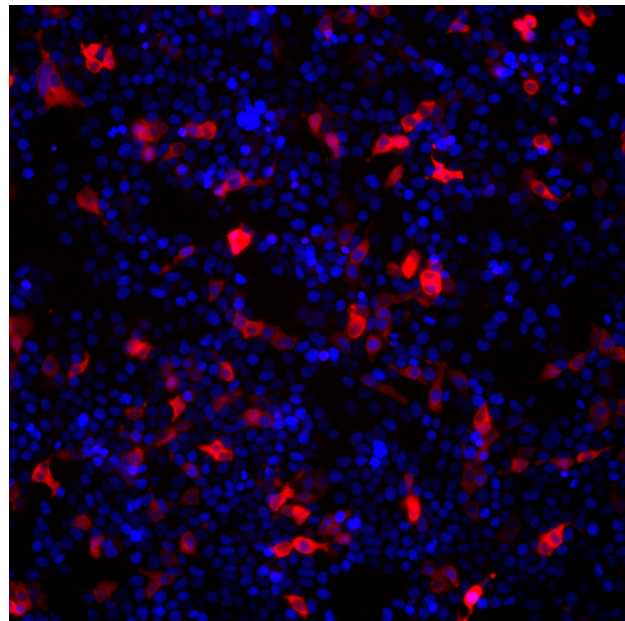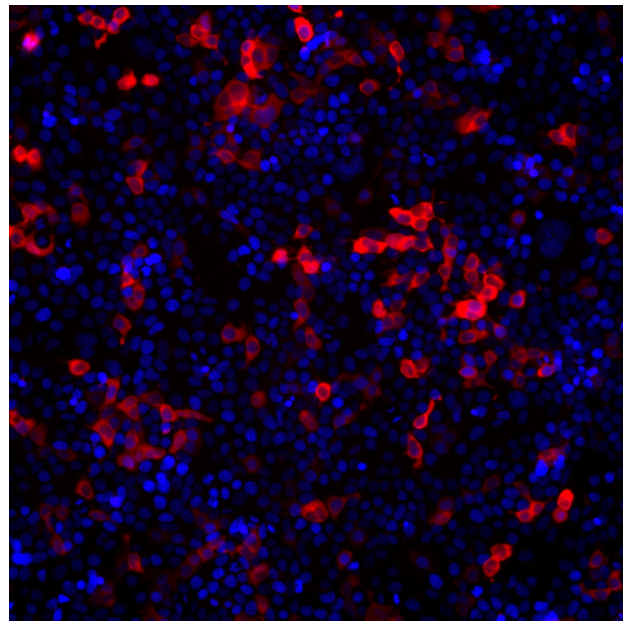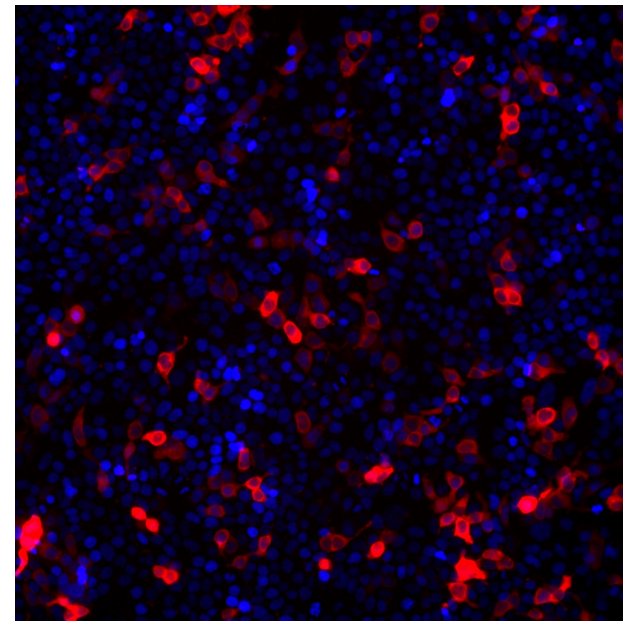

[*CASP11*(C254A)-mCh]

500 ng

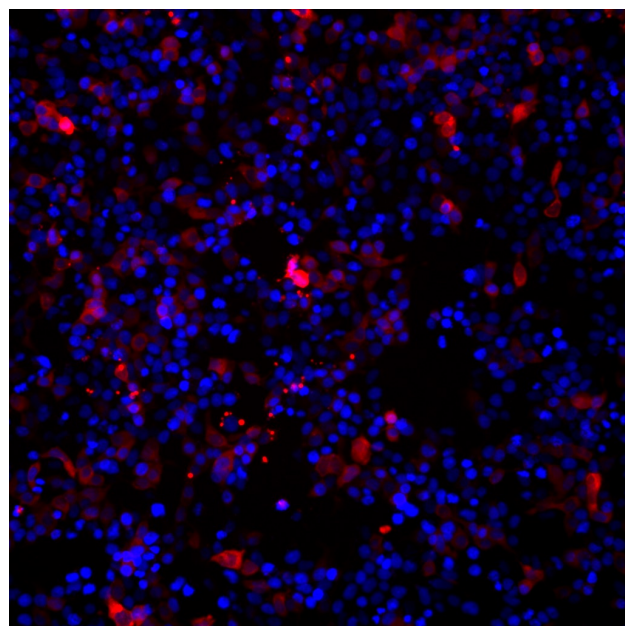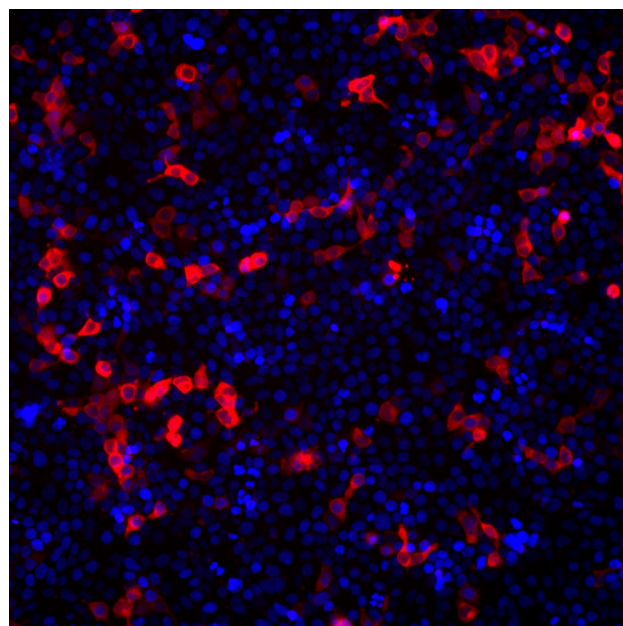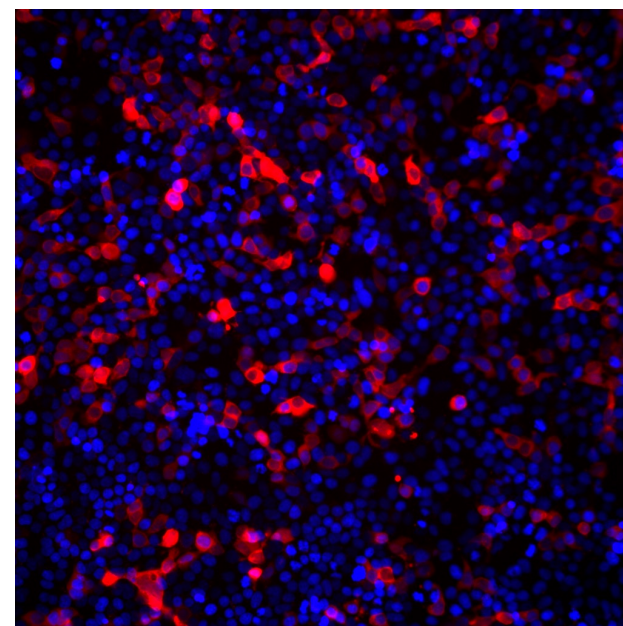

HOECHST mCHERRY

Supplement: Figure 5—source data 2. — Unlabeled wild-type (WT), catalytically inactive (C254A), or non-cleavable (D285A) caspase-11 constructs were transfected into HEK293T cells at increasing doses, together with a fixed dose of catalytically inactive (C254A) mCherry-tagged caspase-11. 18 hr following transfection, cells were imaged by fluorescence microscopy. Nuclei (blue) are stained with Hoechst. [file elife-83725-fig5-data2.zip › Figure 5-source data 2.pdf]

Fig 6-Source Data 1 (6B)

DmrB( $\Delta$ CARD)-CASP11-mCh:

WT

C254A

-AP20187

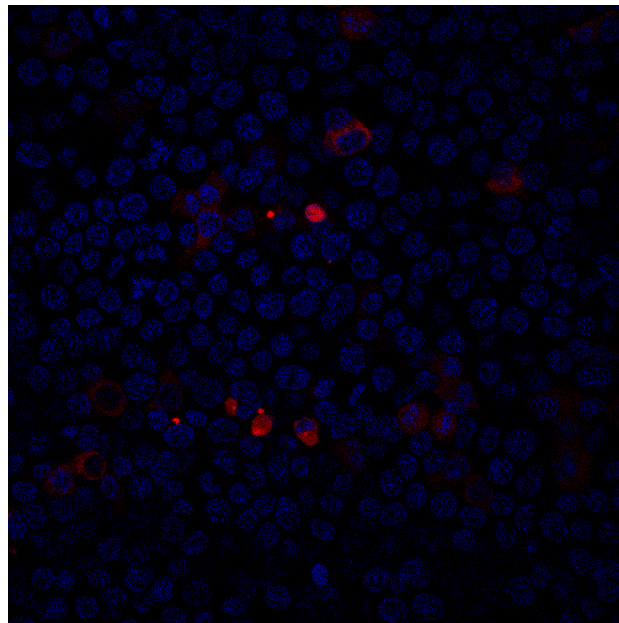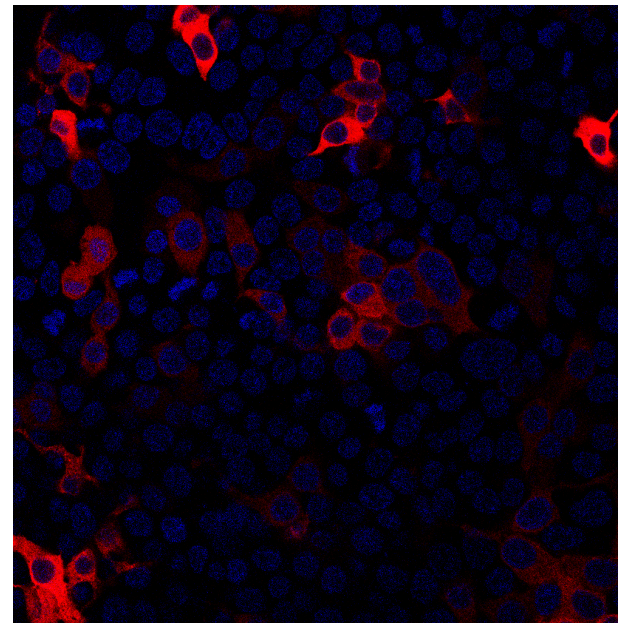

+AP20187

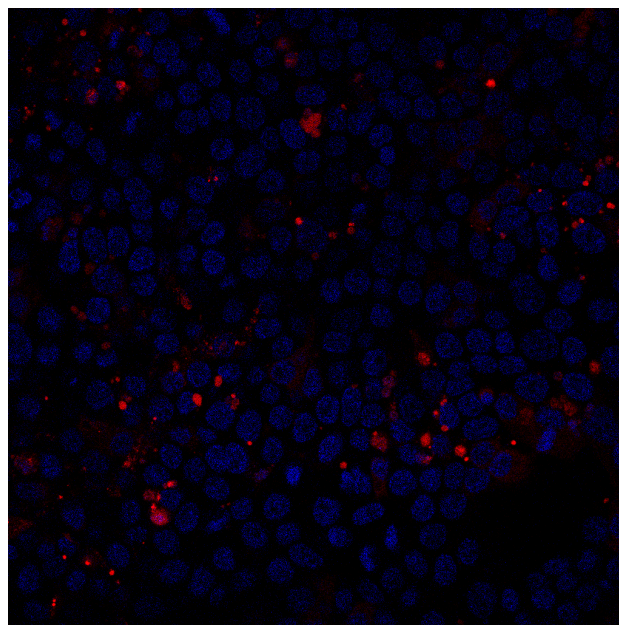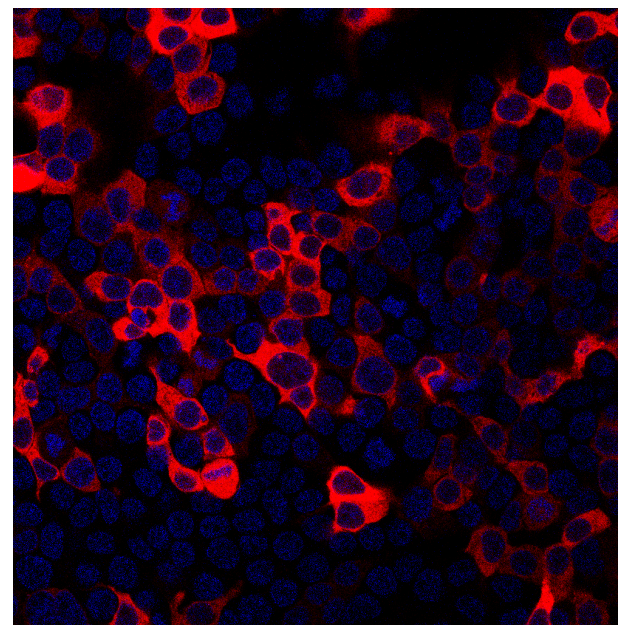

HOECHST mCHERRY

Supplement: Figure 6—source data 1. — HEK293T cells were transfected with WT or catalytically inactive (C254A) DmrB-(ΔCARD)-Casp11-FLAG-mCherry constructs. 24 hr post-transfection, cells were incubated with AP20187 (1 μM) for 6 hr and imaged by confocal microscopy. Nuclei (blue) are stained with Hoechst. [file elife-83725-fig6-data1.zip › Figure 6-source data 1.pdf]

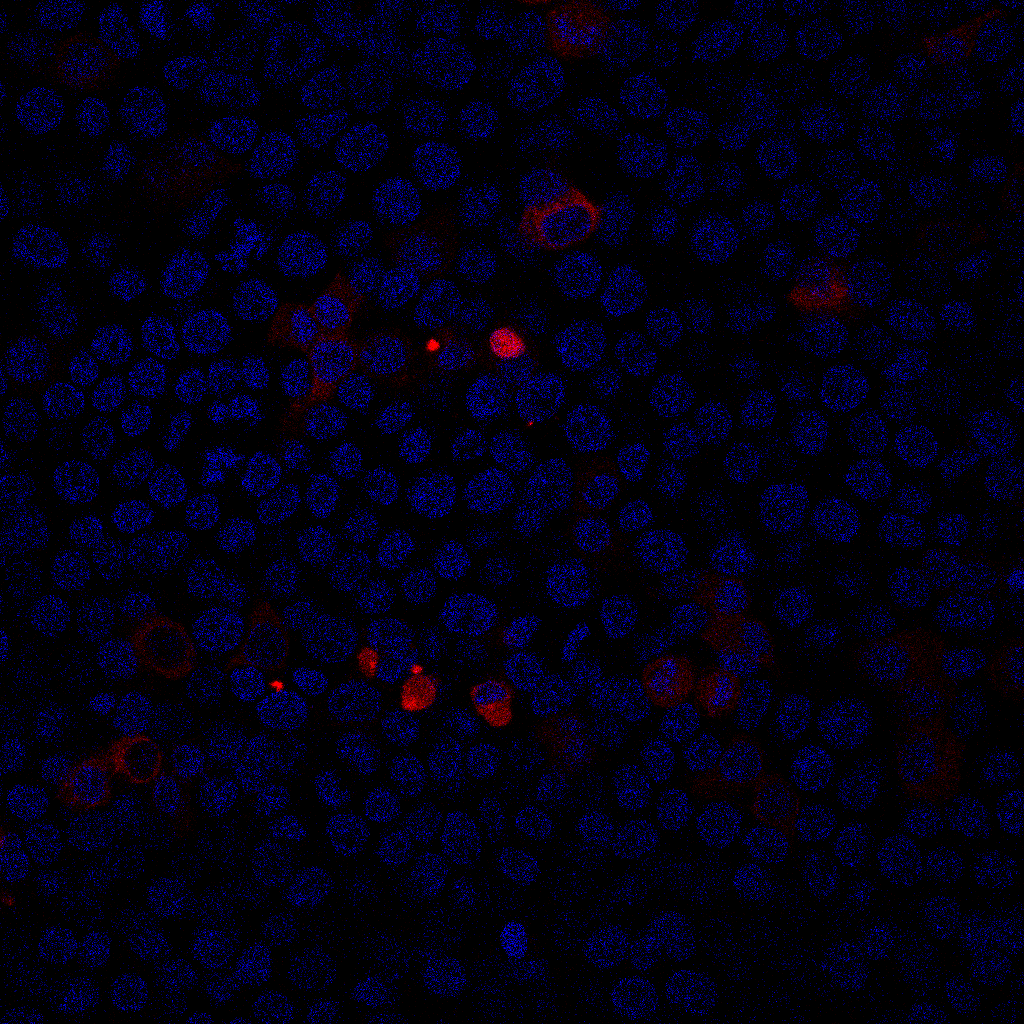

Supplement: Figure 6—source data 1. — HEK293T cells were transfected with WT or catalytically inactive (C254A) DmrB-(ΔCARD)-Casp11-FLAG-mCherry constructs. 24 hr post-transfection, cells were incubated with AP20187 (1 μM) for 6 hr and imaged by confocal microscopy. Nuclei (blue) are stained with Hoechst. [file elife-83725-fig6-data1.zip › No_Dimerizer_DmrB-(╬öCARD)-C11WT-mCh.tif]

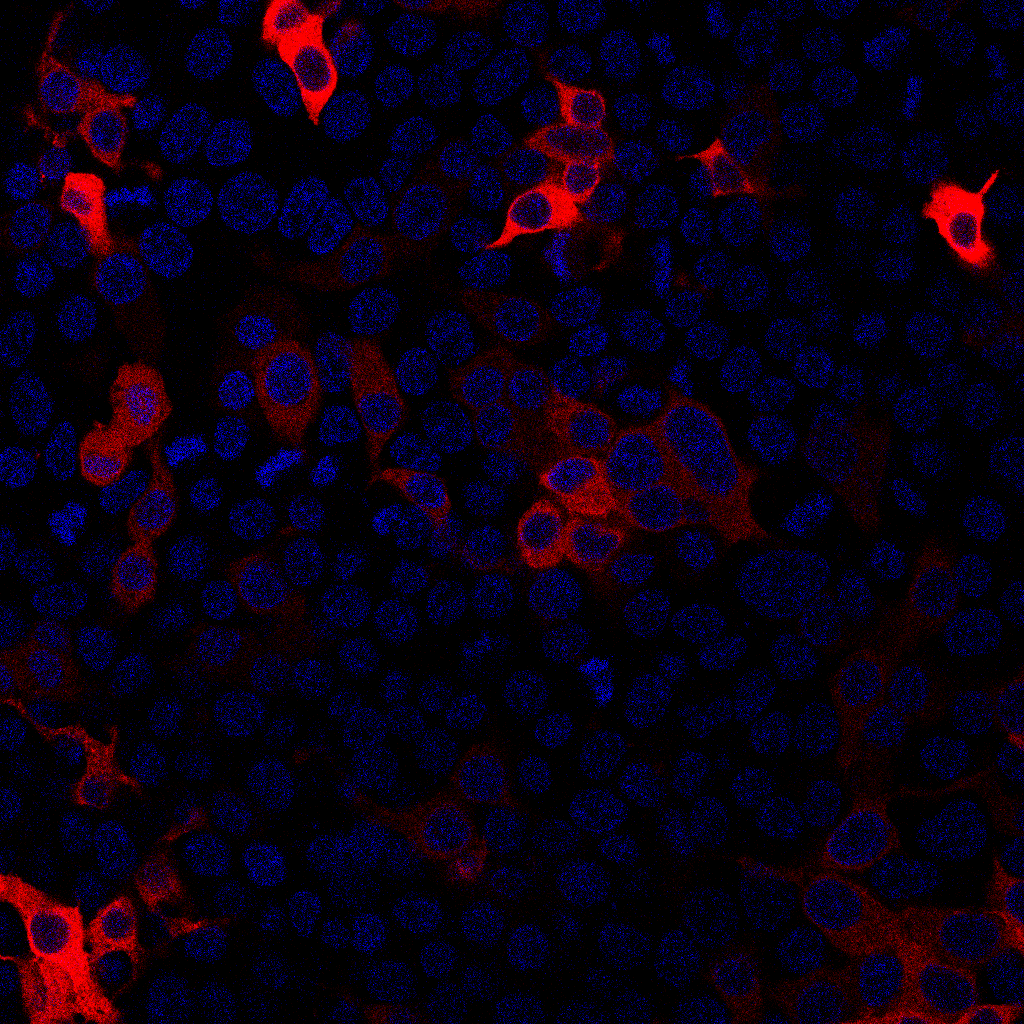

Supplement: Figure 6—source data 1. — HEK293T cells were transfected with WT or catalytically inactive (C254A) DmrB-(ΔCARD)-Casp11-FLAG-mCherry constructs. 24 hr post-transfection, cells were incubated with AP20187 (1 μM) for 6 hr and imaged by confocal microscopy. Nuclei (blue) are stained with Hoechst. [file elife-83725-fig6-data1.zip › No_Dimerizer_DmrB-(╬öCARD)-C254A-mCh.tif]

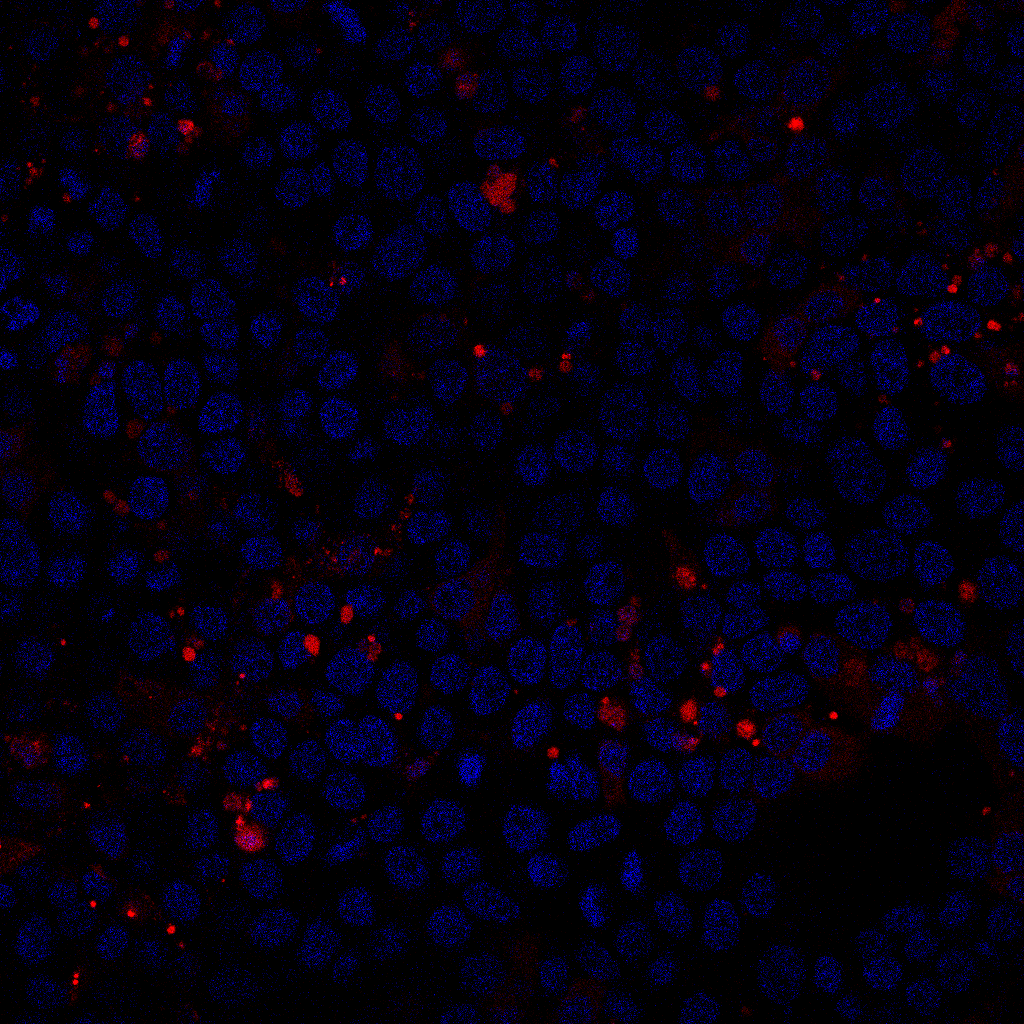

Supplement: Figure 6—source data 1. — HEK293T cells were transfected with WT or catalytically inactive (C254A) DmrB-(ΔCARD)-Casp11-FLAG-mCherry constructs. 24 hr post-transfection, cells were incubated with AP20187 (1 μM) for 6 hr and imaged by confocal microscopy. Nuclei (blue) are stained with Hoechst. [file elife-83725-fig6-data1.zip › Yes_Dimerizer_DmrB-(╬öCARD)-C11WT-mCh.tif]

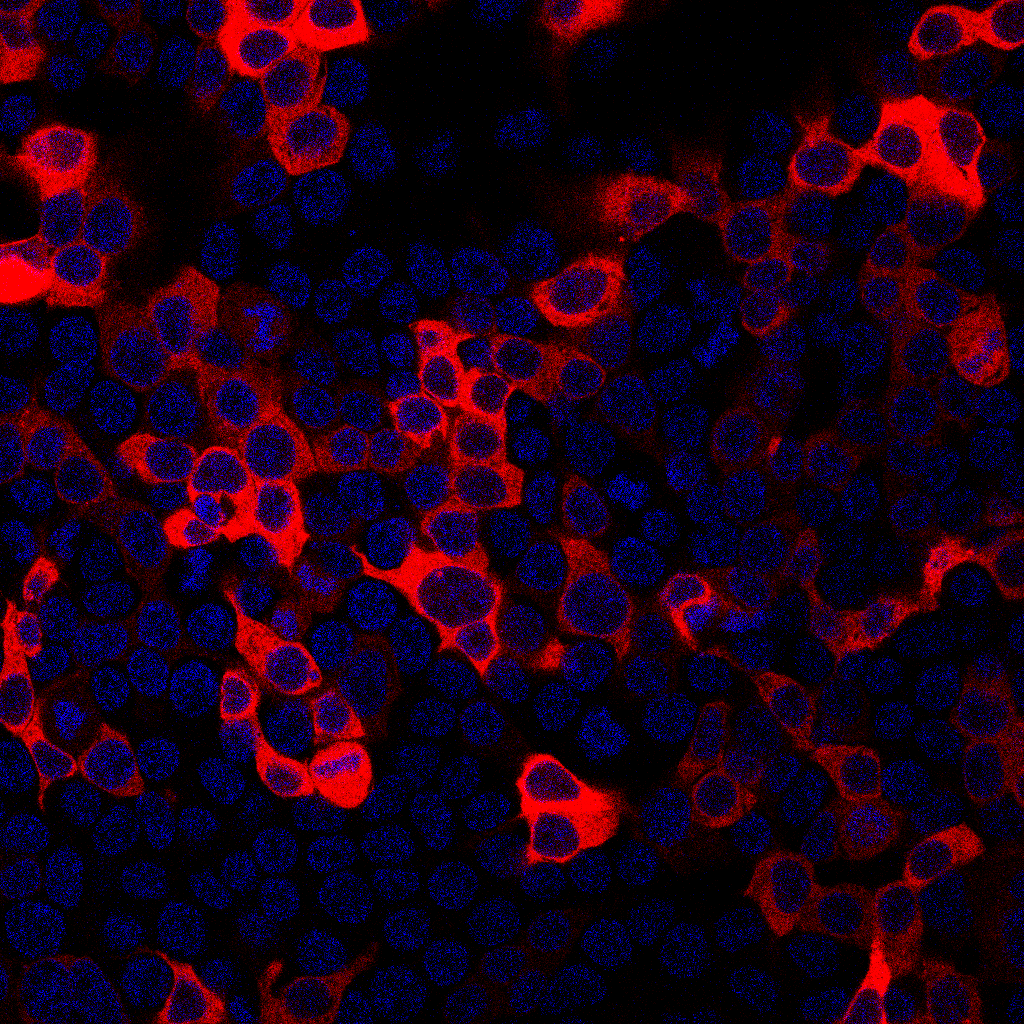

Supplement: Figure 6—source data 1. — HEK293T cells were transfected with WT or catalytically inactive (C254A) DmrB-(ΔCARD)-Casp11-FLAG-mCherry constructs. 24 hr post-transfection, cells were incubated with AP20187 (1 μM) for 6 hr and imaged by confocal microscopy. Nuclei (blue) are stained with Hoechst. [file elife-83725-fig6-data1.zip › Yes_Dimerizer_DmrB-(╬öCARD)-C254A-mCh.tif]

ePE42

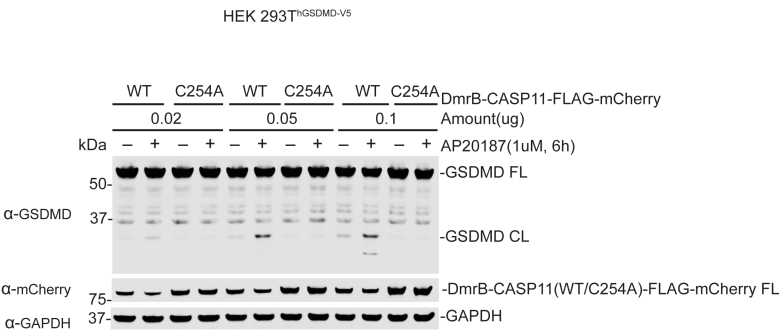

Supplement: Figure 6—source data 2. — HEK293T cells stably expressing human gasdermin D (HEK293ThGSDMD) were transfected with WT or catalytically inactive (C254A) DmrB-(ΔCARD)-Casp11-FLAG-mCherry constructs, and incubated in AP20187 (1 μM) for 6 hr. Lysates were harvested and immunoblotted for GSDMD and mCherry, with GAPDH as loading control. [file elife-83725-fig6-data2.zip › Figure 6-source data 2.pdf]

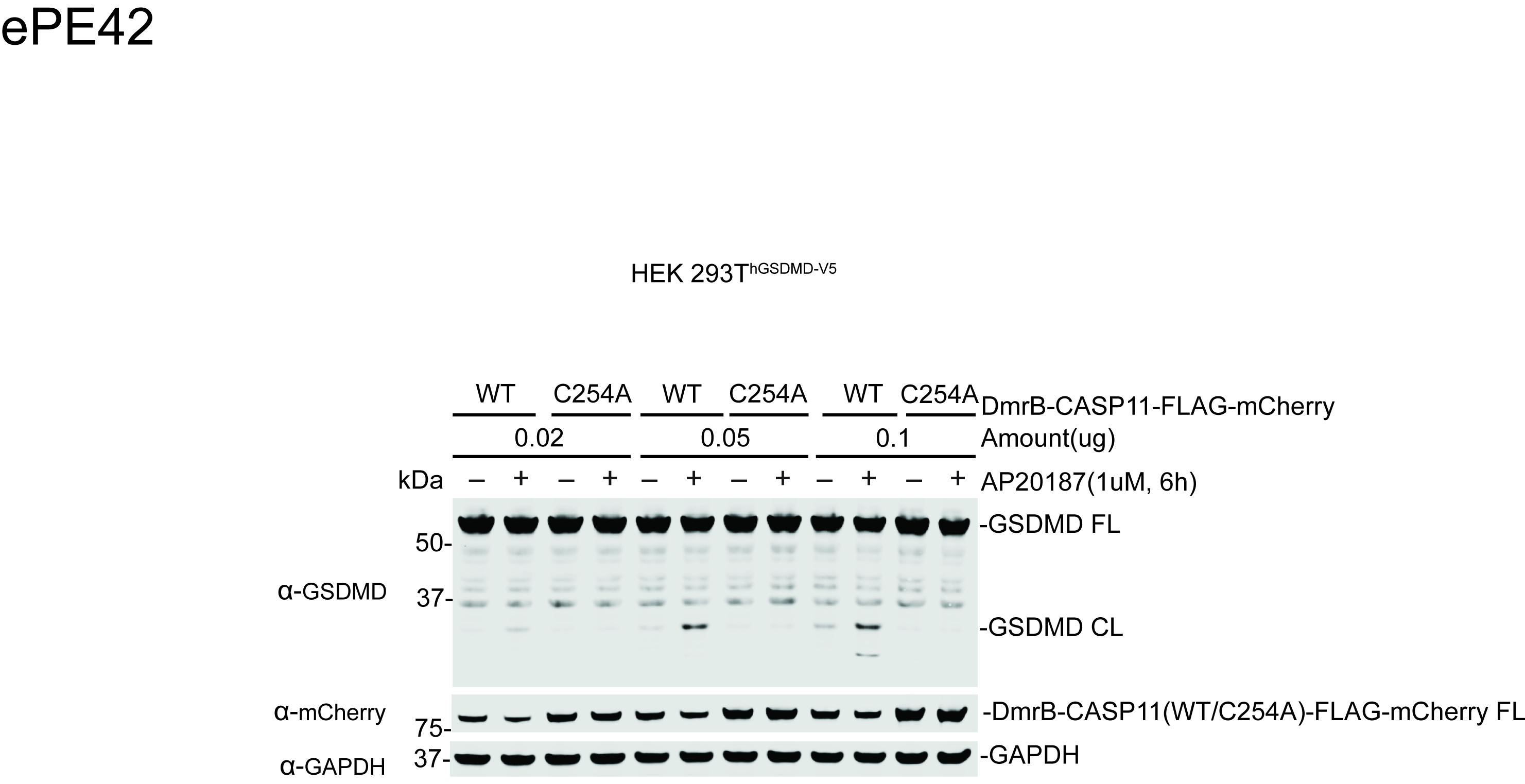

Supplement: Figure 6—source data 2. — HEK293T cells stably expressing human gasdermin D (HEK293ThGSDMD) were transfected with WT or catalytically inactive (C254A) DmrB-(ΔCARD)-Casp11-FLAG-mCherry constructs, and incubated in AP20187 (1 μM) for 6 hr. Lysates were harvested and immunoblotted for GSDMD and mCherry, with GAPDH as loading control. [file elife-83725-fig6-data2.zip › Figure_6D_labeled.tif]

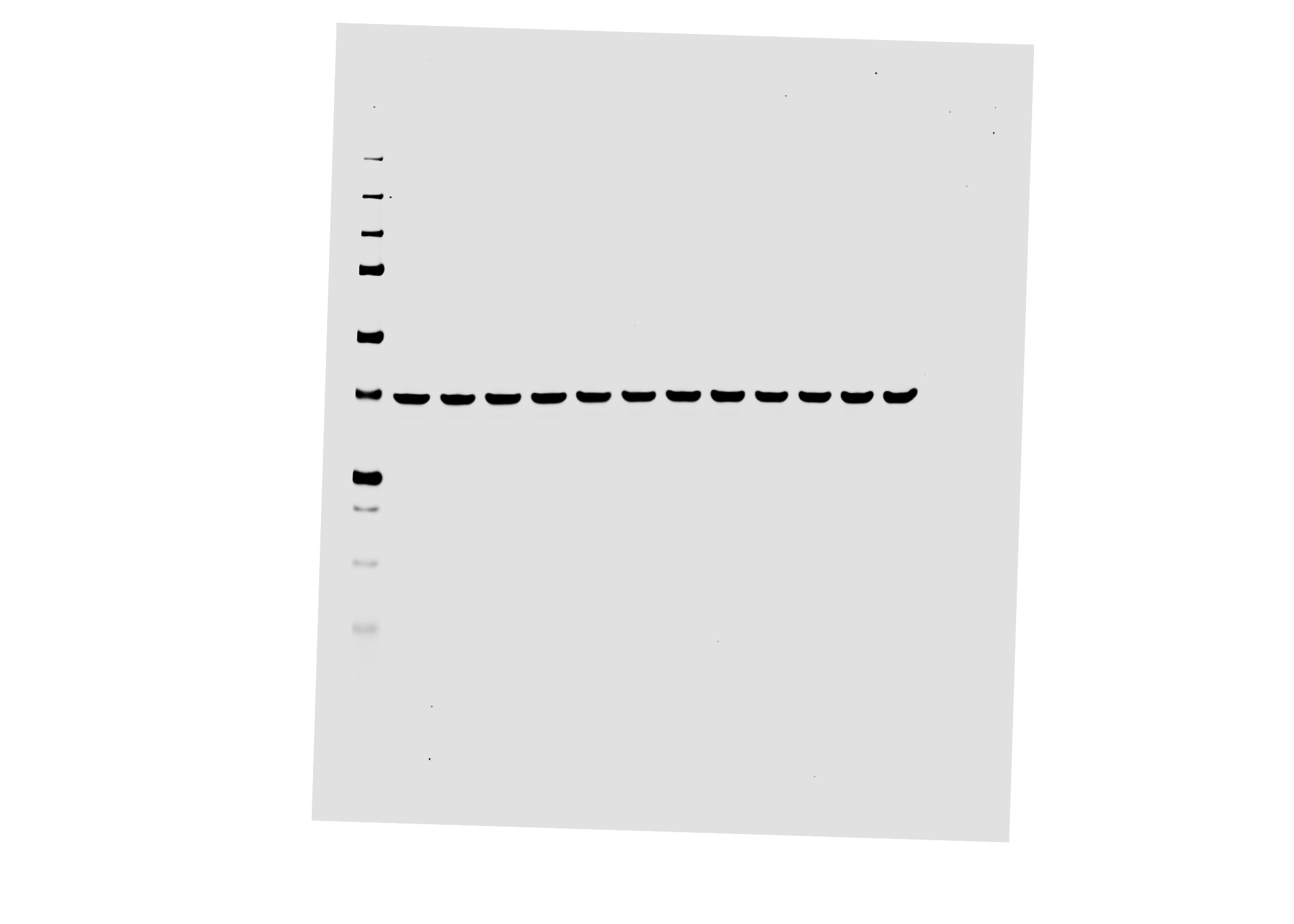

Supplement: Figure 6—source data 2. — HEK293T cells stably expressing human gasdermin D (HEK293ThGSDMD) were transfected with WT or catalytically inactive (C254A) DmrB-(ΔCARD)-Casp11-FLAG-mCherry constructs, and incubated in AP20187 (1 μM) for 6 hr. Lysates were harvested and immunoblotted for GSDMD and mCherry, with GAPDH as loading control. [file elife-83725-fig6-data2.zip › GAPDH.tif]

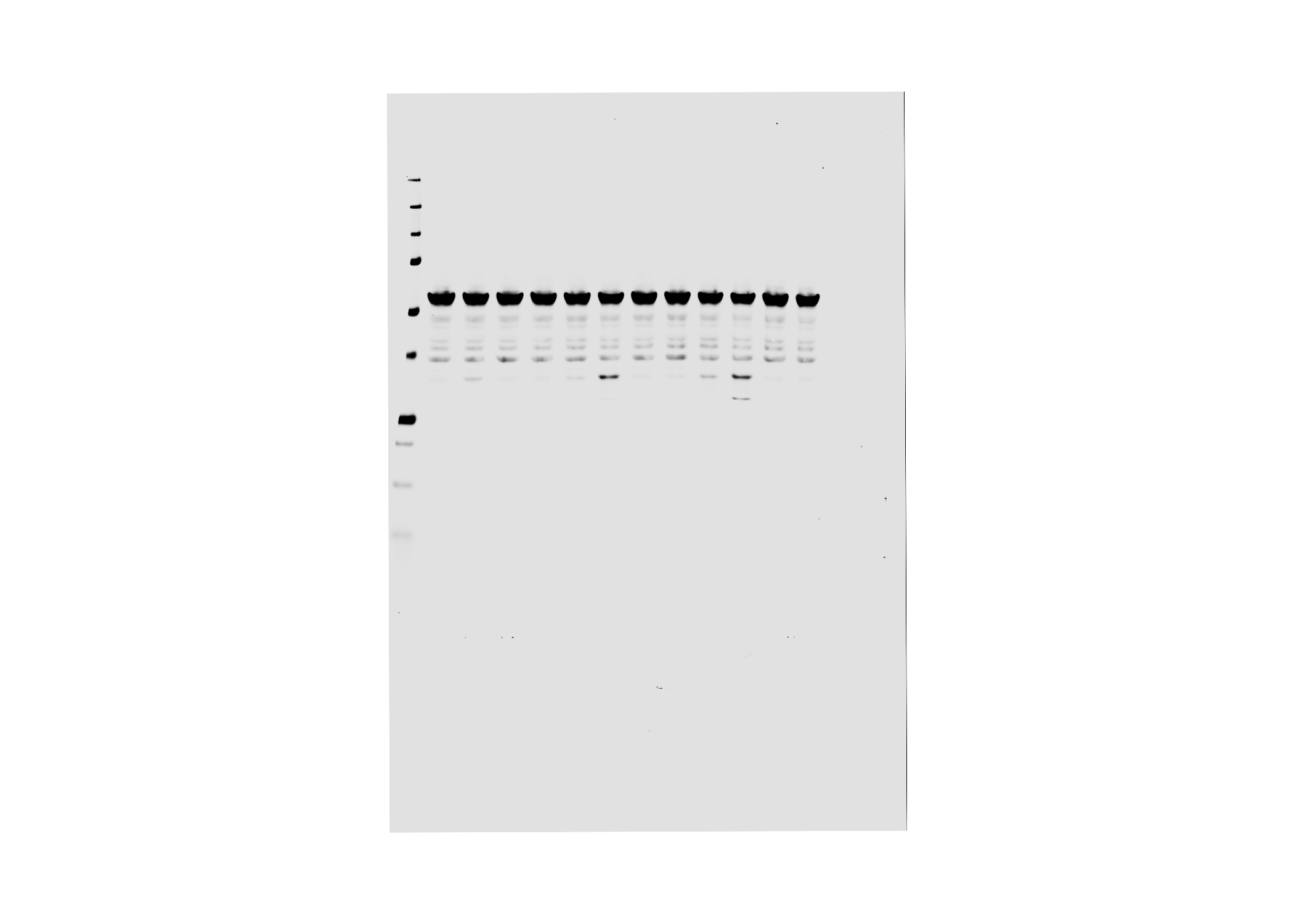

Supplement: Figure 6—source data 2. — HEK293T cells stably expressing human gasdermin D (HEK293ThGSDMD) were transfected with WT or catalytically inactive (C254A) DmrB-(ΔCARD)-Casp11-FLAG-mCherry constructs, and incubated in AP20187 (1 μM) for 6 hr. Lysates were harvested and immunoblotted for GSDMD and mCherry, with GAPDH as loading control. [file elife-83725-fig6-data2.zip › GSDMD.tif]

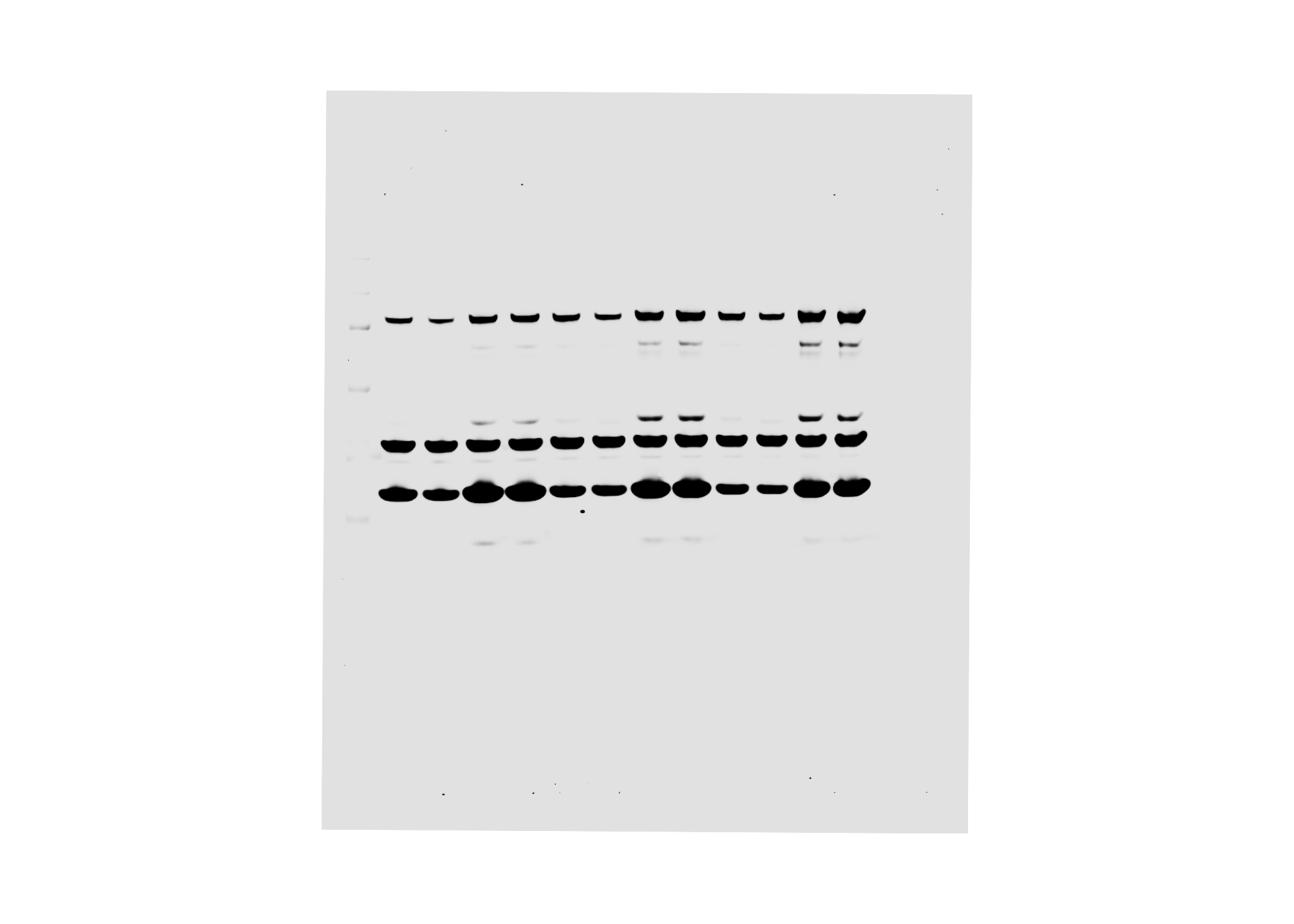

Supplement: Figure 6—source data 2. — HEK293T cells stably expressing human gasdermin D (HEK293ThGSDMD) were transfected with WT or catalytically inactive (C254A) DmrB-(ΔCARD)-Casp11-FLAG-mCherry constructs, and incubated in AP20187 (1 μM) for 6 hr. Lysates were harvested and immunoblotted for GSDMD and mCherry, with GAPDH as loading control. [file elife-83725-fig6-data2.zip › mcherry.tif]

# ePE44(Total Western)

HEK 293T<sup>PARENTAL</sup>

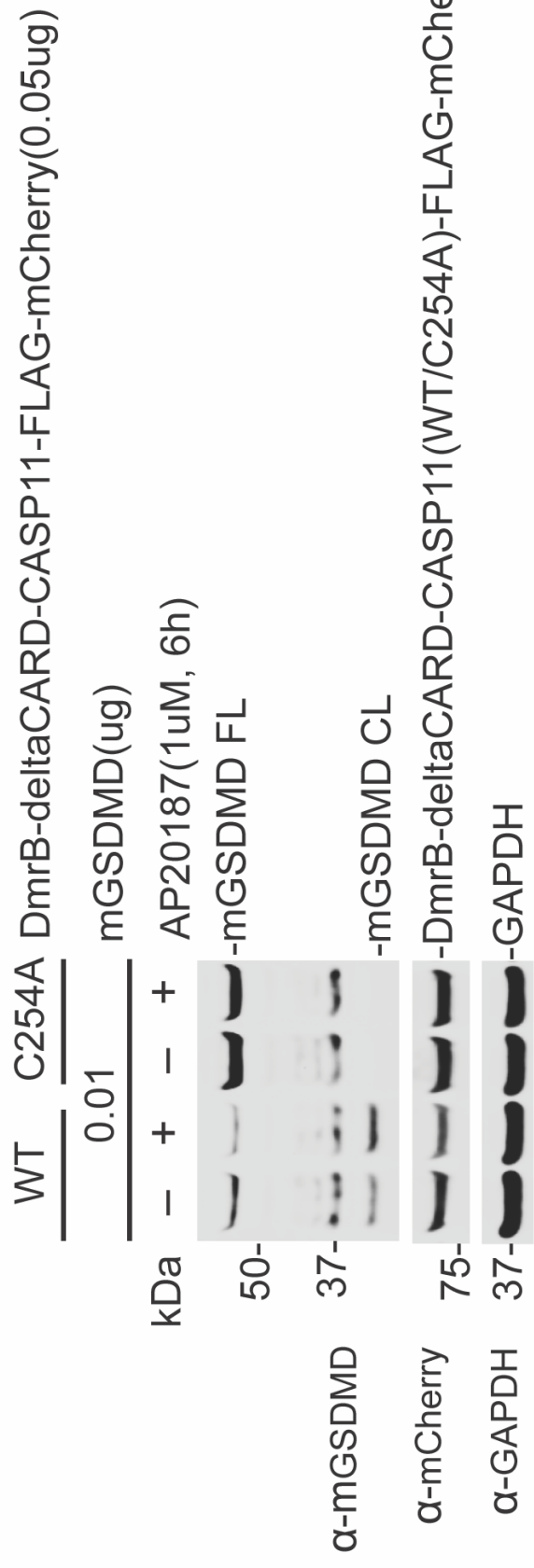

Supplement: Figure 6—figure supplement 1—source data 1. — HEK293T cells were transiently co-transfected with ΔCARD DmrB-Casp11-FLAG-mCherry constructs (0.1 μg; Figure 4A) and murine gasdermin D (GSDMD, 0.01 μg) for 24 hr before the addition of AP20187 (1 μM) for 6 hr. Casp11 protein expression and cytotoxicity were determined by immunoblotting for mCherry and GSDMD in pooled lysates and supernatants. GAPDH is indicated as loading control. FL = full-length; CL = cleaved. [file elife-83725-fig6-figsupp1-data1.zip › Figure 6-figure supplement 1-source data 1.pdf]

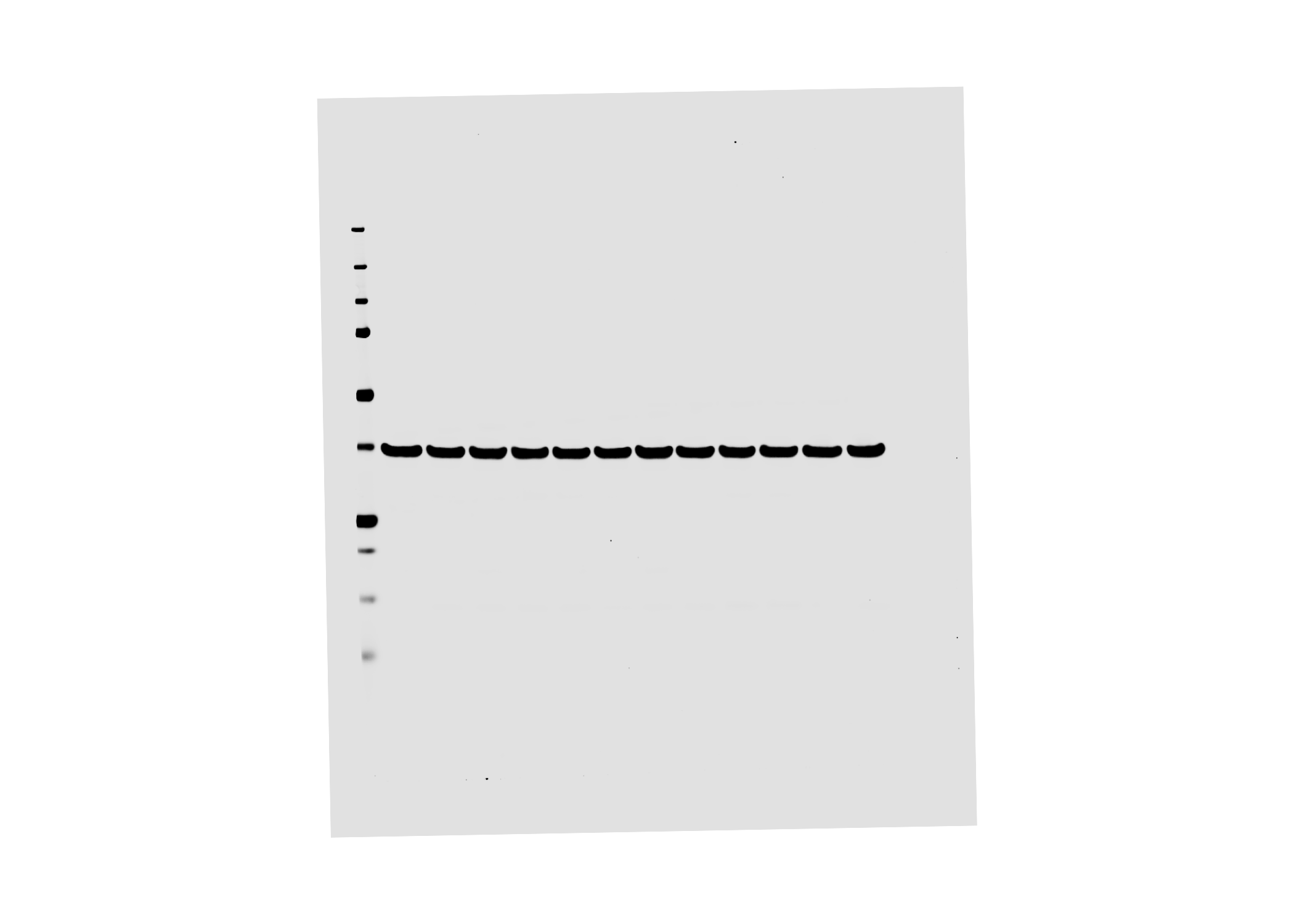

Supplement: Figure 6—figure supplement 1—source data 1. — HEK293T cells were transiently co-transfected with ΔCARD DmrB-Casp11-FLAG-mCherry constructs (0.1 μg; Figure 4A) and murine gasdermin D (GSDMD, 0.01 μg) for 24 hr before the addition of AP20187 (1 μM) for 6 hr. Casp11 protein expression and cytotoxicity were determined by immunoblotting for mCherry and GSDMD in pooled lysates and supernatants. GAPDH is indicated as loading control. FL = full-length; CL = cleaved. [file elife-83725-fig6-figsupp1-data1.zip › GAPDH.tiff]

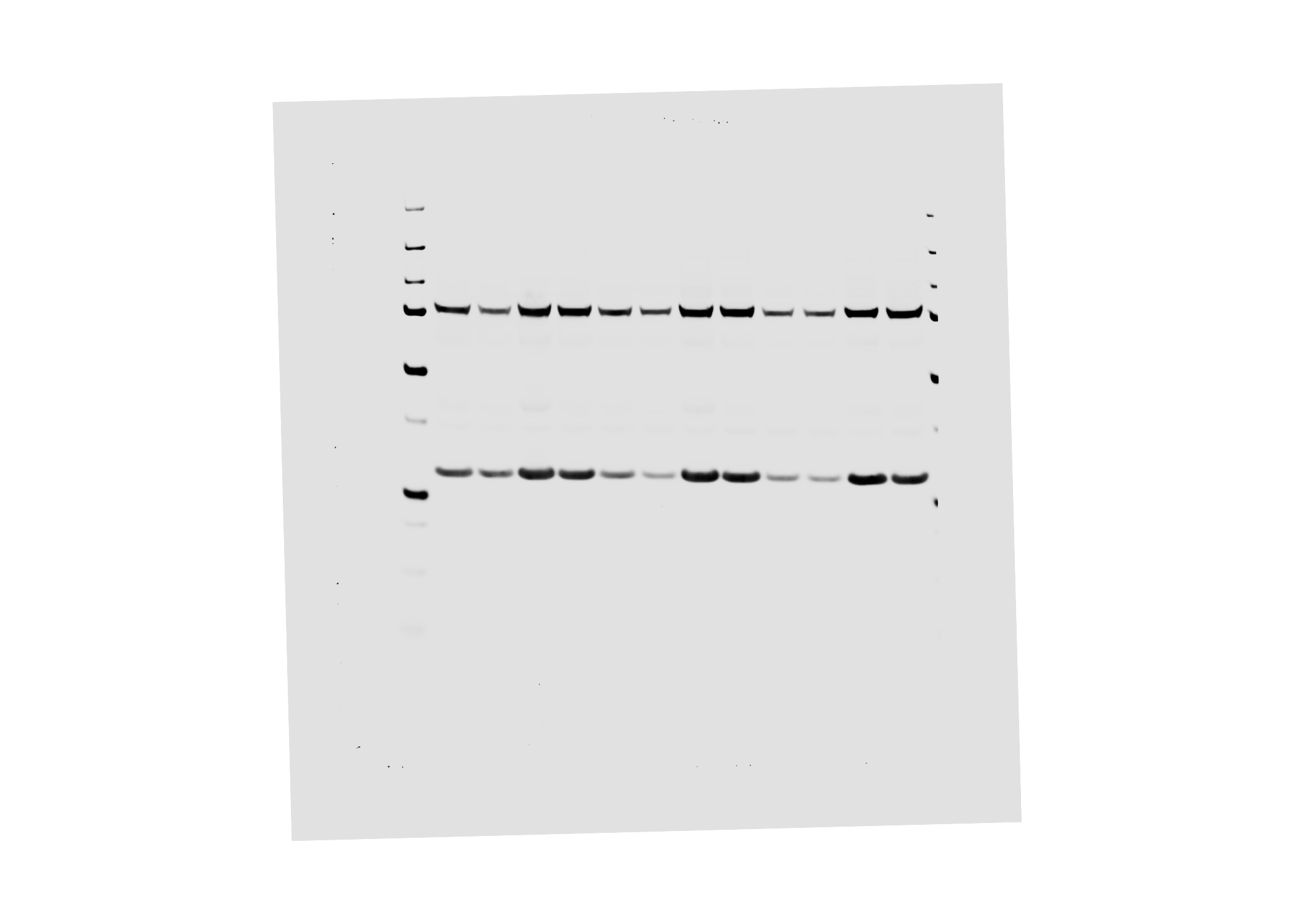

Supplement: Figure 6—figure supplement 1—source data 1. — HEK293T cells were transiently co-transfected with ΔCARD DmrB-Casp11-FLAG-mCherry constructs (0.1 μg; Figure 4A) and murine gasdermin D (GSDMD, 0.01 μg) for 24 hr before the addition of AP20187 (1 μM) for 6 hr. Casp11 protein expression and cytotoxicity were determined by immunoblotting for mCherry and GSDMD in pooled lysates and supernatants. GAPDH is indicated as loading control. FL = full-length; CL = cleaved. [file elife-83725-fig6-figsupp1-data1.zip › mCherry.tiff]

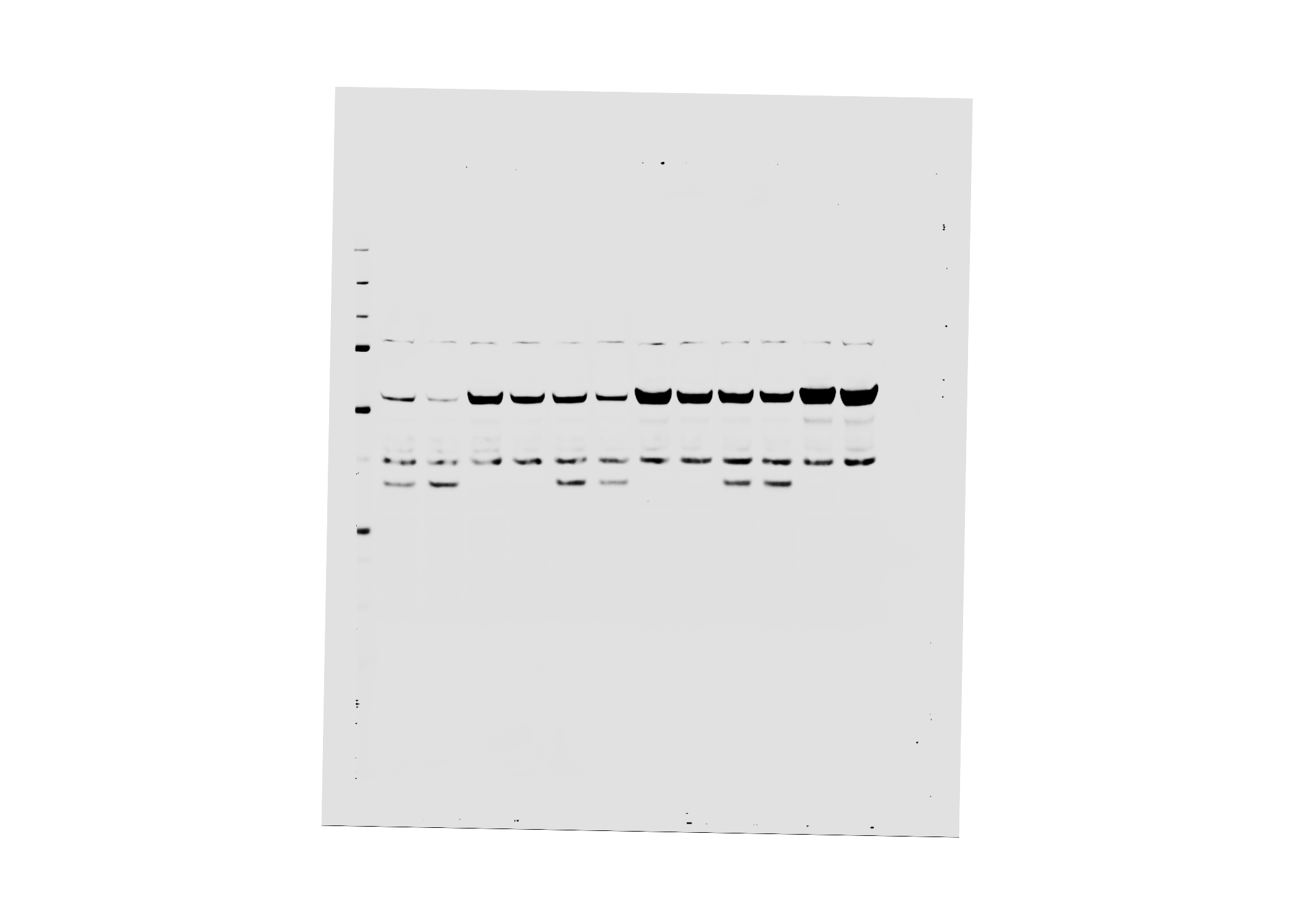

Supplement: Figure 6—figure supplement 1—source data 1. — HEK293T cells were transiently co-transfected with ΔCARD DmrB-Casp11-FLAG-mCherry constructs (0.1 μg; Figure 4A) and murine gasdermin D (GSDMD, 0.01 μg) for 24 hr before the addition of AP20187 (1 μM) for 6 hr. Casp11 protein expression and cytotoxicity were determined by immunoblotting for mCherry and GSDMD in pooled lysates and supernatants. GAPDH is indicated as loading control. FL = full-length; CL = cleaved. [file elife-83725-fig6-figsupp1-data1.zip › mGSDMD.tiff]

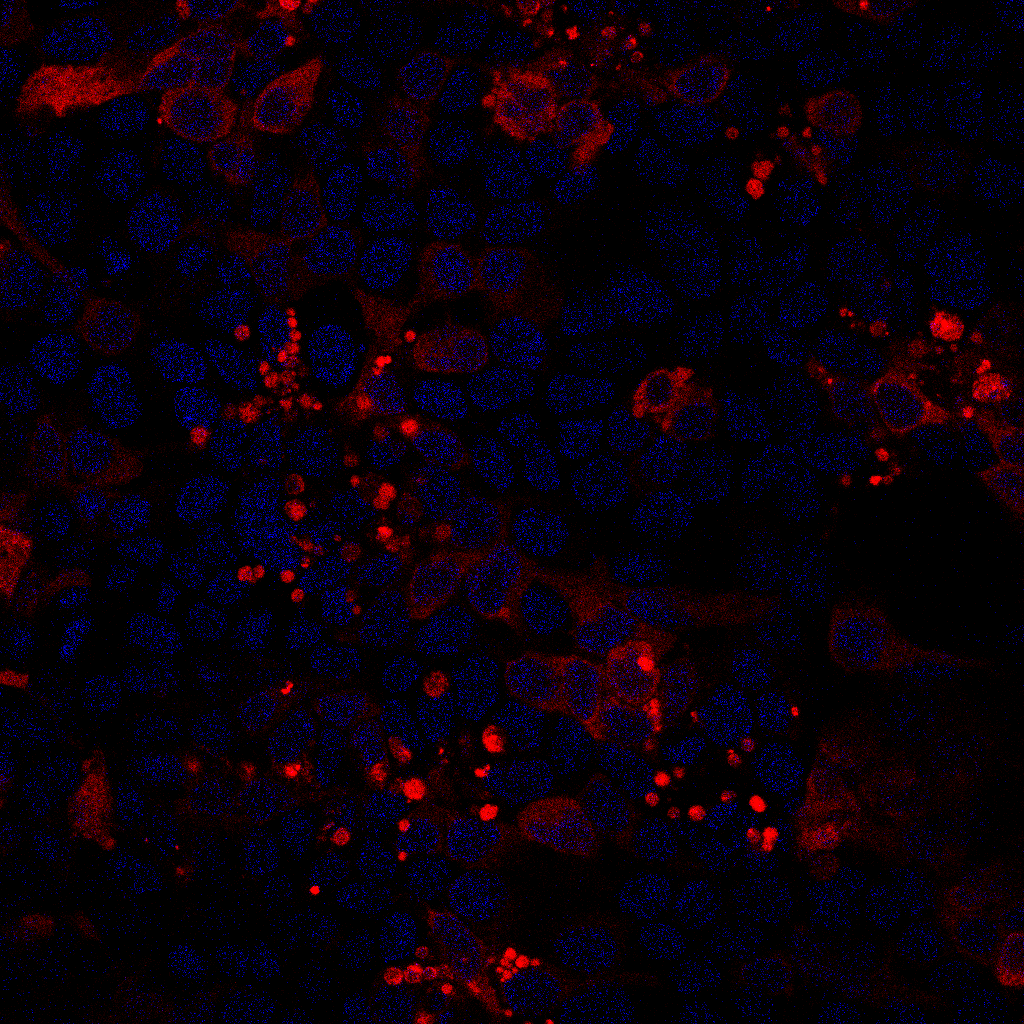

Supplement: Figure 6—figure supplement 2—source data 1. — HEK293T cells were transfected with indicated Casp11-mCherry constructs. 18 hr post-transfection, cells were fixed and prepared for microscopy. Nuclei were stained with Hoechst. [file elife-83725-fig6-figsupp2-data1.zip › (╬öCARD)C11WT-mCh.tif]

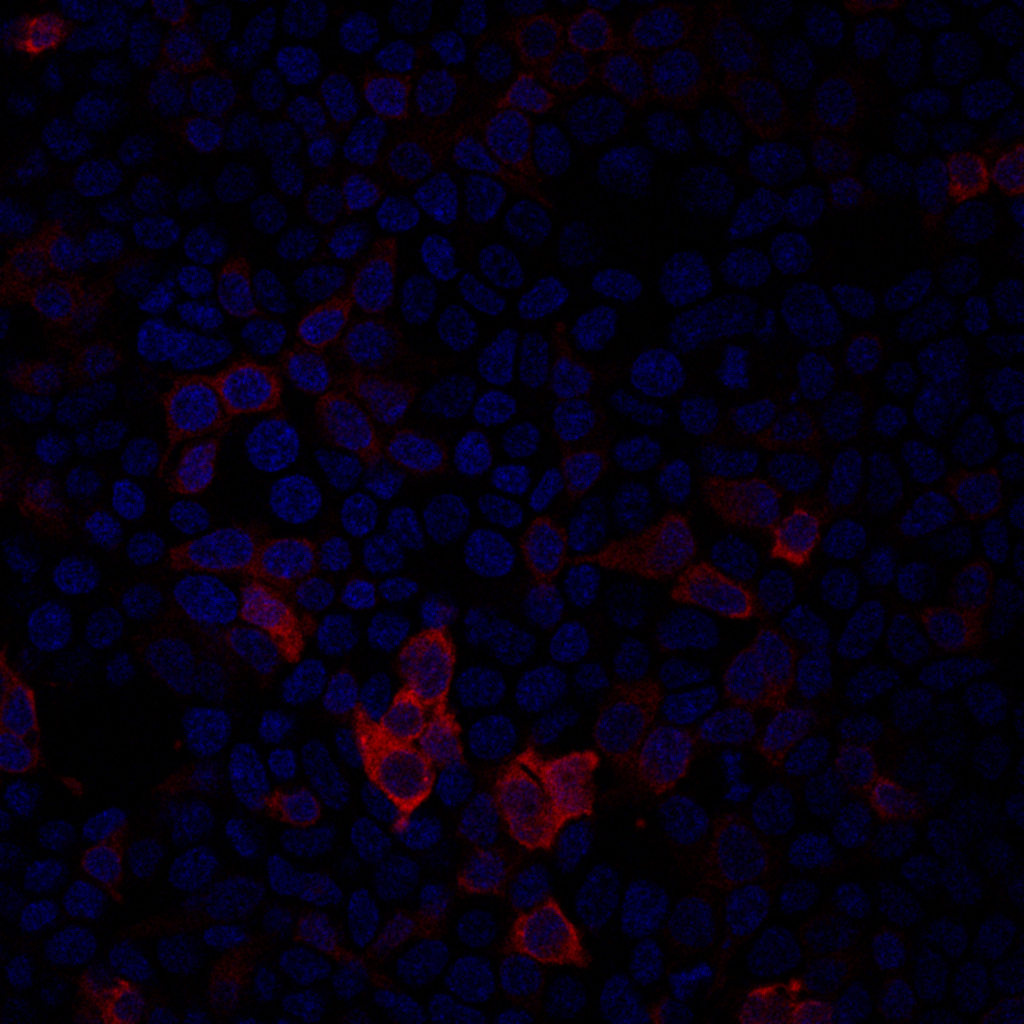

Supplement: Figure 6—figure supplement 2—source data 1. — HEK293T cells were transfected with indicated Casp11-mCherry constructs. 18 hr post-transfection, cells were fixed and prepared for microscopy. Nuclei were stained with Hoechst. [file elife-83725-fig6-figsupp2-data1.zip › (╬öCARD)C254A-mCh.tif]

Fig 6-figure supplement 2 Source Data 1 (6S2A)

CASP11-  
( $\Delta$ CARD)-mCh:

WT

C254A

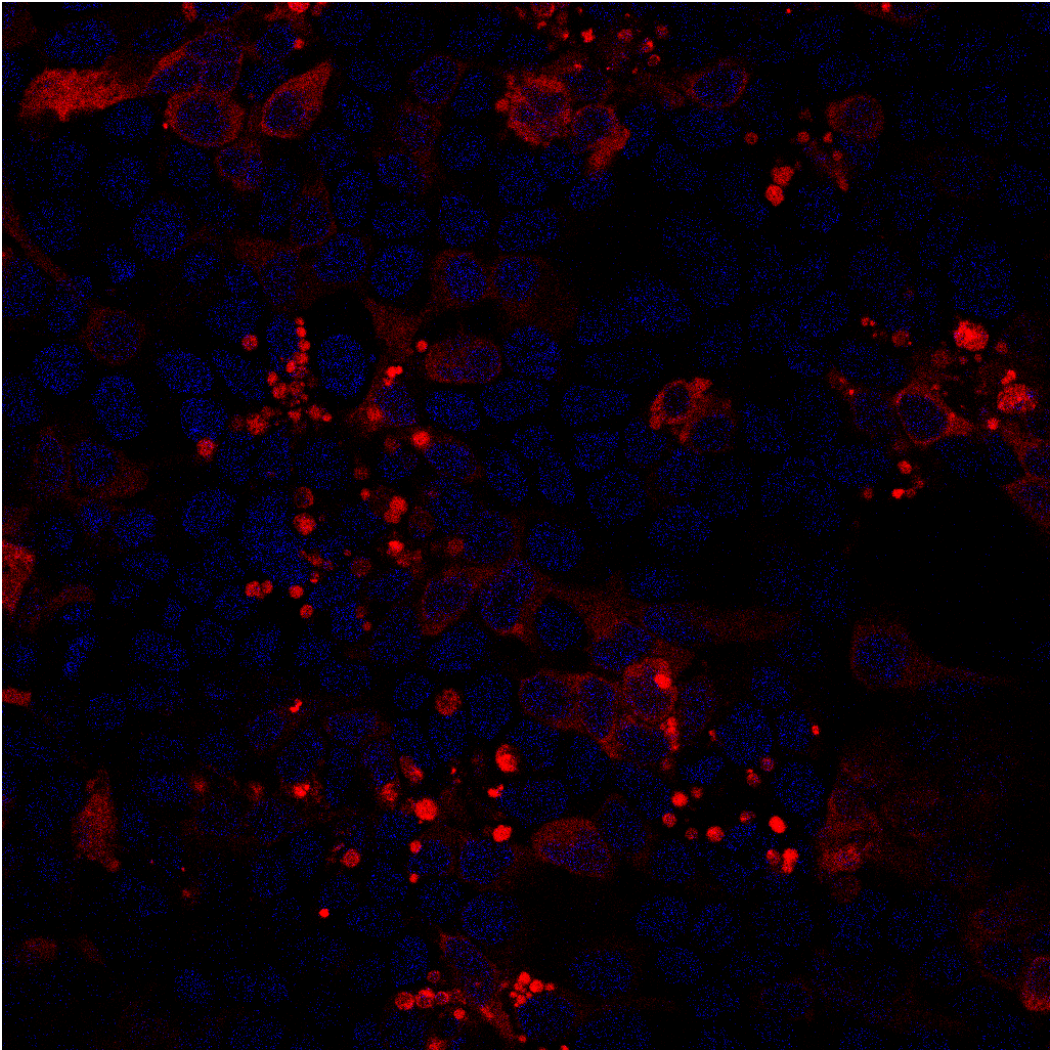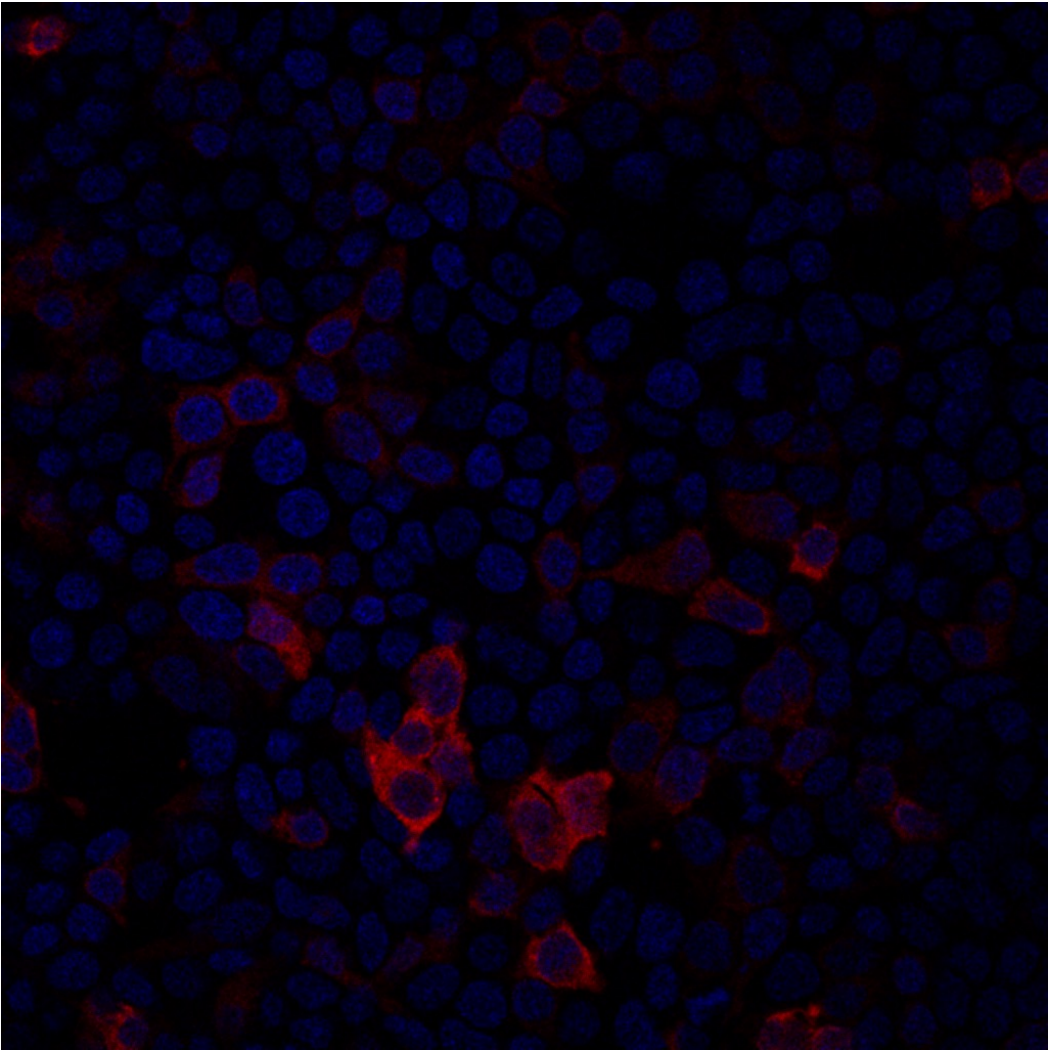

HOECHST mCHERRY

Supplement: Figure 6—figure supplement 2—source data 1. — HEK293T cells were transfected with indicated Casp11-mCherry constructs. 18 hr post-transfection, cells were fixed and prepared for microscopy. Nuclei were stained with Hoechst. [file elife-83725-fig6-figsupp2-data1.zip › Figure 6-figure supplement 2-source data 1.pdf]

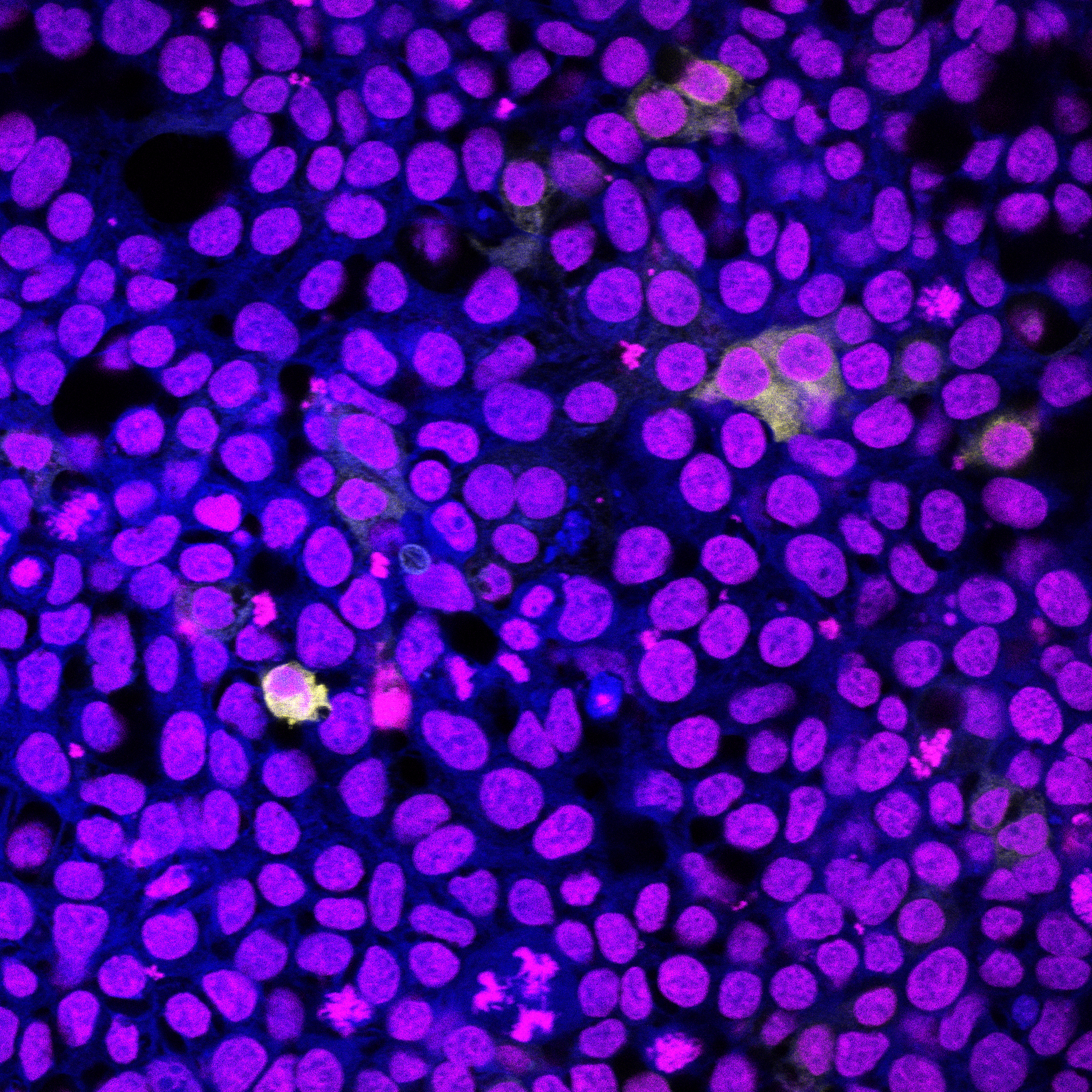

Supplement: Figure 6—figure supplement 2—source data 2. — The fluorescent reporter citrine was fused to the C-terminus of wild-type (WT) or CARD-only Casp11, transfected into HEK293T cells as indicated, and imaged by confocal microscopy 18 hr post-transfection. Nuclei (magenta) were stained with DRAQ7, cytosolic content (blue) was stained with cell tracer violet. [file elife-83725-fig6-figsupp2-data2.zip › C11(WT)-citrine_250.tif]

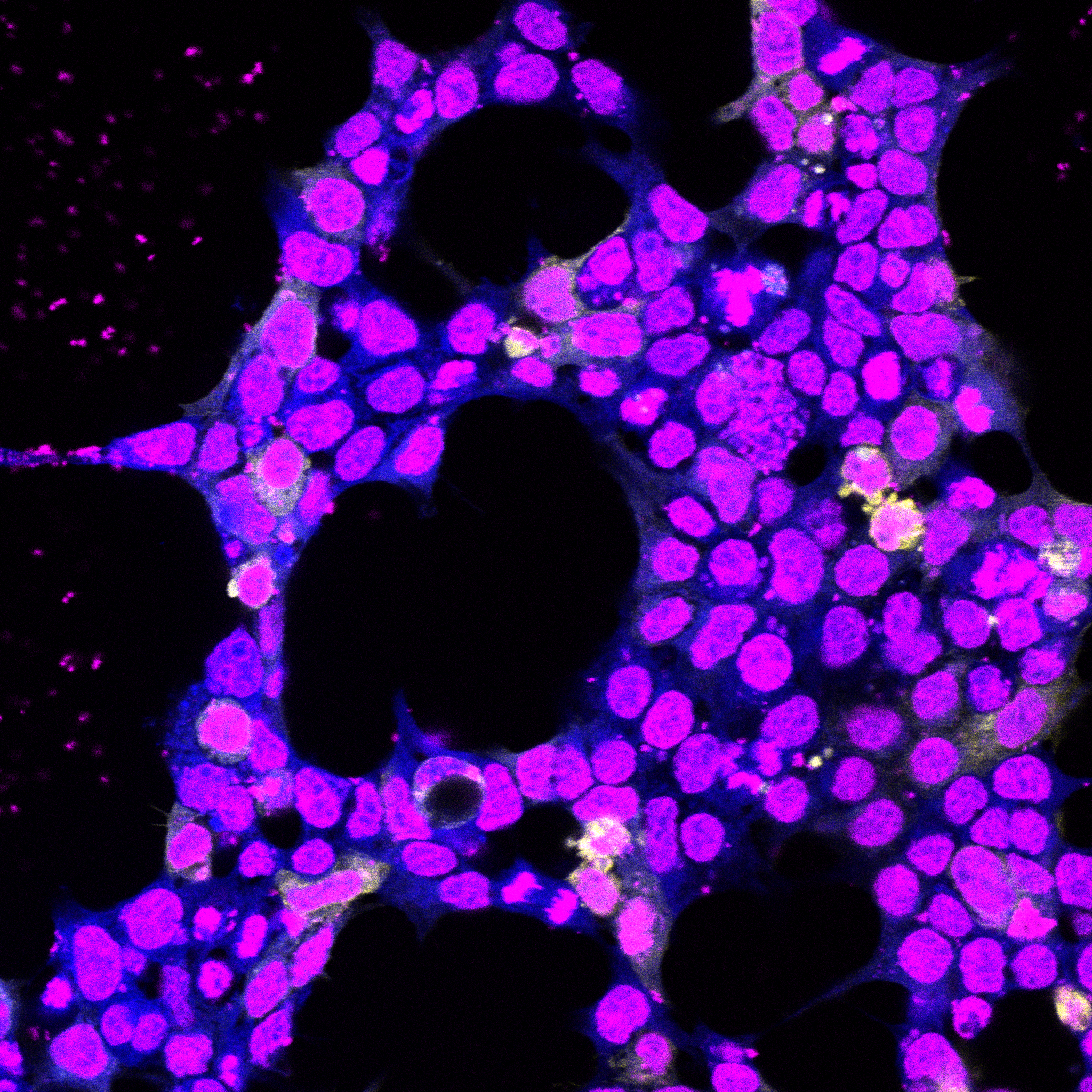

Supplement: Figure 6—figure supplement 2—source data 2. — The fluorescent reporter citrine was fused to the C-terminus of wild-type (WT) or CARD-only Casp11, transfected into HEK293T cells as indicated, and imaged by confocal microscopy 18 hr post-transfection. Nuclei (magenta) were stained with DRAQ7, cytosolic content (blue) was stained with cell tracer violet. [file elife-83725-fig6-figsupp2-data2.zip › C11(WT)-citrine_500.tif]

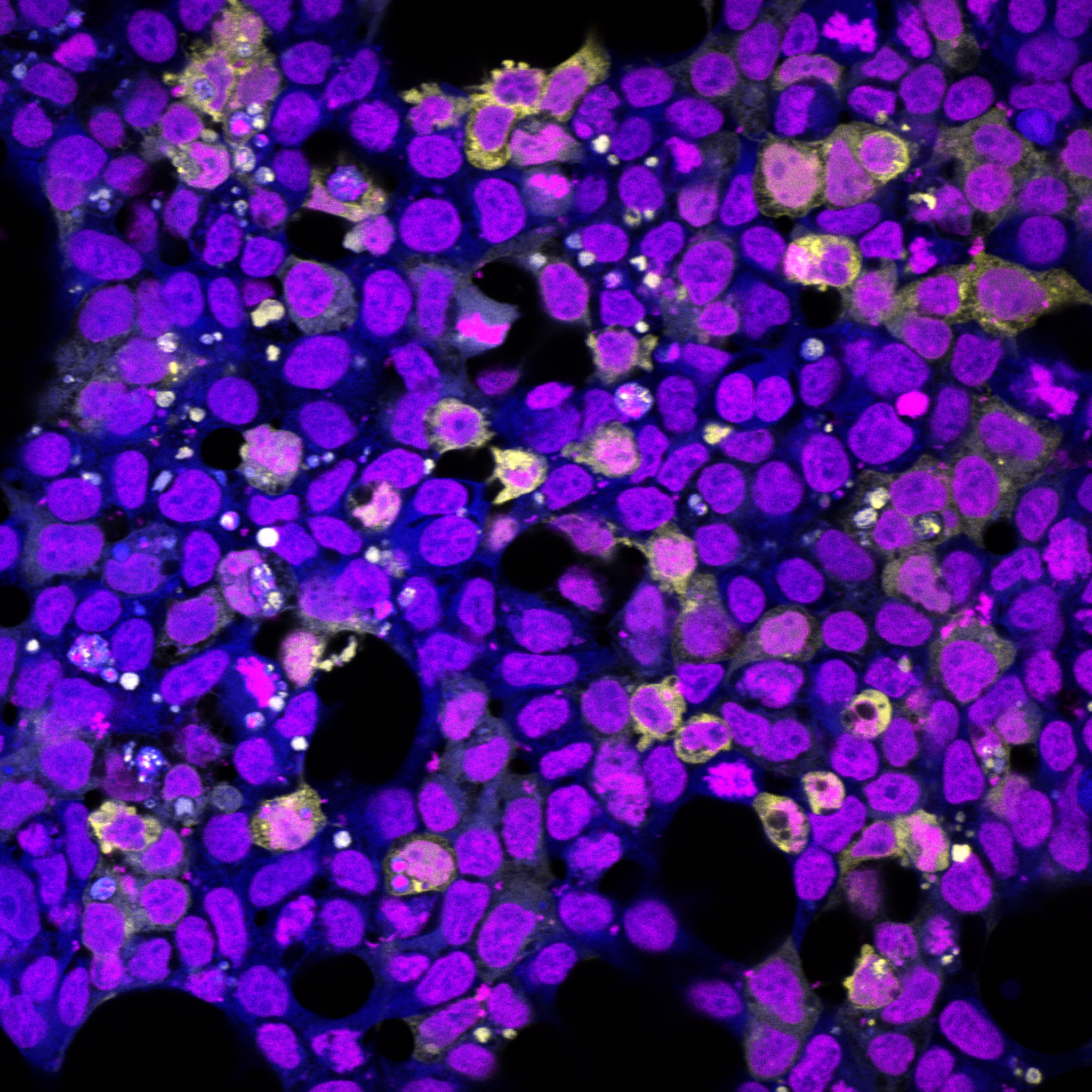

Supplement: Figure 6—figure supplement 2—source data 2. — The fluorescent reporter citrine was fused to the C-terminus of wild-type (WT) or CARD-only Casp11, transfected into HEK293T cells as indicated, and imaged by confocal microscopy 18 hr post-transfection. Nuclei (magenta) were stained with DRAQ7, cytosolic content (blue) was stained with cell tracer violet. [file elife-83725-fig6-figsupp2-data2.zip › C11(WT)-citrine_1000.tif]

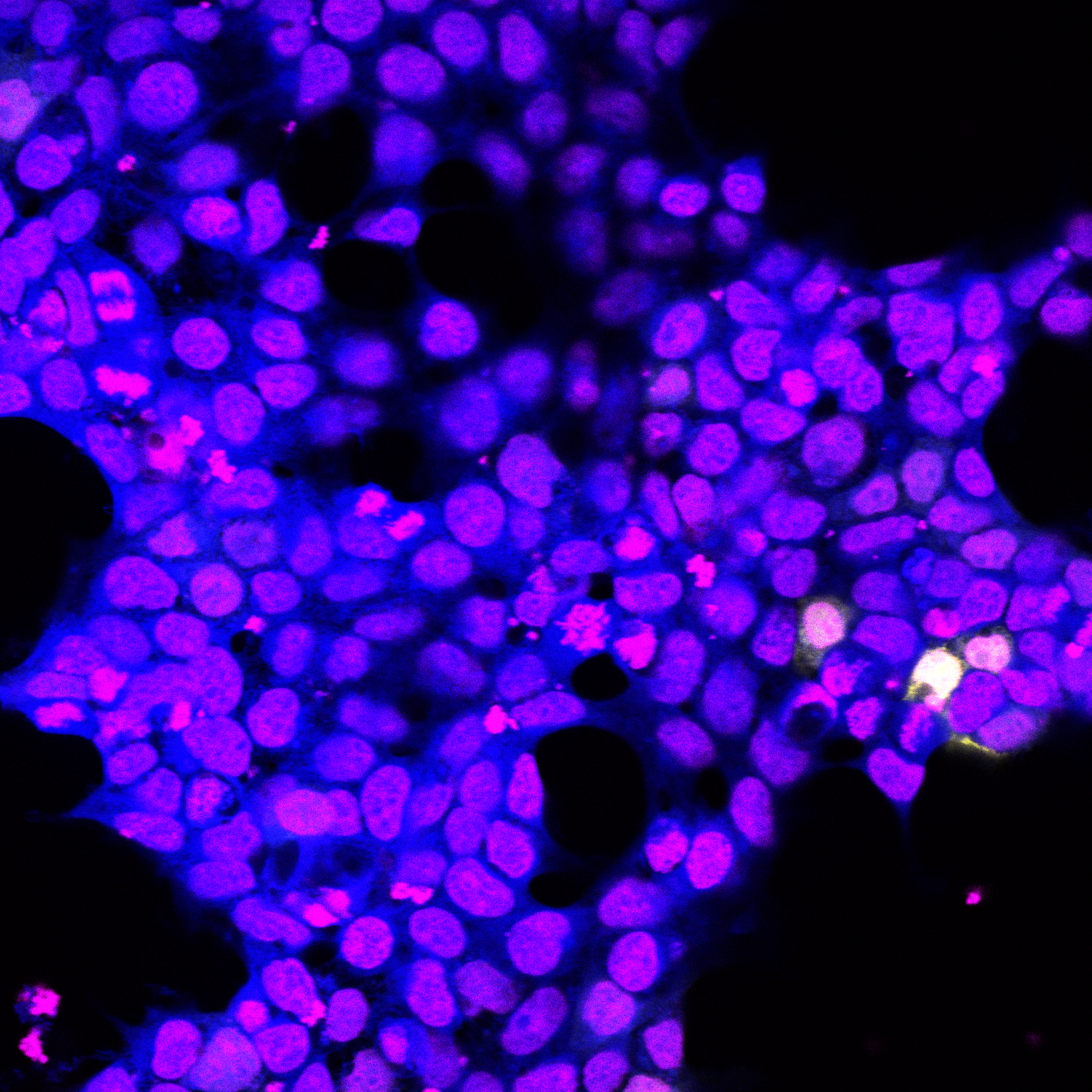

Supplement: Figure 6—figure supplement 2—source data 2. — The fluorescent reporter citrine was fused to the C-terminus of wild-type (WT) or CARD-only Casp11, transfected into HEK293T cells as indicated, and imaged by confocal microscopy 18 hr post-transfection. Nuclei (magenta) were stained with DRAQ7, cytosolic content (blue) was stained with cell tracer violet. [file elife-83725-fig6-figsupp2-data2.zip › CARD-citrine_250.tif]

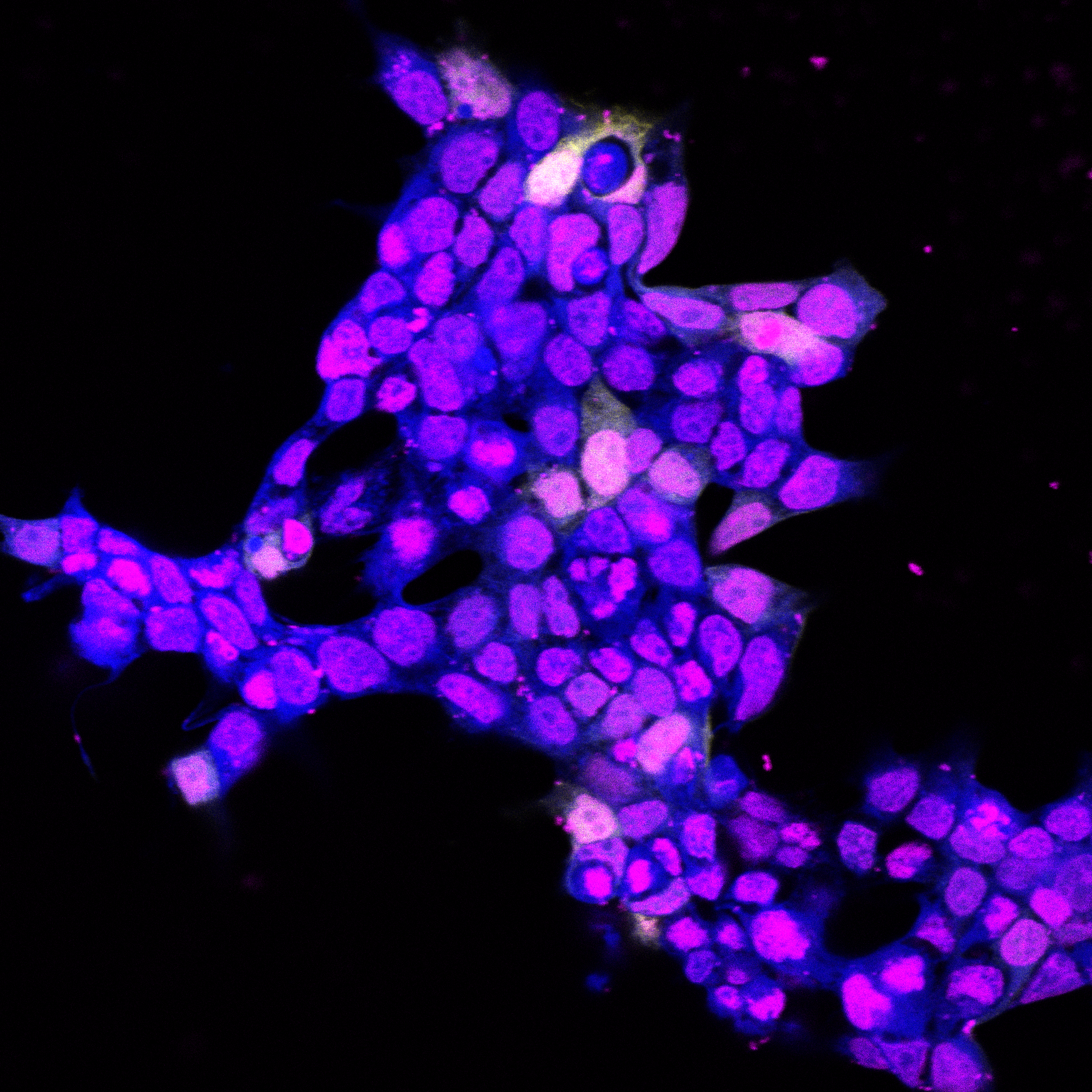

Supplement: Figure 6—figure supplement 2—source data 2. — The fluorescent reporter citrine was fused to the C-terminus of wild-type (WT) or CARD-only Casp11, transfected into HEK293T cells as indicated, and imaged by confocal microscopy 18 hr post-transfection. Nuclei (magenta) were stained with DRAQ7, cytosolic content (blue) was stained with cell tracer violet. [file elife-83725-fig6-figsupp2-data2.zip › CARD-citrine_500.tif]

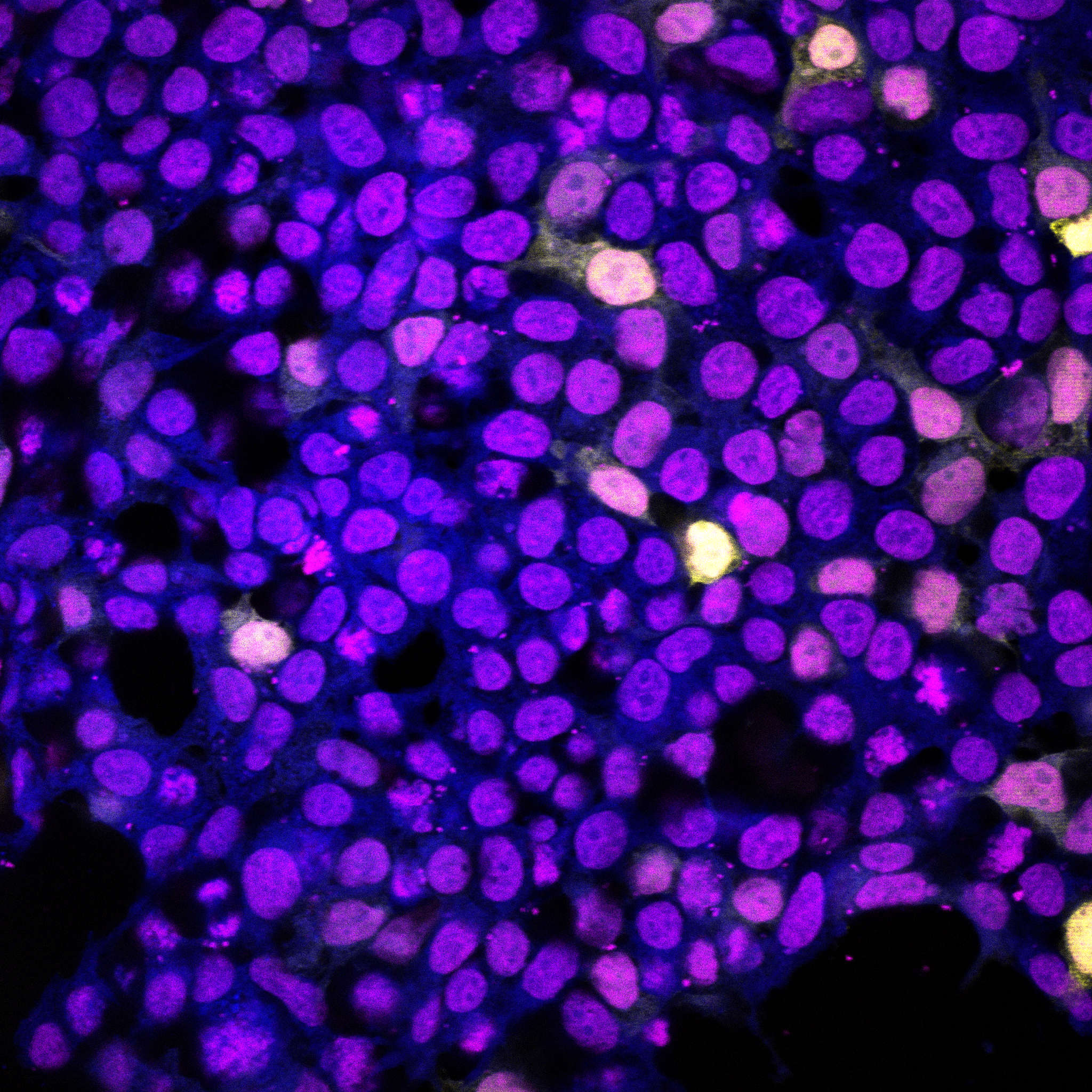

Supplement: Figure 6—figure supplement 2—source data 2. — The fluorescent reporter citrine was fused to the C-terminus of wild-type (WT) or CARD-only Casp11, transfected into HEK293T cells as indicated, and imaged by confocal microscopy 18 hr post-transfection. Nuclei (magenta) were stained with DRAQ7, cytosolic content (blue) was stained with cell tracer violet. [file elife-83725-fig6-figsupp2-data2.zip › CARD-citrine_1000.tif]

0 ng

250 ng

1000 ng

C11(WT)-  
Citrine

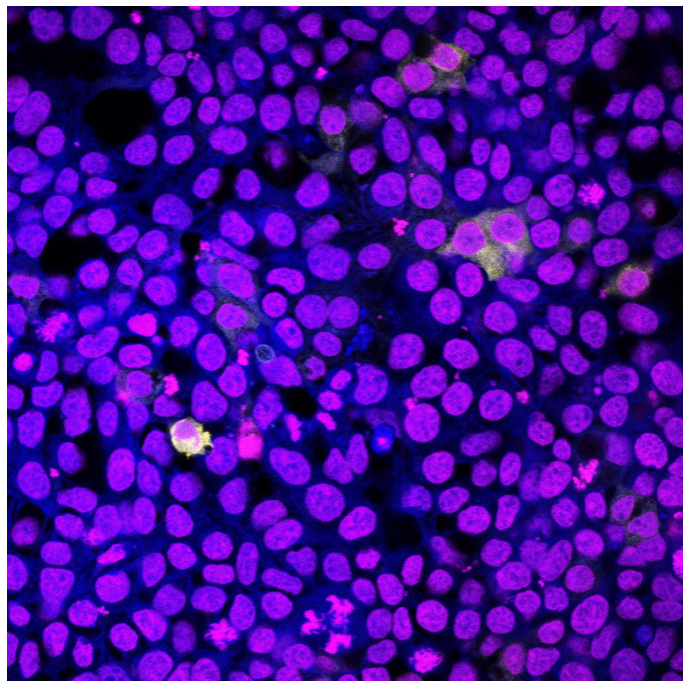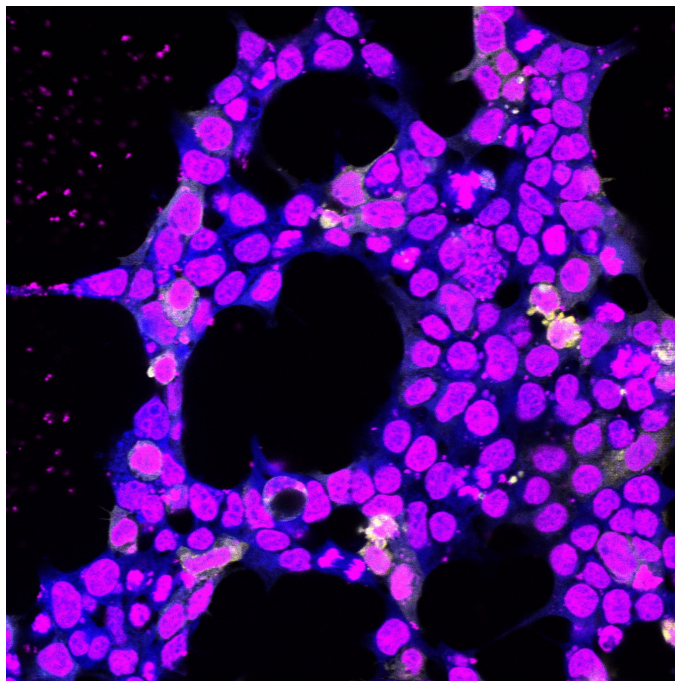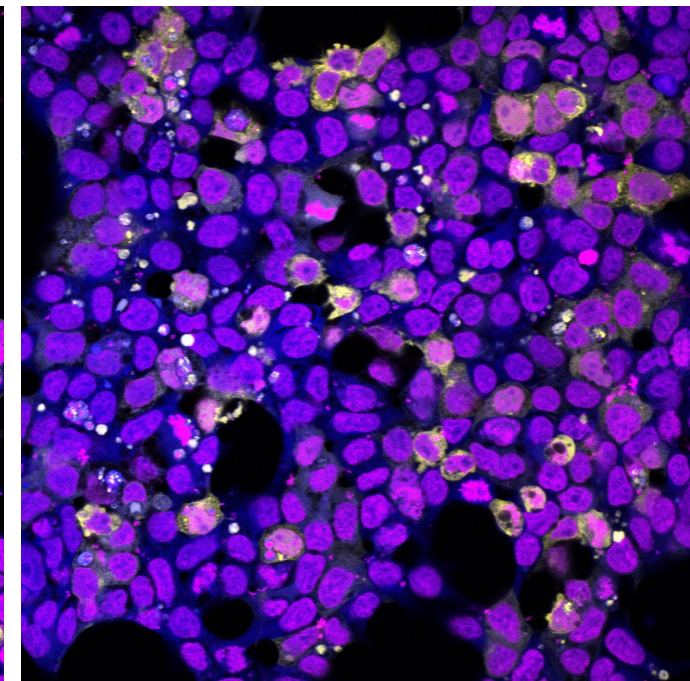

CARD-  
citrine

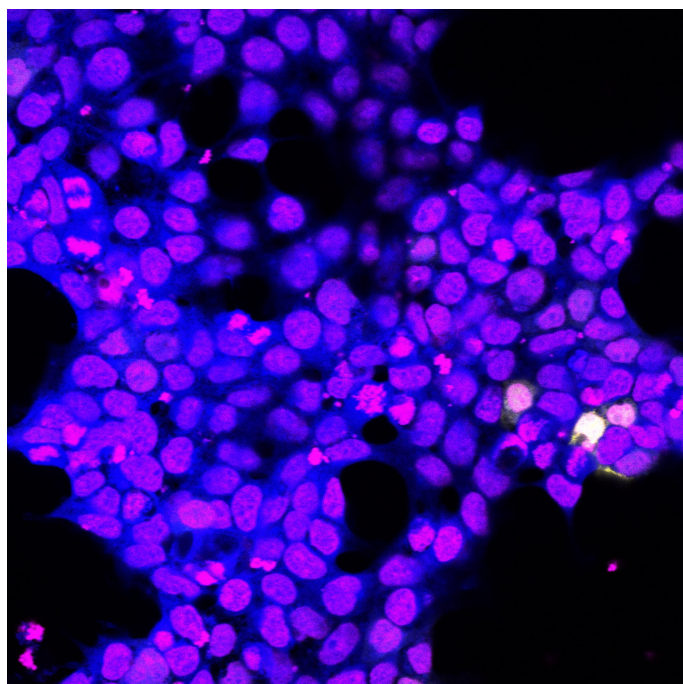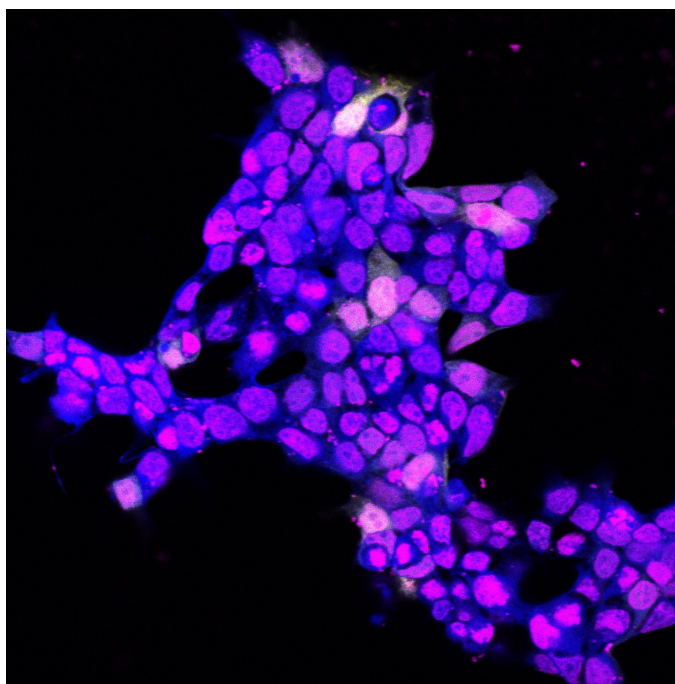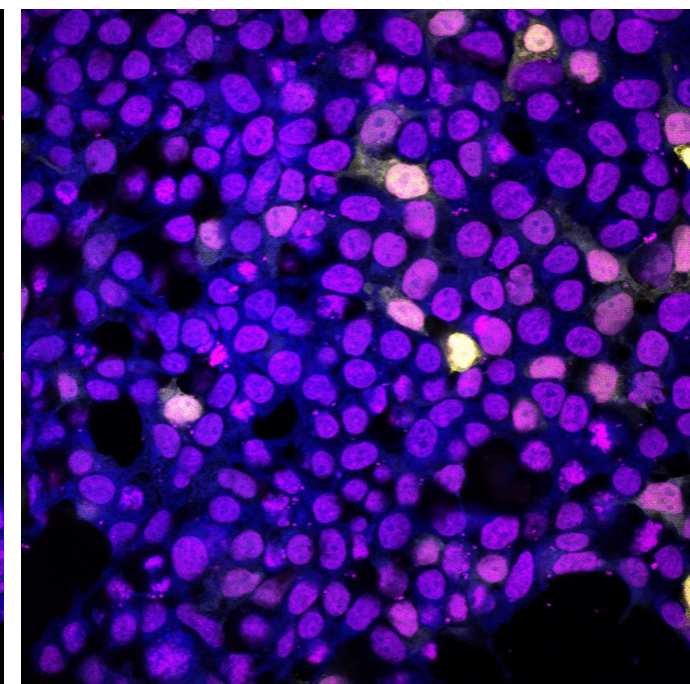

Citrine DRAQ7 Cell Mask

Supplement: Figure 6—figure supplement 2—source data 2. — The fluorescent reporter citrine was fused to the C-terminus of wild-type (WT) or CARD-only Casp11, transfected into HEK293T cells as indicated, and imaged by confocal microscopy 18 hr post-transfection. Nuclei (magenta) were stained with DRAQ7, cytosolic content (blue) was stained with cell tracer violet. [file elife-83725-fig6-figsupp2-data2.zip › Figure 6-figure supplement 2-source data 2.pdf]

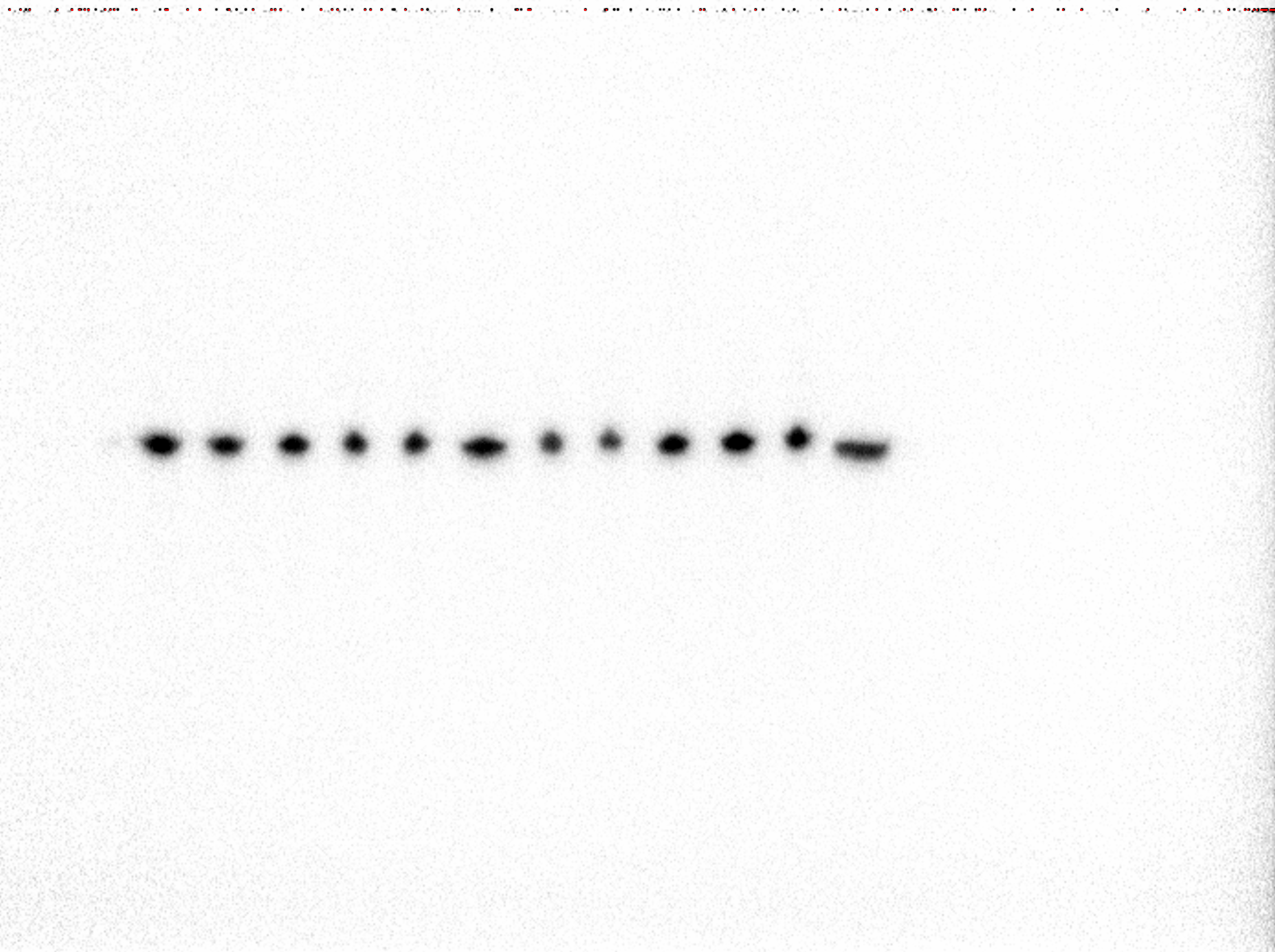

Supplement: Figure 7—source data 1. — HEK293T cells were transfected with the indicated TEV-cleavable Casp11-mCherry constructs, together with increasing doses of V5-TEV protease (0–500 ng/well). Whole-cell lysates were harvested 12 hr post-transfection and immunoblotted for mCherry or β-actin (loading control). [file elife-83725-fig7-data1.zip › Actin.tif]

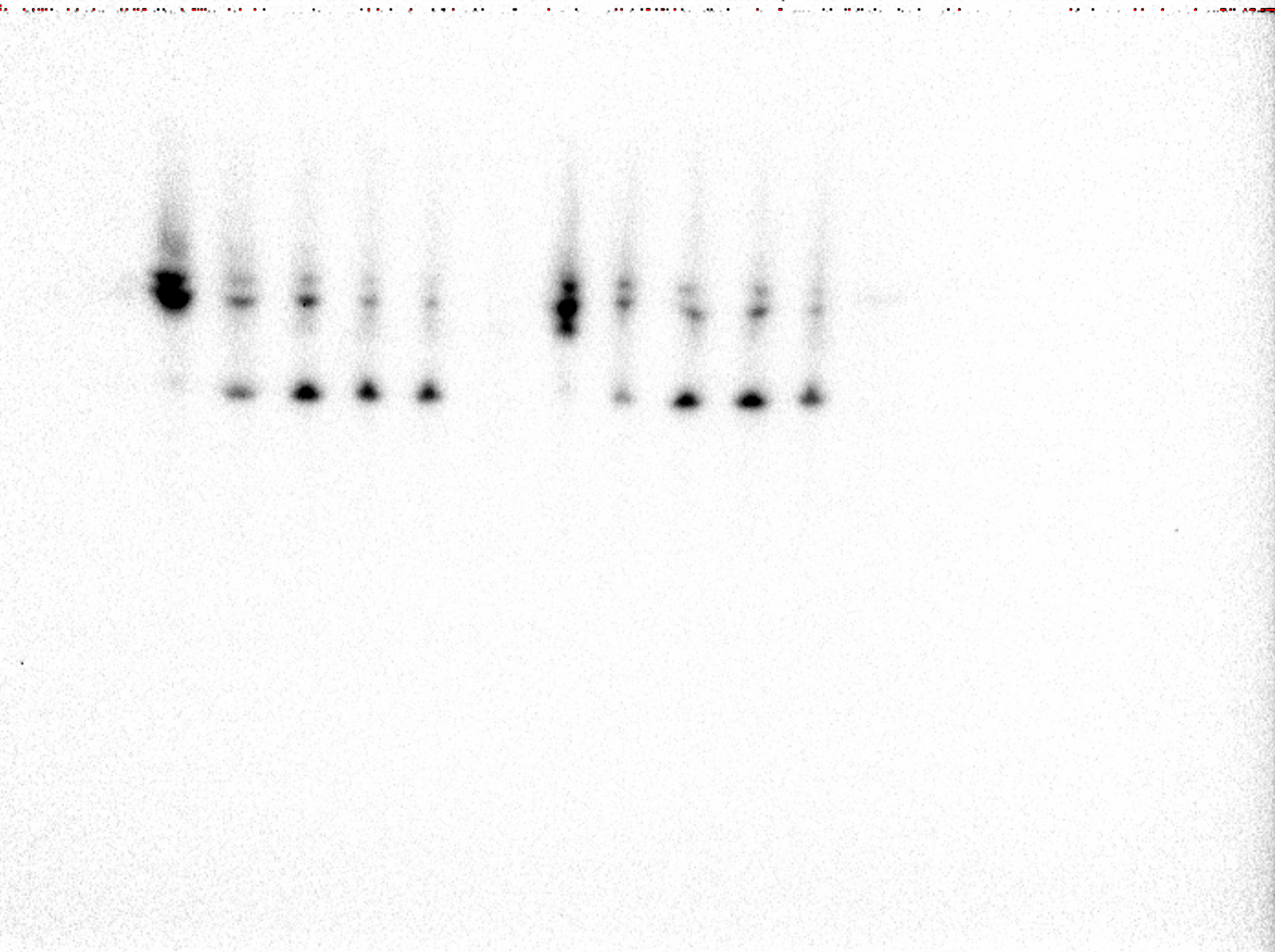

Supplement: Figure 7—source data 1. — HEK293T cells were transfected with the indicated TEV-cleavable Casp11-mCherry constructs, together with increasing doses of V5-TEV protease (0–500 ng/well). Whole-cell lysates were harvested 12 hr post-transfection and immunoblotted for mCherry or β-actin (loading control). [file elife-83725-fig7-data1.zip › Casp11(TEV)-mCh.tif]

7B.

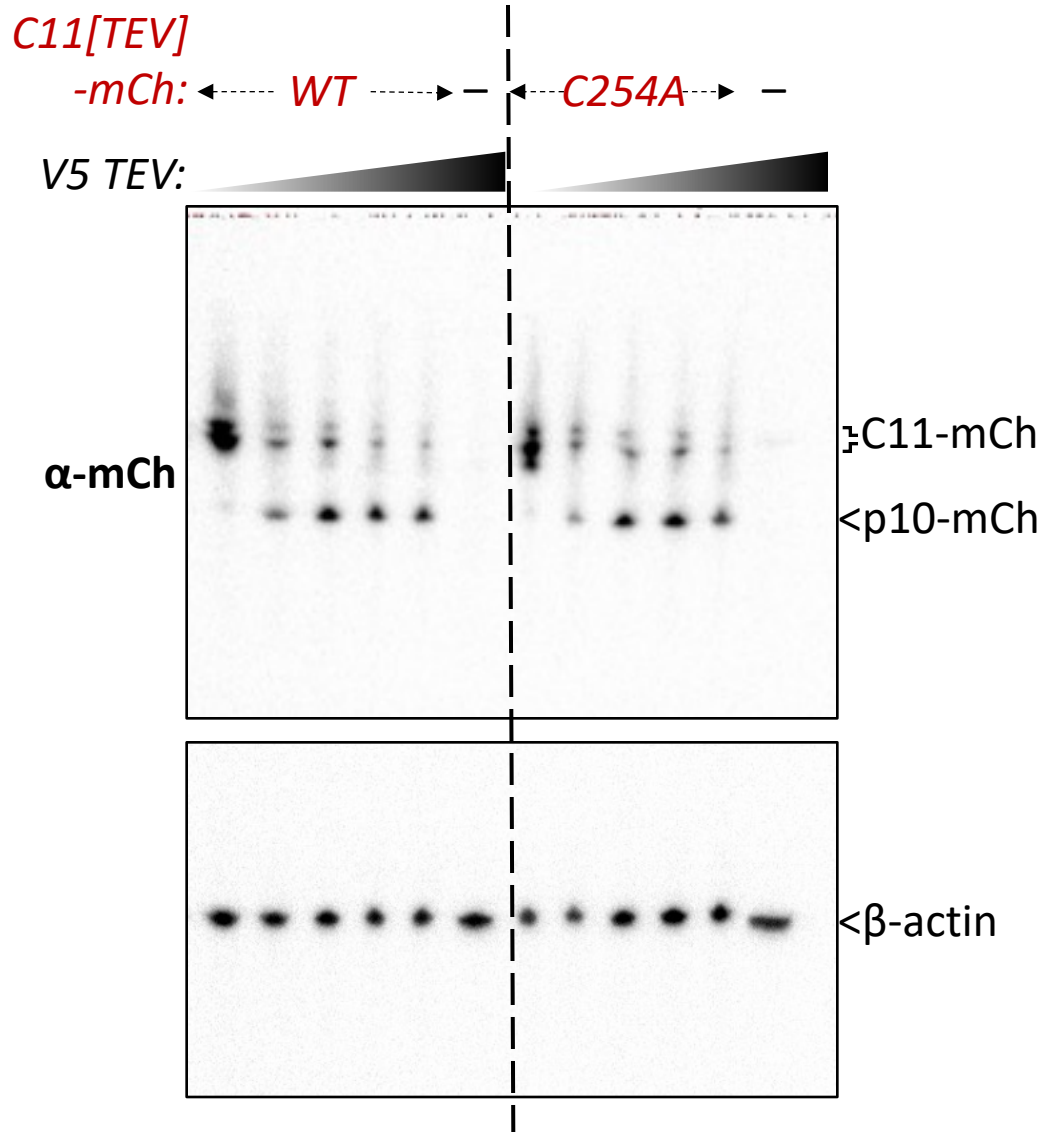

Supplement: Figure 7—source data 1. — HEK293T cells were transfected with the indicated TEV-cleavable Casp11-mCherry constructs, together with increasing doses of V5-TEV protease (0–500 ng/well). Whole-cell lysates were harvested 12 hr post-transfection and immunoblotted for mCherry or β-actin (loading control). [file elife-83725-fig7-data1.zip › Figure 7-source data 1.pdf]

## Slide 1
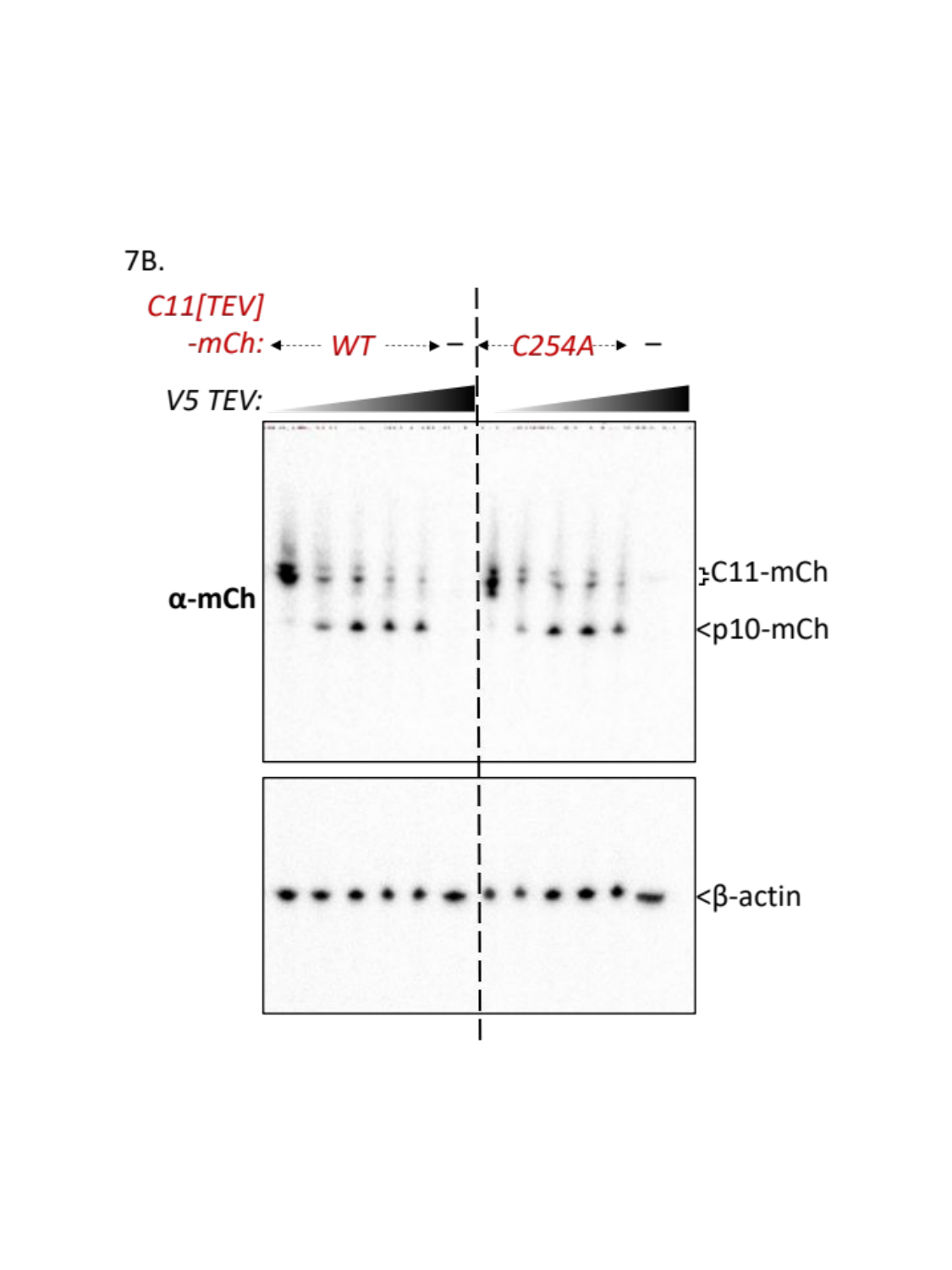

Supplement: Figure 7—source data 1. — HEK293T cells were transfected with the indicated TEV-cleavable Casp11-mCherry constructs, together with increasing doses of V5-TEV protease (0–500 ng/well). Whole-cell lysates were harvested 12 hr post-transfection and immunoblotted for mCherry or β-actin (loading control). [file elife-83725-fig7-data1.zip › Figure_7B_labeled.pptx]
