# Supplementary material for: Catalytic activity and autoprocessing of murine caspase-11 mediate noncanonical inflammasome assembly in response to cytosolic LPS
Source: eLife. 2024 Jan 17;13:e83725. doi: 10.7554/eLife.83725 (PMC10794067; doi:10.7554/eLife.83725)
Supplement: Figure 5—source data 3. — Speck formation in (C) was quantified as percentage of mCherry-expressing cells containing at least one speck. Dose–response curves were plotted by least-squares nonlinear regression ([Log2(agonist) vs. response (three parameters)]; Y = Bottom + (Top-Bottom)/(1 + 10(LogEC50-X))). [file elife-83725-fig5-data3.zip › Figure 5-source data 3.pdf]

		% Speck formation								
**Untagged Plasmid amount (ng)**	**Log(2) (untagged plasmid)**	**WT**			**C254A**			**D285A**		
0	4.96578428	1.571709234	2.419355	2.514507	0.680272	0	0.980392	1.571709	2.419355	2.514507
62.5	5.96578428	3.374233129	5.186972	2.992126	2.232143	0.406504	0	1.646707	1.273885	1.419558
125	6.96578428	3.85126162	4.489338	5.483029	0	0.961539	0.995025	2.725564	0.56243	1.770833
250	7.96578428	8.600182983	6.13563	9.579439	0	0	0	0.943396	2.358491	1.079914
500	8.96578428	13.16489362	16.88742	14.00264	1.413428	0	0.904977	5.194805	4.295943	5.03413

**Statistics**	**WT**	**C254A**	**D285A**
Log(agonist) vs. response (three parameters)			
Y=Bottom + (Top-Bottom)/(1+10^(LogEC50-X))			
Best-fit values			
Bottom	3.208	0.7263	1.614
Top	16.51	0.4167	3024
LogEC50	8.176	6.967	11.94
EC50	150112404	9269616	8.77E+11
Span	13.3	-0.3096	3022
95% CI (profile likelihood)			
Bottom	2.067 to 4.327	0.1138 to 0.955	1.144 to 2.084
Top	13.79 to 20.87	??? to 0.9551	???
LogEC50	7.791 to 8.641	???	8.537 to ???
EC50	61806323 to 4	???	344026539 to ???
Goodness of Fit			
Degrees of Freedom	12	12	12
R squared	0.9235	0.03661	0.7855
Sum of Squares	24.08	6.167	6.326
Sy.x	1.417	0.7169	0.726
Number of points			
# of X values	15	15	15
# Y values analyzed	15	15	15
