# Supplementary material for: Catalytic activity and autoprocessing of murine caspase-11 mediate noncanonical inflammasome assembly in response to cytosolic LPS
Source: eLife. 2024 Jan 17;13:e83725. doi: 10.7554/eLife.83725 (PMC10794067; doi:10.7554/eLife.83725)
Supplement: Figure 5—source data 4. — HEK293T cells stably expressing human gasdermin D were transiently transfected with a fixed dose of empty plasmid (Veh) OR mCherry-tagged C254A caspase-11, plus increasing doses of unlabeled wild-type (WT), catalytically inactive (C254A), or non-cleavable (D285A) caspase-11 constructs. Cytotoxicity was measured as percent lactate dehydrogenase (LDH) release 18 hr post-plasmid transfection (with respect to 1% Triton X-100-induced cytotoxicity). Dose–response curves were plotted by least-squares nonlinear regression ([Log2(agonist) vs. response (three parameters)]; Y = Bottom + (Top-Bottom)/(1 + 10(LogEC50-X)); R2 indicated). [file elife-83725-fig5-data4.zip › Figure 5-source data 4.pdf]

% Cytotoxicity (% LDH release)

Untagged Plasmid amount (ng)	Log(2) (untagged plasmid)	WT C11, Veh						WT C11, C254A-mCh					
0	4.96578428	1.83217119	-2.1639817	0.33181053	0.97280919	-0.4266007	-0.5462084	2.76559865	0.90098771	-3.6665864	-0.9029985	0.96592518	-0.0629267
62.5	5.96578428	1.74561193	-1.8033181	7.48737677	3.68790368	4.11849135	0.15947692	9.27005541	4.55793785	0.71308119	4.92086965	5.10964981	5.5532832
125	6.96578428	2.59677807	-0.8944458	5.43880741	8.10142732	5.31456822	3.23339447	7.89689232	8.21488798	1.94170079	5.19460089	5.79869742	5.00582072
250	7.96578428	8.05001202	2.43808608	11.8586199	11.7255402	7.45554581	7.1924089	12.7535534	13.7219947	4.38448567	7.50715791	9.41383759	8.61152188
500	8.96578428	5.8860303	4.86174561	9.30512142	14.9908301	11.9288733	11.1992664	12.8402795	12.3777403	14.5892556	10.3954944	8.97964321	9.7725199

		C254A C11, C254A-mCh						D285A C11, C254A-mCh					
0	4.96578428	0.84051096	-3.4036533	2.56314235	-0.1960784	-0.3657617	0.56184012	-0.887574	1.19779399	-0.31022	0.89048409	1.38797392	-2.278458
62.5	5.96578428	3.81142596	-0.6699122	4.84750135	2.50754148	-0.6711916	5.4147813	2.67133912	4.24829092	2.47314299	3.52104677	1.21760069	-0.9222871
125	6.96578428	2.45079682	-0.607498	4.63529314	3.71794872	-3.5218703	5.1546003	4.15350146	4.27414259	4.73947263	6.45146635	3.85497831	3.14622567
250	7.96578428	1.66437815	-2.4050264	2.21362294	4.80392157	0.50527903	5.65233786	4.78255874	2.77474579	7.85029011	7.8621567	6.92169646	7.38511165
500	8.96578428	4.08604835	2.41334831	4.38563642	4.46455505	1.26319759	6.51206637	4.22243925	2.71442523	9.59958637	10.3223462	8.3528316	8.5163899

Statistics	WT C11, Veh	WT C11, C254A-mCh	C254A C11, C254A-mCh	D285A C11, C254A-mCh
Log(agonist) vs. response (three parameters)				
Y=Bottom + (Top-Bottom)/(1+10^(LogEC50-X))				
Best-fit values				
Bottom	0.9465	1.871	-14987	0.3077
Top	9.666	10.98	2.683	6.86
LogEC50	7.217	7.026	1.218	6.618
EC50	16472139	10607528	16.51	4153753
Span	8.72	9.112	14989	6.553
95% CI (profile likelihood)				
Bottom	-1.322 to 2.938	-4.248 to 3.969	--	-2.114 to 1.910
Top	7.367 to 12.63	8.177 to 13.80	--	5.517 to 8.342
LogEC50	6.339 to 8.021	5.412 to 7.897	--	5.773 to 7.367
EC50	2183340 to 10[illegible]	258390 to 788[illegible]	--	593140 to 23256880
Goodness of Fit				
Degrees of Freedom	27	27	--	27
R squared	0.587	0.6357	--	0.653
Sum of Squares	258.6	232.6	--	109
Sy.x	3.095	2.935	--	2.009
Number of points				
# of X values	30	30	30	30
# Y values analyzed	30	30	30	30
