# Supplementary material for: Catalytic activity and autoprocessing of murine caspase-11 mediate noncanonical inflammasome assembly in response to cytosolic LPS
Source: eLife. 2024 Jan 17;13:e83725. doi: 10.7554/eLife.83725 (PMC10794067; doi:10.7554/eLife.83725)
Supplement: Figure 7—source data 2. — HEK293T cells from (B) were imaged by fluorescence microscopy and speck formation was quantified as percentage of Casp11-mCherry-expressing cells containing at least one speck. Dose–response curves were plotted by least-squares nonlinear regression ([Log2(agonist) vs. response (three parameters)]; Y = Bottom + (Top-Bottom)/(1 + 10(LogEC50-X)); R2 indicated). [file elife-83725-fig7-data2.zip › Figure 7-source data 2.pdf]

		% Speck formation					
V5-TEV protease plasmid amount (ng)	Log(2) (V5-TEV)	WT(TEV)-mCh			C254A(TEV)-mCh		
0	4.96578428	4.787234	5.274725	3.21285	7.575758	10.625	10.49383
62.5	5.96578428	11.842105	2.212389	10.0877	8.219178	13.31058	6.808511
125	6.96578428	12.598425	6.060606	6.42202	11.68831	12.80788	4.511278
250	7.96578428	26.623377	15.28926	15.8046	8.490566	11.19048	5.882353
500	8.96578428	23.214286	14.36782	19.1176	6.818182	6.077348	1.824818

Statistics	WT(TEV)-mCh	C254A(TEV)-mCh
Log(agonist) vs. response (three parameters)		
Y=Bottom + (Top-Bottom)/(1+10^(LogEC50-X))		
Best-fit values		
Bottom	5.484	9.596
Top	20.31	1.964
LogEC50	7.339	8.763
EC50	21846686	579593975
Span	14.83	-7.632
95% CI (profile likelihood)		
Bottom	1.127 to 9.415	7.600 to 11.86
Top	15.45 to 26.19	??? to 8.468
LogEC50	6.434 to 8.122	5.498 to ???
EC50	2716447 to 132	314544 to ???
Goodness of Fit		
Degrees of Freedom	12	12
R squared	0.6871	0.3303
Sum of Squares	238.2	98.66
Sy.x	4.455	2.867
Number of points		
# of X values	15	15
# Y values analyzed	15	15
