# Supplementary material for: Catalytic activity and autoprocessing of murine caspase-11 mediate noncanonical inflammasome assembly in response to cytosolic LPS
Source: eLife. 2024 Jan 17;13:e83725. doi: 10.7554/eLife.83725 (PMC10794067; doi:10.7554/eLife.83725)
Supplement: Supplementary file 1. [file elife-83725-supp1.docx]

**Supplementary file 1a: Plasmids**

| **Plasmid** | **Vector Backbone** | **Antibiotic Selection** | **Tag** | **Source** |
| --- | --- | --- | --- | --- |
| Casp11-mCh | pReceiver-M56 | Amp, Neo | C-mCherry | Genecopoeia Inc. |
| Casp11(C254A)-mCh | pReceiver-M56 | Amp, Neo | C-mCherry | This paper |
| Casp11(D285A)-mCh | pReceiver-M56 | Amp, Neo | C-mCherry | This paper |
| Casp11(C254A/D285A)-mCh | pReceiver-M56 | Amp, Neo | C-mCherry | This paper |
| pLenti-Casp11-mCh | pTwist-Lenti-SFFV-Puro-WPRE | Amp, Puro | C-mCherry | Twist Biosciences |
| pLenti-Casp11(C254A)-mCh | pTwist-Lenti-SFFV-Puro-WPRE | Amp, Puro | C-mCherry | Twist Biosciences |
| pLenti-Casp11(D285A)-mCh | pTwist-Lenti-SFFV-Puro-WPRE | Amp, Puro | C-mCherry | Twist Biosciences |
| pcDNA-Casp11 | pcDNA3.1 | Amp, Neo | -- | This paper |
| pcDNA-Casp11 (C254A) | pcDNA3.1 | Amp, Neo | -- | This paper |
| pcDNA-Casp11 (D285A) | pcDNA3.1 | Amp, Neo | -- | This paper |
| FL-GSDMD | pcDNA3.1 | Amp, Neo | N-Flag | Judy Lieberman (Addgene) |
| DmrB-(ΔCARD)-Casp11-FL-mCh | pLX307 | Amp, Puro | C-Flag-mCherry | This paper |
| DmrB-(ΔCARD)-Casp11(C254A)-FL-mCh | pLX307 | Amp, Puro | C-Flag-mCherry | This paper |
| pLenti-Casp11[TEV]-mCh | pTwist-Lenti-SFFV-Puro-WPRE | Amp, Puro | C-mCherry | Twist Biosciences |
| pLenti-Casp11(C254A)[TEV]-mCh | pTwist-Lenti-SFFV-Puro-WPRE | Amp, Puro | C-mCherry | Twist Biosciences |
| TEV protease | pCDNA3.1 | Amp, Neo | N-V5 | Andrew Oberst (Addgene) |
| pLenti-Casp11-Citrine | pTwist-Lenti-SFFV-Puro-WPRE | Amp, Puro | C-Citrine | Twist Biosciences |
| pLenti-CARD11-Citrine | pTwist-Lenti-SFFV-Puro-WPRE | Amp, Puro | C-Citrine | Twist Biosciences |
| 2xFlag-Casp11 | pLX307 | Amp, Puro | N-2xFlag | This paper |
| 2xFlag-Casp11(C254A) | pLX307 | Amp, Puro | N-2xFlag | This paper |
| Flag-GFP | pLX307 | Amp, Puro | N-Flag | This paper |
| (ΔCARD)Casp11-mCh | pReceiver-M56 | Amp, Neo | C-mCherry | This paper |
| (ΔCARD)Casp11(C254A)-mCh | pReceiver-M56 | Amp, Neo | C-mCherry | This paper |
| Casp11-Citrine | pTwist-Lenti-SFFV-Puro-WPRE | Amp, Puro | C-Citrine | Twist Biosciences |
| CARD11-Citrine | pTwist-Lenti-SFFV-Puro-WPRE | Amp, Puro | C-Citrine | Twist Biosciences |

**Supplementary file 1b: Oligonucleotides**

| **Gene** | **Mutation** | **Forward** | **Reverse** |
| --- | --- | --- | --- |
| Casp11 | C254A | TGTGCAGGCCGCCAGAGGTGGGA | ATGATGACTTTGGGTTTGTCTC |
| Casp11 | D285A | TATGGAAGCTGCTGCTGTCAAGC | TTCCTAGGTAGATCTACACC |
| Casp11 | ΔCARD | CCAGGCAGCCACCATGGT | CATGGTACCGAATTCCTTCAAGCC |

**Supplementary file 1c: Cell lines**

| **Cell Line** | **Source** |
| --- | --- |
| HEK293T | ATCC |
| HEK293T/2X-FLAG-Casp11 | This paper |
| HEK293T/2X-FLAG-Caps11(C254A) | This paper |
| HEK293T/hGSDMD | This paper |
