# Supplementary material for: Catalytic activity and autoprocessing of murine caspase-11 mediate noncanonical inflammasome assembly in response to cytosolic LPS
Source: eLife. 2024 Jan 17;13:e83725. doi: 10.7554/eLife.83725 (PMC10794067; doi:10.7554/eLife.83725)
Supplement: Figure 2—figure supplement 3—source data 1. — HEK293T cells were transiently transfected with 2X-FLAG-tagged wild-type (WT) or catalytically inactive Casp11 expression plasmids alongside WT or C254A mCherry-tagged Casp11 (5 μg). 48 hr post-transfection, whole-cell lysates were immunoprecipitated by anti-FLAG antibodies as described in ‘Materials and methods,’ and immunoblotted for mCherry, FLAG, or GAPDH as a loading control, as indicated. [file Figure 2-figure supplement 3-source data 1.pdf › Figure 2-figure supplement 3-source data 1.pdf]

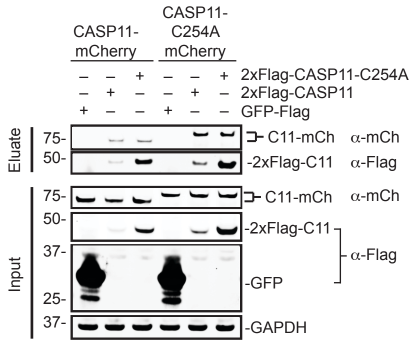
CASP11-mCherry
CASP11-C254A mCherry
2xFlag-CASP11-C254A
2xFlag-CASP11
GFP-Flag
Eluate
Input
75-
50-
37-
25-
C11-mCh
2xFlag-C11
GFP
GAPDH
α-mCh
α-Flag

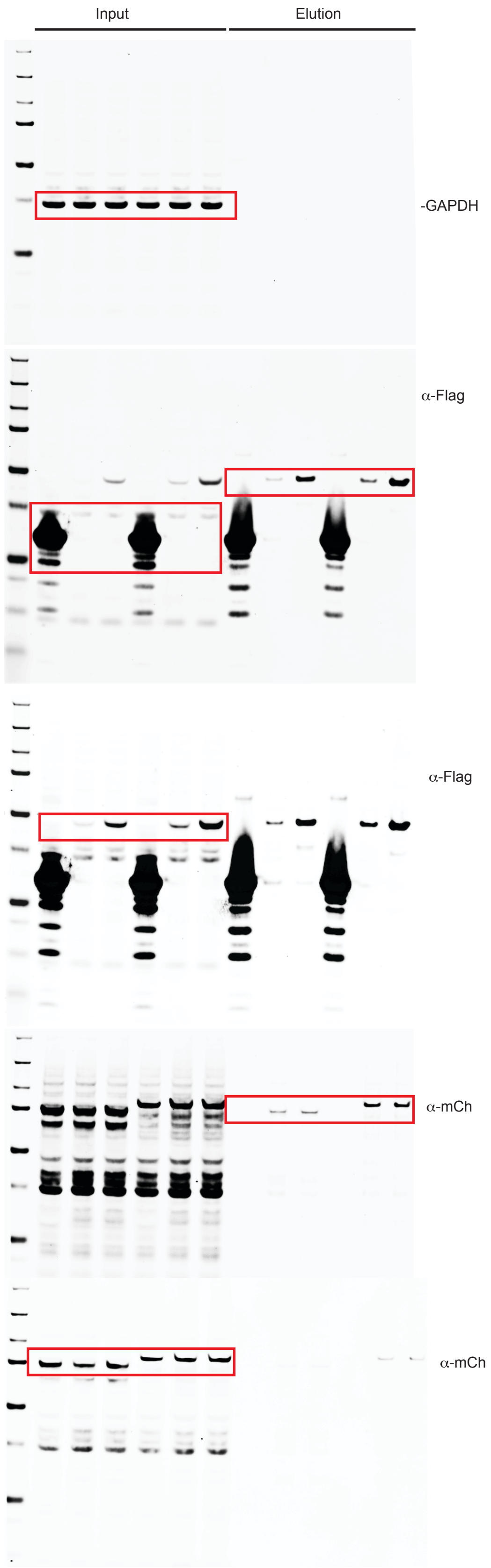
Input
Elution
-GAPDH
α-Flag
α-Flag
α-mCh
α-mCh
